# Supplementary material for: Enantioselective Cyclopropanation Catalyzed by Gold(I)-Carbene Complexes
Source: Molecules. 2022 Sep 7;27(18):5805. doi: 10.3390/molecules27185805 (PMC9501563; doi:10.3390/molecules27185805)
Supplement: Supplementary file 1 [file molecules-27-05805-s001.zip › molecules-1873281-supplementary final.pdf]

Supporting Information

Enantioselective cyclopropanation catalyzed by gold(I)-carbene complexes

Zita Szabo[1], Sophia Ben Ahmed[1], Zoltan Nagy[1], Attila Paczal[1,*], Andras Kotschy[1,*]

[1] Servier Research Institute of Medicinal Chemistry, Záhony u. 7., H-1031 Budapest, Hungary

Table of Contents

1. General information ... 8

2. Guide to the Carbene Ligands **A1-A42** present in Gold(I) Complexes **C1-C42** ... 9

3. Experimental Section ... 11

4. General Procedure for the Preparation of *N,N*-Disubstituted 4,5-Dihydro-1*H*-imidazol-3-ium Chloride Derivatives ... 11

5. General Procedure for Preparation of Silver(I)-carbene Complexes (**B6**, **B15-32**, **B35-40**) ... 12

6. General Procedure for Preparation of Gold(I)-carbene Complexes by Transmetallation (**C6**, **C15-32**, **C35-40**) or Direct Metallation (**C5**, **C41-42**) ... 21

7. Preparation of Acetylene Derivatives (**1a-d**) ... 30

8. General Procedure for Preparation of Substituated Cyclopropyl Ring (**3a-t, 4a-e,g-j,l,o,q-t**) ... 31

9. Comparison of the catalytic activity and selectivity of selected gold(I)-carbene complexes in the reaction of 1a and 2a under the original and optimised conditions ... 39

10. References for Known Compound ... 39

11. NMR Spectra (^{1}H and ^{13}C{1H}) ... 40

Figure S1. ^{1}H NMR (500 MHz) spectrum of **B6** in $CDCl_3$ ... 40

Figure S2. ^{13}C{1H} NMR (125 MHz) spectrum of **B6** in $CDCl_3$ ... 40

Figure S3. ^{1}H NMR (500 MHz) spectrum of **B15** in $CDCl_3$ 41
Figure S4. ^{13}C{1H} NMR (125 MHz) spectrum of **B15** in $CDCl_3$ 41
Figure S5. ^{1}H NMR (500 MHz) spectrum of **B16** in $CDCl_3$ 42
Figure S6. ^{13}C{1H} NMR (125 MHz) spectrum of **B16** in $CDCl_3$ 42
Figure S7. ^{1}H NMR (500 MHz) spectrum of **B17** in $CDCl_3$ 43
Figure S8. ^{13}C{1H} NMR (125 MHz) spectrum of **B17** in $CDCl_3$ 43
Figure S9. ^{1}H NMR (500 MHz) spectrum of **B18** in $CDCl_3$ 44
Figure S10. ^{13}C{1H} NMR (125 MHz) spectrum of **B18** in $CDCl_3$ 44
Figure S11. ^{1}H NMR (500 MHz) spectrum of **B19** in $CDCl_3$ 45
Figure S12. ^{13}C{1H} NMR (125 MHz) spectrum of **B19** in $CDCl_3$ 45
Figure S13. ^{1}H NMR (500 MHz) spectrum of **B20** in $CDCl_3$ 46
Figure S14. ^{13}C{1H} NMR (125 MHz) spectrum of **B20** in $CDCl_3$ 46
Figure S15. ^{1}H NMR (500 MHz) spectrum of **B21** in $CDCl_3$ 47
Figure S16. ^{13}C{1H} NMR (125 MHz) spectrum of **B21** in $CDCl_3$ 47
Figure S17. ^{1}H NMR (500 MHz) spectrum of **B22** in $CDCl_3$ 48
Figure S18. ^{13}C{1H} NMR (125 MHz) spectrum of **B22** in $CDCl_3$ 48
Figure S19. ^{1}H NMR (500 MHz) spectrum of **B23** in $CDCl_3$ 49
Figure S20. ^{13}C{1H} NMR (125 MHz) spectrum of **B23** in $CDCl_3$ 49
Figure S21. ^{1}H NMR (500 MHz) spectrum of **B24** in $CDCl_3$ 50
Figure S22. ^{13}C{1H} NMR (125 MHz) spectrum of **B24** in $CDCl_3$ 50
Figure S23. ^{1}H NMR (400 MHz) spectrum of **B25** in $CDCl_3$ 51
Figure S24. ^{13}C{1H} NMR (100 MHz) spectrum of **B25** in $CDCl_3$ 51
Figure S25. ^{1}H NMR (400 MHz) spectrum of **B26** in $CDCl_3$ 52
Figure S26. ^{13}C{1H} NMR (100 MHz) spectrum of **B26** in $CDCl_3$ 52
Figure S27. ^{1}H NMR (500 MHz) spectrum of **B27** in $CDCl_3$ 53
Figure S28. ^{13}C{1H} NMR (125 MHz) spectrum of **B27** in $CDCl_3$ 53
Figure S29. ^{1}H NMR (500 MHz) spectrum of **B28** in $CDCl_3$ 54

Figure S30. $^{13}C\{1H\}$ NMR (125 MHz) spectrum of **B28** in $CDCl_3$ 54
Figure S31. ^{1}H NMR (500 MHz) spectrum of **B29** in $CDCl_3$ 55
Figure S32. $^{13}C\{1H\}$ NMR (125 MHz) spectrum of **B29** in $CDCl_3$ 55
Figure S33. ^{1}H NMR (500 MHz) spectrum of **B30** in $CDCl_3$ 56
Figure S34. $^{13}C\{1H\}$ NMR (125 MHz) spectrum of **B30** in $CDCl_3$ 56
Figure S35. ^{1}H NMR (500 MHz) spectrum of **B31** in $CDCl_3$ 57
Figure S36. $^{13}C\{1H\}$ NMR (125 MHz) spectrum of **B31** in $CDCl_3$ 57
Figure S37. ^{1}H NMR (500 MHz) spectrum of **B32** in $CDCl_3$ 58
Figure S38. $^{13}C\{1H\}$ NMR (125 MHz) spectrum of **B32** in $CDCl_3$ 58
Figure S39. ^{1}H NMR (500 MHz) spectrum of **B35** in $CDCl_3$ 59
Figure S40. $^{13}C\{1H\}$ NMR (125 MHz) spectrum of **B35** in $CDCl_3$ 59
Figure S41. ^{1}H NMR (500 MHz) spectrum of **B36** in $CDCl_3$ 60
Figure S42. $^{13}C\{1H\}$ NMR (125 MHz) spectrum of **B36** in $CDCl_3$ 60
Figure S43. ^{1}H NMR (500 MHz) spectrum of **B37** in $CDCl_3$ 61
Figure S44. $^{13}C\{1H\}$ NMR (125 MHz) spectrum of **B37** in $CDCl_3$ 61
Figure S45. ^{1}H NMR (500 MHz) spectrum of **B38** in $CDCl_3$ 62
Figure S46. $^{13}C\{1H\}$ NMR (125 MHz) spectrum of **B38** in $CDCl_3$ 62
Figure S47. ^{1}H NMR (500 MHz) spectrum of **B39** in $CDCl_3$ 63
Figure S48. $^{13}C\{1H\}$ NMR (125 MHz) spectrum of **B39** in $CDCl_3$ 63
Figure S49. ^{1}H NMR (500 MHz) spectrum of **B40** in $CDCl_3$ 64
Figure S50. $^{13}C\{1H\}$ NMR (125 MHz) spectrum of **B40** in $CDCl_3$ 64
Figure S51. ^{1}H NMR (400 MHz) spectrum of **C5** in $CDCl_3$ 65
Figure S52. $^{13}C\{1H\}$ NMR (100 MHz) spectrum of **C5** in $CDCl_3$ 65
Figure S53. ^{1}H NMR (500 MHz) spectrum of **C6** in $CDCl_3$ 66
Figure S54. $^{13}C\{1H\}$ NMR (125 MHz) spectrum of **C6** in $CDCl_3$ 66
Figure S55. ^{1}H NMR (500 MHz) spectrum of **C15** in $CDCl_3$ 67
Figure S56. $^{13}C\{1H\}$ NMR (125 MHz) spectrum of **C15** in $CDCl_3$ 67

Figure S57. ^{1}H NMR (500 MHz) spectrum of **C16** in $CDCl_3$.. 68

Figure S58. ^{13}C{1H} NMR (125 MHz) spectrum of **C16** in $CDCl_3$.............................. 68

Figure S59. ^{1}H NMR (500 MHz) spectrum of **C17** in $CDCl_3$.. 69

Figure S60. ^{13}C{1H} NMR (125 MHz) spectrum of **C17** in $CDCl_3$.............................. 69

Figure S61. ^{1}H NMR (500 MHz) spectrum of **C18** in $CDCl_3$.. 70

Figure S62. ^{13}C{1H} NMR (125 MHz) spectrum of **C18** in $CDCl_3$.............................. 70

Figure S63. ^{1}H NMR (500 MHz) spectrum of **C19** in $CDCl_3$.. 71

Figure S64. ^{13}C{1H} NMR (125 MHz) spectrum of **C19** in $CDCl_3$.............................. 71

Figure S65. ^{1}H NMR (500 MHz) spectrum of **C20** in $CDCl_3$.. 72

Figure S66. ^{13}C{1H} NMR (125 MHz) spectrum of **C20** in $CDCl_3$.............................. 72

Figure S67. ^{1}H NMR (500 MHz) spectrum of **C21** in $CDCl_3$.. 73

Figure S68. ^{13}C{1H} NMR (125 MHz) spectrum of **C21** in $CDCl_3$.............................. 73

Figure S69. ^{1}H NMR (500 MHz) spectrum of **C22** in $CDCl_3$.. 74

Figure S70. ^{13}C{1H} NMR (125 MHz) spectrum of **C22** in $CDCl_3$.............................. 74

Figure S71. ^{1}H NMR (500 MHz) spectrum of **C23** in $CDCl_3$.. 75

Figure S72. ^{13}C{1H} NMR (125 MHz) spectrum of **C23** in $CDCl_3$.............................. 75

Figure S73. ^{1}H NMR (500 MHz) spectrum of **C24** in $CDCl_3$.. 76

Figure S74. ^{13}C{1H} NMR (125 MHz) spectrum of **C24** in $CDCl_3$.............................. 76

Figure S75. ^{1}H NMR (400 MHz) spectrum of **C25** in $CDCl_3$.. 77

Figure S76. ^{13}C{1H} NMR (100 MHz) spectrum of **C25** in $CDCl_3$.............................. 77

Figure S77. ^{1}H NMR (400 MHz) spectrum of **C26** in $CDCl_3$.. 78

Figure S78. ^{13}C{1H} NMR (100 MHz) spectrum of **C26** in $CDCl_3$.............................. 78

Figure S79. ^{1}H NMR (500 MHz) spectrum of **C27** in $CDCl_3$.. 79

Figure S80. ^{13}C{1H} NMR (125 MHz) spectrum of **C27** in $CDCl_3$.............................. 79

Figure S81. ^{1}H NMR (500 MHz) spectrum of **C28** in $CDCl_3$.. 80

Figure S82. ^{13}C{1H} NMR (125 MHz) spectrum of **C28** in $CDCl_3$.............................. 80

Figure S83. ^{1}H NMR (500 MHz) spectrum of **C29** in $CDCl_3$.. 81

Figure S84. $^{13}C\{1H\}$ NMR (125 MHz) spectrum of **C29** in $CDCl_3$ 81

Figure S85. ^{1}H NMR (500 MHz) spectrum of **C30** in $CDCl_3$ 82

Figure S86. $^{13}C\{1H\}$ NMR (125 MHz) spectrum of **C30** in $CDCl_3$ 82

Figure S87. ^{1}H NMR (500 MHz) spectrum of **C31** in $CDCl_3$ 83

Figure S88. $^{13}C\{1H\}$ NMR (125 MHz) spectrum of **C31** in $CDCl_3$ 83

Figure S89. ^{1}H NMR (500 MHz) spectrum of **C32** in $CDCl_3$ 84

Figure S90. $^{13}C\{1H\}$ NMR (125 MHz) spectrum of **C32** in $CDCl_3$ 84

Figure S91. ^{1}H NMR (500 MHz) spectrum of **C35** in $CDCl_3$ 85

Figure S92. $^{13}C\{1H\}$ NMR (125 MHz) spectrum of **C35** in $CDCl_3$ 85

Figure S93. ^{1}H NMR (500 MHz) spectrum of **C36** in $CDCl_3$ 86

Figure S94. $^{13}C\{1H\}$ NMR (125 MHz) spectrum of **C36** in $CDCl_3$ 86

Figure S95. ^{1}H NMR (500 MHz) spectrum of **C37** in $CDCl_3$ 87

Figure S96. $^{13}C\{1H\}$ NMR (125 MHz) spectrum of **C37** in $CDCl_3$ 87

Figure S97. ^{1}H NMR (500 MHz) spectrum of **C38** in $CDCl_3$ 88

Figure S98. $^{13}C\{1H\}$ NMR (125 MHz) spectrum of **C38** in $CDCl_3$ 88

Figure S99. ^{1}H NMR (500 MHz) spectrum of **C39** in $CDCl_3$ 89

Figure S100. $^{13}C\{1H\}$ NMR (125 MHz) spectrum of **C39** in $CDCl_3$ 89

Figure S101. ^{1}H NMR (500 MHz) spectrum of **C40** in $CDCl_3$ 90

Figure S102. $^{13}C\{1H\}$ NMR (125 MHz) spectrum of **C40** in $CDCl_3$ 90

Figure S103. ^{1}H NMR (500 MHz) spectrum of **C41** in $CDCl_3$ 91

Figure S104. $^{13}C\{1H\}$ NMR (125 MHz) spectrum of **C41** in $CDCl_3$ 91

Figure S105. ^{1}H NMR (500 MHz) spectrum of **C42** in $CDCl_3$ 92

Figure S106. $^{13}C\{1H\}$ NMR (125 MHz) spectrum of **C42** in $CDCl_3$ 92

Figure S107. ^{1}H NMR (500 MHz) spectrum of **1a** in DMSO-d_6 93

Figure S108. $^{13}C\{1H\}$ NMR (125 MHz) spectrum of **1a** in DMSO-d_6 93

Figure S109. ^{1}H NMR (500 MHz) spectrum of **1b** in DMSO-d_6 94

Figure S110. $^{13}C\{1H\}$ NMR (125 MHz) spectrum of **1b** in DMSO-d_6 94

Figure S111. ^{1}H NMR (500 MHz) spectrum of **1c** in DMSO-d_6 95

Figure S112. ^{13}C{1H} NMR (125 MHz) spectrum of **1c** in DMSO-d_6 95

Figure S113. ^{1}H NMR (500 MHz) spectrum of **1d** in DMSO-d_6.................................... 96

Figure S114. ^{13}C{1H} NMR (125 MHz) spectrum of **1d** in DMSO-d_6.......................... 96

Figure S115. ^{1}H NMR (500 MHz) spectrum of **3a** in DMSO-d_6.................................... 97

Figure S116. ^{13}C{1H} NMR (125 MHz) spectrum of **3a** in DMSO-d_6.......................... 97

Figure S117. ^{1}H NMR (500 MHz) spectrum of **4a** in DMSO-d_6.................................... 98

Figure S118. ^{13}C{1H} NMR (125 MHz) spectrum of **4a** in DMSO-d_6.......................... 98

Figure S119. ^{1}H NMR (500 MHz) spectrum of **3b** in DMSO-d_6.................................... 99

Figure S120. ^{13}C{1H} NMR (125 MHz) spectrum of **3b** in DMSO-d_6.......................... 99

Figure S121. ^{1}H NMR (500 MHz) spectrum of **4b** in DMSO-d_6.................................. 100

Figure S122. ^{13}C{1H} NMR (125 MHz) spectrum of **4b** in DMSO-d_6........................ 100

Figure S123. ^{1}H NMR (500 MHz) spectrum of **3c** in DMSO-d_6 101

Figure S124. ^{13}C{1H} NMR (125 MHz) spectrum of **3c** in DMSO-d_6 101

Figure S125. ^{1}H NMR (500 MHz) spectrum of **4c** in DMSO-d_6 102

Figure S126. ^{13}C{1H} NMR (125 MHz) spectrum of **4c** in DMSO-d_6 102

Figure S127. ^{1}H NMR (500 MHz) spectrum of **3d** in DMSO-d_6.................................. 103

Figure S128. ^{13}C{1H} NMR (125 MHz) spectrum of **3d** in DMSO-d_6........................ 103

Figure S129. ^{1}H NMR (500 MHz) spectrum of **4d** in DMSO-d_6.................................. 104

Figure S130. ^{13}C{1H} NMR (125 MHz) spectrum of **4d** in DMSO-d_6........................ 104

Figure S131. ^{1}H NMR (500 MHz) spectrum of **3e** in DMSO-d_6 105

Figure S132. ^{13}C{1H} NMR (125 MHz) spectrum of **3e** in DMSO-d_6 105

Figure S133. ^{1}H NMR (500 MHz) spectrum of **4e** in DMSO-d_6 106

Figure S134. ^{13}C{1H} NMR (125 MHz) spectrum of **4e** in DMSO-d_6 106

Figure S135. ^{1}H NMR (500 MHz) spectrum of **3f** in DMSO-d_6.................................. 107

Figure S136. ^{13}C{1H} NMR (125 MHz) spectrum of **3f** in DMSO-d_6......................... 107

Figure S137. ^{1}H NMR (500 MHz) spectrum of **3g** in DMSO-d_6 108

Figure S138. $^{13}C\{1H\}$ NMR (125 MHz) spectrum of **3g** in DMSO-*d6* 108

Figure S139. ^{1}H NMR (500 MHz) spectrum of **4g** in DMSO-*d6* 109

Figure S140. $^{13}C\{1H\}$ NMR (125 MHz) spectrum of **4g** in DMSO-*d6* 109

Figure S141. ^{1}H NMR (500 MHz) spectrum of **3h** in DMSO-*d6* 110

Figure S142. $^{13}C\{1H\}$ NMR (125 MHz) spectrum of **3h** in DMSO-*d6* 110

Figure S143. ^{1}H NMR (500 MHz) spectrum of **4h** in DMSO-*d6* 111

Figure S144. $^{13}C\{1H\}$ NMR (125 MHz) spectrum of **4h** in DMSO-*d6* 111

Figure S145. ^{1}H NMR (500 MHz) spectrum of **3i** in DMSO-*d6* 112

Figure S146. $^{13}C\{1H\}$ NMR (125 MHz) spectrum of **3i** in DMSO-*d6* 112

Figure S147. ^{1}H NMR (500 MHz) spectrum of **4i** in DMSO-*d6* 113

Figure S148. $^{13}C\{1H\}$ NMR (125 MHz) spectrum of **4i** in DMSO-*d6* 113

Figure S149. ^{1}H NMR (500 MHz) spectrum of **3j** in DMSO-*d6* 114

Figure S150. $^{13}C\{1H\}$ NMR (125 MHz) spectrum of **3j** in DMSO-*d6* 114

Figure S151. ^{1}H NMR (500 MHz) spectrum of **4j** in DMSO-*d6* 115

Figure S152. $^{13}C\{1H\}$ NMR (125 MHz) spectrum of **4j** in DMSO-*d6* 115

Figure S153. ^{1}H NMR (500 MHz) spectrum of **3k** in DMSO-*d6* 116

Figure S154. $^{13}C\{1H\}$ NMR (125 MHz) spectrum of **3k** in DMSO-*d6* 116

Figure S155. ^{1}H NMR (500 MHz) spectrum of **3l** in DMSO-*d6* 117

Figure S156. $^{13}C\{1H\}$ NMR (125 MHz) spectrum of **3l** in DMSO-*d6* 117

Figure S157. ^{1}H NMR (500 MHz) spectrum of **4l** in DMSO-*d6* 118

Figure S158. $^{13}C\{1H\}$ NMR (125 MHz) spectrum of **4l** in DMSO-*d6* 118

Figure S159. ^{1}H NMR (500 MHz) spectrum of **3m** in DMSO-*d6* 119

Figure S160. $^{13}C\{1H\}$ NMR (125 MHz) spectrum of **3m** in DMSO-*d6* 119

Figure S161. ^{1}H NMR (500 MHz) spectrum of **3n** in DMSO-*d6* 120

Figure S162. $^{13}C\{1H\}$ NMR (125 MHz) spectrum of **3n** in DMSO-*d6* 120

Figure S163. ^{1}H NMR (500 MHz) spectrum of **3o** in DMSO-*d6* 121

Figure S164. $^{13}C\{1H\}$ NMR (125 MHz) spectrum of **3o** in DMSO-*d6* 121

Figure S165. ^{1}H NMR (500 MHz) spectrum of **4o** in DMSO-*d6* 122

Figure S166. ^{13}C{1H} NMR (125 MHz) spectrum of **4o** in DMSO-*d6* 122

Figure S167. ^{1}H NMR (500 MHz) spectrum of **3p** in DMSO-*d6* 123

Figure S168. ^{13}C{1H} NMR (125 MHz) spectrum of **3p** in DMSO-*d6* 123

Figure S169. ^{1}H NMR (500 MHz) spectrum of **3q-4q** in DMSO-*d6* 124

Figure S170. ^{13}C{1H} NMR (125 MHz) spectrum of **3q-4q** in DMSO-*d6* 124

Figure S171. ^{1}H NMR (500 MHz) spectrum of **3r** in DMSO-*d6* 125

Figure S172. ^{13}C{1H} NMR (125 MHz) spectrum of **3r** in DMSO-*d6* 125

Figure S173. ^{1}H NMR (500 MHz) spectrum of **4r** in DMSO-*d6* 126

Figure S174. ^{13}C{1H} NMR (125 MHz) spectrum of **4r** in DMSO-*d6* 126

Figure S175. ^{1}H NMR (500 MHz) spectrum of **3s-4s** in DMSO-*d6* 127

Figure S176. ^{13}C{1H} NMR (125 MHz) spectrum of **3s-4s** in DMSO-*d6* 127

Figure S177. ^{1}H NMR (500 MHz) spectrum of **3t-4t** in DMSO-*d6* 128

Figure S178. ^{13}C{1H} NMR (125 MHz) spectrum of **3t-4t** in DMSO-*d6* 128

1. General information

All reagents obtained from commercial sources were used without further purification. Anhydrous solvents were obtained from commercial sources and used without further drying. The reactions were monitored using LCMS and GCMS instruments. Analytical LC-MS: Agilent HP1200 LC with Agilent 6140 quadrupole MS, operating in positive or negative ion electrospray ionisation mode. Molecular weight scan range was 100 to 1350 m/z. Parallel UV detection was done at 210 nm and 254 nm. Samples were supplied as a 1 mM solution in MeCN or in THF/water (1:1) with 5 µL loop injection. LCMS analyses were performed on two instruments, one of which was operated with basic, and the other with acidic eluents. Basic LCMS: Gemini-NX, 3 µm, C18, 50 mm × 3.00 mm i.d. column at 23°C, at a flow rate of 1 mL min^{-1} using 5 mM aq. NH_4HCO_3 solution and MeCN as eluents. Acidic LCMS: ZORBAX Eclipse XDB-C18, 1.8 µm, 50 mm × 4.6 mm i.d. column at 40°C, at a flow rate of 1 mL min^{-1} using water and MeCN as eluents, both containing 0.02 V/V% formic acid. Combination gas chromatography and low-resolution mass spectrometry were performed on Agilent 6850 gas chromatograph and Agilent 5975C mass spectrometer using 15 m × 0.25 mm column with 0.25 µm HP-5MS coating and helium as carrier gas. Ion source: EI^+, 70 eV, 230°C, quadrupole: 150°C, interface: 300°C. Flash chromatography was performed on ISCO CombiFlash Rf 200i with pre-packed silica-gel cartridges (Redi*Sep*®*R*f Gold High Performance). Analytical Chiral method development were performed: Lux® 5 µm i-Amylose-1 (250 x 4.6 mm), Lux® 5 µm i-Amylose-3 (250 x 4.6 mm), Lux® 5 µm i-Cellulose-5 (250 x 4.6 mm). LC-MS: Agilent HP1200 LC. Chiral separations were performed on a KNAUER Smartline Preparative HPLC system with a (R, R) WHELKO O-1 50 mm × 500 mm, 10 µm column running at a flow rate of 50 mL min^{-1} with UV diode array detection (210 – 285 nm). Chiral purity was determined on an Agilent 1100 HPLC system with a WHELKO O-1, 250 mm × 4.6 mm, 10 µm column running at a flow rate of 1 mL min^{-1} with UV diode array detection (210 – 285 nm). ^1H NMR and proton-decoupled $^{13}C\{1H\}$ NMR measurements were performed on Bruker Avance III 500 MHz spectrometer and Bruker Avance III 400 MHz spectrometer, using DMSO-d_6 or $CDCl_3$ as solvent. ^1H and $^{13}C\{1H\}$ NMR data are in the form of delta values, given in part per million (ppm), using the residual peak of the solvent as internal standard (DMSO-d_6: 2.50 ppm (^1H) / 39.5 ppm (^{13}C); $CDCl_3$: 7.26 ppm (^1H) / 77.0 ppm (^{13}C)). Splitting patterns are designated as: s (singlet), d (doublet), t (triplet), q (quartet), sp (septet), m (multiplet), br s (broad singlet), dd (doublet of doublets), td (triplet of doublets), qd (quartet of doublets). In some cases two sets of signals appear in the spectra due to hindered rotation. HRMS were determined on a Shimadzu IT-TOF, ion source temperature 200°C, ESI +/-, ionization voltage: (+-)4.5 kV. Mass resolution min. 10000. Melting points were determined by OptiMelt melting view apparatus at ramp rates of 2 °C·min–1 in sealed glass capillaries and are uncorrected. All products had an LC purity above 95% that was corroborated by their ^1H NMR spectrum unless specifically mentioned otherwise.

2. Guide to the Carbene Ligands A1-A42 present in Gold(I) Complexes C1-C42

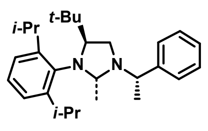

[H]: **A1** [AgCl]: **B1** [AuCl]: **C1**

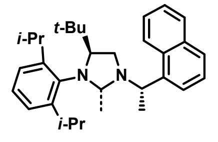

[H]: **A3** [AgCl]: **B3** [AuCl]: **C3**

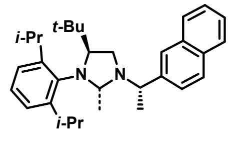

[H]: **A5** [AuCl]: **C5**

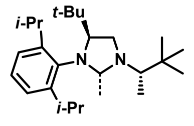

[H]: **A7** [AgCl]: **B7** [AuCl]: **C7**

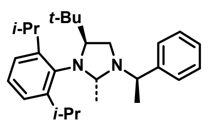

[H]: **A2** [AgCl]: **B2** [AuCl]: **C2**

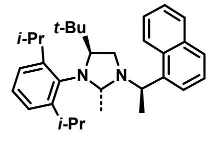

[H]: **A4** [AgCl]: **B4** [AuCl]: **C4**

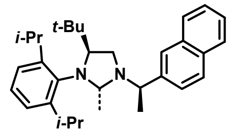

[H]: **A6** [AgCl]: **B6** [AuCl]: **C6**

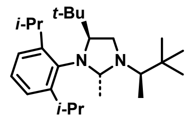

[H]: **A8** [AgCl]: **B8** [AuCl]: **C8**

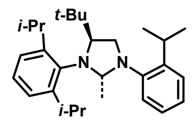

[H]: **A9** [AgCl]: **B9** [AuCl]: **C9**

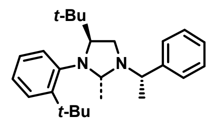

[H]: **A11** [AgCl]: **B11** [AuCl]: **C11**

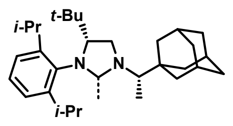

[H]: **A13** [AgCl]: **B13** [AuCl]: **C13**

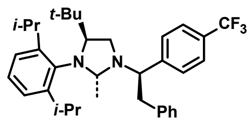

[H]: **A15** [AgCl]: **B15** [AuCl]: **C15**

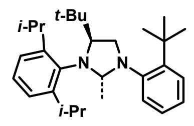

[H]: **A10** [AgCl]: **B10** [AuCl]: **C10**

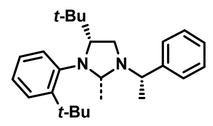

[H]: **A12** [AgCl]: **B12** [AuCl]: **C11**

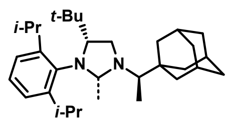

[H]: **A14** [AgCl]: **B14** [AuCl]: **C14**

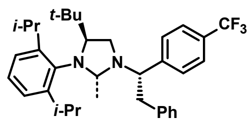

[H]: **A16** [AgCl]: **B16** [AuCl]: **C16**

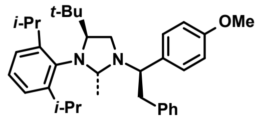

[H]: **A17** [AgCl]: **B17** [AuCl]: **C17**

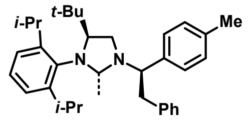

[H]: **A19** [AgCl]: **B19** [AuCl]: **C19**

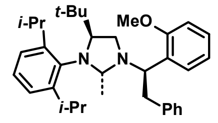

[H]: **A21** [AgCl]: **B21** [AuCl]: **C21**

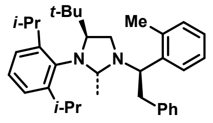

[H]: **A23** [AgCl]: **B23** [AuCl]: **C23**

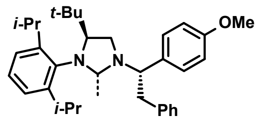

[H]: **A18** [AgCl]: **B18** [AuCl]: **C18**

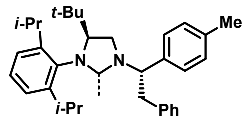

[H]: **A20** [AgCl]: **B20** [AuCl]: **C20**

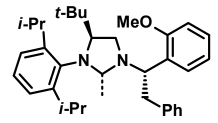

[H]: **A22** [AgCl]: **B22** [AuCl]: **C22**

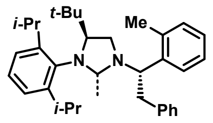

[H]: **A24** [AgCl]: **B24** [AuCl]: **C24**

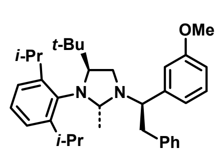

[H]: **A25** [AgCl]: **B25** [AuCl]: **C25**

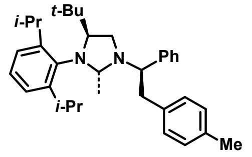

[H]: **A27** [AgCl]: **B27** [AuCl]: **C27**

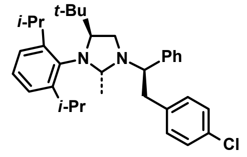

[H]: **A29** [AgCl]: **B29** [AuCl]: **C29**

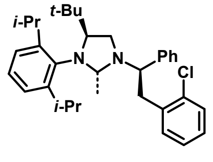

[H]: **A30** [AgCl]: **B30** [AuCl]: **C30**

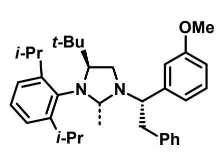

[H]: **A26** [AgCl]: **B26** [AuCl]: **C26**

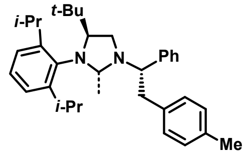

[H]: **A28** [AgCl]: **B28** [AuCl]: **C28**

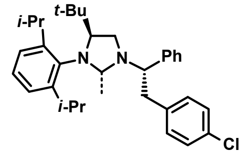

[H]: **A30** [AgCl]: **B30** [AuCl]: **C30**

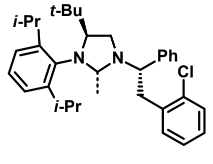

[H]: **A32** [AgCl]: **B32** [AuCl]: **C32**

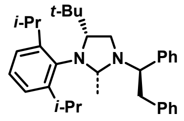

[H]: **A33** [AgCl]: **B33** [AuCl]: **C33**

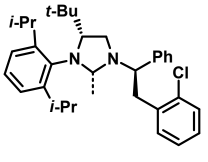

[H]: **A35** [AgCl]: **B35** [AuCl]: **C35**

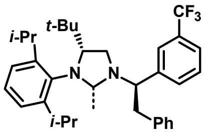

[H]: **A37** [AgCl]: **B37** [AuCl]: **C37**

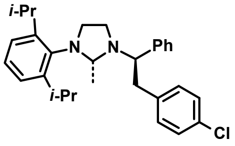

[H]: **A39** [AgCl]: **B39** [AuCl]: **C39**

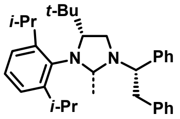

[H]: **A34** [AgCl]: **B34** [AuCl]: **C34**

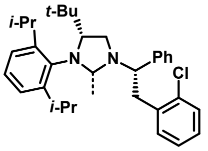

[H]: **A36** [AgCl]: **B36** [AuCl]: **C36**

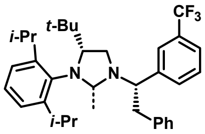

[H]: **A38** [AgCl]: **B38** [AuCl]: **C38**

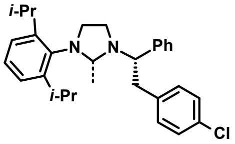

[H]: **A40** [AgCl]: **B40** [AuCl]: **C40**

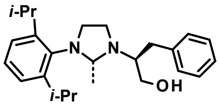

[H]: **A41** [AuCl]: **C41**

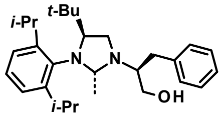

[H]: **A42** [AuCl]: **C42**

3. Experimental Section

4. General Procedure for the Preparation of *N,N*-Disubstituted 4,5-Dihydro-1*H*-imidazol-3-ium Chloride Derivatives

The preparation of **A1-A12**[1] and **A13-A40**[2] has already been reported. In all cases the following General procedure was used.

To a 1 mmol of the appropriate diamine 5 mL $HC(OEt)_3$ and 1.2 eq. NH_4Cl were added was added in one portion. The mixture was heated to reflux temperature and was stirred for 2-8 hours, when complete conversion was observed. The volatiles were removed under reduced pressure and the residue was purified via column chromatography using DCM and 1.2% methanolic ammonia as eluents. The crude product was recrystallized from DCM-Et_2O.

(*2S*)-*2*-[*3*-(*2,6*-diisopropylphenyl)-*4,5*-dihydroimidazol-*1*-ium-*1*-yl]-*3*-phenyl-propan-*1*-ol-chloride (A41)

Step 1

***2*-[[(*1S*)-*1*-benzyl-*2*-hydroxy-ethyl]amino]-*N*-(*2,6*-diisopropylphenyl)acetamide**

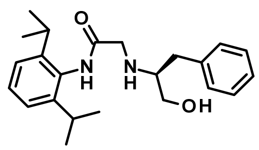

1.0 g *2*-chloro-*N*-(*2,6*-diisopropylphenyl)acetamide[3] (3.94 mmol, 1 equiv) and 715 mg (*2R*)-*2*-amino-*3*-phenyl-propan-*1*-ol (4.73 mmol, 1.2 equiv) were dissolved in 20 mL dry acetonitrile at rt and 1.09 g K_2CO_3 (7.88 mmol, 2 equiv) was added. The reaction mixture was then warmed up to 80°C and stirred for 24 h, while full conversion was observed. *Celite* was added to the reaction mixture and the volatiles were removed under reduced pressure. Then it was purified via flash chromatography silica gel column using heptane and EtOAc as eluents. 580 mg desired product was obtained (1.57 mmol, 40% Yield) as light-yellow oil.

^{1}H NMR (500 MHz, DMSO-d_6) δ 9.13 (s, 1 H), 7.29-7.11 (m, 5 H), 7.23 (m, 1 H), 7.13 (d, J = 7.8 Hz, 2 H), 4.69 (t, J = 5.2 Hz, 1 H), 3.39/3.35 (m+m, 2 H), 3.37/3.28 (m+m, 2 H), 2.96 (sp, J = 6.8 Hz, 2 H), 2.74 (m, 1 H), 2.71/2.65 (m+m, 2 H), 2.45 (brs, 1 H), 1.09/1.08 (d, J = 6.8/2.4 Hz, 12 H). ^{13}C{1H} NMR (125 MHz, DMSO-d_6) δ 171.9, 140.1, 127.8, 123.2, 62.9, 61.9, 50.2, 38.1, 28.4, 23.9. HRMS calcd for ($C_{23}H_{33}N_2O_2$) $[M + H]^+$ 369.2551, found 369.2537.

Step 2

(*2S*)-*2*-[*2*-(*2,6*-diisopropylanilino)ethylamino]-*3*-phenyl-propan-*1*-ol

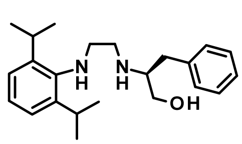

222 mg $LiAlH_4$ (6.00 mmol, 4 equiv) was suspended in 20 mL dry THF at 0°C and 790 mg $AlCl_3$ (6.00 mmol, 4 equiv) was also added, then 550 mg *2*-[[(*1S*)-*1*-benzyl-*2*-hydroxy-ethyl]amino]-*N*-(*2,6*-diisopropylphenyl) acetamide (1.50 mmol, 1 equiv) from Step 1 in THF was added dropwise to the previous mixture at 0°C. The reaction mixture was stirred at 60°C for 1h. After completion, at 0°C 0.22 ml water, 0.44 ml 2N NaOH and 0.44 ml water was added to the reaction mixture. Then added 5 mL MTBE and stirred for 5 min. The mixture was NOT filtered, *Celite* was added and the volatiles were removed under reduced pressure. This mixture was purified via preparative reversed phase chromatography using NH_4HCO_3 and acetonitrile as eluents. 425 mg desired diamine (1.20 mmol, 80% Yield) were obtained as light-yellow oil.

^{1}H NMR (500 MHz, DMSO-d_6) δ 7.27 (t, J = 7.2 Hz, Ar-H, 2 H), 7.22 (d, J = 7.2 Hz, Ar-H, 2 H), 7.17 (t, J = 7.2 Hz, Ar-H, 1 H), 7.01 (d, J = 7.6 Hz, Ar-H, 2 H), 6.94 (t, J = 7.6 Hz, Ar-H, 1 H), 4.54 (t, J = 5.2 Hz, OH, 1 H), 3.71 (t, J = 6.0 Hz, NH, 1 H), 3.33/3.28 (m+m, CH_2, 2 H), 3.29 (sp, J = 6.7 Hz, CH,

2 H), 2.78 (m, CH_2, 2 H), 2.78/2.73 (m+m, CH_2, 2 H), 2.73 (m, CH, 1 H), 2.66 (d, J = 6.4 Hz, CH_2, 2 H), 1.71 (m, NH, 1 H), 1.13 (d, J = 6.7 Hz, CH_3, 12 H). $^{13}C\{1H\}$ NMR (125 MHz, DMSO-d_6) δ 143.7, 142.1, 139.9, 129.3, 128.1, 125.7, 123.1, 123.0, 62.2, 60.7, 51.7, 46.8, 37.8, 26.8, 24.3. HRMS calcd for ($C_{23}H_{35}N_2O$) $[M + H]^+$ 355.2744, found 355.2750.

Step 3

***(2S)-2-*[*3-*(*2,6*-diisopropylphenyl)*-4,5*-dihydroimidazol-*1*-ium-*1*-yl]-*3*-phenyl-propan-*1*-ol-chloride (A41)**

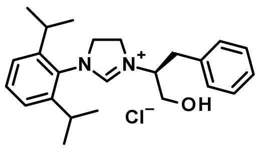

Using General procedure I, 355 mg starting material from Step 2 (1.00 mmol). Yield: 182 mg (0.48 mmol, 48%), off-white crystal. Op: 224-226°C. ^1H NMR (500 MHz, DMSO-d_6) δ 8.83 (s, CH, 1 H), 7.45 (t, J = 7.8 Hz, Ar-H, 1 H), 7.40-7.26 (m, Ar-H, 5 H), 7.31 (dd, J = 7.8, 1.4 Hz, Ar-H, 1 H), 7.28 (dd, J = 7.8, 1.4 Hz, Ar-H, 1 H), 5.51 (t, J = 5.7 Hz, OH, 1 H), 4.21/4.16 (m+m, CH_2, 2 H), 4.13 (m, CH, 1 H), 4.13/3.99 (m+m, CH_2, 2 H), 3.74-3.62 (m, CH_2, 2 H), 2.99/2.91 (dd+m, J = 14.5, 4.9 Hz, CH_2, 2 H), 2.91 (sp, J = 6.7 Hz, CH, 1 H), 2.36 (sp, J = 6.7 Hz, CH, 1 H), 1.21 (d, J = 6.7 Hz, CH_3, 3 H), 1.13 (d, J = 6.7 Hz, CH_3, 3 H), 1.11 (d, J = 6.7 Hz, CH_3, 3 H), 0.93 (d, J = 6.7 Hz, CH_3, 3 H). $^{13}C\{1H\}$ NMR (125 MHz, DMSO-d_6) δ 159.1, 146.5, 146.3, 137.0, 130.6, 130.3, 128.9, 128.6, 126.8, 124.6, 62.2, 59.5, 52.7, 45.2, 33.3, 27.8, 24.7, 24.5, 23.7, 23.5. HRMS calcd for ($C_{24}H_{33}N_2O$) $[M]^+$ 365.2593, found 365.2590.

5. General Procedure for Preparation of Silver(I)-carbene Complexes (B6, B15-32, B35-40)

The solution of the corresponding dihydroimidazolium salt in DCM (10 mL/mmol) and 0.55 equivalents of silver oxide were mixed at room temperature, protected from light, until complete conversion was achieved. The reaction was followed by NMR. The reaction time was usully between 1-8 hours depending on the size of the side chains.

After complete conversion, the solution was filtered through a pad of celite and the filtrate was evaporated. The resulting solid was recrystallized from DCM/DEE solution.

The **B1-4**, **B7-14**, and **B33-34** silver(I)-carbene complexes were prepared by following published procedures.[1]

(*4S*)*-4-terc*-Butyl*-3-*(*2',6'*-diisopropylphenyl)*-1-*[(*1R*)*-2'*-naphtylethyl]imidazolidin-2-ylidene-silver-chloride (B6)

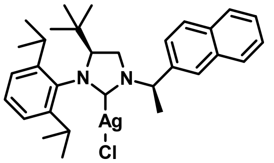

Starting material: 1.20 g ***(4S)-4-terc*-Butyl*-3-*(*2',6'*-diisopropyl-phenyl)*-1-*[(*1R*)*-2'*-naphtylethyl]*-4,5*-dihydroimidazolium Chloride**[2] (2.52 mmol). Yield: 1.07 g (1.83 mmol, 72.6%), brown solid. Mp: 208-210°C. ^1H NMR (500 MHz, $CDCl_3$) δ 7.96-7.54 (m, Ar-H, 4 H), 7.91 (d, J = 8.4 Hz, Ar-H, 1 H), 7.85 (s, Ar-H, 1 H), 7.54 (dd, J = 8.4, 1.5 Hz, Ar-H, 1 H), 7.36 (t, J = 7.7 Hz, Ar-H, 1 H), 7.23 (dd, J = 7.7, 1.2 Hz, Ar-H, 1 H), 7.17 (dd, J = 7.7, 1.2 Hz, Ar-H, 1 H), 5.78 (q, J = 7.0 Hz, CH, 1 H), 3.90 (dd, J = 11.8, 10.1 Hz, CH, 1 H), 3.53/3.31 (t+t, J = 10.1 Hz, J = 11.8 Hz, CH_2, 2 H), 3.51 (sp, J = 6.8 Hz, CH, CH, 1 H), 2.69 (sp, J = 6.8 Hz, CH, CH, 1 H), 1.88 (d, J = 7.0 Hz, CH_3, 3 H), 1.54 (d, J = 6.8 Hz, CH, CH_3, 3 H), 1.34 (d, J = 6.8 Hz, CH, CH_3, 3 H), 1.30 (d, J = 6.8 Hz, CH, CH_3, 3 H), 1.26 (d, J = 6.8 Hz, CH, CH_3, 3 H), 0.78 (s, CH_3, 9 H). $^{13}C\{1H\}$ NMR (125 MHz, $CDCl_3$) δ 208.0, 146.6, 146.0, 137.2, 135.6, 133.3, 133.2, 129.5, 129.0, 128.2, 127.9, 126.9, 126.8, 125.8, 125.6, 125.3, 125.2, 76.2, 59.4, 46.0, 35.0, 29.1, 28.2, 27.8, 26.5, 26.3, 23.7, 23.5, 16.9. HRMS calcd for ($C_{31}H_{40}AgClN_2$) $[M + H]^+$ 582.1931, found 582.1925.

(4S)-4-terc-Butyl-3-(2',6'-diisopropylphenyl)-1-[(1R)-1-(4-trifluoromethyl)-2-phenylethyl]imidazolidin-2-ylidene-silver-chloride (B15)

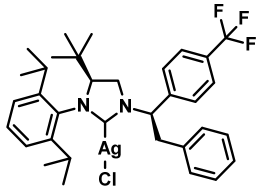

Starting material: 1.29 g **(4S)-4-terc-Butyl-3-(2',6'-diisopropylphenyl)-1-[(1R)-1-(4-trifluoromethyl)-2-phenylethyl] -4,5-dihydroimidazolium Chloride**[2] (2.26 mmol). Yield: 1.22 g (1.80 mmol, 79.5%), pink solid. Mp: 232-234°C. ^{1}H NMR (500 MHz, $CDCl_3$) δ 7.77 (d, J = 8.1 Hz, Ar-H, 2 H), 7.64 (d, J = 8.1 Hz, Ar-H, 2 H), 7.49-7.32 (m, Ar-H, 5 H), 7.29 (t, J = 7.8 Hz, Ar-H, 1 H), 7.11 (dd, J = 7.8, 1.5 Hz, Ar-H, 1 H), 7.09 (dd, J = 7.8, 1.5 Hz, Ar-H, 1 H), 5.85 (dd, J = 12.1, 4.2 Hz, CH, 1 H), 3.95 (t, J = 11.5 Hz, CH, 1 H), 3.54/3.36 (dd+dd, J = 14.0, 4.2 Hz, J = 14.0, 12.1 Hz, CH_2, 2 H), 3.49/3.28 (t+t, J = 11.5 Hz, J = 11.5 Hz, CH_2, 2 H), 2.71 (sp, J = 6.7 Hz, CH, 1 H), 2.58 (sp, J = 6.7 Hz, CH, 1 H), 1.27 (d, J = 6.7 Hz, CH_3, 3 H), 1.20 (d, J = 6.7 Hz, CH_3, 3 H), 1.18 (d, J = 6.7 Hz, CH_3, 3 H), 1.18 (d, J = 6.7 Hz, CH_3, 3 H), 0.67 (s, CH_3, 9 H). ^{13}C{1H} NMR (125 MHz, $CDCl_3$) δ 209.7, 146.3, 146.0, 136.3, 136.0, 129.5, 129.4, 128.9, 127.7, 127.5, 126.3, 125.4, 125.3, 75.5, 64.4, 46.9, 36.6, 28.8, 28.2, 27.6, 26.6, 26.5, 23.8, 23.5. HRMS calcd for ($C_{34}H_{41}AgClF_3N_2$) $[M]^+$ 676.1961, found 676.1935.

(4S)-4-terc-Butyl-3-(2',6'-diisopropylphenyl)-1-[(1S)-1-(4-trifluoromethyl)-2-phenylethyl]imidazolidin-2-ylidene-silver-chloride (B16)

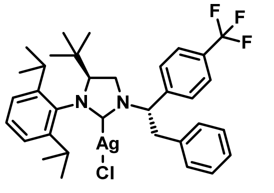

Starting material: 563 mg **(4S)-4-terc-Butyl-3-(2',6'-diisopropylphenyl)-1-[(1S)-1-(4-trifluoromethyl)-2-phenylethyl] -4,5-dihydroimidazolium Chloride**[2] (0.99 mmol). Yield: 355 mg (0.53 mmol, 53.1%), white solid. Mp: 199-201°C. ^{1}H NMR (500 MHz, $CDCl_3$) δ 7.77 (m, J = 8.2 Hz, Ar-H, 2 H), 7.64 (m, J = 8.2 Hz, Ar-H, 2 H), 7.47-7.27 (m, Ar-H, 5 H), 7.29 (t, J = 7.7 Hz, Ar-H, 1 H), 7.15 (dd, J = 7.7, 1.4 Hz, Ar-H, 1 H), 7.06 (dd, J = 7.7, 1.4 Hz, Ar-H, 1 H), 5.82 (dd, J = 11.9, 4.8 Hz, CH, 1 H), 3.87 (dd, J = 11.5, 10.4 Hz, CH, 1 H), 3.76/3.17 (t+t, J = 11.5 Hz, J = 10.4 Hz, CH, 2 H), 3.56/3.43 (dd+dd, J = 14.3, 4.8 Hz, J = 14.3, 11.9 Hz, CH_2, 2 H), 3.28 (sp, J = 6.7 Hz, CH, 1 H), 2.13 (sp, J = 6.7 Hz, CH, 1 H), 1.46 (d, J = 6.7 Hz, CH_3, 3 H), 1.27 (d, J = 6.7 Hz, CH_3, 3 H), 1.20 (d, J = 6.7 Hz, CH_3, 3 H), 0.88 (d, J = 6.7 Hz, CH_3, 3 H), 0.60 (s, CH_3, 9 H). ^{13}C{1H} NMR (125 MHz, $CDCl_3$) δ 209.7, 146.5, 145.8, 141.7, 136.2, 135.8, 129.5, 129.4, 129.0, 127.7, 127.6, 126.3, 125.5, 125.1, 76.0, 66.0, 64.5, 46.2, 35.9, 34.4, 29.1, 27.8, 27.6, 26.9, 26.1, 23.5, 23.4. HRMS calcd for ($C_{34}H_{41}AgClF_3N_2$) $[M]^+$ 676.1961, found 676.1971.

(4S)-4-terc-Butyl-3-(2',6'-diisopropylphenyl)-1-[(1R)-1-(4-methoxyphenyl)-2-phenylethyl]imidazolidin-2-ylidene-silver-chloride (B17)

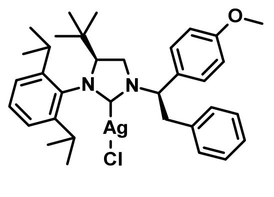

Starting material: 1.20 g **(4S)-4-terc-Butyl-3-(2',6'-diisopropylphenyl)-1-[(1R)-1-(4-methoxyphenyl)-2-phenylethyl] -4,5-dihydroimidazolium Chloride**[2] (2.25 mmol). Yield: 1.14 g (1.77 mmol, 78.8%), white solid. Mp: 221-223°C. ^{1}H NMR (500 MHz, $CDCl_3$) δ 7.48-7.28 (m, Ar-H, 5 H), 7.42 (m, Ar-H, 2 H), 7.27 (t, J = 7.7 Hz, Ar-H, 1 H), 7.10 (dd, J = 7.7, 1.4 Hz, Ar-H, 1 H), 7.07 (dd, J = 7.7, 1.4 Hz, Ar-H, 1 H), 7.01 (m, Ar-H, 2 H), 5.73 (dd, J = 12.0, 4.1 Hz, CH, 1 H), 3.90 (m, CH, 1 H), 3.89 (s, CH_3, 3 H), 3.47/3.31 (m+m, CH_2, 2 H), 3.45/3.31 (m+m, CH_2, 2 H), 2.75 (sp, J = 6.7 Hz, CH, 1 H), 2.59 (sp, J = 6.7 Hz, CH, 1 H), 1.25 (d, J = 6.7 Hz, CH_3, 3 H), 1.18 (d, J = 6.7 Hz, CH_3, 3 H), 1.17 (d, J = 6.7 Hz, CH_3, 3 H), 1.17 (d, J = 6.7 Hz, CH_3, 3 H), 0.66 (s, CH_3, 9 H). ^{13}C{1H} NMR (125 MHz, $CDCl_3$) δ 208.7, 159.8, 146.7, 145.9, 136.6, 129.6, 129.4, 129.2, 128.5, 127.4, 125.4, 125.0, 114.5, 75.9, 66.0, 64.7, 55.5, 46.1, 36.3, 34.4, 29.0, 27.8, 27.5, 26.9, 26.2, 23.5, 23.5. HRMS calcd for ($C_{34}H_{44}AgClN_2O$) $[M]^+$ 638.2193, found 638.2224.

(*4S*)-*4-terc*-Butyl-*3*-(*2',6'*-diisopropylphenyl)-*1*-[(*1S*)-*1*-(*4*-methoxyphenyl)-*2*-phenylethyl]imidazolidin-2-ylidene-silver-chloride (B18)

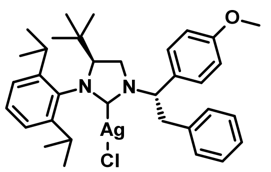

Starting material: 57 mg **(*4S*)-*4-terc*-Butyl-*3*-(*2',6'*-diisopropylphenyl)-*1*-[(*1S*)-*1*-(*4*-methoxyphenyl)-*2*-phenylethyl] -*4,5*-dihydroimidazolium Chloride**[2] (0.11 mmol). Yield: 39 mg (0.06 mmol, 57.0%), white solid. Mp: °C. 221-223 ^{1}H NMR (500 MHz, $CDCl_3$) δ 7.45-7.24 (m, Ar-H, 5 H), 7.42 (m, Ar-H, 2 H), 7.27 (t, J = 7.7 Hz, Ar-H, 1 H), 7.14 (dd, J = 7.7, 1.1 Hz, Ar-H, 1 H), 7.04 (dd, J = 7.7, 1.1 Hz, Ar-H, 1 H), 7.00 (m, Ar-H, 2 H), 5.72 (dd, J = 11.8, 4.6 Hz, CH, 1 H), 3.88 (s, CH_3, 3 H), 3.84 (dd, J = 11.3, 10.6 Hz, CH, 1 H), 3.71/3.18 (t+t, J = 11.3, J = 10.6 Hz, CH_2, 2 H), 3.49/3.37 (dd+dd, J = 14.1, 4.6 J = 14.1, 11.8 Hz, CH_2, 2 H), 3.31 (sp, J = 6.7 Hz, CH, 1 H), 2.16 (sp, J = 6.7 Hz, CH, 1 H), 1.45 (d, J = 6.7 Hz, CH_3, 3 H), 1.26 (d, J = 6.7 Hz, CH_3, 3 H), 1.20 (d, J = 6.7 Hz, CH_3, 3 H), 0.86 (d, J = 6.7 Hz, CH_3, 3 H), 0.58 (s, CH_3, 9 H). ^{13}C{1H} NMR (125 MHz, $CDCl_3$) δ 208.6, 159.7, 146.4, 146.2, 136.8, 136.6, 129.5, 129.3, 129.2, 129.0, 128.3, 127.4, 125.3, 125.2, 114.5, 75.2, 66.0, 64.4, 55.5, 46.6, 28.7, 28.1, 27.7, 26.6, 26.4, 23.8, 23.5. HRMS calcd for ($C_{34}H_{44}AgClN_2O$) $[M]^+$ 638.2193, found 638.2225.

(*4S*)-*4-terc*-Butyl-*3*-(*2',6'*-diisopropylphenyl)-*1*-[(*1R*)-*1*-(*p*-tolyl)-*2*-phenylethyl]imidazolidin-2-ylidene-silver-chloride (B19)

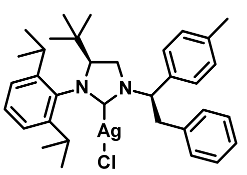

Starting material: 328 mg **(*4S*)-*4-terc*-Butyl-*3*-(*2',6'*-diisopropyl -phenyl)-*1*-[(*1R*)-*1*-(*p*-tolyl)-*2*-phenylethyl]-*4,5*-dihydroimidazolium Chloride**[2] (0.63 mmol). Yield: 319 mg (0.51 mmol, 80.6%), yellow solid. Mp: 229-231°C. ^{1}H NMR (500 MHz, $CDCl_3$) δ 7.43-7.30 (m, Ar-H, 5 H), 7.39 (t, J = 7.7 Hz, Ar-H, 1 H), 7.38 (d, J = 7.8 Hz, Ar-H, 2 H), 7.28 (d, J = 7.8 Hz, Ar-H, 2 H), 7.08 (dd, J = 7.7, 1.4 Hz, Ar-H, 1 H), 7.06 (dd, J = 7.7, 1.4 Hz, Ar-H, 1 H), 5.73 (dd, J = 12.1, 4.1 Hz, CH, 1 H), 3.89 (t, J = 11.7 Hz, CH, 1 H), 3.48/3.32 (m+m, CH_2, 2 H), 3.43/3.31 (t+t, J = 11.7 Hz, J = 11.7 Hz, CH_2, 2 H), 2.73 (sp, J = 6.7 Hz, CH, 1 H), 2.58 (sp, J = 6.7 Hz, CH, 1 H), 2.42 (s, CH_3, 3 H), 1.23 (d, J = 6.7 Hz, CH_3, 3 H), 1.20 (d, J = 6.7 Hz, CH_3, 3 H), 1.17 (d, J = 6.7 Hz, CH_3, 3 H), 1.17 (d, J = 6.7 Hz, CH_3, 3 H), 0.64 (s, CH_3, 9 H). ^{13}C{1H} NMR (125 MHz, $CDCl_3$) δ 209.4, 146.7, 145.9, 138.7, 136.6, 134.6, 129.9, 129.6, 129.4, 129.2, 127.4, 127.2, 125.4, 125.0, 75.9, 66.0, 64.9, 46.2, 36.1, 34.4, 29.0, 27.8, 27.5, 26.9, 26.2, 23.5, 23.5, 21.3. HRMS calcd for ($C_{34}H_{44}AgClN_2$) $[M]^+$ 622.2244, found 622.2178.

(*4S*)-*4-terc*-Butyl-*3*-(*2',6'*-diisopropylphenyl)-*1*-[(*1S*)-*1*-(*p*-tolyl)-*2*-phenylethyl]imidazolidin-2-ylidene-silver-chloride (B20)

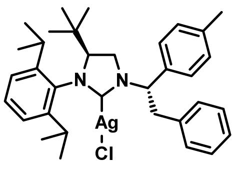

Starting material: 247 mg **(*4S*)-*4-terc*-Butyl-*3*-(*2',6'*-diisopropylphenyl)-*1*-[(*1S*)-*1*-(*p*-tolyl)-*2*-phenylethyl]-*4,5*-dihydro-imidazolium Chloride**[2] (0.48 mmol). Yield: 142 mg (0.23 mmol, 47.7%), off-white solid. Mp: 210-212°C. ^{1}H NMR (500 MHz, $CDCl_3$) δ 7.37-7.24 (m, Ar-H, 5 H), 7.38 (d, J = 7.5 Hz, Ar-H, 2 H), 7.37 (t, J = 7.7 Hz, Ar-H, 1 H), 7.26 (d, J = 7.5 Hz, Ar-H, 2 H), 7.12 (dd, J = 7.7, 1.3 Hz, Ar-H, 1 H), 7.03 (dd, J = 7.7, 1.3 Hz, Ar-H, 1 H), 5.71 (dd, J = 11.8, 4.6 Hz, CH, 1 H), 3.82 (dd, J = 11.6, 10.6 Hz, CH, 1 H), 3.69/3.17 (t+t, J = 11.6 Hz, J = 10.6 Hz, CH_2, 2 H), 3.49/3.36 (dd+dd, J = 14.2, 4.6 Hz, J = 14.2, 11.8 Hz, CH_2, 2 H), 3.30 (sp, J = 6.7 Hz, CH, 1 H), 2.40 (s, CH_3, 3 H), 2.13 (sp, J = 6.7 Hz, CH, 1 H), 1.44 (d, J = 6.7 Hz, CH_3, 3 H), 1.24 (d, J = 6.7 Hz, CH_3, 3 H), 1.18 (d, J = 6.7 Hz, CH_3, 3 H), 0.85 (d, J = 6.7 Hz, CH_3, 3 H), 0.57 (s, CH_3, 9 H). ^{13}C{1H} NMR (125 MHz, $CDCl_3$) δ 208.5, 146.4, 146.2, 136.8, 134.5, 129.8, 129.3, 129.3, 129.0, 127.4, 126.9, 126.9, 125.3, 125.2, 75.2, 64.6, 64.6, 46.7, 36.7, 34.7, 28.7, 28.1, 27.6, 26.6, 26.5, 23.8, 23.5, 21.3. HRMS calcd for ($C_{34}H_{44}AgClN_2$) $[M]^+$ 622.2244, found 622.2160.

(*4S*)-*4*-*terc*-Butyl-*3*-(*2'*,*6'*-diisopropylphenyl)-*1*-[(*1R*)-*1*-(*2*-methoxyphenyl)-*2*-phenylethyl]imidazolidin-2-ylidene-silver-chloride (B21)

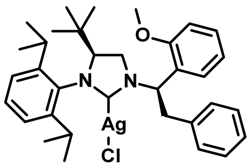

Starting material: 408 mg **(*4S*)-*4*-*terc*-Butyl-*3*-(*2'*,*6'*-diisopropylphenyl)-*1*-[(*1R*)-*1*-(*2*-methoxyphenyl)-*2*-phenylethyl] -*4*,*5*-dihydro-imidazolium Chloride**[2] (0.77 mmol). Yield: 365 mg (0.57 mmol, 74.5%), off-white solid. Mp: 237-239°C. ^{1}H NMR (500 MHz, $CDCl_3$) δ 7.42-7.28 (m, Ar-H, 5 H), 7.51 (d, J = 7.9 Hz, Ar-H, 1 H), 7.43 (t, J = 7.9 Hz, Ar-H, 1 H), 7.23 (t, J = 7.6 Hz, Ar-H, 1 H), 7.08 (t, J = 7.9 Hz, Ar-H, 1 H), 7.06 (dd, J = 7.6, 1.2 Hz, Ar-H, 1 H), 7.03 (dd, J = 7.6, 1.2 Hz, Ar-H, 1 H), 6.99 (d, J = 7.9 Hz, Ar-H, 1 H), 6.04 (dd, J = 12.5, 3.7 Hz, CH, 1 H), 3.88 (s, CH_3, 3 H), 3.84 (t, J = 11.7 Hz, CH, 1 H), 3.50/3.27 (t+m, J = 11.3 Hz, CH_2, 2 H), 3.45/3.26 (dd+dd, J = 14.0, 12.5 Hz, J = 14.0, 3.7 Hz, CH_2, 2 H), 2.69 (sp, J = 6.7 Hz, CH, 1 H), 2.53 (sp, J = 6.7 Hz, CH, 1 H), 1.20 (d, J = 6.7 Hz, CH_3, 3 H), 1.15 (d, J = 6.7 Hz, CH_3, 3 H), 1.12 (d, J = 6.7 Hz, CH_3, 3 H), 1.11 (d, J = 6.7 Hz, CH_3, 3 H), 0.65 (s, CH_3, 9 H). ^{13}C{1H} NMR (125 MHz, $CDCl_3$) δ 209.9, 158.2, 146.8, 145.8, 137.1, 130.1, 129.7, 129.1, 129.1, 127.4, 127.3, 125.7, 125.3, 124.9, 120.3, 111.2, 75.4, 59.6, 55.7, 46.5, 36.4, 34.4, 28.8, 28.4, 27.8, 27.8, 27.5, 27.0, 26.3, 23.9, 23.6, 23.4. HRMS calcd for ($C_{34}H_{44}AgN_2O$) [M - Cl]$^{+}$ 603.2505, found 603.2505.

(*4S*)-*4*-*terc*-Butyl-*3*-(*2'*,*6'*-diisopropylphenyl)-*1*-[(*1S*)-*1*-(*2*-methoxyphenyl)-*2*-phenylethyl]imidazolidin-2-ylidene-silver-chloride (B22)

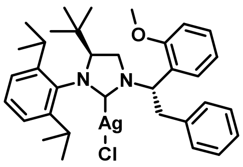

Starting material: 641 mg **(*4S*)-*4*-*terc*-Butyl-*3*-(*2'*,*6'*-diisopropylphenyl)-*1*-[(*1S*)-*1*-(*2*-methoxyphenyl)-*2*-phenylethyl] -*4*,*5*-dihydro-imidazolium Chloride**[2] (1.20 mmol). Yield: 392 mg (0.61 mmol, 51.0%), light pink solid. Mp: 188-190°C. ^{1}H NMR (500 MHz, $CDCl_3$) δ 7.52 (d, J = 7.6 Hz, Ar-H, 1 H), 7.42-7.23 (m, Ar-H, 5 H), 7.38 (t, J = 7.6 Hz, Ar-H, 1 H), 7.24 (t, J = 7.6 Hz, Ar-H, 1 H), 7.10 (dd, J = 7.6, 1.4 Hz, Ar-H, 1 H), 7.07 (t, J = 7.6 Hz, Ar-H, 1 H), 7.02 (dd, J = 7.6, 1.4 Hz, Ar-H, 1 H), 6.96 (d, J = 7.6 Hz, Ar-H, 1 H), 5.90 (dd, J = 12.2, 4.1 Hz, CH, 1 H), 3.91 (s, CH_3, 3 H), 3.80 (dd, J = 11.6, 9.6 Hz, CH, 1 H), 3.74/3.06 (t+t, J = 11.6 Hz, J = 9.6 Hz, CH_2, 2 H), 3.52/3.33 (dd+dd, J = 14.0, 12.2 Hz, J = 14.0, 4.1 Hz, CH_2, 2 H), 3.32 (sp, J = 6.7 Hz, CH, 1 H), 2.28 (sp, J = 6.7 Hz, CH, 1 H), 1.46 (d, J = 6.7 Hz, CH_3, 3 H), 1.22 (d, J = 6.7 Hz, CH_3, 3 H), 1.21 (d, J = 6.7 Hz, CH_3, 3 H), 0.84 (d, J = 6.7 Hz, CH_3, 3 H), 0.51 (s, CH_3, 9 H). ^{13}C{1H} NMR (125 MHz, $CDCl_3$) δ 209.9, 157.6, 146.8, 146.2, 146.0, 137.3, 131.6, 131.3, 130.1, 129.9, 129.3, 129.2, 129.0, 127.3, 127.2, 125.5, 125.2, 125.0, 120.3, 110.9, 74.8, 60.2, 59.3, 55.4, 55.2, 47.9, 47.8, 36.9, 34.9, 34.6, 29.1, 28.7, 27.9, 27.6, 26.4, 26.3, 26.0, 26.3, 23.9, 23.6. HRMS calcd for ($C_{34}H_{44}AgN_2O$) [M - Cl]$^{+}$ 603.2505, found 603.2499.

(*4S*)-*4*-*terc*-Butyl-*3*-(*2'*,*6'*-diisopropylphenyl)-*1*-[(*1R*)-*1*-(*o*-tolyl)-*2*-phenylethyl]imidazolidin-2-ylidene-silver-chloride (B23)

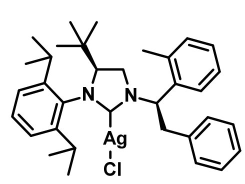

Starting material: 134 mg **(*4S*)-*4*-*terc*-Butyl-*3*-(*2'*,*6'*-diisopropylphenyl)-*1*-[(*1R*)-*1*-(*o*-tolyl)-*2*-phenylethyl] -*4*,*5*-dihydro-imidazolium Chloride**[2] (0.26 mmol). Yield: 113 mg (0.18 mmol, 69.9%), off-white solid. Mp: 253-255°C. ^{1}H NMR (500 MHz, $CDCl_3$) δ 7.56 (m, Ar-H, 1 H), 7.48-7.32 (m, Ar-H, 5 H), 7.40-7.30 (m, Ar-H, 3 H), 7.24 (t, J = 7.7Hz, Ar-H, 1 H), 7.07 (dd, J = 7.7, 1.4 Hz, Ar-H, 1 H), 7.03 (dd, J = 7.7, 1.4 Hz, Ar-H, 1 H), 5.76 (dd, J = 12.1, 3.3 Hz, CH, 1 H), 3.85 (t, J = 11.6 Hz, CH, 1 H), 3.51/3.16 (t+t, J = 11.6 Hz, J = 11.6 Hz, CH_2, 2 H), 3.49/3.32 (t+dd, J = 12.1 Hz, J = 13.7, 3.3 Hz, CH_2, 2 H), 2.72 (sp, J = 6.7 Hz, CH, 1 H), 2.54 (sp, J = 6.7 Hz, CH, 1 H), 2.44 (s, CH_3, 3 H), 1.21 (d, J = 6.7 Hz, CH_3, 3 H), 1.19 (d, J = 6.7 Hz, CH_3, 3 H), 1.08 (d, J = 6.7 Hz, CH_3, 3 H), 1.08 (d, J = 6.7 Hz, CH_3, 3 H), 0.71 (s, CH_3, 9 H). ^{13}C{1H} NMR (125 MHz, $CDCl_3$) δ 209.7, 146.7, 145.7, 138.5, 137.0, 136.5, 135.2, 131.8, 129.9, 129.5, 129.3, 128.9, 127.7, 126.7,

126.4, 125.4, 125.0, 75.7, 63.2, 46.3, 37.0, 34.5, 28.9, 28.1, 27.4, 27.0, 26.3, 23.6, 23.5, 20.4. HRMS calcd for ($C_{34}H_{44}AgN_2$) [M - Cl]$^+$ 587.2554, found 587.2551.

(*4S*)-*4-terc*-Butyl-*3*-(*2',6'*-diisopropylphenyl)-*1*-[(*1S*)-*1*-(*o*-tolyl)-*2*-phenylethyl]imidazolidin-2-ylidene-silver-chloride (B24)

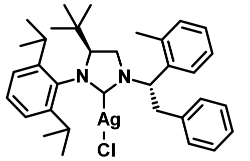

Starting material: 95 mg **(*4S*)-*4-terc*-Butyl-*3*-(*2',6'*-diisopropylphenyl)-*1*-[(*1S*)-*1*-(*o*-tolyl)-*2*-phenylethyl] -*4,5*-dihydro-imidazolium Chloride**[2] (0.18 mmol). Yield: 47 mg (0.08 mmol, 41.0%), white solid. Mp: 243-245°C. ^{1}H NMR (500 MHz, $CDCl_3$) δ 7.55 (m, Ar-H, 1 H), 7.39 (m, Ar-H, 4 H), 7.36-7.24 (m, Ar-H, 3 H), 7.26 (m, Ar-H, 1 H), 7.25 (t, J = 7.7, 1.5 Hz, Ar-H, 1 H), 7.11 (dd, J = 7.7, 1.5 Hz, Ar-H, 1 H), 7.04 (dd, J = 7.7, 1.5 Hz, Ar-H, 1 H), 5.77 (dd, J = 11.3, 4.3 Hz, CH, 1 H), 3.88 (dd, J = 11.9, 10.0 Hz, CH, 1 H), 3.75/3.06 (t+t, J = 11.9 Hz, J = 10.0 Hz, CH_2, 2 H), 3.53/3.35 (dd+dd, J = 14.1, 11.3 Hz, J = 14.1, 4.3 Hz, CH_2, 2 H), 3.26 (sp, J = 6.7 Hz, CH, 1 H), 2.49 (s, CH_3, 3 H), 2.36 (sp, J = 6.7 Hz, CH, 1 H), 1.41(d, J = 6.7 Hz, CH_3, 3 H), 1.25 (d, J = 6.7 Hz, CH_3, 3 H), 1.22 (d, J = 6.7 Hz, CH_3, 3 H), 0.92 (d, J = 6.7 Hz, CH_3, 3 H), 0.53 (s, CH_3, 9 H). ^{13}C{1H} NMR (125 MHz, $CDCl_3$) δ 209.9, 146.3, 145.9, 137.7, 137.0, 136.5, 135.3, 131.8, 129.6, 129.3, 129.2, 128.8, 127.5, 126.4,126.3, 125.3, 125.1, 74.6, 62.4, 47.2, 37.5, 34.8, 28.8, 28.1, 27.5, 26.6, 26.3, 24.0, 23.6, 20.6. HRMS calcd for ($C_{34}H_{44}AgN_2$) [M - Cl]$^+$ 587.2554, found 587.2557.

(*4S*)-*4-terc*-Butyl-*3*-(*2',6'*-diisopropylphenyl)-*1*-[(*1R*)-*1*-(*3*-methoxyphenyl)-*2*-phenylethyl]imidazolidin-2-ylidene-silver-chloride (B25)

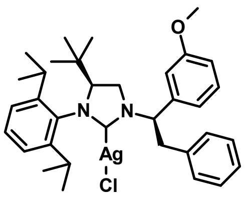

Starting material: 200 mg **(*4S*)-*4-terc*-Butyl-*3*-(*2',6'*-diisopropylphenyl)-*1*-[(*1R*)-*1*-(*3*-methoxyphenyl)-*2*-phenylethyl] -*4,5*-dihydroimidazolium Chloride**[2] (0.38 mmol). Yield: 189 mg (0.30 mmol, 78.7%), off-white solid. Mp: 195-197°C. ^{1}H NMR (400 MHz, $CDCl_3$) δ 7.44 (t, J = 8.2 Hz, Ar-H, 1 H), 7.44 (t, J = 7.0 Hz, Ar-H, 2 H), 7.41 (d, J = 7.0 Hz, Ar-H, 2 H), 7.35 (t, J = 7.0 Hz, Ar-H, 1 H), 7.29 (t, J = 7.7 Hz, Ar-H, 1 H), 7.12 (d, J = 7.7, 1.5 Hz, Ar-H, 1 H), 7.10 (dd, J = 8.2, 2.4 Hz, Ar-H, 1 H), 7.09 (d, J = 7.7, 1.5 Hz, Ar-H, 1 H), 7.03 (t, J = 1.9 Hz, Ar-H, 1 H), 6.99 (dd, J = 8.2, 2.4 Hz, Ar-H, 1 H), 5.75 (dd, J = 12.2, 4.2 Hz, CH, 1 H), 3.94 (t, J = 11.6 Hz, CH, 1 H), 3.89 (s, CH_3, 3 H), 3.53/3.32 (dd+dd, J = 14.2, 4.2 Hz, J = 14.2, 12.2 Hz, CH_2, 2 H), 3.47/3.35 (t+t, J = 11.6 Hz, J = 11.6 Hz, CH_2, 2 H), 2.75 (sp, J = 6.7 Hz, CH, 1 H), 2.64 (sp, J = 6.7 Hz, CH, 1 H), 1.27 (d, J = 6.7 Hz, CH_3, 3 H), 1.20 (d, J = 6.7 Hz, CH_3, 3 H), 1.20 (d, J = 6.7 Hz, CH_3, 3 H), 1.19 (d, J = 6.7 Hz, CH_3, 3 H), 0.68 (s, CH_3, 9 H). ^{13}C{1H} NMR (100 MHz, $CDCl_3$) δ 209.1, 160.3, 146.7, 145.9, 139.3, 136.4, 130.2, 129.6, 129.4, 129.3, 127.4, 125.4, 125.0, 119.3, 114.2, 113.0, 76.9, 65.9, 55.5, 55.5, 46.3, 36.1, 34.4, 29.1, 27.9, 27.5, 26.9, 26.2, 23.6, 23.5. HRMS calcd for ($C_{34}H_{44}AgN_2O$) [M - Cl]$^+$ 603.2505, found 603.2501.

(*4S*)-*4-terc*-Butyl-*3*-(*2',6'*-diisopropylphenyl)-*1*-[(*1S*)-*1*-(*3*-methoxyphenyl)-*2*-phenylethyl]imidazolidin-2-ylidene-silver-chloride (B26)

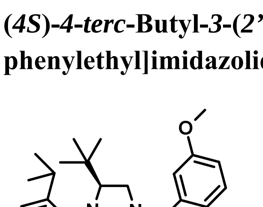

Starting material: 150 mg **(*4S*)-*4-terc*-Butyl-*3*-(*2',6'*-diisopropylphenyl)-*1*-[(*1S*)-*1*-(*3*-methoxyphenyl)-*2*-phenylethyl] -*4,5*-dihydroimidazolium Chloride**[2] (0.28 mmol). Yield: 90 mg (0.14 mmol, 50.0%), white solid. Mp: 245-247°C. ^{1}H NMR (400 MHz, $CDCl_3$) δ 7.43 (t, J = 7.9 Hz, Ar-H, 1 H), 7.42 (t, J = 6.5 Hz, Ar-H, 2 H), 7.40 (d, J = 6.5 Hz, Ar-H, 2 H), 7.32 (t, J = 6.5 Hz, Ar-H, 1 H), 7.29 (t, J = 7.7 Hz, Ar-H, 1 H), 7.15 (dd, J = 7.7, 1.5 Hz, Ar-H, 1 H), 7.10 (dd, J = 7.9, 2.4 Hz, Ar-H, 1 H), 7.07 (dd, J = 7.7, 1.5 Hz, Ar-H, 1 H), 7.05 (t, J = 1.9 Hz, Ar-H, 1 H), 6.97 (dd, J = 7.9, 2.4 Hz, Ar-H, 1 H), 5.74 (dd, J = 11.9, 4.6 Hz, CH, 1 H), 3.88 (s, CH_3, 3 H), 3.87 (dd, J = 11.5, 10.7 Hz, CH, 1 H), 3.73/3.22 (t+t, J = 11.5 Hz, J = 10.7 Hz,

CH_2, 2 H), 3.55/3.39 (dd+dd, *J* = 14.3, 4.6 Hz, *J* = 14.3, 11.9 Hz, CH_2, 2 H), 3.35 (sp, *J* = 6.7 Hz, CH, 1 H), 2.16 (sp, *J* = 6.7 Hz, CH, 1 H), 1.46 (d, *J* = 6.7 Hz, CH_3, 3 H), 1.28 (d, *J* = 6.7 Hz, CH_3, 3 H), 1.21 (d, *J* = 6.7 Hz, CH_3, 3 H), 0.89 (d, *J* = 6.7 Hz, CH_3, 3 H), 0.61 (s, CH_3, 9 H). $^{13}C\{1H\}$ NMR (100 MHz, $CDCl_3$) δ 209.3, 160.2, 146.3, 146.2, 139.2, 136.9, 136.4, 130.2, 129.3, 129.3, 129.0, 127.4, 125.2, 119.1, 114.3, 112.6, 75.2, 64.7, 55.4, 46.8, 36.5, 34.6, 28.7, 28.2, 27.6, 26.5, 26.5, 23.8, 23.5. HRMS calcd for ($C_{34}H_{44}AgN_2O$) $[M - Cl]^+$ 603.2505, found 603.2501.

(*4S*)-*4*-*terc*-Butyl-*3*-(*2',6'*-diisopropylphenyl)-*1*-[(*1R*)-*2*-(*p*-tolyl)-*1*-phenylethyl]imidazolidin-2-ylidene-silver-chloride (B27)

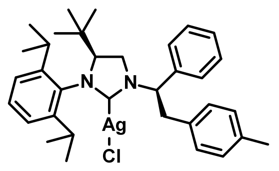

Starting material: 65 mg **(*4S*)-*4*-*terc*-Butyl-*3*-(*2',6'*-diisopropylphenyl)-*1*-[(*1R*)-*2*-(*p*-tolyl)-*1*-phenylethyl] -*4,5*-dihydroimidazolium Chloride**[2] (0.13 mmol). Yield: 58 mg (0.09 mmol, 74.0%), off-white solid. Mp: 255-257°C. ^1H NMR (500 MHz, $CDCl_3$) δ 7.55-7.41 (m, Ar-H, 5 H), 7.30 (m, Ar-H, 2 H), 7.29 (t, *J* = 7.7 Hz, Ar-H, 1 H), 7.24 (m, Ar-H, 2 H), 7.11 (dd, *J* = 7.7, 1.2 Hz, Ar-H, 1 H), 7.09 (dd, *J* = 7.7, 1.2 Hz, Ar-H, 1 H), 5.79 (dd, *J* = 12.2, 4.2 Hz, CH, 1 H), 3.93 (t, *J* = 11.8 Hz, CH, 1 H), 3.48/3.31 (m+m, CH_2, 2 H), 3.47/3.32 (m+m, CH_2, 2 H), 2.77 (sp, *J* = 6.7 Hz, CH, 1 H), 2.62 (sp, *J* = 6.7 Hz, CH, 1 H), 2.40 (s, CH_3, 3 H), 1.26 (d, *J* = 6.7 Hz, CH_3, 3 H), 1.21 (d, *J* = 6.7 Hz, CH_3, 3 H), 1.19 (d, *J* = 6.7 Hz, CH_3, 3 H), 1.19 (d, *J* = 6.7 Hz, CH_3, 3 H), 0.67 (s, CH_3, 9 H). $^{13}C\{1H\}$ NMR (125 MHz, $CDCl_3$) δ 208.9, 146.7, 145.9, 137.7, 136.9, 136.6, 133.3, 129.9, 129.5, 129.4, 129.2, 128.7, 127.3, 125.3, 125.1, 75.9, 65.0, 46.2, 46.1, 35.6, 34.4, 29.0, 27.7, 27.5, 27.5, 26.9, 26.2, 23.5, 21.4. HRMS calcd for ($C_{34}H_{44}AgN_2$) $[M - Cl]^+$ 587.2554, found 587.2525.

(*4S*)-*4*-*terc*-Butyl-*3*-(*2',6'*-diisopropylphenyl)-*1*-[(*1S*)-*2*-(*p*-tolyl)-*1*-phenylethyl]imidazolidin-2-ylidene-silver-chloride (B28)

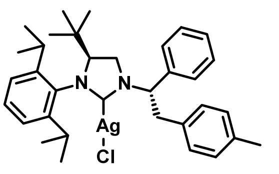

Starting material: 190 mg **(*4S*)-*4*-*terc*-Butyl-*3*-(*2',6'*-diisopropylphenyl)-*1*-[(*1S*)-*2*-(*p*-tolyl)-*1*-phenylethyl]-*4,5*-dihydroimidazolium Chloride**[2] (0.37 mmol). Yield: 118 mg (0.19 mmol, 51.5%), off-white solid. Mp: 217-219°C. ^1H NMR (500 MHz, $CDCl_3$) δ 7.54-7.36 (m, Ar-H, 5 H), 7.29 (m, Ar-H, 2 H), 7.28 (t, *J* = 7.7 Hz, Ar-H, 1 H), 7.21 (m, Ar-H, 2 H), 7.15 (dd, *J* = 7.7, 1.3 Hz, Ar-H, 1 H), 7.05 (dd, *J* = 7.7, 1.3 Hz, Ar-H, 1 H), 5.76 (dd, *J* = 12.0, 4.4 Hz, CH, 1 H), 3.85 (dd, *J* = 11.6, 10.3 Hz, CH, 1 H), 3.74/3.18 (t+t, *J* = 11.6 Hz, *J* = 10.3 Hz, CH, 2 H), 3.50/3.36 (dd+dd, *J* = 14.4, 4.4 Hz, *J* = 14.4, 12.0 Hz, CH_2, 2 H), 3.32 (sp, *J* = 6.7 Hz, CH, 1 H), 2.35 (s, CH_3, 3 H), 2.18 (sp, *J* = 6.7 Hz, CH, 1 H), 1.47 (d, *J* = 6.7 Hz, CH_3, 3 H), 1.27 (d, *J* = 6.7 Hz, CH_3, 3 H), 1.21 (d, *J* = 6.7 Hz, CH_3, 3 H), 0.86 (d, *J* = 6.7 Hz, CH_3, 3 H), 0.59 (s, CH_3, 9 H). $^{13}C\{1H\}$ NMR (125 MHz, $CDCl_3$) δ 208.8, 146.4, 146.1, 137.7, 136.8, 136.6, 133.5, 130.0, 129.3, 129.2, 128.9, 128.7, 127.1, 125.3, 125.2, 75.2, 64.8, 46.7, 46.6, 36.0, 34.7, 28.7, 28.2, 27.6, 26.4, 26.3, 23.8, 23.5, 21.3. HRMS calcd for ($C_{34}H_{44}AgN_2$) $[M - Cl]^+$ 587.2554, found 587.2511.

(*4S*)-*4*-*terc*-Butyl-*3*-(*2',6'*-diisopropylphenyl)-*1*-[(*1R*)-*2*-(*4*-chlorophenyl)-*1*-phenylethyl]imidazolidin-2-ylidene-silver-chloride (B29)

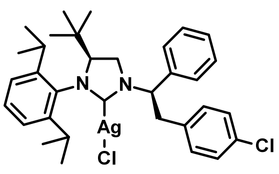

Starting material: 230 mg **(*4S*)-*4*-*terc*-Butyl-*3*-(*2',6'*-diisopropylphenyl)-*1*-[(*1R*)-*2*-(*4*-chlorophenyl)-*1*-phenylethyl] -*4,5*-dihydroimidazolium Chloride**[2] (0.43 mmol). Yield: 198 mg (0.31 mmol, 71.8%), off-white solid. Mp: 257-259°C. ^1H NMR (500 MHz, DMSO-d_6) δ 7.54 (d, *J* = 8.1 Hz, Ar-H, 2 H), 7.53 (m, Ar-H, 4 H), 7.44 (m, Ar-H, 1 H), 7.36 (d, *J* = 8.1 Hz, Ar-H, 2 H), 7.31 (t, *J* = 7.7 Hz, Ar-H, 1 H), 7.19 (dm, *J* = 7.7 Hz, Ar-H, 1 H), 7.18 (dm, *J* = 7.7 Hz, Ar-H, 1 H), 5.64 (dd, *J* = 12.2, 3.9 Hz, CH, 1 H), 3.96 (t, *J* = 11.9 Hz, CH, 1 H),

3.69/3.34 (t+t, *J* = 11.9 Hz, *J* = 11.9 Hz, CH_2, 2 H), 3.61/3.42 (dd+dd, *J* = 13.8, 12.2 Hz, *J* = 13.8, 3.9 Hz, CH_2, 2 H), 2.68 (sp, *J* = 6.7 Hz, CH, 1 H), 2.64 (sp, *J* = 6.7 Hz, CH, 1 H), 1.23 (d, *J* = 6.7 Hz, CH_3, 3 H), 1.16 (d, *J* = 6.7 Hz, CH_3, 3 H), 1.13 (d, *J* = 6.7 Hz, CH_3, 3 H), 1.09 (d, *J* = 6.7 Hz, CH_3, 3 H), 0.62 (s, CH_3, 9 H). $^{13}C\{1H\}$ NMR (125 MHz, DMSO-d_6) δ 207.0, 146.2, 145.9, 137.9, 136.6, 131.5, 131.3, 129.0, 128.9, 128.4, 128.3, 127.1, 124.8, 124.7, 75.3, 75.2, 64.7, 46.2, 46.1, 34.1, 34.0, 28.3, 27.2, 26.6, 26.3, 25.7, 23.0, 22.9. HRMS calcd for ($C_{33}H_{41}AgCl_2N_2$) $[M]^+$ 642.1698, found 642.1652.

(*4S*)-*4*-*terc*-Butyl-*3*-(*2',6'*-diisopropylphenyl)-*1*-[(*1S*)-*2*-(*4*-chlorophenyl)-*1*-phenylethyl]imidazolidin-2-ylidene-silver-chloride (B30)

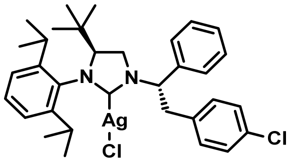

Starting material: 185 mg **(*4S*)-*4*-*terc*-Butyl-*3*-(*2',6'*-diisopropylphenyl)-*1*-[(*1S*)-*2*-(*4*-chlorophenyl)-*1*-phenylethyl] -*4,5*-dihydroimidazolium Chloride**[2] (0.34 mmol). Yield: 195 mg (0.88 mmol, 87.9%), light pink solid. Mp: 199-201°C. ^1H NMR (500 MHz, DMSO-d_6) δ 7.55 (d, *J* = 7.2 Hz, Ar-H, 2 H), 7.53 (d, *J* = 8.2 Hz, Ar-H, 2 H), 7.51 (t, *J* = 7.2 Hz, Ar-H, 2 H), 7.42 (t, *J* = 7.2 Hz, Ar-H, 1 H), 7.39 (d, *J* = 8.2 Hz, Ar-H, 2 H), 7.32 (t, *J* = 7.7 Hz, Ar-H, 1 H), 7.22 (d, *J* = 7.7 Hz, Ar-H, 1 H), 7.17 (d, *J* = 7.7 Hz, Ar-H, 1 H), 5.59 (dd, *J* = 12.0, 4.3 Hz, CH, 1 H), 4.07/3.10 (t+t, *J* = 11.6 Hz, *J* = 11.6 Hz, CH_2, 2 H), 3.82 (t, *J* = 11.6 Hz, CH, 1 H), 3.57/3.47 (dd+dd, *J* = 13.9, 12.0 Hz, *J* = 13.9, 4.3 Hz, CH_2, 2 H), 3.22 (sp, *J* = 6.7 Hz, CH, 1 H), 2.19 (sp, *J* = 6.7 Hz, CH, 1 H), 1.31 (d, *J* = 6.7 Hz, CH_3, 3 H), 1.21 (d, *J* = 6.7 Hz, CH_3, 3 H), 1.21 (d, *J* = 6.7 Hz, CH_3, 3 H), 0.82 (d, *J* = 6.7 Hz, CH_3, 3 H), 0.52 (s, CH_3, 9 H). $^{13}C\{1H\}$ NMR (125 MHz, DMSO-d_6) δ 207.2, 146.2, 145.9, 138.1, 136.8, 136.5, 131.3, 131.0, 129.1, 128.9, 128.5, 128.3, 126.9, 125.0, 124.7, 74.7, 64.2, 47.2, 35.0, 33.9, 28.1, 27.5, 26.8, 25.9, 25.7, 23.1, 22.9. HRMS calcd for ($C_{33}H_{41}AgCl_2N_2$) $[M]^+$ 642.1698, found 642.1623.

(*4S*)-*4*-*terc*-Butyl-*3*-(*2',6'*-diisopropylphenyl)-*1*-[(*1R*)-*2*-(*2*-chlorophenyl)-*1*-phenylethyl]imidazolidin-2-ylidene-silver-chloride (B31)

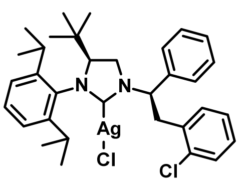

Starting material: 300 mg **(*4S*)-*4*-*terc*-Butyl-*3*-(*2',6'*-diisopropylphenyl)-*1*-[(*1R*)-*2*-(*2*-chlorophenyl)-*1*-phenylethyl] -*4,5*-dihydroimidazolium Chloride**[2] (0.56 mmol). Yield: 291 mg (0.45 mmol, 80.9%), white solid. Mp: 217-219°C. ^1H NMR (500 MHz, $CDCl_3$) δ 7.54-7.42 (m, Ar-H, 5 H), 7.52-7.24 (m, Ar-H, 4 H), 7.28 (t, *J* = 7.8 Hz, Ar-H, 1 H), 7.11 (dd, *J* = 7.8, 1.5 Hz, Ar-H, 1 H), 7.08 (dd, *J* = 7.8, 1.5 Hz, Ar-H, 1 H), 5.88 (dd, *J* = 11.8, 4.1 Hz, CH, 1 H), 3.92 (t, *J* = 11.6 Hz, CH, 1 H), 3.68/3.52 (dd+dd, *J* = 14.4, 11.8 Hz, *J* = 14.4, 4.1 Hz, CH_2, 2 H), 3.53/3.32 (t+t, *J* = 11.6 Hz, *J* = 11.6 Hz, CH_2, 2 H), 2.86 (sp, *J* = 6.7 Hz, CH, 1 H), 2.60 (sp, *J* = 6.7 Hz, CH, 1 H), 1.25 (d, *J* = 6.7 Hz, CH_3, 3 H), 1.20 (d, *J* = 6.7 Hz, CH_3, 3 H), 1.19 (d, *J* = 6.7 Hz, CH_3, 3 H), 1.19 (d, *J* = 6.7 Hz, CH_3, 3 H), 0.67 (s, CH_3, 9 H). $^{13}C\{1H\}$ NMR (125 MHz, $CDCl_3$) δ 209.1, 146.6, 146.0, 137.5, 136.6, 134.5, 134.3, 131.4, 130.5, 129.5, 129.3, 128.9, 128.8, 127.5, 127.3, 125.3, 125.1, 76.2, 66.0, 63.4, 46.6, 34.4, 33.2, 29.0, 27.8, 27.7, 26.8, 26.2, 15.4. HRMS calcd for ($C_{33}H_{41}AgClN_2$) $[M - Cl]^+$ 607.2009, found 607.2004.

(*4S*)-*4*-*terc*-Butyl-*3*-(*2',6'*-diisopropylphenyl)-*1*-[(*1S*)-*2*-(*2*-chlorophenyl)-*1*-phenylethyl]imidazolidin-2-ylidene-silver-chloride (B32)

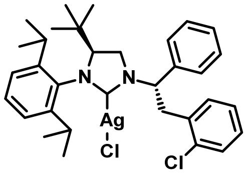

Starting material: 500 mg **(*4S*)-*4*-*terc*-Butyl-*3*-(*2',6'*-diisopropylphenyl)-*1*-[(*1S*)-*2*-(*2*-chlorophenyl)-*1*-phenylethyl] -*4,5*-dihydroimidazolium Chloride**[2] (0.93 mmol). Yield: 312 mg (0.48 mmol, 52.0%), white solid. Mp: 211-213°C. ^1H NMR (500 MHz, $CDCl_3$) δ 7.53-7.40 (m, Ar-H, 5 H), 7.51-7.20 (m, Ar-H, 4 H), 7.27 (t, *J* = 7.8 Hz, Ar-H, 1 H), 7.13 (dd, *J* = 7.8, 1.5 Hz, Ar-H, 1 H), 7.05 (dd, *J* = 7.8, 1.5 Hz, Ar-H, 1 H), 5.81 (dd, *J* = 11.8, 3.9 Hz,

CH, 1 H), 3.93 (m, CH, 1 H), 3.93/3.09 (m+m, CH_2, 2 H), 3.83/3.44 (dd+dd, *J* = 14.2, 11.8 Hz, *J* = 14.2, 3.9 Hz, CH_2, 2 H), 3.31 (sp, *J* = 6.7 Hz, CH, 1 H), 2.39 (sp, *J* = 6.7 Hz, CH, 1 H), 1.45 (d, *J* = 6.7 Hz, CH_3, 3 H), 1.26 (d, *J* = 6.7 Hz, CH_3, 3 H), 1.26 (d, *J* = 6.7 Hz, CH_3, 3 H), 0.90 (d, *J* = 6.7 Hz, CH_3, 3 H), 0.57 (s, CH_3, 9 H). $^{13}C\{1H\}$ NMR (125 MHz, $CDCl_3$) δ 208.5, 146.3, 146.1, 137.3, 136.4, 134.8, 134.1, 131.4, 130.2, 129.3, 129.2, 128.9, 128.8, 128.4, 127.2, 125.3, 125.2, 75.3, 64.6, 47.1, 34.6, 33.5, 28.8, 28.6, 28.2, 27.6, 26.6, 26.5, 23.9, 23.6. HRMS calcd for ($C_{33}H_{41}AgClN_2$) $[M - Cl]^+$ 607.2009, found 607.2006.

(*4R*)-*4-terc*-Butyl-*3*-(*2',6'*-diisopropylphenyl)-*1*-[(*1R*)-*2*-(*3*-chlorophenyl)-*1*-phenylethyl]imidazolidin-2-ylidene-silver-chloride (B35)

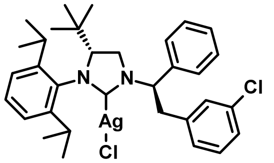

Starting material: 500 mg **(*4R*)-*4-terc*-Butyl-*3*-(*2',6'*-diisopropylphenyl)-*1*-[(*1R*)-*2*-(*3*-chlorophenyl)-*1*-phenylethyl] -*4,5*-dihydroimidazolium Chloride**[2] (0.93 mmol). Yield: 226 mg (0.35 mmol, 37.7%), brown solid. Mp: 202-204°C. ^1H NMR (500 MHz, $CDCl_3$) δ 7.52-7.40 (m, Ar-H, 5 H), 7.42-7.25 (m, Ar-H, 4 H), 7.28 (t, *J* = 7.7 Hz, Ar-H, 1 H), 7.14 (dd, *J* = 7.7, 1.5 Hz, Ar-H, 1 H), 7.06 (dd, *J* = 7.7, 1.5 Hz, Ar-H, 1 H), 5.71 (dd, *J* = 11.8, 4.7 Hz, CH, 1 H), 3.88 (dd, *J* = 11.6, 10.5 Hz, CH, 1 H), 3.71/3.17 (t+t, *J* = 11.6 Hz, *J* = 10.5 Hz, CH_2, 2 H), 3.51/3.42 (dd+dd, *J* = 14.3, 4.7 Hz, *J* = 14.3, 11.8 Hz, CH_2, 2 H), 3.30 (sp, *J* = 6.7 Hz, CH, 1 H), 2.19 (sp, *J* = 6.7 Hz, CH, 1 H), 1.46 (d, *J* = 6.7 Hz, CH_3, 3 H), 1.27 (d, *J* = 6.7 Hz, CH_3, 3 H), 1.25 (d, *J* = 6.7 Hz, CH_3, 3 H), 0.89 (d, *J* = 6.7 Hz, CH_3, 3 H), 0.58 (s, CH_3, 9 H). $^{13}C\{1H\}$ NMR (125 MHz, $CDCl_3$) δ 208.8, 146.3, 146.1, 138.9, 137.2, 136.4, 134.6, 131.1, 129.4, 129.3, 129.2, 128.9, 127.7, 127.4, 127.0, 125.3, 125.2, 75.3, 64.6, 47.2, 36.4, 29.0, 28.2, 27.7, 26.4, 26.2, 23.7, 23.5. HRMS calcd for ($C_{33}H_{41}AgClN_2$) $[M - Cl]^+$ 607.2009, found 607.2004.

(*4R*)-*4-terc*-Butyl-*3*-(*2',6'*-diisopropylphenyl)-*1*-[(*1S*)-*2*-(*3*-chlorophenyl)-*1*-phenylethyl]imidazolidin-2-ylidene-silver-chloride (B36)

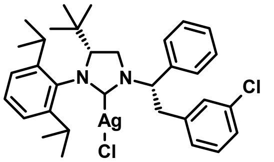

Starting material: 500 mg **(*4R*)-*4-terc*-Butyl-*3*-(*2',6'*-diisopropylphenyl)-*1*-[(*1S*)-*2*-(*3*-chlorophenyl)-*1*-phenylethyl] -*4,5*-dihydroimidazolium Chloride**[2] (0.93 mmol). Yield: 371 mg (0.58 mmol, 61.9%), light brown solid. Mp: 242-244°C. ^1H NMR (500 MHz, $CDCl_3$) δ 7.54-7.43 (m, Ar-H, 5 H), 7.50-7.24 (m, Ar-H, 4 H), 7.28 (t, *J* = 7.7 Hz, Ar-H, 1 H), 7.12 (dd, *J* = 7.7, 1.5 Hz, Ar-H, 1 H), 7.08 (dd, *J* = 7.7, 1.5 Hz, Ar-H, 1 H), 5.73 (dd, *J* = 12.3, 3.8 Hz, CH, 1 H), 3.93 (t, *J* = 11.7 Hz, CH, 1 H), 3.48/3.31 (dd+dd, *J* = 14.0, 3.8 Hz, *J* = 14.0, 12.3 Hz, CH_2, 2 H), 3.47/3.31 (t+t, *J* = 11.7 Hz, *J* = 11.7 Hz, CH_2, 2 H), 2.82 (sp, *J* = 6.7 Hz, CH, 1 H), 2.59 (sp, *J* = 6.7 Hz, CH, 1 H), 1.25 (d, *J* = 6.7 Hz, CH_3, 3 H), 1.24 (d, *J* = 6.7 Hz, CH_3, 3 H), 1.20 (d, *J* = 6.7 Hz, CH_3, 3 H), 1.18 (d, *J* = 6.7 Hz, CH_3, 3 H), 0.72 (s, CH_3, 9 H). $^{13}C\{1H\}$ NMR (125 MHz, $CDCl_3$) δ 209.1, 146.7, 145.8, 138.7, 137.2, 136.5, 134.4, 131.5, 129.6, 129.5, 129.4, 129.0, 127.9, 127.6, 127.2, 125.5, 125.1, 76.2, 65.3, 46.2, 35.7, 29.0, 27.9, 27.7, 26.9, 26.2, 23.6, 23.5. HRMS calcd for ($C_{33}H_{41}AgClN_2$) $[M - Cl]^+$ 607.2009, found 607.2004.

(*4R*)-*4-terc*-Butyl-*3*-(*2',6'*-diisopropylphenyl)-*1*-[(*1R*)-*1*-(*3*-trifluoromethyl)-*2*-phenylethyl]imidazolidin-2-ylidene-silver-chloride (B37)

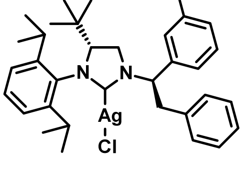

Starting material: 475 mg **(*4R*)-*4-terc*-Butyl-*3*-(*2',6'*-diisopropylphenyl)-*1*-[(*1R*)-*1*-(*3*-trifluoromethyl)-*2*-phenylethyl] -*4,5*-dihydroimidazolium Chloride**[2] (0.83 mmol). Yield: 363 mg (0.54 mmol, 64.4%), white solid. Mp: 215-217°C. ^1H NMR (500 MHz, $CDCl_3$) δ 7.72 (m, Ar-H, 1 H), 7.71 (m, Ar-H, 1 H), 7.71 (m, Ar-H, 1 H), 7.64 (m, Ar-H, 1 H), 7.48-7.32 (m, Ar-H, 5 H), 7.29 (t, *J* = 7.7 Hz, Ar-H, 1 H), 7.11 (dd, *J* = 7.7, 1.5 Hz, Ar-H, 1 H), 7.09 (dd,

J = 7.7, 1.5 Hz, Ar-H, 1 H), 5.84 (dd, *J* = 12.2, 4.1 Hz, CH, 1 H), 3.95 (t, *J* = 11.6 Hz, CH, 1 H), 3.60/3.33 (dd+dd, *J* = 14.0, 4.1 Hz, *J* = 14.0, 12.2 Hz, CH_2, 2 H), 3.46/3.24 (t+t, *J* = 11.6 Hz, *J* = 11.6 Hz, CH_2, 2 H), 2.73 (sp, *J* = 6.7 Hz, CH, 1 H), 2.61 (sp, *J* = 6.7 Hz, CH, 1 H), 1.27 (d, *J* = 6.7 Hz, CH_3, 3 H), 1.19 (d, *J* = 6.7 Hz, CH_3, 3 H), 1.19 (d, *J* = 6.7 Hz, CH_3, 3 H), 1.18 (d, *J* = 6.7 Hz, CH_3, 3 H), 0.66 (s, CH_3, 9 H). ^{13}C{1H} NMR (125 MHz, $CDCl_3$) δ 209.8, 146.6, 145.9, 139.0, 136.3, 135.8, 131.8, 131.6, 130.5, 130.0, 129.6, 129.5, 129.4, 127.6, 125.7, 125.4, 125.1, 123.8, 76.2, 64.4, 46.3, 35.9, 29.2, 27.8, 27.6, 26.9, 26.0, 23.5, 23.4. HRMS calcd for ($C_{34}H_{41}AgF_3N_2$) $[M - Cl]^+$ 641.2273, found 641.2268.

(*4R*)-*4*-*terc*-Butyl-*3*-(*2'*,*6'*-diisopropylphenyl)-*1*-[(*1S*)-*1*-(*3*-trifluoromethyl)-*2*-phenylethyl]imidazolidin-2-ylidene-silver-chloride (B38)

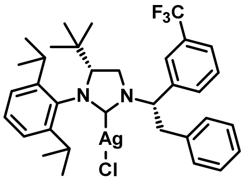

Starting material: 200 mg **(*4R*)-*4*-*terc*-Butyl-*3*-(*2'*,*6'*-diisopropylphenyl)-*1*-[(*1S*)-*1*-(*3*-trifluoromethyl)-*2*-phenylethyl] -*4*,*5*-dihydroimidazolium Chloride**[2] (0.35 mmol). Yield: 124 mg (0.18 mmol, 52.2%), white solid. Mp: 201-212°C. ^{1}H NMR (500 MHz, $CDCl_3$) δ 7.71 (m, Ar-H, 1 H), 7.70 (m, Ar-H, 1 H), 7.69 (m, Ar-H, 1 H), 7.63 (m, Ar-H, 1 H), 7.44-7.28 (m, Ar-H, 5 H), 7.28 (t, *J* = 7.7 Hz, Ar-H, 1 H), 7.14 (dd, *J* = 7.7, 1.5 Hz, Ar-H, 1 H), 7.04 (dd, *J* = 7.7, 1.5 Hz, Ar-H, 1 H), 5.83 (dd, *J* = 12.0, 4.7 Hz, CH, 1 H), 3.86 (dd, *J* = 11.6, 10.6 Hz, CH, 1 H), 3.70/3.10 (t+t, *J* = 11.6 Hz, *J* = 10.6 Hz, CH_2, 2 H), 3.62/3.36 (dd+dd, *J* = 14.3, 4.7 Hz, *J* = 14.3, 12.0 Hz, CH_2, 2 H), 3.32 (sp, *J* = 6.7 Hz, CH, 1 H), 2.12 (sp, *J* = 6.7 Hz, CH, 1 H), 1.45 (d, *J* = 6.7 Hz, CH_3, 3 H), 1.27 (d, *J* = 6.7 Hz, CH_3, 3 H), 1.18 (d, *J* = 6.7 Hz, CH_3, 3 H), 0.87 (d, *J* = 6.7 Hz, CH_3, 3 H), 0.58 (s, CH_3, 9 H). ^{13}C{1H} NMR (125 MHz, $CDCl_3$) δ 209.7, 146.3, 146.0, 138.9, 136.2, 136.0, 131.6, 131.4, 130.3, 130.0, 129.5, 129.4, 129.0, 127.6, 125.6, 125.3, 125.2, 123.6, 75.4, 64.0, 46.8, 36.3, 34.6, 28.7, 28.3, 27.5, 26.5, 26.4, 23.8, 23.4. HRMS calcd for ($C_{34}H_{41}AgF_3N_2$) $[M - Cl]^+$ 641.2273, found 641.2270.

***3*-(*2'*,*6'*-diisopropylphenyl)-*1*-[(*1R*)-*2*-(*4*-chlorophenyl)-*1*-phenylethyl]imidazolidin-2-ylidene-silver-chloride (B39)**

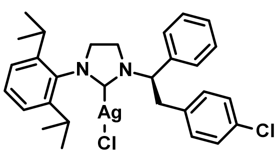

Starting material: 200 mg ***3*-(*2'*,*6'*-diisopropylphenyl)-*1*-[(*1R*)-*2*-(*4*-chlorophenyl)-*1*-phenylethyl]-*4*,*5*-dihydroimidazolium Chloride**[2] (0.42 mmol). Yield: 151 mg (0.26 mmol, 61.8%), white solid. Mp: 226-228°C. ^{1}H NMR (500 MHz, $CDCl_3$) δ 7.51-7.39 (m, Ar-H, 5 H), 7.40 (dm, *J* = 8.5 Hz, Ar-H, 2 H), 7.33 (t, *J* = 7.6 Hz, Ar-H, 1 H), 7.33 (dm, *J* = 8.5 Hz, Ar-H, 2 H), 7.14 (dd, *J* = 7.6, 1.4 Hz, Ar-H, 1 H), 7.13 (dd, *J* = 7.6, 1.4 Hz, Ar-H, 1 H), 5.79 (dd, *J* = 12.0, 4.7 Hz, CH, 1 H), 3.70/3.41 (m+m, CH_2, 2 H), 3.67/3.59 (m+m, CH_2, 2 H), 3.48/3.38 (dd+dd, *J* = 14.2, 4.7 Hz, *J* = 14.2, 12.0 Hz, CH_2, 2 H), 2.75 (sp, *J* = 6.7 Hz, CH, 1 H), 2.20 (sp, *J* = 6.7 Hz, CH, 1 H), 1.25 (d, *J* = 6.7 Hz, CH_3, 3 H), 1.19 (d, *J* = 6.7 Hz, CH_3, 3 H), 1.12 (d, *J* = 6.7 Hz, CH_3, 3 H), 1.10 (d, *J* = 6.7 Hz, CH_3, 3 H). ^{13}C{1H} NMR (125 MHz, $CDCl_3$) δ 205.4, 147.0, 146.4, 137.2, 135.3, 134.5, 133.2, 130.5, 130.0, 129.4, 129.3, 128.9, 127.1, 124.9, 124.5, 64.4, 53.4, 44.3, 36.0, 28.7, 28.3, 25.5, 25.2, 24.6, 23.8. HRMS calcd for ($C_{29}H_{33}AgClN_2$) $[M - Cl]^+$ 551.1383, found 551.1379.

***3*-(*2'*,*6'*-diisopropylphenyl)-*1*-[(*1S*)-*2*-(*4*-chlorophenyl)-*1*-phenylethyl]imidazolidin-2-ylidene-silver-chloride (B40)**

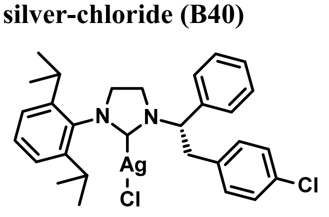

Starting material: 200 mg ***3*-(*2'*,*6'*-diisopropylphenyl)-*1*-[(*1S*)-*2*-(*4*-chlorophenyl)-*1*-phenylethyl] -*4*,*5*-dihydroimidazolium Chloride**[2] (0.42 mmol). Yield: 195 mg (0.33 mmol, 79.8%), white solid. Mp: 213-2155°C. ^{1}H NMR (500 MHz, $CDCl_3$) δ 7.51-7.39 (m, Ar-H, 5 H), 7.40 (dm, *J* = 8.5 Hz, Ar-H, 2 H), 7.33 (t, *J* = 7.6 Hz, Ar-H, 1 H), 7.33 (dm, *J* = 8.5 Hz, Ar-H, 2 H), 7.14 (dd, *J* = 7.6, 1.4 Hz, Ar-H, 1 H), 7.13 (dd,

J = 7.6, 1.4 Hz, Ar-H, 1 H), 5.79 (dd, J = 12.0, 4.7 Hz, CH, 1 H), 3.70/3.41 (m+m, CH_2, 2 H), 3.67/3.59 (m+m, CH_2, 2 H), 3.48/3.38 (dd+dd, J = 14.2, 4.7 Hz, J = 14.2, 12.0 Hz, CH_2, 2 H), 2.75 (sp, J = 6.7 Hz, CH, 1 H), 2.20 (sp, J = 6.7 Hz, CH, 1 H), 1.25 (d, J = 6.7 Hz, CH_3, 3 H), 1.19 (d, J = 6.7 Hz, CH_3, 3 H), 1.12 (d, J = 6.7 Hz, CH_3, 3 H), 1.10 (d, J = 6.7 Hz, CH_3, 3 H). $^{13}C\{1H\}$ NMR (125 MHz, $CDCl_3$) δ 205.4, 147.0, 146.4, 137.2, 135.3, 134.5, 133.2, 130.5, 130.0, 129.4, 129.3, 128.9, 127.1, 124.9, 124.5, 64.4, 53.4, 44.3, 36.0, 28.7, 28.3, 25.5, 25.2, 24.6, 23.8. HRMS calcd for ($C_{29}H_{33}AgClN_2$) $[M - Cl]^+$ 551.1383, found 551.1379.

6. General Procedure for Preparation of Gold(I)-carbene Complexes by Transmetallation (C6, C15-32, C35-40) or Direct Metallation (C5, C41-42)

Transmetallation:

A solution of the corresponding silver NHC complex (**B6**, **B15-32**, **B35-40**) in DCM (10 mL/mmol) and 1 equivalent of dimethylsulfide-gold-chloride reagent were stirred at room temperature until complete conversion was achieved, which usually took 1 hour. The reaction was followed by NMR. The resulting solution was filtered through a pad of celite and the filtrate was evaporated. The resulting oily solid was recrystallized from DCM/DEE.

Direct Metallation:

The corresponding dihydroimidazolium salt[2] was dissolved in deoxygenated acetone (5 mL/mmol) and dimethylsulfide-gold-chloride (1 equivalent) was added. The reaction was stirred at 60°C in the presence of K_2CO_3 (2 equivalents). The reaction was followed by NMR and it took 4-6 hours to achieve complete conversion. The resulting solution was filtered through a pad of celite and the filtrate was evaporated. The remaining oily solid was recrystallized from DCM/DEE.

The **C1-4**, **C7-14**, and **C33-34** gold(I)-carbene complexes were prepared by following published procedures.[1]

(*4S*)-*4-tert*-Butyl-*3*-(*2',6'*-diisopropylphenyl)-*1*-[(*1S*)-*2'*-naphtylethyl]imidazolidin-2-ylidene-gold-chloride (C5)

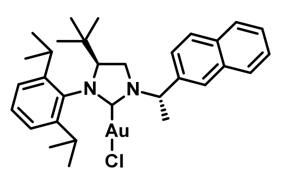

Starting material: 40 mg **(*4S*)-*4-tert*-Butyl-*3*-(*2',6'*-diisopropylphenyl)-*1*-[(*1S*)-*2'*-naphtylethyl]-*4,5*-dihydro-imidazolium Chloride**[1] (0.084 mmol). Yield: 27 mg (0.040 mmol, 47.9%), light yellow solid. Mp: 140-142°C. ^1H NMR (400 MHz, $CDCl_3$) δ 7.95-7.50 (m, Ar-H, 7 H), 7.36 (t, J = 7.7 Hz, Ar-H, 1 H), 7.21 (dd, J = 7.7, 1.5 Hz, Ar-H, 1 H), 7.15 (dd, J = 7.7, 1.5 Hz, Ar-H, 1 H), 6.25 (q, J = 7.0 Hz, CH, 1 H), 3.95 (dd, J = 11.7, 10.6 Hz, CH, 1 H), 3.73/3.06 (t+t, J = 11.7 Hz, J = 10.6 Hz, CH_2, 2 H), 3.46 (sp, J = 6.8 Hz, CH, 1 H), 2.74 (sp, J = 6.8 Hz, CH, 1 H), 1.87 (d, J = 7.0 Hz, CH_3, 3 H), 1.64 (d, J = 6.8 Hz, CH_3, 3 H), 1.36 (d, J = 6.8 Hz, CH_3, 3 H), 1.31 (d, J = 6.8 Hz, CH_3, 3 H), 1.31 (d, J = 6.8 Hz, CH_3, 3 H), 0.61 (s, CH_3, 9 H). $^{13}C\{1H\}$ NMR (100 MHz, $CDCl_3$) δ 196.8, 147.0, 146.0, 136.7, 135.4, 133.3, 133.2, 129.6, 128.9, 128.3, 127.9, 126.8, 126.7, 125.7, 125.5, 125.2, 75.4, 58.2, 45.7, 34.8, 29.2, 28.5, 27.3, 26.4, 26.1, 23.9, 23.7, 16.7. HRMS calcd for ($C_{33}H_{43}AuClN_3$) $[M – Cl + CH_3CN]^+$ 678.3123, found 678.3120.

(*4S*)-*4-tert*-Butyl-*3*-(*2',6'*-diisopropylphenyl)-*1*-[(*1R*)-*2'*-naphtylethyl]imidazolidin-2-ylidene-gold-chloride (C6)

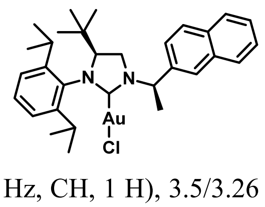

Starting material: 550 mg **B6** (0.94 mmol). Yield: 551 mg (0.82 mmol, 86.9%), white solid. Mp: 218-220°C. ^{1}H NMR (500 MHz, $CDCl_3$) δ 7.93-7.52 (m, Ar-H, 5 H), 7.85 (s, Ar-H, 1 H), 7.66 (dd, J = 8.5, 1.8 Hz, Ar-H, 1 H), 7.35 (t, J = 7.7 Hz, Ar-H, 1 H), 7.21 (dd, J = 7.7, 1.5 Hz, Ar-H, 1 H), 7.14 (dd, J = 7.7, 1.5 Hz, Ar-H, 1 H), 6.31 (q, J = 6.9 Hz, CH, 1 H), 3.78 (dd, J = 11.7, 10.4 Hz, CH, 1 H), 3.5/3.26 (t+t, J = 11.7 Hz, J = 10.4 Hz, CH_2, 2 H), 3.48 (sp, J = 6.8 Hz, CH, 1 H), 2.63 (sp, J = 6.8 Hz, CH, 1 H), 1.85 (d, J = 6.9 Hz, CH_3, 3 H), 1.60 (d, J = 6.8 Hz, CH_3, 3 H), 1.37 (d, J = 6.8 Hz, CH_3, 3 H), 1.33 (d, J = 6.8 Hz, CH_3, 3 H), 1.22 (d, J = 6.8 Hz, CH_3, 3 H), 0.76 (s, CH_3, 9 H). ^{13}C{1H} NMR (125 MHz, $CDCl_3$) δ 196.4, 147.1, 145.9, 135.6, 133.2, 129.5, 128.9, 128.2, 127.9, 126.8, 126.8, 125.7, 125.5, 125.4, 125.2, 75.8, 58.1, 45.4, 34.8, 29.2, 28.4, 27.3, 26.5, 26.1, 23.8, 23.6, 16.1. HRMS calcd for ($C_{31}H_{40}AuClN_2$) $[M]^+$ 672.2546, found 672.2540.

(*4S*)-*4-tert*-Butyl-*3*-(*2',6'*-diisopropylphenyl)-*1*-[(*1R*)-*1*-(*4*-trifluoromethyl)-*2*-phenylethyl]imidazolidin-2-ylidene-gold-chloride (C15)

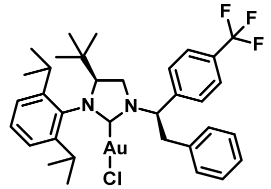

Starting material: 1.22 g **B15** (1.79 mmol). Yield: 998 mg (1.30 mmol, 72.5%), off-white solid. Mp: 261-263°C. ^{1}H NMR (500 MHz, $CDCl_3$) δ 7.74 (d, J = 8.5 Hz, Ar-H, 2 H), 7.70 (d, J = 8.5 Hz, Ar-H, 2 H), 7.46 (d, J = 7.4 Hz, Ar-H, 2 H), 7.40 (t, J = 7.4 Hz, Ar-H, 2 H), 7.33 (t, J = 7.5 Hz, Ar-H, 1 H), 7.30 (t, J = 7.7 Hz, Ar-H, 1 H), 7.10 (dd, J = 7.7, 1.4 Hz, Ar-H, 1 H), 7.07 (dd, J = 7.7, 1.4 Hz, Ar-H, 1 H), 6.47 (dd, J = 12.0, 4.8 Hz, CH, 1 H), 3.85 (t, J = 11.5 Hz, CH, 1 H), 3.58/3.35 (dd+dd, J = 14.3, 4.8 Hz, J = 14.3, 12.0 Hz, CH_2, 2 H), 3.43/3.29 (t+t, J = 11.5 Hz, J = 11.5 Hz, CH_2, 2 H), 2.73 (sp, J = 6.7 Hz, CH, 1 H), 2.54 (sp, J = 6.7 Hz, CH, 1 H), 1.34 (d, J = 6.7 Hz, CH_3, 3 H), 1.26 (d, J = 6.7 Hz, CH_3, 3 H), 1.24 (d, J = 6.7 Hz, CH_3, 3 H), 1.16 (d, J = 6.7 Hz, CH_3, 3 H), 0.61 (s, CH_3, 9 H). ^{13}C{1H} NMR (125 MHz, $CDCl_3$) δ 197.9, 147.0, 145.7, 135.4, 129.8, 129.6, 129.0, 127.7, 127.5, 126.2, 125.4, 125.1, 75.5, 62.2, 45.6, 35.6, 29.2, 27.8, 27.3, 26.0, 23.7, 23.6. HRMS calcd for ($C_{34}H_{41}AuF_3N_2$) $[M - Cl]^+$ 731.2887, found 731.2896.

(*4S*)-*4-tert*-Butyl-*3*-(*2',6'*-diisopropylphenyl)-*1*-[(*1S*)-*1*-(*4*-trifluoromethyl)-*2*-phenylethyl]imidazolidin-2-ylidene-gold-chloride (C16)

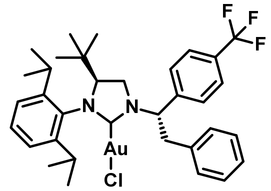

Starting material: 316 mg **B16** (0.466 mmol). Yield: 319 mg (0.416 mmol, 89.2%), light pink solid. Mp: 250-252°C. ^{1}H NMR (500 MHz, $CDCl_3$) δ 7.75 (d, J = 8.5 Hz, Ar-H, 2 H), 7.71 (d, J = 8.5 Hz, Ar-H, 2 H), 7.43 (d, J = 7.5 Hz, Ar-H, 2 H), 7.40 (t, J = 7.5 Hz, Ar-H, 2 H), 7.32 (t, J = 7.5 Hz, Ar-H, 1 H), 7.29 (t, J = 7.7 Hz, Ar-H, 1 H), 7.13 (dd, J = 7.7, 1.4 Hz, Ar-H, 1 H), 7.03 (dd, J = 7.7, 1.4 Hz, Ar-H, 1 H), 6.40 (dd, J = 11.8, 4.9 Hz, CH, 1 H), 3.72 (m, CH, 1 H), 3.72/3.13 (m+m, CH_2, 2 H), 3.56/3.41 (dd+dd, J = 14.4, 4.9 Hz, J = 14.4, 11.8 Hz, CH_2, 2 H), 3.26 (sp, J = 6.7 Hz, CH, 1 H), 1.99 (sp, J = 6.7 Hz, CH, 1 H), 1.53 (d, J = 6.7 Hz, CH_3, 3 H), 1.26 (d, J = 6.7 Hz, CH_3, 3 H), 1.14 (d, J = 6.7 Hz, CH_3, 3 H), 0.92 (d, J = 6.7 Hz, CH_3, 3 H), 0.59 (s, CH_3, 9 H). ^{13}C{1H} NMR (125 MHz, $CDCl_3$) δ 198.0, 146.8, 146.1, 141.4, 136.0, 135.8, 130.9, 129.6, 129.2, 129.1, 127.6, 126.2, 125.3, 125.2, 75.2, 62.5, 46.0, 36.2, 28.8, 28.4, 27.3, 26.4, 23.8, 23.6. HRMS calcd for ($C_{34}H_{44}AuF_3N_3$) $[M – Cl + NH_3]^+$ 748.3153, found 748.3157.

(*4S*)-*4-tert*-Butyl-*3*-(*2',6'*-diisopropylphenyl)-*1*-[(*1R*)-*1*-(*4*-methoxyphenyl)-*2*-phenylethyl]imidazolidin-2-ylidene-gold-chloride (C17)

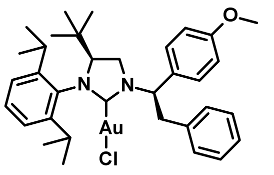

Starting material: 1.11 g **B17** (1.73 mmol). Yield: 1.23 g (1.68 mmol, 97.4%), off-white solid. Mp: 225-227°C. ^{1}H NMR (500 MHz, $CDCl_3$) δ 7.49 (d, J = 8.7 Hz, Ar-H, 2 H), 7.44 (d, J = 7.5 Hz, Ar-H, 2 H), 7.38 (t, J = 7.5 Hz, Ar-H, 2 H), 7.30 (t, J = 7.5 Hz, Ar-H, 1 H), 7.28 (t, J = 7.7 Hz, Ar-H, 1 H), 7.09 (dd, J = 7.7, 1.2 Hz, Ar-H, 1 H), 7.06 (dd, J = 7.7, 1.2 Hz, Ar-H, 1 H), 6.98 (d, J = 8.7 Hz, Ar-H, 2 H), 6.34 (dd, J = 12.0, 4.7 Hz, CH, 1 H), 3.87 (s, CH_3, 3 H), 3.80 (t, J = 11.3 Hz, CH, 1 H), 3.50/3.30 (dd+dd, J = 14.0, 4.7 Hz, J = 14.0, 12.0 Hz, CH_2, 2 H), 3.39/3.32 (t+t, 11.3 Hz, J = 11.3 Hz, CH_2, 2 H), 2.76 (sp, J = 6.7 Hz, CH, 1 H), 2.54 (sp, J = 6.7 Hz, CH, 1 H), 1.33 (d, J = 6.7 Hz, CH_3, 3 H), 1.26 (d, J = 6.7 Hz, CH_3, 3 H), 1.22 (d, J = 6.7 Hz, CH_3, 3 H), 1.16 (d, J = 6.7 Hz, CH_3, 3 H), 0.61 (s, CH_3, 9 H). ^{13}C{1H} NMR (125 MHz, $CDCl_3$) δ 197.0, 159.8, 147.1, 145.8, 136.3, 136.1, 129.5, 129.4, 128.9, 128.6, 127.2, 125.2, 125.0, 114.4, 75.3, 62.3, 55.5, 45.5, 35.9, 29.1, 27.8, 27.3, 26.7, 26.0, 23.7, 23.5. HRMS calcd for ($C_{34}H_{47}AuN_3O$) [M – Cl + NH_3]$^+$ 710.3385, found 710.3376

(*4S*)-*4-tert*-Butyl-*3*-(*2',6'*-diisopropylphenyl)-*1*-[(*1S*)-*1*-(*4*-methoxyphenyl)-*2*-phenylethyl]imidazolidin-2-ylidene-gold-chloride (C18)

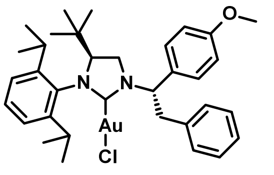

Starting material: 29 mg **B18** (0.05 mmol). Yield: 27 mg (0.04 mmol, 81.7%), purple solid. Mp: 257-259°C. ^{1}H NMR (500 MHz, $CDCl_3$) δ 7.50 (d, J = 8.7 Hz, Ar-H, 2 H), 7.42 (d, J = 7.5 Hz, Ar-H, 2 H), 7.37 (t, J = 7.5 Hz, Ar-H, 2 H), 7.28 (t, J = 7.5 Hz, Ar-H, 1 H), 7.27 (t, J = 7.7 Hz, Ar-H, 1 H), 7.17 (dd, J = 7.7, 1.4 Hz, Ar-H, 1 H), 7.02 (dd, J = 7.7, 1.4 Hz, Ar-H, 1 H), 6.98 (d, J = 8.7 Hz, Ar-H, 2 H), 6.29 (dd, J = 11.7, 5.0 Hz, CH, 1 H), 3.86 (s, CH_3, 3 H), 3.68 (m, CH, 1 H), 3.67/3.15 (m+t, J = 9.1 Hz, CH_2, 2 H), 3.49/3.36 (dd+dd, J = 14.5, 5.0 Hz, J = 14.5, 11.7 Hz, CH_2, 2 H), 3.28 (sp, J = 6.7 Hz, CH, 1 H), 2.02 (sp, J = 6.7 Hz, CH, 1 H), 1.53 (d, J = 6.7 Hz, CH_3, 3 H), 1.25 (d, J = 6.7 Hz, CH_3, 3 H), 1.14 (d, J = 6.7 Hz, CH_3, 3 H), 0.90 (d, J = 6.7 Hz, CH_3, 3 H), 0.58 (s, CH_3, 9 H). ^{13}C{1H} NMR (125 MHz, $CDCl_3$) δ 197.1, 159.7, 146.9, 146.2, 136.6, 136.3, 129.4, 129.3, 129.2, 129.0, 128.4, 127.3, 125.2, 125.1, 114.4, 75.0, 62.5, 55.5, 45.7, 36.4, 28.7, 28.3, 27.3, 26.4. HRMS calcd for ($C_{34}H_{47}AuN_3O$) [M – Cl + NH_3]$^+$ 710.3385, found 710.3383.

(*4S*)-*4-tert*-Butyl-*3*-(*2',6'*-diisopropylphenyl)-*1*-[(*1R*)-*1*-(*p*-tolyl)-*2*-phenylethyl]imidazolidin-2-ylidene-gold-chloride (C19)

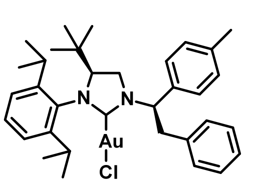

Starting material: 292 mg **B19** (0.468 mmol). Yield: 281 mg (0.394 mmol, 84.2%), purple solid. Mp: 240-242°C. ^{1}H NMR (500 MHz, $CDCl_3$) δ 7.45 (d, J = 7.9 Hz, Ar-H, 2 H), 7.45 (d, J = 7.5 Hz, Ar-H, 2 H), 7.38 (t, J = 7.5 Hz, Ar-H, 2 H), 7.30 (t, J = 7.5 Hz, Ar-H, 1 H), 7.28 (t, J = 7.7 Hz, Ar-H, 1 H), 7.27 (d, J = 7.9 Hz, Ar-H, 2 H), 7.09 (dd, J = 7.7, 1.5 Hz, Ar-H, 1 H), 7.06 (dd, J = 7.7, 1.5 Hz, Ar-H, 1 H), 6.35 (dd, J = 12.0, 4.7 Hz, CH, 1 H), 3.81 (t, J =11.6 Hz, CH, 1 H), 3.52/3.31 (dd+dd, J = 14.3, 4.7 Hz, J = 14.3, 12.0 Hz, CH_2, 2 H), 3.40/3.33 (t+t, J = 11.6 Hz, J = 11.6 Hz, CH_2, 2 H), 2.76 (sp, J = 6.7 Hz, CH, 1 H), 2.56 (sp, J = 6.7 Hz, CH, 1 H), 2.42 (s, CH_3, 3 H), 1.33 (d, J = 6.7 Hz, CH_3, 3 H), 1.26 (d, J = 6.7 Hz, CH_3, 3 H), 1.22 (d, J = 6.7 Hz, CH_3, 3 H), 1.16 (d, J = 6.7 Hz, CH_3, 3 H), 0.60 (s, CH_3, 9 H). ^{13}C{1H} NMR (125 MHz, $CDCl_3$) δ 197.2, 147.1, 145.8, 138.6, 136.3, 136.1, 134.5, 129.8, 129.8, 129.4, 129.4, 128.9, 127.2, 127.2, 125.2, 125.0, 75.4, 62.5, 45.6, 35.8, 29.1, 27.8, 27.3, 26.8, 26.0, 23.7, 23.5, 21.3. HRMS calcd for ($C_{34}H_{44}AuN_2$) [M - Cl]$^+$ 677.3170, found 677.3179.

(*4S*)-*4-tert*-Butyl-*3*-(*2',6'*-diisopropylphenyl)-*1*-[(*1S*)-*1*-(*p*-tolyl)-*2*-phenylethyl]imidazolidin-2-ylidene-gold-chloride (C20)

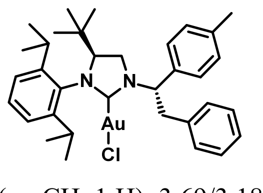

Starting material: 117 mg **B20** (0.188 mmol). Yield: 99 mg (0.139 mmol, 74.0%), light purple solid. Mp: 226-228°C. ^{1}H NMR (500 MHz, $CDCl_3$) δ 7.48 (d, J = 8.0 Hz, Ar-H, 2 H), 7.47-7.27 (m, Ar-H, 5 H), 7.29 (t, J = 7.7 Hz, Ar-H, 1 H), 7.28 (d, J = 8.0 Hz, Ar-H, 2 H), 7.14 (dd, J = 7.7, 1.4 Hz, Ar-H, 1 H), 7.04 (dd, J = 7.7, 1.4 Hz, Ar-H, 1 H), 6.33 (dd, J = 11.8, 5.0 Hz, CH, 1 H), 3.71 (m, CH, 1 H), 3.69/3.18 (m+m, CH_2, 2 H), 3.53/3.39 (dd+dd, J = 14.5, 5.0 Hz, J = 14.5, 11.8 Hz, CH_2, 2 H), 3.31 (sp, J = 6.7 Hz, CH, 1 H), 2.42 (s, CH_3, 3 H), 2.04 (sp, J = 6.7 Hz, CH, 1 H), 1.55 (d, J = 6.7 Hz, CH_3, 3 H), 1.27 (d, J = 6.7 Hz, CH_3, 3 H), 1.16 (d, J = 6.7 Hz, CH_3, 3 H), 0.93 (d, J = 6.7 Hz, CH_3, 3 H), 0.60 (s, CH_3, 9 H). ^{13}C{1H} NMR (125 MHz, $CDCl_3$) δ 197.3, 146.9, 146.2, 138.4, 136.6, 136.3, 134.3, 129.8, 129.4, 129.2, 129.0, 127.3, 127.1, 125.2, 125.1, 75.0, 62.8, 45.8, 36.3, 28.7, 28.3, 27.3, 26.4, 23.8, 23.6, 21.3. HRMS calcd for ($C_{34}H_{44}AuClN_2$) $[M]^+$ 712.2859, found 712.2844.

(*4S*)-*4*-*tert*-Butyl-*3*-(*2'*,*6'*-diisopropylphenyl)-*1*-[(*1R*)-*1*-(*2*-methoxyphenyl)-*2*-phenylethyl]imidazolidin-2-ylidene-gold-chloride (C21)

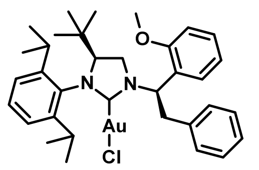

Starting material: 333 mg **B21** (0.520 mmol). Yield: 318 mg (0.436 mmol, 83.8%), light purple solid. Mp: 231-233°C. ^{1}H NMR (500 MHz, $CDCl_3$) δ 7.52 (dd, J = 7.6, 1.3 Hz, Ar-H, 1 H), 7.45 (d, J = 7.3 Hz, Ar-H, 2 H), 7.42 (td, J = 7.6, 1.3 Hz, Ar-H, 1 H), 7.37 (t, J = 7.3 Hz, Ar-H, 2 H), 7.30 (t, J = 7.3 Hz, Ar-H, 1 H), 7.25 (t, J = 7.7 Hz, Ar-H, 1 H), 7.06 (dd, J = 7.7, 1.3 Hz, Ar-H, 1 H), 7.06 (td, J = 7.6, 0.8 Hz, Ar-H, 1 H), 7.02 (dd, J = 7.7, 1.3 Hz, Ar-H, 1 H), 6.99 (dd, J = 7.6, 0.8 Hz, Ar-H, 1 H), 6.44 (dd, J = 12.0, 4.3 Hz, CH, 1 H), 3.88 (s, CH_3, 3 H), 3.77 (t, J = 11.7 Hz, CH, 1 H), 3.51/3.27 (dd+t, J = 11.7, 11.2 Hz, J = 11.2 Hz, CH_2, 2 H), 3.48/3.31 (dd+dd, J = 14.0, 12.0 Hz, J = 14.0, 4.3 Hz, CH_2, 2 H), 2.73 (sp, J = 6.7 Hz, CH, 1 H), 2.53 (sp, J = 6.7 Hz, CH, 1 H), 1.26 (d, J = 6.7 Hz, CH_3, 3 H), 1.20 (d, J = 6.7 Hz, CH_3, 3 H), 1.20 (d, J = 6.7 Hz, CH_3, 3 H), 1.16 (d, J = 6.7 Hz, CH_3, 3 H), 0.66 (s, CH_3, 9 H). ^{13}C{1H} NMR (125 MHz, $CDCl_3$) δ 197.9, 158.5, 147.2, 145.7, 136.7, 136.5, 130.1, 130.0, 129.2, 128.8, 127.7, 127.2, 125.7, 125.2, 124.9, 120.2, 111.2, 74.8, 58.5, 55.5, 46.3, 36.3, 28.8, 27.7, 27.3, 26.9, 26.1, 23.8, 23.6. HRMS calcd for ($C_{34}H_{47}AuN_3O$) $[M - Cl + NH_3]^+$ 710.3385, found 710.3384.

(*4S*)-*4*-*tert*-Butyl-*3*-(*2'*,*6'*-diisopropylphenyl)-*1*-[(*1S*)-*1*-(*2*-methoxyphenyl)-*2*-phenylethyl]imidazolidin-2-ylidene-gold-chloride (C22)

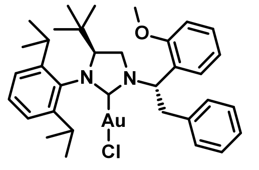

Starting material: 362 mg **B22** (0.566 mmol). Yield: 269 mg (0.369 mmol, 65.2%), off-white solid. Mp: 243-245°C. ^{1}H NMR (500 MHz, $CDCl_3$) δ 7.56 (dd, J = 7.6, 1.3 Hz, Ar-H, 1 H), 7.44 (d, J = 7.5 Hz, Ar-H, 2 H), 7.37 (td, J = 8.2, 1.3 Hz, Ar-H, 1 H), 7.36 (t, J = 7.5 Hz, Ar-H, 2 H), 7.26 (t, J = 7.7 Hz, Ar-H, 1 H), 7.26 (t, J = 7.5 Hz, Ar-H, 1 H), 7.10 (dd, J = 7.7, 1.5 Hz, Ar-H, 1 H), 7.04 (td, J = 7.6, 0.8 Hz, Ar-H, 1 H), 7.02 (dd, J = 7.7, 1.5 Hz, Ar-H, 1 H), 6.95 (dd, J = 8.2, 0.8 Hz, Ar-H, 1 H), 6.20 (dd, J = 11.7, 4.8 Hz, CH, 1 H), 3.90 (s, CH_3, 3 H), 3.72/3.02 (m+m, CH_2, 2 H), 3.72 (m, CH, 1 H), 3.63/3.39 (dd+dd, J = 14.2, 11.7 Hz, J = 14.2, 4.8 Hz, CH_2, 2 H), 3.32 (sp, J = 6.7 Hz, CH, 1 H), 2.24 (sp, J = 6.7 Hz, CH, 1 H), 1.55 (d, J = 6.7 Hz, CH_3, 3H), 1.24 (d, J = 6.7 Hz, CH_3, 3H), 1.20 (d, J = 6.7 Hz, CH_3, 3 H), 0.93 (d, J = 6.7 Hz, CH_3, 3 H), 0.53 (s, CH_3, 9 H). ^{13}C{1H} NMR (125 MHz, $CDCl_3$) δ 198.0, 157.9, 146.7, 146.1, 137.2, 136.8, 130.0, 129.5, 129.2, 129.0, 127.9, 127.2, 125.9, 125.1, 120.5, 110.8, 74.5, 58.9, 55.0, 47.9, 36.9, 28.8, 28.1, 27.2, 26.3, 23.8, 23.6. HRMS calcd for ($C_{34}H_{47}AuN_3O$) $[M - Cl + NH_3]^+$ 710.3385, found 710.3376.

(*4S*)-*4*-*tert*-Butyl-*3*-(*2'*,*6'*-diisopropylphenyl)-*1*-[(*1R*)-*1*-(*o*-tolyl)-*2*-phenylethyl]imidazolidin-2-ylidene-gold-chloride (C23)

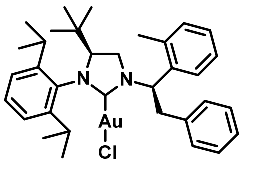

Starting material: 84 mg **B23** (0.138 mmol). Yield: 63 mg (0.088 mmol, 64.1%), light purple solid. Mp: 252-254°C. ^{1}H NMR (500 MHz, $CDCl_3$) δ 7.62-7.30 (m, Ar-H, 4 H), 7.45-7.31 (m, Ar-H, 5 H), 7.27 (t, J = 7.7 Hz, Ar-H, 1 H), 7.09 (dd, J = 7.7, 1.2 Hz, Ar-H, 1 H), 7.04 (dd, J = 7.7, 1.2 Hz, Ar-H, 1 H), 6.24 (dd, J = 11.5, 4.0 Hz, CH, 1 H), 3.80 (t, J = 11.8 Hz, CH, 1 H), 3.54/3.20 (dd+t, J = 11.8, 11.4 Hz, J = 11.4 Hz, CH_2, 2 H), 3.51/3.36 (dd+dd, J = 13.7, 11.5 Hz, J = 13.7, 4.0 Hz, CH_2, 2 H), 2.79 (sp, J = 6.7 Hz, CH, 1 H), 2.54 (s, CH_3, 3 H), 2.52 (sp, J = 6.7 Hz, CH, 1 H), 1.22 (d, J = 6.7 Hz, CH_3, 3 H), 1.22 (d, J = 6.7 Hz, CH_3, 3 H), 1.22 (d, J = 6.7 Hz, CH_3, 3 H), 1.18 (d, J = 6.7 Hz, CH_3, 3 H), 0.73 (s, CH_3, 9 H). ^{13}C{1H} NMR (125 MHz, $CDCl_3$) δ 197.9, 147.1, 145.6, 139.1, 136.5, 136.2, 135.2, 131.8, 130.2, 129.4, 129.1, 128.8, 127.5, 126.8, 126.2, 125.3, 125.0, 75.2, 61.6, 45.9, 36.9, 29.0, 27.6, 27.5, 26.9, 26.0, 23.7, 20.7. HRMS calcd for ($C_{34}H_{47}AuN_3$) $[M – Cl + NH_3]^+$ 694.3436, found 694.3428.

(*4S*)-*4-tert*-Butyl-*3*-(*2',6'*-diisopropylphenyl)-*1*-[(*1S*)-*1*-(*o*-tolyl)-*2*-phenylethyl]imidazolidin-2-ylidene-gold-chloride (C24)

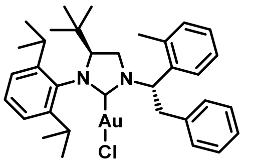

Starting material: 30 mg **B24** (0.048 mmol). Yield: 24 mg (0.034 mmol, 70.0%), off-white solid. Mp: 253-255°C. ^{1}H NMR (500 MHz, $CDCl_3$) δ 7.59-7.24 (m, Ar-H, 4 H), 7.45-7.22 (m, Ar-H, 5 H), 7.29 (t, J = 7.7 Hz, Ar-H, 1 H), 7.12 (dd, J = 7.7, 1.4 Hz, Ar-H, 1 H), 7.06 (dd, J = 7.7, 1.4 Hz, Ar-H, 1 H), 6.27 (dd, J = 10.1, 5.8 Hz, CH, 1 H), 3.83 (dd, J = 11.8, 10.1 Hz, CH, 1 H), 3.75/3.06 (t+t, J = 11.8 Hz, J = 10.1 Hz, CH_2, 2 H), 3.53/3.43 (dd+dd, J = 14.1, 10.1 Hz, J = 14.1, 5.8 Hz, CH_2, 2 H), 3.26 (sp, J = 6.7 Hz, CH, 1 H), 2.58 (s, CH_3, 3 H), 2.34 (sp, J = 6.7 Hz, CH, 1 H), 1.51 (d, J = 6.7 Hz, CH_3, 3 H), 1.26 (d, J = 6.7 Hz, CH_3, 3 H), 1.24 (d, J = 6.7 Hz, CH_3, 3 H), 1.02 (d, J = 6.7 Hz, CH_3, 3 H), 0.58 (s, CH_3, 9 H). ^{13}C{1H} NMR (125 MHz, $CDCl_3$) δ 197.5, 146.8, 146.1, 138.2, 136.7, 136.2, 135.0, 131.8, 129.5, 129.4, 129.1, 128.8, 127.3, 126.7, 126.2, 125.2, 125.1, 74.5, 61.1, 46.8, 37.6, 29.0, 28.4, 27.1, 26.5, 26.4, 23.9, 23.6, 21.1. HRMS calcd for ($C_{34}H_{47}AuN_3$) $[M – Cl + NH_3]^+$ 694.3436, found 694.3442.

(*4S*)-*4-tert*-Butyl-*3*-(*2',6'*-diisopropylphenyl)-*1*-[(*1R*)-*1*-(*3*-methoxyphenyl)-*2*-phenylethyl]imidazolidin-2-ylidene-gold-chloride (C25)

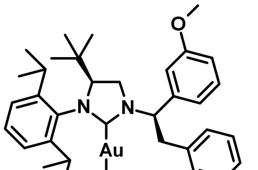

Starting material: 100 mg **B25** (0.16 mmol). Yield: 77 mg (0.68 mmol, 67.6%), off-white solid. Mp: 207-209°C.^{1}H NMR (400 MHz, $CDCl_3$) δ 7.50-7.28 (m, Ar-H, 5 H), 7.39 (t, J = 8.3 Hz, Ar-H, 1 H), 7.29 (t, J = 7.7 Hz, Ar-H, 1 H), 7.16 (dd, J = 8.3, 2.3 Hz, Ar-H, 1 H), 7.11 (t, J = 2.0 Hz, Ar-H,1 H), 7.10 (dd, J = 7.7, 1.5 Hz, Ar-H, 1 H), 7.07 (dd, J = 7.7, 1.5 Hz, Ar-H, 1 H), 6.96 (dd, J = 8.3, 2.3 Hz, Ar-H, 1 H), 6.35 (dd, J = 12.3, 4.7 Hz, CH, 1 H), 3.87 (s, CH_3, 3 H), 3.84 (t, J = 11.2 Hz, CH, 1 H), 3.56/3.32 (dd+dd, J = 14.4, 4.7 Hz, J = 14.4, 12.3 Hz, CH_2, 2 H), 3.42/3.35 (t+t, J = 11.2 Hz, CH_2, 2 H), 2.75 (sp, J = 6.7 Hz, CH, 1 H), 2.60 (sp, J = 6.7 Hz, CH, 1 H), 1.33 (d, J = 6.7 Hz, CH_3, 3 H), 1.26 (d, J = 6.7 Hz, CH_3, 3 H), 1.24 (d, J = 6.7 Hz, CH_3, 3 H), 1.17 (d, J = 6.7 Hz, CH_3, 3 H), 0.62 (s, CH_3, 9 H). ^{13}C{1H} NMR (100 MHz, $CDCl_3$) δ 197.4, 160.2, 147.1, 145.8, 139.2, 136.0, 130.1, 129.8, 129.5, 128.9, 127.3, 125.3, 125.0, 119.3, 114.1, 113.1, 75.4, 62.7, 55.5, 45.7, 35.8, 34.3, 29.2, 27.8, 27.3, 26.8, 26.0, 23.7, 23.5. HRMS calcd for ($C_{36}H_{47}AuN_3O$) $[M – Cl + CH_3CN]^+$ 734.3385, found 734.3375.

(*4S*)-*4-tert*-Butyl-*3*-(*2',6'*-diisopropylphenyl)-*1*-[(*1S*)-*1*-(*3*-methoxyphenyl)-*2*-phenylethyl]imidazolidin-2-ylidene-gold-chloride (C26)

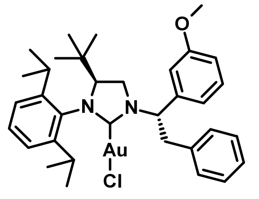

Starting material: 70 mg **B26** (0.11 mmol). Yield: 70 mg (0.096 mmol, 87.8%), off-white solid. Mp: 270-272°C. ^{1}H NMR (400 MHz, $CDCl_3$) δ 7.45 (t, J = 7.6 Hz, Ar-H, 2 H), 7.43 (d, J = 7.6 Hz, Ar-H, 2 H), 7.42 (t, J = 8.2 Hz, Ar-H, 1 H), 7.33 (t, J = 7.6 Hz, Ar-H, 1 H), 7.31 (t, J = 7.7 Hz, Ar-H, 1 H), 7.19 (t, J = 2.4 Hz, Ar-H, 1 H), 7.18 (dd, J = 8.2, 2.3 Hz, Ar-H, 1 H), 7.16 (dd, J = 7.7, 1.5 Hz, Ar-H, 1 H), 7.06 (dd, J = 7.7, 1.5 Hz, Ar-H, 1 H), 6.97 (dd, J = 8.2, 2.3 Hz, Ar-H, 1 H), 6.33 (dd, J = 11.9, 4.9 Hz, CH, 1 H), 3.89 (s, CH_3, 3 H), 3.74 (dd, J = 11.6, 9.6 Hz, CH, 1 H), 3.69/3.19 (dd+t, J = 11.6, 9.6 Hz, J = 9.6 Hz, CH_2, 2 H), 3.58/3.39 (dd+dd, J = 14.6, 4.9 Hz, J = 14.6, 11.9 Hz, CH_2, 2 H), 3.34 (sp, J = 6.7 Hz, CH, 1 H), 2.05 (sp, J = 6.7 Hz, CH, 1 H), 1.56 (d, J = 6.7 Hz, CH_3, 3 H), 1.28 (d, J = 6.7 Hz, CH_3, 3 H), 1.17 (d, J = 6.7 Hz, CH_3, 3 H), 0.95 (d, J = 6.7 Hz, CH_3, 3 H), 0.62 (s, CH_3, 9 H). ^{13}C{1H} NMR (100 MHz, $CDCl_3$) δ 197.6, 160.1, 146.8, 146.2, 139.0, 136.5, 136.2, 130.1, 129.4, 129.2, 129.0, 127.3, 125.2, 125.1, 119.1, 114.6, 112.4, 75.0, 62.8, 55.4, 45.9, 36.0, 34.4, 28.7, 28.4, 27.2, 26.5, 26.4, 23.7, 23.6. HRMS calcd for ($C_{36}H_{47}AuN_3O$) [M – Cl + CH_3CN]$^+$ 734.3385, found 734.3380.

(*4S*)-*4*-*tert*-Butyl-*3*-(*2',6'*-diisopropylphenyl)-*1*-[(*1R*)-*2*-(*p*-tolyl)-*1*-phenylethyl]imidazolidin-2-ylidene-gold-chloride (C27)

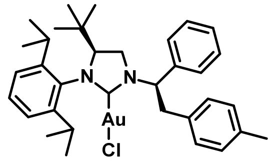

Starting material: 45 mg **B27** (0.072 mmol). Yield: 36 mg (0.051 mmol, 70.0%), light purple solid. Mp: 257-259°C. ^{1}H NMR (500 MHz, $CDCl_3$) δ 7.57 (d, J = 7.5 Hz, Ar-H, 2 H), 7.47 (t, J = 7.5 Hz, Ar-H, 2 H), 7.41 (t, J = 7.5 Hz, Ar-H, 1 H), 7.35 (d, J = 7.7 Hz, Ar-H, 2 H), 7.28 (t, J = 7.7 Hz, Ar-H, 1 H), 7.19 (d, J = 7.7 Hz, Ar-H, 2 H), 7.09 (d, J = 7.7 Hz, Ar-H, 1 H), 7.06 (d, J = 7.7 Hz, Ar-H, 1 H), 6.37 (dd, J = 12.0, 4.6 Hz, CH, 1 H), 3.82 (t, J = 11.6 Hz, CH, 1 H), 3.51/3.29 (dd+dd, J = 14.4, 4.6 Hz, J = 14.4, 12.0 Hz, CH_2, 2 H), 3.41/3.31 (t+t, J = 11.6 Hz, J = 11.6 Hz, CH_2, 2 H), 2.76 (sp, J = 6.7 Hz, CH, 1 H), 2.57 (sp, J = 6.7 Hz, CH, 1 H), 2.37 (s, CH_3, 3 H), 1.35 (d, J = 6.7 Hz, CH_3, 3 H), 1.26 (d, J = 6.7 Hz, CH_3, 3 H), 1.22 (d, J = 6.7 Hz, CH_3, 3 H), 1.16 (d, J = 6.7 Hz, CH_3, 3 H), 0.60 (s, CH_3, 9 H). ^{13}C{1H} NMR (125 MHz, $CDCl_3$) δ 197.3, 147.1, 145.8, 137.7, 136.8, 136.2, 132.8, 129.7, 129.5, 129.4, 129.1, 128.7, 127.3, 125.2, 125.0, 75.3, 62.7, 45.6, 35.3, 29.1, 27.9, 27.1, 26.8, 26.0, 23.7, 23.6, 21.3. HRMS calcd for ($C_{34}H_{44}AuClN_2$) [M]$^+$ 712.2859, found 712.2800.

(*4S*)-*4*-*tert*-Butyl-*3*-(*2',6'*-diisopropylphenyl)-*1*-[(*1S*)-*2*-(*p*-tolyl)-*1*-phenylethyl]imidazolidin-2-ylidene-gold-chloride (C28)

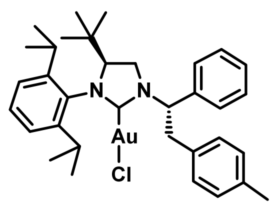

Starting material: 100 mg **B28** (0.160 mmol). Yield: 87 mg (0.122 mmol, 76.1%), light purple solid. Mp: 216-218°C. ^{1}H NMR (500 MHz, $CDCl_3$) δ 7.57 (d, J = 7.5 Hz, Ar-H, 2 H), 7.46 (t, J = 7.5 Hz, Ar-H, 2 H), 7.39 (t, J = 7.5 Hz, Ar-H, 1 H), 7.31 (d, J = 7.8 Hz, Ar-H, 2 H), 7.27 (t, J = 7.7 Hz, Ar-H, 1 H), 7.18 (d, J = 7.8 Hz, Ar-H, 2 H), 7.12 (dd, J = 7.7, 1.2 Hz, Ar-H, 1 H), 7.02 (dd, J = 7.7, 1.2 Hz, Ar-H, 1 H), 6.32 (dd, J = 11.9, 4.9 Hz, CH, 1 H), 3.69 (m, CH, 1 H), 3.69/3.14 (m+m, CH_2, 2 H), 3.49/3.34 (dd+dd, J = 14.4, 4.9 Hz, J = 14.4, 11.9 Hz, CH_2, 2 H), 3.29 (sp, J = 6.7 Hz, CH, 1 H), 2.35 (s, CH_3, 3 H), 2.03 (sp, J = 6.7 Hz, CH, 1 H), 1.53 (d, J = 6.7 Hz, CH_3, 3 H), 1.25 (d, J = 6.7 Hz, CH_3, 3 H), 1.14 (d, J = 6.7 Hz, CH_3, 3 H), 0.89 (d, J = 6.7 Hz, CH_3, 3 H), 0.57 (s, CH_3, 9 H). ^{13}C{1H} NMR (125 MHz, $CDCl_3$) δ 197.4, 146.9, 146.2, 137.4, 136.7, 136.3, 133.3, 129.8, 129.4, 129.1, 129.0, 128.6, 127.2, 125.1, 75.0, 63.0, 45.8, 35.7, 28.8, 28.3, 27.3, 26.4, 26.1, 23.8, 23.6, 21.3. HRMS calcd for ($C_{34}H_{44}AuClN_2$) [M]$^+$ 712.2859, found 712.2781.

(*4S*)-*4*-*tert*-Butyl-*3*-(*2'*,*6'*-diisopropylphenyl)-*1*-[(*1R*)-*2*-(*4*-chlorophenyl)-*1*-phenylethyl]imidazolidin-2-ylidene-gold-chloride (C29)

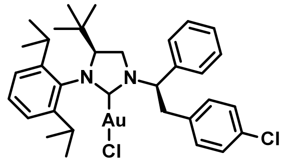

Starting material: 175 mg **B29** (0.272 mmol). Yield: 175 mg (0.233 mmol, 85.9%), light purple solid. Mp: 262-264°C. ^{1}H NMR (500 MHz, $CDCl_3$) δ 7.60-7.41 (m, Ar-H, 5 H), 7.44 (m, Ar-H, 2 H), 7.39 (m, Ar-H, 2 H), 7.31 (t, J = 7.7 Hz, Ar-H, 1 H), 7.13 (dd, J = 7.7, 1.4 Hz, Ar-H, 1 H), 7.09 (dd, J = 7.7, 1.4 Hz, Ar-H, 1 H), 6.42 (dd, J = 12.0, 4.8 Hz, CH, 1 H), 3.86 (t, J = 11.5 Hz, CH, 1 H), 3.54/3.34 (dd+dd, J = 14.3, 4.8 Hz, J = 14.3, 12.0 Hz, CH_2, 2 H), 3.38/3.33 (t+t, J = 11.5 Hz, J = 11.5 Hz, CH_2, 2 H), 2.73 (sp, J = 6.7 Hz, CH, 1 H), 2.58 (sp, J = 6.7 Hz, CH, 1 H), 1.42 (d, J = 6.7 Hz, CH_3, 3 H), 1.29 (d, J = 6.7 Hz, CH_3, 3 H), 1.25 (d, J = 6.7 Hz, CH_3, 3 H), 1.23 (d, J = 6.7 Hz, CH_3, 3 H), 0.64 (s, CH_3, 9 H). ^{13}C{1H} NMR (125 MHz, $CDCl_3$) δ 197.4, 147.1, 145.7, 137.2, 136.0, 134.4, 133.2, 131.3, 129.6, 129.2, 129.0, 128.9, 127.2, 125.3, 125.0, 75.4, 62.6, 45.6, 35.1, 29.2, 28.1, 27.0, 26.8, 26.0, 23.7, 23.5. HRMS calcd for ($C_{33}H_{41}AuCl_2N_2$) $[M]^+$ 732.2312, found 732.2237.

(*4S*)-*4*-*tert*-Butyl-*3*-(*2'*,*6'*-diisopropylphenyl)-*1*-[(*1S*)-*2*-(*4*-chlorophenyl)-*1*-phenylethyl]imidazolidin-2-ylidene-gold-chloride (C30)

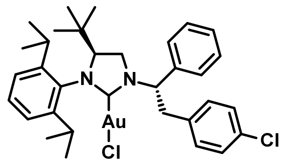

Starting material: 174 mg **B30** (0.270 mmol). Yield: 114 mg (0.155 mmol, 57.6%), light pink solid. Mp: 212-214°C. ^{1}H NMR (500 MHz, $CDCl_3$) δ 7.62-7.39 (m, Ar-H, 5 H), 7.41 (m, Ar-H, 2 H), 7.38 (m, Ar-H, 2 H), 7.31 (t, J = 7.7 Hz, Ar-H, 1 H), 7.15 (dm, J = 7.7 Hz, Ar-H, 1 H), 7.07 (dm, J = 7.7 Hz, Ar-H, 1 H), 6.36 (dd, J = 12.0, 4.9 Hz, CH, 1 H), 3.73 (t, J = 11.0 Hz, CH, 1 H), 3.64/3.16 (t+t, J = 11.0 Hz, J = 11.0 Hz, CH_2, 2 H), 3.53/3.40 (dd+dd, J = 14.5, 4.9 Hz, J = 14.5, 12.0 Hz, CH_2, 2 H), 3.30 (sp, J = 6.7 Hz, CH, 1 H), 2.04 (sp, J = 6.7 Hz, CH, 1 H), 1.55 (d, J = 6.7 Hz, CH_3, 3 H), 1.27 (d, J = 6.7 Hz, CH_3, 3 H), 1.20 (d, J = 6.7 Hz, CH_3, 3 H), 1.00 (d, J = 6.7 Hz, CH_3, 3 H), 0.60 (s, CH_3, 9 H). ^{13}C{1H} NMR (125 MHz, $CDCl_3$) δ 197.5, 146.8, 146.1, 136.9, 136.0, 134.9, 133.2, 130.6, 129.5, 129.2, 128.8, 127.1, 125.2, 125.2, 75.1, 62.8, 45.8, 35.5, 28.9, 28.4, 27.3, 26.4, 26.1, 25.1, 23.8, 23.6. HRMS calcd for ($C_{33}H_{41}AuCl_2N_2$) $[M]^+$ 732.2312, found 732.2240.

(*4S*)-*4*-*tert*-Butyl-*3*-(*2'*,*6'*-diisopropylphenyl)-*1*-[(*1R*)-*2*-(*2*-chlorophenyl)-*1*-phenylethyl]imidazolidin-2-ylidene-gold-chloride (C31)

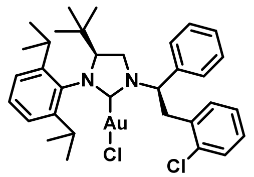

Starting material: 150 mg **B31** (0.233 mmol). Yield: 139 mg (0.190 mmol, 81.4%), off-white solid. Mp: 211-213°C. ^{1}H NMR (500 MHz, $CDCl_3$) δ 7.64-7.23 (m, Ar-H, 4 H), 7.63-7.41 (m, Ar- H, 5 H), 7.30 (t, J = 7.7 Hz, Ar-H, 1 H), 7.11 (dd, J = 7.7, 1.4 Hz, Ar-H, 1 H), 7.08 (dd, J = 7.7, 1.4 Hz, Ar-H, 1 H), 6.45 (dd, J = 11.8, 4.4 Hz, CH, 1 H), 3.83 (t, J = 11.6 Hz, CH, 1 H), 3.71/3.53 (dd+dd, J = 14.7, 11.8 Hz, J = 14.7, 4.4 Hz, CH_2, 2 H), 3.47/3.34 (t+t, J = 11.6 Hz, J = 11.6 Hz, CH_2, 2 H), 2.86 (sp, J = 6.7 Hz, CH, 1 H), 2.56 (sp, J = 6.7 Hz, CH, 1 H), 1.34 (d, J = 6.7 Hz, CH_3, 3 H), 1.28 (d, J = 6.7 Hz, CH_3, 3 H), 1.24 (d, J = 6.7 Hz, CH_3, 3 H), 1.19 (d, J = 6.7 Hz, CH_3, 3 H), 0.65 (s, CH_3, 9 H). ^{13}C{1H} NMR (125 MHz, $CDCl_3$) δ 197.4, 147.0, 145.8, 137.4, 136.3, 134.6, 134.0, 131.6, 130.3, 129.5, 129.2, 128.8, 128.7, 127.4, 127.3, 125.2, 125.1, 75.6, 66.0, 61.6, 45.9, 32.5, 29.1, 27.9, 27.2, 26.7, 26.0, 23.7, 23.6. HRMS calcd for ($C_{33}H_{41}AuCl_2N_2$) $[M]^+$ 732.2312, found 732.2286.

(*4S*)-*4*-*tert*-Butyl-*3*-(*2'*,*6'*-diisopropylphenyl)-*1*-[(*1S*)-*2*-(*2*-chlorophenyl)-*1*-phenylethyl]imidazolidin-2-ylidene-gold-chloride (C32)

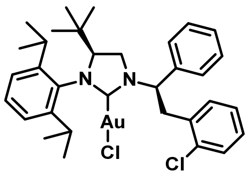

Starting material: 150 mg **B32** (0.233 mmol). Yield: 129 mg (0.176 mmol, 75.6%), light purple solid. Mp: 242-244°C. ^{1}H NMR (500 MHz, $CDCl_3$) δ 7.61-7.39 (m, Ar-H, 5 H), 7.59-7.39 (m, Ar-H, 2 H), 7.33 (dt, J = 7.7, 1.5 Hz, Ar-H, 1 H), 7.28 (t, J = 7.7 Hz, Ar-H, 1 H), 7.24 (dt, J = 7.7, 1.5 Hz, Ar-H, 1 H), 7.13 (dd, J = 7.7, 1.5 Hz, Ar-H, 1 H), 7.04 (dd, J = 7.7, 1.5 Hz, Ar-H, 1 H), 6.37 (dd, J = 12.0, 4.0 Hz, CH, 1 H), 3.88/3.08 (dd+t, J = 11.9, 10.1 Hz, J = 10.1 Hz, CH_2, 2 H), 3.83 (t, J = 11.9 Hz, CH, 1 H), 3.83/3.45 (dd+dd, J = 14.3, 12.0 Hz, J = 14.3, 4.0 Hz, CH_2, 2 H), 3.28 (sp, J = 6.7 Hz, CH, 1 H), 2.26 (sp, J = 6.7 Hz, CH, 1 H), 1.53 (d, J = 6.7 Hz, CH_3, 3 H), 1.26 (d, J = 6.7 Hz, CH_3, 3 H), 1.23 (d, J = 6.7 Hz, CH_3, 3 H), 0.93 (d, J = 6.7 Hz, CH_3, 3 H), 0.58 (s, CH_3, 9 H). ^{13}C{1H} NMR (125 MHz, $CDCl_3$) δ 197.2, 146.8, 146.2, 137.2, 136.1, 134.5, 134.2, 131.6, 130.0, 129.4, 129.2, 128.8, 128.8, 128.0, 127.2, 125.2, 75.0, 63.0, 46.3, 32.9, 28.8, 28.3, 27.3, 26.5, 23.8, 23.6. HRMS calcd for ($C_{33}H_{41}AuCl_2N_2$) $[M]^+$ 732.2312, found 732.2300.

(*4R*)-*4*-*tert*-Butyl-*3*-(*2’,6’*-diisopropylphenyl)-*1*-[(*1R*)-*2*-(*3*-chlorophenyl)-*1*-phenylethyl]imidazolidin-2-ylidene-gold-chloride (C35)

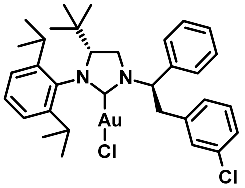

Starting material: 200 mg **B35** (0.310 mmol). Yield: 189 mg (0.258 mmol, 83.0%), light purple solid. Mp: 219-221°C. ^{1}H NMR (500 MHz, $CDCl_3$) δ 7.57-7.26 (m, Ar-H, 9 H), 7.28 (t, J = 7.7 Hz, Ar-H, 1 H), 7.13 (dd, J = 7.7, 1.5 Hz, Ar-H, 1 H), 7.04 (dd, J = 7.7, 1.5 Hz, Ar-H, 1 H), 6.30 (dd, J = 11.8, 4.8 Hz, CH, 1 H), 3.76 (dd, J = 11.6, 10.8 Hz, CH, 1 H), 3.66/3.13 (dd+t, J = 11.6, 10.8 Hz, J = 10.8 Hz, CH_2, 2 H), 3.51/3.39 (dd+dd, J = 14.4, 4.8 Hz, J = 14.4, 11.8 Hz, CH_2, 2 H), 3.28 (sp, J = 6.7 Hz, CH, 1 H), 2.08 (sp, J = 6.7 Hz, CH, 1 H), 1.54 (d, J = 6.7 Hz, CH_3, 3 H), 1.25 (d, J = 6.7 Hz, CH_3, 3 H), 1.21 (d, J = 6.7 Hz, CH_3, 3 H), 0.93 (d, J = 6.7 Hz, CH_3, 3 H), 0.58 (s, CH_3, 9 H). ^{13}C{1H} NMR (125 MHz, $CDCl_3$) δ 197.4, 146.8, 146.1, 138.6, 136.9, 136.1, 134.4, 130.7, 129.5, 129.2, 128.9, 127.6, 127.1, 125.2, 75.0, 63.0, 46.0, 35.8, 34.5, 29.0, 28.4, 27.3, 26.4, 26.2, 23.8, 23.6. HRMS calcd for ($C_{33}H_{44}AuClN_3$) $[M - Cl + NH_3]^+$ 714.2889, found 714.2895. Anal. Calcd. for $C_{33}H_{41}AuCl_2N_2$: C, 54.03; H, 5.36; N, 3.82. Found: C, 54.37; H, 5.65; N, 3.63.

(*4R*)-*4*-*tert*-Butyl-*3*-(*2’,6’*-diisopropylphenyl)-*1*-[(*1S*)-*2*-(*3*-chlorophenyl)-*1*-phenylethyl]imidazolidin-2-ylidene-gold-chloride (C36)

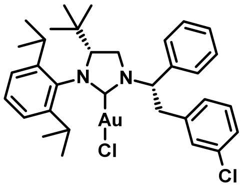

Starting material: 250 mg **B36** (0.388 mmol). Yield: 250 mg (0.341 mmol, 87.9%), off-white solid. Mp: 256-258°C. ^{1}H NMR (500 MHz, $CDCl_3$) δ 7.57-7.29 (m, Ar-H, 9 H), 7.29 (t, J = 7.7 Hz, Ar-H, 1 H), 7.10 (dd, J = 7.7, 1.5 Hz, Ar-H, 1 H), 7.06 (dd, J = 7.7, 1.5 Hz, Ar-H, 1 H), 6.33 (dd, J = 12.2, 4.2 Hz, CH, 1 H), 3.83 (t, J = 11.8 Hz, CH, 1 H), 3.51/3.31 (dd+dd, J = 14.2, 4.2 Hz, J = 14.2, 12.2 Hz, CH_2, 2 H), 3.43/3.31 (t+dd, J = 11.4 Hz, J = 11.8, 11.4 Hz, CH_2, 2 H), 2.81 (sp, J = 6.7 Hz, CH, 1 H), 2.55 (sp, J = 6.7 Hz, CH, 1 H), 1.32 (d, J = 6.7 Hz, CH_3, 3 H), 1.26 (d, J = 6.7 Hz, CH_3, 3 H), 1.23 (d, J = 6.7 Hz, CH_3, 3 H), 1.22 (d, J = 6.7 Hz, CH_3, 3 H), 0.68 (s, CH_3, 9 H). ^{13}C{1H} NMR (125 MHz, $CDCl_3$) δ 197.5, 147.1, 145.7, 138.2, 137.2, 136.2, 134.3, 130.9, 129.6, 129.5, 129.3, 128.9, 128.4, 127.4, 127.2, 125.3, 125.0, 75.6, 63.0, 45.6, 35.3, 29.2, 28.0, 27.2, 26.8, 25.9, 23.7, 23.6. HRMS calcd for ($C_{33}H_{44}AuClN_3$) $[M - Cl + NH_3]^+$ 714.2889, found 714.2905. Anal. Calcd. for $C_{33}H_{41}AuCl_2N_2$: C, 54.03; H, 5.36; N, 3.82. Found: C, 54.26; H, 5.65; N, 3.56.

(*4R*)-*4*-*tert*-Butyl-*3*-(*2’,6’*-diisopropylphenyl)-*1*-[(*1R*)-*1*-(*3*-trifluoromethyl)-*2*-phenylethyl]imidazolidin-2-ylidene-gold-chloride (C37)

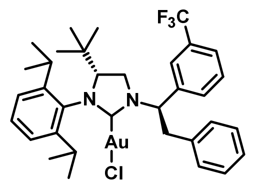

Starting material: 245 mg **B37** (0.361 mmol). Yield: 235 mg (0.306 mmol, 84.8%), light pink solid. Mp: 234-236°C. ^{1}H NMR (500 MHz, $CDCl_3$) δ 7.80 (d, J = 7.7 Hz, Ar-H, 1 H), 7.77 (brs., Ar-H, 1 H), 7.68 (d, J = 7.7 Hz, Ar-H, 1 H), 7.62 (t, J = 7.7 Hz, Ar-H, 1 H), 7.47-7.29 (m, Ar-H, 5 H), 7.29 (t, J = 7.7 Hz, Ar-H, 1 H), 7.14 (dd, J = 7.7, 1.4 Hz, Ar-H, 1 H), 7.04 (dd, J = 7.7, 1.4 Hz, Ar-H, 1 H), 6.41 (dd, J = 12.0, 5.0 Hz, CH, 1 H), 3.74 (t, J = 11.5 Hz, CH, 1 H), 3.66/3.08 (dd+t, J = 11.5, 10.6 Hz, J = 10.6 Hz, CH_2, 2 H), 3.64/3.36 (dd+dd, J = 14.5, 5.0 Hz, J = 14.5, 12.0 Hz, CH_2, 2 H), 3.30 (sp, J = 6.7 Hz, CH, 1 H), 2.02 (sp, J = 6.7 Hz, CH, 1 H), 1.55 (d, J = 6.7 Hz, CH_3, 3 H), 1.27 (d, J = 6.7 Hz, CH_3, 3 H), 1.14 (d, J = 6.7 Hz, CH_3, 3 H), 0.94 (d, J = 6.7 Hz, CH_3, 3 H), 0.59 (s, CH_3, 9 H). ^{13}C{1H} NMR (125 MHz, $CDCl_3$) δ 198.3, 146.8, 146.0, 138.8, 135.9, 135.8, 130.5, 129.9, 129.6, 129.1, 129.1, 127.5, 125.5, 125.5, 125.2, 123.5, 123.5, 75.0, 62.2, 45.9, 35.9, 28.8, 28.5, 27.1, 26.4, 26.4, 23.7, 23.5. HRMS calcd for ($C_{34}H_{41}AuF_3N_2$) [M - Cl]$^+$ 731.2887, found 731.2855.

(*4R*)-*4-tert*-Butyl-*3*-(*2',6'*-diisopropylphenyl)-*1*-[(*1S*)-*1*-(*3*-trifluoromethyl)-*2*-phenylethyl]imidazolidin-2-ylidene-gold-chloride (C38)

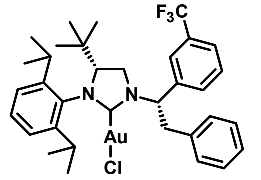

Starting material: 63 mg **B38** (0.093 mmol). Yield: 21 mg (0.027 mmol, 29.5%), light purple solid. Mp: 97-99°C. ^{1}H NMR (500 MHz, $CDCl_3$) δ 7.79 (d, J = 7.9 Hz, Ar-H, 1 H), 7.76 (brs., Ar-H, 1 H), 7.70 (d, J = 7.9 Hz, Ar-H, 1 H), 7.61 (t, J = 7.9 Hz, Ar-H, 1 H), 7.47 (d, J = 7.5 Hz, Ar-H, 2 H), 7.40 (t, J = 7.5 Hz, Ar-H, 2 H), 7.33 (t, J = 7.5 Hz, Ar-H, 1 H), 7.29 (t, J = 7.7 Hz, Ar-H, 1 H), 7.10 (dd, J = 7.7, 1.5 Hz, Ar-H, 1 H), 7.08 (dd, J = 7.7, 1.5 Hz, Ar-H, 1 H), 6.45 (dd, J = 12.1, 4.7 Hz, CH, 1 H), 3.86 (t, J = 11.8 Hz, CH, 1 H), 3.63/3.33 (dd+dd, J = 14.3, 4.7 Hz, J = 14.3, 12.1 Hz, CH_2, 2 H), 3.41/3.25 (dd+t, J = 11.8, 11.1 Hz, J = 11.1 Hz, CH_2, 2 H), 2.74 (sp, J = 6.7 Hz, CH, 1 H), 2.57 (sp, J = 6.7 Hz, CH, 1 H), 1.33 (d, J = 6.7 Hz, CH_3, 3 H), 1.28 (d, J = 6.7 Hz, CH_3, 3 H), 1.25 (d, J = 6.7 Hz, CH_3, 3 H), 1.16 (d, J = 6.7 Hz, CH_3, 3 H), 0.61 (s, CH_3, 9 H). ^{13}C{1H} NMR (125 MHz, $CDCl_3$) δ 198.1, 147.0, 145.8, 138.9, 135.9, 135.4, 130.7, 129.9, 129.8, 129.6, 129.1, 127.5, 125.6, 125.3, 125.1, 123.7, 75.6, 62.1, 45.7, 35.6, 29.4, 27.8, 27.3, 26.8, 25.8, 23.6, 23.5. HRMS calcd for ($C_{34}H_{41}AuF_3N_2$) [M - Cl]$^+$ 731.2887, found 731.2872.

***3*-(*2',6'*-diisopropylphenyl)-*1*-[(*1R*)-*2*-(*4*-chlorophenyl)-*1*-phenylethyl]imidazolidin-2-ylidene-gold-chloride (C39)**

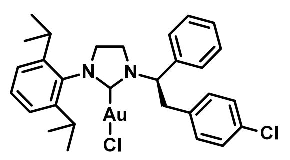

Starting material: 100 mg **B39** (0.170 mmol). Yield: 97 mg (0.143 mmol, 84.2%), light purple solid. Mp: 261-263°C. ^{1}H NMR (500 MHz, $CDCl_3$) δ 7.59-7.40 (m, Ar-H, 5 H), 7.39 (s, Ar-H, 2 H), 7.39 (s, Ar-H, 2 H), 7.33 (t, J = 7.7 Hz, Ar-H, 1 H), 7.14 (dd, J = 7.7, 1.4 Hz, Ar-H, 1 H), 7.12 (dd, J = 7.7, 1.4 Hz, Ar-H, 1 H), 6.35 (dd, J = 12.0, 4.9 Hz, CH, 1 H), 3.70/3.41 (m+m, CH_2, 2 H), 3.62/3.54 (m+m, CH_2, 2 H), 3.50/3.37 (dd+dd, J = 14.3, 4.9 Hz, J = 14.3, 12.0 Hz, CH_2, 2 H), 2.76 (sp, J = 6.7 Hz, CH, 1 H), 2.14 (sp, J = 6.7 Hz, CH, 1 H), 1.32 (d, J = 6.7 Hz, CH_3, 3 H), 1.20 (d, J = 6.7 Hz, CH_3, 3 H), 1.18 (d, J = 6.7 Hz, CH_3, 3 H), 1.09 (d, J = 6.7 Hz, CH_3, 3 H). ^{13}C{1H} NMR (125 MHz, $CDCl_3$) δ 194.2, 147.0, 146.3, 137.0, 135.0, 134.0, 133.1, 130.7, 130.0, 129.3, 129.2, 128.8, 127.2, 124.9, 124.4, 62.5, 53.1, 43.5, 35.6, 28.8, 28.4, 25.3, 24.9, 24.6, 23.8. HRMS calcd for ($C_{29}H_{34}AuCl_2N_2$) [M]$^+$ 676.1686, found 676.1699.

***3*-(*2',6'*-diisopropylphenyl)-*1*-[(*1S*)-*2*-(*4*-chlorophenyl)-*1*-phenylethyl]imidazolidin-2-ylidene-gold-chloride (C40)-H145862**

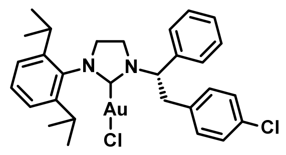

Starting material: 120 mg **B40** (0.204 mmol). Yield: 127 mg (0.188 mmol, 91.9%), light purple solid. Mp: 264-266°C. ^{1}H NMR (500 MHz, $CDCl_3$) δ 7.59-7.40 (m, Ar-H, 5 H), 7.39 (s, Ar-H, 2 H), 7.39 (s, Ar-H, 2 H), 7.33 (t, J = 7.7 Hz, Ar-H, 1 H), 7.14 (dd, J = 7.7, 1.4 Hz, Ar-H, 1 H), 7.12 (dd, J = 7.7, 1.4 Hz, Ar-H, 1 H), 6.35 (dd, J = 12.0, 4.9 Hz, CH, 1 H), 3.70/3.41 (m+m, CH_2, 2 H), 3.62/3.54 (m+m, CH_2, 2 H), 3.50/3.37 (dd+dd, J = 14.3, 4.9 Hz, J = 14.3, 12.0 Hz, CH_2, 2 H), 2.76 (sp, J = 6.7 Hz, CH, 1 H), 2.14 (sp, J = 6.7 Hz, CH, 1 H), 1.32 (d, J = 6.7 Hz, CH_3, 3 H), 1.20 (d, J = 6.7 Hz, CH_3, 3 H), 1.18 (d, J = 6.7 Hz, CH_3, 3 H), 1.09 (d, J = 6.7 Hz, CH_3, 3 H). ^{13}C{1H} NMR (125 MHz, $CDCl_3$) δ 194.2, 147.0, 146.3, 137.0, 135.0, 134.0, 133.1, 130.7, 130.0, 129.3, 129.2, 128.8, 127.2, 124.9, 124.4, 62.5, 53.1, 43.5, 35.6, 28.8, 28.4, 25.3, 24.9, 24.6, 23.8. HRMS calcd for ($C_{29}H_{34}AuCl_2N_2$) $[M]^+$ 676.1686, found 676.1653.

***3*-(*2’,6’*-diisopropylphenyl)-*1*-[(*1S*)-*1*-benzyl-*2*-hydroxiethyl]imidazolidin-2-ylidene-gold-chloride (C41)**

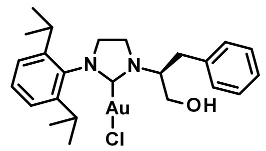

Starting material: 50 mg ***3*-(*2’,6’*-diisopropylphenyl)-*1*-[(*1S*)-*1*-benzyl-*2*-hydroxiethyl]-*4,5*-dihydroimidazolium Chloride**[2] (0.125 mmol). Yield: 52 mg (0.087 mmol, 69.9%), light purple solid. Mp: 160-162°C. ^{1}H NMR (500 MHz, $CDCl_3$) δ 7.36 (t, J = 7.2 Hz, Ar-H, 2 H), 7.34 (d, J = 7.2 Hz, Ar-H, 2 H), 7.33 (t, J = 7.7 Hz, Ar-H, 1 H), 7.29 (t, J = 7.2 Hz, Ar-H, 1 H), 7.15 (dd, J = 7.7, 1.4 Hz, Ar-H, 1 H), 7.12 (dd, J = 7.7, 1.4 Hz, Ar-H, 1 H), 5.07 (m, CH, 1 H), 4.02/3.94 (dt+dt, J = 11.7, 4.5 Hz, J = 11.7, 7.3 Hz, CH_2, 2 H), 3.84/3.75 (m+m, CH_2, 2 H), 3.71/3.55 (m+m, CH_2, 2 H), 3.09/3.00 (dd+dd, J = 14.4, 10.5 Hz, J = 14.4, 10.5 Hz, CH_2, 2 H), 2.89 (sp, J = 6.7 Hz, CH, 1 H), 2.33 (sp, J = 6.7 Hz, CH, 1 H), 1.75 (dd, J = 7.3, 4.5 Hz, OH, 1 H), 1.32 (d, J = 6.7 Hz, CH_3, 3 H), 1.21 (d, J = 6.7 Hz, CH_3, 3 H), 1.19 (d, J = 6.7 Hz, CH_3, 3 H), 1.07 (d, J = 6.7 Hz, CH_3, 3 H). ^{13}C{1H} NMR (125 MHz, $CDCl_3$) δ 194.4, 146.8, 146.7, 136.4, 134.4, 129.9, 129.2, 129.0, 127.2, 124.8, 124.6, 63.6, 62.3, 53.0, 45.5, 35.3, 28.6, 28.2, 25.2, 25.2, 24.6, 24.2. HRMS calcd for ($C_{26}H_{35}AuN_3O$) $[M - Cl + CH_3CN]^+$ 602.2446, found 602.2439.

(*4S*)-*4*-*tert*-Butyl-*3*-(*2’,6’*-diisopropylphenyl)-*1*-[(*1S*)-*1*-benzyl-*2*-hydroxiethyl]imidazolidin-2-ylidene-gold-chloride (C42)

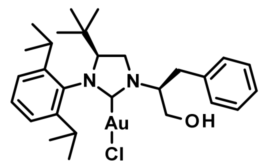

Starting material: 50 mg **(*4S*)-*4*-*tert*-Butyl-*3*-(*2’,6’*-diisopropylphenyl)-*1*-[(*1S*)-*1*-benzyl-*2*-hydroxiethyl]-*4,5*-dihydroimidazolium Chloride**[2] (0.109 mmol). Yield: 20 mg (0.031 mmol, 28.0%), light purple solid. Mp: 195-197°C. ^{1}H NMR (500 MHz, $CDCl_3$) δ 7.39-7.24 (m, Ar-H, 5 H), 7.30 (t, J = 7.7 Hz, Ar-H, 1 H), 7.14 (dd, J = 7.7, 1.4 Hz, Ar-H, 1 H), 7.06 (dd, J = 7.7, 1.4 Hz, Ar-H, 1 H), 5.01 (m, CH, 1 H), 4.05-3.93 (m, CH_2, 2 H), 3.76 (t, J = 11.0 Hz, CH, 1 H), 3.68/3.59 (t, J = 11.0 Hz, J = 11.0 Hz, CH_2, 1 H), 3.36 (sp, J = 6.7 Hz, CH, 1 H), 3.09 (d, J = 8.0 Hz, CH_2, 2 H), 2.24 (sp, J = 6.7 Hz, CH, 1 H), 1.68 (dd, J = 6.6, 4.6 Hz, OH, 1 H), 1.52 (d, J = 6.7 Hz, CH_3, 3 H), 1.27 (d, J = 6.7 Hz, CH_3, 3 H), 1.18 (d, J = 6.7 Hz, CH_3, 3 H), 1.04 (d, J = 6.7 Hz, CH_3, 3 H), 0.71 (s, CH_3, 9 H). ^{13}C{1H} NMR (125 MHz, $CDCl_3$) δ 197.5, 147, 146.1, 125.2, 125.2, 75.2, 64, 62.4, 35.2, 28.8, 28.2, 27.2, 26.6, 26.2, 23.7, 23.6. HRMS calcd for ($C_{30}H_{43}AuN_3O$) $[M - Cl + CH_3CN]^+$ 658.3062, found 658.3040.

7. Preparation of Acetylene Derivatives (1a-d)

0.01 equivalent of magnesium perchlorate were suspended in dimethylpropanoyl-2,2-dimethylpropanoate (1.05 equivalent), which suspension was stirred for 30 minutes at 40°C. After half

an hour, the appropriate alcohol derivative was added and stirred for overnight at 70°C. The transformation was monitored with a GC-MS.

After complete conversion, the mixture was allowed to cool to room temperature, then saturated aqueous $NaHCO_3$ was added until neutral pH was reached. It was then extracted with diethylether and the combined organic phase was dried over $MgSO_4$. The crude product was purified by short-path vacuum distillation.

***1*,*1*-dimethylprop-*2*-ynyl-*2*,*2*-dimethylpropanoate (1a)**

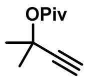

Starting materials: 20.0 g 2-methylbut-3-yn-2-ol (238 mmol). Yield: 25.3 g (150 mmol, 63.2%), colorless oil. Bp: 177-179°C. ^1H NMR (400 MHz, DMSO-*d6*) δ 3.46 (s, CH, 1 H), 1.58 (s, CH_3, 6 H), 1.11 (s, CH_3, 9 H). $^{13}C\{1H\}$ NMR (100 MHz, DMSO-*d6*) δ 175.5, 74.7, 70.8, 38.5, 28.4, 26.7. HRMS calcd for ($C_{10}H_{16}O_2$) $[M]^+$ 168.1150, found 168.1148.

(*1*-ethynylcyclohexyl)-*2*,*2*-dimethylpropanoate (1b)

1b was identified as the residue from the distillation.

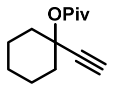

Starting materials: 15.0 g 1-ethynylcyclohexanol (121 mmol). Yield: 18.2 g (87.5 mmol, 72.4%), orange oil. ^1H NMR (500 MHz, DMSO-*d6*) δ 3.51 (s, CH, 1 H), 1.92 (m, CH_2, 4 H), 1.51 (m, CH_2, 4 H), 1.39 (m, CH_2, 2 H), 1.13 (s, CH_3, 9 H). $^{13}C\{1H\}$ NMR (125 MHz, DMSO-*d6*) δ 175.3, 76.3, 73.6, 38.7, 36.1, 26.8, 24.4, 21.7. HRMS calcd for ($C_9H_{11}O_2$) $[M - C_4H_9]^+$ 151.0754 found 151.0761.

***1*-phenylprop-*2*-ynyl-*2*,*2*-dimethylpropanoate (1c)**

After extraction **1c** was purified by column chromatography, instead of distillation, using heptane/acetone eluents.

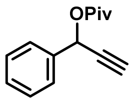

Starting materials: 15.0 g 1-phenylprop-2-yn-1-ol (114 mmol). Yield: 3.05 g (14.1 mmol, 12.4%), yellow oil. ^1H NMR (500 MHz, DMSO-*d6*) δ 7.55-7.32 (m, Ar-H, 5 H), 6.38 (d, J = 2.2 Hz, CH, 1 H), 3.78 (d, J = 2.2 Hz, CH, 1 H), 1.15 (s, CH_3, 9 H). $^{13}C\{1H\}$ NMR (125 MHz, DMSO-*d6*) δ 176.0, 136.8, 128.8, 128.7, 127.0, 80.6, 78.3, 64.7, 38.2, 26.6. HRMS calcd for ($C_{14}H_{16}O_2$) $[M]^+$ 216.1150 found 216.1157.

***tert*-butyl-*3*-(*1*-acetoxyprop-*2*-ynyl)benzoate (1d)**

After extraction **1d** was purified by column chromatography, instead of distillation, using heptane/EtOAc eluents.

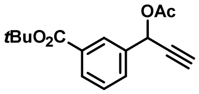

Starting materials: 1.16 g tert-butyl 3-(1-hydroxyprop-2-ynyl)benzoate (5 mmol). Yield: 959 mg (3.5 mmol, 70%), yellow oil. ^1H NMR (500 MHz, DMSO-*d6*) δ 8.06 (t, J = 1.8 Hz, 1 H), 7.93 (dm, 1 H), 7.76 (dm, 1 H), 7.55 (t, J = 7.8 Hz, 1 H), 6.49 (d, J = 2.2 Hz, 1 H), 3.85 (d, J = 2.2 Hz, 1 H), 2.09 (s, 3 H), 1.55 (s, 9 H). $^{13}C\{1H\}$ NMR (125 MHz, DMSO-*d6*) δ 169.1, 164.4, 137.2, 131.8, 131.6, 129.5, 129.1, 127.8, 81.0, 78.8, 64.3, 27.7, 20.5. HRMS calcd for ($C_{16}H_{18}O_4$) $[M]^+$ 274.1205 found 274.1204.

8. General Procedure for Preparation of Substituated Cyclopropyl Ring (3a-t, 4a-e,g-j,l,o,q-t)

1 mol% **C36** and 1 mol% $AgSbF_6$ catalysts were placed in a 4 mL vial, then dissolved in the mixture of TFE 0.25 mL/mmol. This mixture was stirred at room temperature for 10 minutes. In an other 4 mL vial the corresponding 1 equivalent of acetylene (**1a-d**) and the corresponding 1.25 equivalents of olefin derivative (**2a-i**) were mixed and dissolved in TFE : HFIPA (0.25 mL : 0.50 mL/mmol)

The two vials were cooled to -25°C using a freezer and after reaching the specified temperature, they were combined. The reaction mixture was left to stand for 60 hours at the mentioned temperature without stirring. The transformation was followed by HPLC and GC-MS systems.

During the work up procedure, the reaction mixture was evaporated and the resulting mixture of diastereomers were dissolved in 2-3 mL of DCM and were separated by flash chromatography using 120 g Silica Gold column.

[*1*-(*cis*-*2*-acetoxy-*2*-methyl-cyclopropyl)-*2*-methyl-prop-*1*-enyl] *2,2*-dimethylpropanoate (3a) and [*1*-(*trans*-*2*-acetoxy-*2*-methyl-cyclopropyl)-*2*-methyl-prop-*1*-enyl] *2,2*-dimethylpropanoate (4a)

Starting materials: 168 mg **1a** (1 mmol) és 125 mg **2a** (1.25 mmol). dr: 71/29, ee%: 92/88 (Amylose-3, Heptane/IPA, 97/3, 0.5 mL/min).

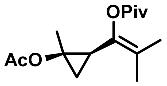

Yield (**3a**): 129 mg (0.480 mmol, 68%), yellow oil. ^{1}H NMR (500 MHz, DMSO-d_6) δ 1.90 (s, CH_3, 3 H), 1.86 (t, J = 8.6 Hz, CH, 1 H), 1.73/1.48 (s+s, CH_3, 6 H), 1.48 (s, CH_3, 3 H), 1.18 (s, CH_3, 9 H), 1.03/0.77 (dd+t, J = 10.0, 6.7 Hz, J = 6.7 Hz, CH_2, 2 H). ^{13}C{1H} NMR (125 MHz, DMSO-d_6) δ 175.1, 169.9, 137.0, 121.2, 59.1, 38.4, 27.1, 26.9, 23.6, 21.2, 21.0, 18.7, 17.6, 17.1. HRMS calcd for ($C_{15}H_{28}NO_4$) [M + NH_4]$^+$ 286.2013, found 286.2006.

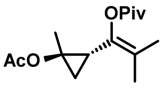

Yield (**4a**): 32 mg (0.120 mmol, 42%), yellow oil. ^{1}H NMR (500 MHz, DMSO-d_6) δ 2.02 (dm, J = 9.0 Hz, CH, 1 H), 1.94 (s, CH_3, 3 H), 1.77/1.51 (s+s, CH_3, 6 H), 1.38 (s, CH_3, 3 H), 1.21 (s, CH_3, 9 H), 1.14/0.62 (dd+dd, J = 10.5, 6.3 Hz, J = 7.2, 6.3 Hz, CH_2, 2 H). ^{13}C{1H} NMR (125 MHz, DMSO-d_6) δ 175.3, 169.9, 137.5, 122.4, 59.6, 38.4, 26.9, 24, 21.1, 18.4, 17.4. HRMS calcd for ($C_{15}H_{25}O_4$) [M + H]$^+$ 269.17, found 269.1745.

[(*cis*-*2*-acetoxy-*2*-methyl-cyclopropyl)-cyclohexylidene-methyl] *2,2*-dimethylpropanoate (3b) and [(*trans*-*2*-acetoxy-*2*-methyl-cyclopropyl)-cyclohexylidene-methyl] *2,2*-dimethylpropanoate (4b)

Starting materials: 208 mg **1b** (1 mmol) and 125 mg **2a** (1.25 mmol). dr: 68/32, ee%: 90/84 (Amylose-3, Heptane/IPA, 97/3, 0.5 mL/min).

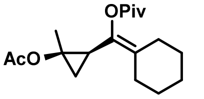

Yield (**3b**): 142 mg (0.462 mmol, 68%), colorless oil. ^{1}H NMR (500 MHz, DMSO-d_6) δ 2.32-1.29 (m, CH_2, 10 H), 1.91 (s, CH_3, 3 H), 1.85 (dd, J = 9.9, 7.9 Hz, CH, 1 H), 1.47 (s, CH_3, 3 H), 1.18 (s, CH_3, 9 H), 1.02/0.76 (dd+dd, J = 9.9, 6.5 Hz, J = 6.5 Hz, CH_2, 2 H). ^{13}C{1H} NMR (125 MHz, DMSO-d_6) δ 175.3, 169.8, 134.1, 128.7, 59.1, 38.4, 26.9, 23.3, 21.1, 21.0, 17.0. HRMS calcd for ($C_{18}H_{32}NO_4$) [M + NH_4]$^+$ 326.2326 found 326.2324.

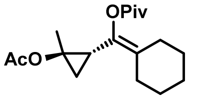

Yield (**4b**): 59 mg (0.192 mmol, 60%), yellow oil. ^{1}H NMR (500 MHz, DMSO-d_6) δ 2.38-1.33 (m, CH_2, 10 H), 2.02 (dd, J = 10.7, 7.2 Hz, CH, 1 H), 1.94 (s, CH_3, 3 H), 1.39 (s, CH_3, 3 H), 1.20 (s, CH_3, 9 H), 1.14/0.61 (dd+dd, J = 10.7, 6.3 Hz, J = 7.2, 6.3 Hz, CH_2, 2 H). ^{13}C{1H} NMR (125 MHz, DMSO-d_6) δ 175.5, 169.9, 134.7, 129.8, 59.7, 38.4, 23.7, 23.7, 21.0, 17.5, 17.5. HRMS calcd for ($C_{18}H_{32}NO_4$) [M + NH_4]$^+$ 326.2326 found 326.2327.

[*(Z)*-*1*-(*cis*-*2*-acetoxy-*2*-methyl-cyclopropyl)-*2*-phenyl-vinyl] *2,2*-dimethylpropanoate (3c) and [*(Z)*-*1*-(*trans*-*2*-acetoxy-*2*-methyl-cyclopropyl)-*2*-phenyl-vinyl] *2,2*-dimethylpropanoate (4c)

Starting materials: 216 mg **1c** (1 mmol) and 125 mg **2a** (1.25 mmol). dr: 73/27, ee%: 56/64 (Amylose-1, Heptane/IPA, 99/1, 0.5 mL/min).

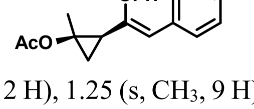

Yield (**3c**): 166 mg (0.530 mmol, 76%), yellow oil. ^{1}H NMR (500 MHz, DMSO-d_6) δ 7.40-7.20 (m, Ar-H, 5 H), 6.18 (s, CH, 1 H), 2.10 (dd, J = 10.3, 7.30 Hz, CH, 1 H), 1.95 (s, CH_3, 3 H), 1.46 (s, CH_3, 3 H), 1.26/1.05 (m+t, J = 7.0 Hz, CH_2, 2 H), 1.25 (s, CH_3, 9 H). ^{13}C{1H} NMR (125 MHz, DMSO-d_6) δ 175.2, 169.9, 146.4, 133.8, 117.4, 59.7, 38.5, 27.2, 26.8, 21.1, 17.3, 17.0. HRMS calcd for ($C_{19}H_{25}O_4$) [M + H]$^+$ 317.1747, found 317.1749.

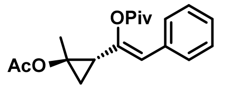

Yield (**4c**): 75 mg (0.237 mmol, 88%), yellow oil. ^{1}H NMR (500 MHz, DMSO-d_6) δ 7.38-7.14 (m, Ar-H, 5 H), 6.17 (s, CH, 1 H), 1.91 (s, CH_3, 3 H), 1.85 (dd, J = 9.2, 8.2 Hz, CH, 1 H), 1.51 (s, CH_3, 3 H), 1.21 (s, CH_3, 9 H), 1.20-1.12 (m, CH_2, 2 H). ^{13}C{1H} NMR (125 MHz, DMSO-d_6) δ 175.1, 169.9, 145.4, 134.1, 116.7, 59.8, 38.5, 26.9, 26.7, 21.3, 20.9, 17.3. HRMS calcd for ($C_{19}H_{25}O_4$) [M + H]$^+$ 317.1747, found 317.1748.

[*1*-[*cis*-*2*-(*3*-methoxy-*3*-oxo-propyl)cyclopropyl]-*2*-methyl-prop-*1*-enyl] *2,2*-dimethylpropanoate (3d) and [*1*-[*trans*-*2*-(*3*-methoxy-*3*-oxo-propyl)cyclopropyl]-*2*-methyl-prop-*1*-enyl] *2,2*-dimethylpropanoate (4d)

Starting materials: 168 mg **1a** (1 mmol) and 142 mg **2b** (1.25 mmol). dr: 54/46, ee%: 56/64 (Amylose-3, Heptane/IPA, 97/3, 0.5 mL/min).

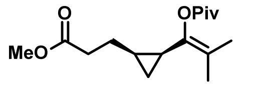

Yield (**3d**): 88 mg (0.313 mmol, 58%), yellow oil. ^{1}H NMR (500 MHz, DMSO-d_6) δ 3.58 (s, CH_3, 3 H), 2.36 (td, J = 7.4, 2.9 Hz, CH_2, 2 H), 1.74 (s, CH_3, 3 H), 1.73 (m, CH, 1 H), 1.50 (s, CH_3, 3 H), 1.63/1.24 (m+m, CH_2, 2 H), 1.20 (s, CH_3, 9 H), 0.99 (m, CH, 1 H), 0.73/0.14 (m+m, CH_2, 2 H). ^{13}C{1H} NMR (125 MHz, DMSO-d_6) δ 175.2, 173.2, 139.4, 120.6, 51.2, 33.2, 27.0, 24.7, 18.5, 17.4, 17.1, 16.5, 9.7. HRMS calcd for ($C_{16}H_{27}O_4$) [M + H]$^+$ 283.1904, found 283.1905.

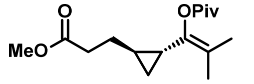

Yield (**4d**): 86 mg (0.312 mmol, 64%), yellow oil. ^{1}H NMR (500 MHz, DMSO-d_6) δ 3.57 (s, CH_3, 3 H), 2.35 (t, J = 7.0 Hz, CH_2, 2 H), 1.74 (s, CH_3, 3 H), 1.50 (m, CH, 1 H), 1.48 (m, CH_2, 2 H), 1.42 (s, CH_3, 3 H), 1.18 (s, CH_3, 9 H), 0.82 (m, CH, 1 H), 0.49 (m, CH_2, 2 H). ^{13}C{1H} NMR (125 MHz, DMSO-d_6) δ 175.3, 173.1, 140.8, 118.0, 51.2, 33.0, 28.4, 27.0, 18.2, 17.9, 17.6, 17.3, 11.6. HRMS calcd for ($C_{16}H_{27}O_4$) [M + H]$^+$ 283.1904, found 283.1905.

[*1*-[*cis*-*2*-[*2*-(*1,3*-dioxoisoindolin-*2*-yl)ethyl]cyclopropyl]-*2*-methyl-prop-*1*-enyl] *2,2*-dimethylpropanoate (3e) and [*1*-[*trans*-*2*-[*2*-(*1,3*-dioxoisoindolin-*2*-yl)ethyl]cyclopropyl]-*2*-methyl-prop-*1*-enyl] *2,2*-dimethylpropanoate (4e)

Starting materials: 168 mg **1a** (1 mmol) and 251 mg **2c** (1.25 mmol). dr: 57/43, ee%: 77/75 (Amylose-3, Heptane/IPA, 97/3, 0.5 mL/min).

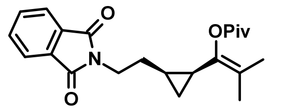

Yield (**3e**): 122 mg (0.330 mmol, 58%), yellow oil. ^{1}H NMR (500 MHz, DMSO-d_6) δ 7.89-7.80 (m, Ar-H, 4 H), 3.72-3.58 (m, CH_2, 2 H), 1.73/1.44 (m+m, CH_2, 2 H), 1.72 (m, CH, 1 H), 1.61(s, CH_3, 3 H), 1.25 (s, CH_3, 3 H), 1.16 (s, CH_3, 3 H), 1.00 (m, CH, 1 H), 0.70/0.04 (m+m, CH_2, 2 H). ^{13}C{1H} NMR (125 MHz, DMSO-d_6) δ 175.1, 167.9, 139.4, 131.7, 120.1, 37.4, 27.7, 26.9, 18.3, 17.2, 15.9, 15.8, 9.3. HRMS calcd for ($C_{22}H_{28}NO_4$) [M + H]$^+$ 370.2013 found 370.2014.

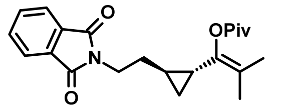

Yield (**4e**): 79 mg (0.215 mmol, 50%), yellow oil. ^{1}H NMR (500 MHz, DMSO-d_6) δ 7.95-7.72 (m, Ar-H, 4 H), 3.73-3.54 (m, CH_2, 2 H), 1.69/1.47 (m+m, CH_2, 2 H), 1.60 (s, CH_3, 3 H), 1.32 (s, CH_3, 3 H), 1.47 (m, CH, 1 H), 1.12 (s, CH_3, 9 H), 0.76 (m, CH, 1 H), 0.47-0.39 (m, CH_2, 2 H). ^{13}C{1H} NMR (125 MHz, DMSO-d_6) δ 175.3, 167.9, 140.2, 131.6, 117.7, 37.1, 31.4, 18.0, 17.9, 17.6, 17.5, 15.2, 10.9. HRMS calcd for ($C_{22}H_{27}NO_4Na$) [M + Na]$^+$ 392.1832 found 392.1834.

[*2*-methyl-*1*-[*cis*-*2*-(*3*-thienyl)cyclopropyl]prop-*1*-enyl] *2,2*-dimethylpropanoate (3f)

Starting materials: 168 mg **1a** (1 mmol) and 137 mg **2d** (1.25 mmol). dr (cis/trans): 100/0, ee%: 56 (Amylose-1, Heptane/IPA, 99/1, 0.5 mL/min).

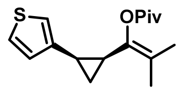

Yield (**3f**): 183 mg (0.330 mmol, 66%), yellow oil. ^{1}H NMR (500 MHz, DMSO-d_6) δ 7.37 (dd, J = 5.0, 3.0 Hz, 1 H), 7.02 (dd, J = 3.0, 1.2 Hz, 1 H), 6.79 (dd, J = 5.0, 1.2 Hz, 1 H), 2.33 (m, 1 H), 2.09 (m, 1 H), 1.51/1.37 (d+d, J = 0.8 Hz / J = 1.4 Hz, 6

H), 1.2/0.83 (m+m, 2 H), 2.3 (s, 9 H). $^{13}C\{1H\}$ NMR (125 MHz, DMSO-d_6) δ 175.9, 140.9, 138.9, 127.6, 125.2, 122.1, 120.1, 27.3, 20.9, 19.4, 18.7/17.6, 12.6. HRMS calcd for ($C_{16}H_{23}O_2S$) $[M + H]^+$ 279.1427 found 279.1413.

[*1*-[*cis*-*2*-(*4*-chlorophenyl)cyclopropyl]-*2*-methyl-prop-*1*-enyl] *2,2*-dimethylpropanoate (3g) and [*1*-[*trans*-*2*-(*4*-chlorophenyl)cyclopropyl]-*2*-methyl-prop-*1*-enyl] *2,2*-dimethylpropanoate (4g)

Starting materials: 168 mg **1a** (1 mmol) and 137 mg **2e** (1.25 mmol). dr (cis/trans): 80/20, ee%: 26/24 (Amylose-3, Heptane/IPA, 97/3, 0.5 mL/min).

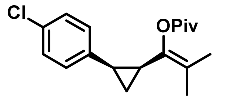

Yield (**3g**): 182 mg (0.656 mmol, 82%), yellow oil. ^{1}H NMR (500 MHz, DMSO-d_6) δ 7.28 (dm, J = 8.5 Hz, Ar-H, 2 H), 7.06 (dm, J = 8.5 Hz, Ar-H, 2 H), 2.32 (m, CH, 1 H), 2.18 (m, CH, 1 H), 1.48 (s, CH_3, 3 H), 1.35 (s, CH_3, 3 H), 1.24/0.94 (m+m, CH_2, 2 H), 1.13 (s, CH_3, 9 H). $^{13}C\{1H\}$ NMR (125 MHz, DMSO-d_6) δ 175.4, 138.4, 137.8, 130.0, 128.9, 127.5, 122.1, 26.8, 22.6, 21.5, 18.3, 17.1, 11.4. HRMS calcd for ($C_{18}H_{24}ClO_2$) $[M + H]^+$ 307.1459 found 307.1460.

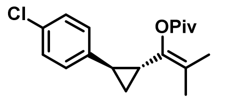

Yield (**4g**): 48 mg (0.156 mmol, 78%), yellow oil. ^{1}H NMR (500 MHz, DMSO-d_6) δ 7.30 (dm, J = 8.5 Hz, Ar-H, 2 H), 7.14 (dm, J = 8.5 Hz, Ar-H, 2 H), 2.00-1.94 (m, CH, 1 H), 2.00-1.94 (m, CH, 1 H), 1.73 (s, CH_3, 3 H), 1.47 (s, CH_3, 3 H), 1.22 (s, CH_3, 9 H), 1.16/1.04 (m+m, CH_2, 2 H). $^{13}C\{1H\}$ NMR (125 MHz, DMSO-d_6) δ 175.6, 141.0, 139.8, 130.1, 128.2, 127.4, 119.5, 26.9, 23.4, 22.0, 18.3, 17.6, 14.8. HRMS calcd for ($C_{18}H_{24}ClO_2$) $[M + H]^+$ 307.1459 found 307.1461.

[*1*-[*cis*-*2*-(*3*-bromophenyl)cyclopropyl]-*2*-methyl-prop-1-enyl] *2,2*-dimethylpropanoate (3h) and [*1*-[*trans*-*2*-(*3*-bromophenyl)cyclopropyl]-*2*-methyl-prop-1-enyl] *2,2*-dimethylpropanoate (4h)

Starting materials: 168 mg **1a** (1 mmol) and 229 mg **2f** (1.25 mmol). dr (cis/trans): 83/17, ee%: 0/0 (Amylose-3, Heptane/IPA, 97/3, 0.5 mL/min).

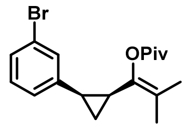

Yield (**3h**): 210 mg (0.597 mmol, 72%), yellow oil. ^{1}H NMR (500 MHz, DMSO-d_6) δ 7.33 (dm, J = 7.8 Hz, Ar-H, 1 H), 7.21 (t, J = 1.8 Hz, Ar-H, 1 H), 7.18 (t, J = 7.8 Hz, Ar-H, 1 H), 7.06 (d, J = 7.8 Hz, Ar-H, 1 H), 2.33 (m, CH, 1 H), 2.20 (m, CH, 1 H), 1.49 (s, CH_3, 3 H), 1.36 (s, CH_3, 3 H), 1.26/0.96 (m+m, CH_2, 2 H), 1.15 (s, CH_3, 9 H). $^{13}C\{1H\}$ NMR (125 MHz, DMSO-d_6) δ 175.3, 142.3, 137.7, 129.7, 129.6, 128.2, 126.4, 122.2, 121.0, 38.3, 26.8, 22.7, 21.7, 18.3, 17.1, 11.6. HRMS calcd for ($C_{19}H_{24}BrO_2$) $[M + H]^+$ 351.0954, found 351.0957.

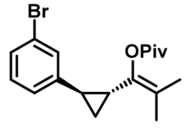

Yield (**4h**): 39 mg (0.112 mmol, 66%), yellow oil. ^{1}H NMR (500 MHz, DMSO-d_6) δ 7.34 (dd, J = 7.8, 1.1 Hz, Ar-H, 1 H), 7.33 (m, Ar-H, 1 H), 7.21 (t, J = 7.8 Hz, Ar-H, 1 H), 7.12 (dt, J = 7.8, 1.1 Hz, Ar-H, 1 H), 2.03 (m, CH, 1 H), 1.98 (m, CH, 1 H), 1.73 (s, CH_3, 3 H), 1.47 (s, CH_3, 3 H), 1.22 (s, CH_3, 9 H), 1.20/1.04 (m+m, CH_2, 2 H). $^{13}C\{1H\}$ NMR (125 MHz, DMSO-d_6) δ 175.5, 144.9, 139.8, 130.4, 128.5, 128.3, 124.7, 121.8, 119.6, 26.9, 23.5, 22.1, 18.3, 17.6, 14.9. HRMS calcd for ($C_{18}H_{24}BrO_2$) $[M + H]^+$ 351.0960 found 351.0954.

***2*-methyl-*1*-[*cis*-*2*-(o-tolyl)cyclopropyl]prop-*1*-enyl] *2,2*-dimethylpropanoate (3i) and *2*-methyl-*1*-[*trans*-*2*-(o-tolyl)cyclopropyl]prop-*1*-enyl] *2,2*-dimethylpropanoate (4i)**

Starting materials: 168 mg **1a** (1 mmol) and 147 mg **2g** (1.25 mmol). dr (cis/trans): 75/25, ee%: 46/16 (Amylose-3, Heptane/IPA, 97/3, 0.5 mL/min).

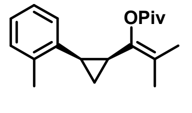

Yield (**3i**): 163 mg (0.570 mmol, 76%), yellow oil.^{1}H NMR (500 MHz, DMSO-d_6) δ 7.10 (m, Ar-H, 1 H), 7.07-7.02 (m, Ar-H, 2 H), 6.90 (m, Ar-H, 1 H), 2.32 (s, CH_3, 3 H), 2.30 (m, CH, 1 H), 2.25 (m, CH, 1 H), 1.65 (s, CH_3, 3 H), 1.29 (s, CH_3, 3 H), 1.17/0.99 (m+m, CH_2, 2 H), 1.04 (s, CH_3, 9 H). $^{13}C\{1H\}$ NMR (125 MHz, DMSO-

d_6) δ 175.1, 138.7, 137.4, 136.6, 129.3, 126.5, 125.7, 125.0, 120.2, 38.2, 26.8, 20.5, 20.0, 19.3, 18.4, 17.5, 9.4. HRMS calcd for ($C_{19}H_{27}O_2$) $[M + H]^+$ 287.2006, found 287.2006.

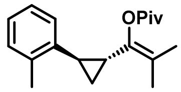

Yield (**4i**): 44 mg (0.155 mmol, 62%), yellow oil.^{1}H NMR (500 MHz, DMSO-d_6) δ 7.13 (m, Ar-H, 1 H), 7.12-7.05 (m, Ar-H, 2 H), 6.97 (m, Ar-H, 1 H), 2.30 (s, CH_3, 3 H), 1.95 (m, CH, 1 H), 1.93 (m, CH, 1 H), 1.76 (s, CH_3, 3 H), 1.48 (s, CH_3, 3 H), 1.23 (s, CH_3, 9 H), 1.09/1.02 (m+m, CH_2, 2 H). ^{13}C{1H} NMR (125 MHz, DMSO-d_6) δ 175.4, 140.0, 139.3, 136.9, 129.5, 125.9, 125.8, 124.9, 118.4, 38.6, 26.9, 20.9, 19.7, 19.3, 18.3, 17.8, 12.4. HRMS calcd for ($C_{19}H_{27}O_2$) $[M + H]^+$ 287.2006, found 287.2006.

[*2*-methyl-*1*-[*cis*-*2*-(*4*-nitrophenyl)cyclopropyl]prop-*1*-enyl] *2,2*-dimethylpropanoate (3j) and [*2*-methyl-*1*-[*trans*-*2*-(*4*-nitrophenyl)cyclopropyl]prop-*1*-enyl] *2,2*-dimethylpropanoate (4j)

Starting materials: 168 mg **1a** (1 mmol) and 186 mg **2h** (1.25 mmol). dr (cis/trans): 87/13, ee%: 35/17 (Amylose-3, Heptane/IPA, 97/3, 0.5 mL/min).

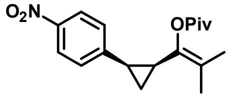

Yield (**3j**): 152 mg (0.478 mmol, 55%), yellow oil. ^{1}H NMR (500 MHz, DMSO-d_6) δ 8.08 (m, 2 H), 7.28 (m, 2 H), 2.5 (m, 1 H), 2.34 (m, 1 H), 1.44/1.34 (s+s, 6 H), 1.38/1.1 (m+m, 2 H), 1.13 (s, 9 H). ^{13}C{1H} NMR (125 MHz, DMSO-d_6) δ 175.2, 148.5, 145.3, 137.2, 127.9, 122.8, 122.5, 38.2, 26.7, 23.4, 23.0, 18.1/16.8, 12.8. HRMS calcd for ($C_{18}H_{24}NO_4$) $[M + H]^+$ 318.1714, found 318.1701.

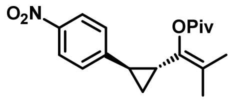

Yield (**4j**): 26 mg (0.084 mmol, 65%), yellow oil. ^{1}H NMR (500 MHz, DMSO-d_6) δ 8.11 (m, 2 H), 7.39 (m, 2 H), 2.17 (m, 1 H), 2.14 (m, 1 H), 1.72/1.48 (s+s, 6 H), 1.33/1.18 (m+m, 2 H), 1.23 (s, 9 H). ^{13}C{1H} NMR (125 MHz, DMSO-d_6) δ 175.5, 150.8, 145.5, 139.3, 126.5, 123.5, 120.1, 38.5, 26.9, 24.9, 22.7, 18.3, 17.6, 15.9. HRMS calcd for ($C_{18}H_{24}NO_4$) $[M + H]^+$ 318.1714, found 318.1696.

[*2*-methyl-*1*-(*cis*-*2*-phenylcyclopropyl)prop-*1*-enyl] *2,2*-dimethylpropanoate (3k)

Starting materials: 168 mg **1a** (1 mmol) and 130 mg **2i** (1.25 mmol). dr (cis/trans): 100/0, ee%: 30/- (Amylose-1, Heptane/IPA, 99/1, 0.5 mL/min).

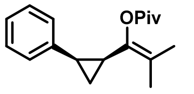

Yield (**3k**): 217 mg (0.800 mmol, 80%), yellow oil.^{1}H NMR (500 MHz, DMSO-d_6) δ 7.26-6.99 (m, 5 H), 2.3 (m, 1 H), 2.17 (m, 1 H), 1.47/1.33 (d+d, J = 1.4, 1.0 Hz, 6 H), 1.21/0.95 (m+m, 2 H), 1.13 (s, 9 H). ^{13}C{1H} NMR (125 MHz, DMSO-d_6) δ 175.4, 139.1, 138.1, 127.5, 127.1, 125.4, 121.6, 38.2, 26.8, 23.1, 21.3, 18.2/17.0, 11.1. HRMS calcd for ($C_{18}H_{25}O_2$) $[M + H]^+$ 273.1863, found 273.1850.

[*2*-methyl-*1*-(*cis*-*2*-methyl-*3*-phenyl-cyclopropyl)prop-*1*-enyl] *2,2*-dimethylpropanoate (3l) and [*2*-methyl-*1*-(*trans*-*2*-methyl-*3*-phenyl-cyclopropyl)prop-*1*-enyl] *2,2*-dimethylpropanoate (4l)

Starting materials: 168 mg **1a** (1 mmol) and 130 mg **2j** (1.25 mmol). dr (cis/trans): 74/26, ee%: 16/56 (Amylose-1, Heptane/IPA, 99/1, 0.5 mL/min).

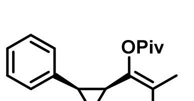

Yield (**3l**): 184 mg (0.643 mmol, 87%), yellow oil. ^{1}H NMR (500 MHz, DMSO-d_6) δ 7.20 (tm, J = 7.7 Hz, Ar-H, 2 H), 7.11 (tm, J = 7.7 Hz, Ar-H, 1 H), 6.99 (dm, J = 7.7 Hz, Ar-H, 2 H), 2.05 (dd, J = 8.6, 5.7 Hz, CH, 1 H), 1.91 (m, CH, 1 H), 1.41 (s, CH_3, 3 H), 1.36 (s, CH_3, 3 H) 1.29 (m, CH, 1 H), 1.15 (d, J = 6.0 Hz, CH_3, 3 H), 1.13 (s, CH_3, 9 H). ^{13}C{1H} NMR (125 MHz, DMSO-d_6) δ 175.4, 139.5, 138.1, 121.5, 38.3, 31.8, 29.8, 26.8, 19.9, 18.3, 18.2, 17.0. HRMS calcd for ($C_{19}H_{27}O_2$) $[M + H]^+$ 287.2006, found 287.2005.

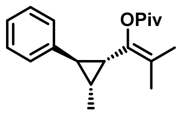

Yield (**4l**): 62 mg (0.216 mmol, 83%), yellow oil. ^{1}H NMR (500 MHz, DMSO-d_6) δ 7.28-7.01 (m, Ar-H, 5 H), 2.02 (m, CH, 1 H), 1.76 (s, CH_3, 3 H), 1.61 (t, J = 5.4 Hz, CH, 1 H), 1.54 (s, CH_3, 3 H), 1.47 (m, CH, 1 H), 1.23 (s, CH_3, 9 H), 1.10 (d, J = 6.2 Hz, CH_3, 3 H). ^{13}C{1H} NMR (125 MHz, DMSO-d_6) δ 175.4, 142.0, 139.2, 138.6,

128.2, 125.6, 121.3, 38.4, 29.2, 28.5, 27.0, 23.1, 18.5, 17.3, 14.2. HRMS calcd for ($C_{19}H_{27}O_2$) $[M + H]^+$ 287.2006, found 287.2007.

[*1*-(*cis*-*2,3*-diphenylcyclopropyl)-*2*-methyl-prop-*1*-enyl] *2,2*-dimethylpropanoate (3m)

Starting materials: 168 mg **1a** (1 mmol) and 225 mg **2k** (1.25 mmol). dr (cis/trans): 100/0, ee%: 44/- (Amylose-1, Heptane/IPA, 99/1, 0.5 mL/min).

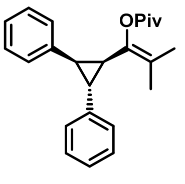

Yield (**3m**): 181 mg (0.520 mmol, 52%), yellow oil. ^{1}H NMR (500 MHz, DMSO-*d₆*) δ 7.30 (m, Ar-H, 2 H), 7.27 (m, Ar-H, 2 H), 7.21 (m, Ar-H, 2 H), 7.18 (m, Ar-H, 1 H), 7.17 (m, Ar-H, 1 H), 7.16 (m, Ar-H, 2 H), 2.68 (dd, *J* = 9.0, 5.9 Hz, CH, 1 H), 2.56 (m, CH, 1 H), 2.49 (dd, *J* = 9.0, 5.9 Hz, CH, 1 H), 1.48 (brs, CH_3, 3 H), 1.41 (d, *J* = 1.3 Hz, CH_3, 3 H), 1.13 (s, CH_3, 9 H). ^{13}C{1H} NMR (125 MHz, DMSO-d_6) δ 175.5, 137.2, 128.4, 127.7, 127.1, 126.0, 126.0, 125.8, 122.3, 38.3, 33.8, 31.5, 29.6, 26.9, 18.3, 17.1. HRMS calcd for ($C_{16}H_{27}O_2$) $[M + H]^+$ 349.2162, found 349.2164.

[cyclohexylidene-[*cis*-*2*-(*2*-methoxy-*2*-oxo-ethyl)-*3*-phenyl-cyclopropyl]methyl] *2,2*-dimethyl-propanoate (3n)

Starting materials: 208 mg **1b** (1 mmol) and 220 mg **2l** (1.25 mmol). dr (cis/trans): 100/0, ee%: 64/- (Amylose-1, Heptane/IPA, 99/1, 0.5 mL/min).

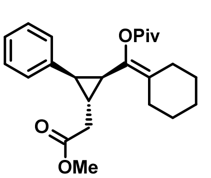

Yield (**3n**): 134 mg (0.350 mmol, 35%), yellow oil. ^{1}H NMR (500 MHz, DMSO-d_6) δ 7.28-6.97 (m, 5 H), 3.58 (s, 3 H), 2.49/2.37 (dd+dd, *J* = 16.0, 6.6 Hz, 2 H), 2.22 (dd, *J* = 8.8, 5.6 Hz, 1 H), 2.10 (m, 1 H), 1.99-0.28 (m, 10 H), 1.60 (m, 1 H), 1.19 (s, 9 H). ^{13}C{1H} NMR (125 MHz, DMSO-d_6) δ 175.5, 172.1, 138.6, 134.1, 128.9, 128.8, 127.5, 127.1, 125.5, 51.3, 38.3, 37.4, 30.7, 28.1, 27.8, 26.8, 26.7, 26.2, 26.1, 25.6, 20.6. HRMS calcd for ($C_{24}H_{32}O_4Na$) $[M + Na]^+$ 407.2192, found 407.2195.

[*cis*-(*2*-acetoxy-*2*-phenyl-cyclopropyl)-cyclohexylidene-methyl] *2,2*-dimethylpropanoate (3o) and [*trans*-(*2*-acetoxy-*2*-phenyl-cyclopropyl)-cyclohexylidene-methyl] *2,2*-dimethylpropanoate (4o)

Starting materials: 168 mg **1a** (1 mmol) and 202 mg **2m** (1.25 mmol). dr (cis/trans): 67/33, ee%: 68/70 (Amylose-1, Heptane/IPA, 99/1, 0.5 mL/min).

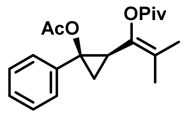

Yield (**3o**): 166 mg (0.502 mmol, 75%), yellow oil. ^{1}H NMR (500 MHz, DMSO-d_6) δ 7.35-7.2 (m, 5 H), 2.25 (m, 1 H), 2.04 (s, 3 H), 1.83/1.2 (dd+dd, *J* = 6.9, 10.2 Hz, 2 H), 1.69/1.54 (s+s, 6 H), 1.23 (s, 9 H). ^{13}C{1H} NMR (125 MHz, DMSO-d_6) δ 175.1, 169.7, 140.1, 136.3, 128.3, 126.6, 124.1, 122.4, 61.9, 38.4, 28.0, 26.9, 20.8, 18.9, 18.6/17.6. HRMS calcd for ($C_{20}H_{26}O_4Na$) $[M + Na]^+$ 353.17233, found 353.1725.

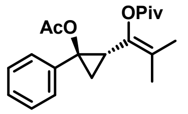

Yield (**4o**): 71 mg (0.214 mmol, 65%), yellow oil. ^{1}H NMR (500 MHz, DMSO-d_6) δ. 7.33-7.19 (m, 5 H), 2.49 (m, 1 H), 2.01 (s, 3 H), 1.61/1.31 (q+q, *J* = 4 Hz, 6 H), 1.53/1.5 (m+m, 2 H), 1.06 (s, 9 H). ^{13}C{1H} NMR (125 MHz, DMSO-d_6) δ. 175.2, 169.7, 136.8, 136.2, 127.6, 127.2, 127.0, 126.6, 122.4, 63.5, 38.2, 27.1, 26.7, 20.9, 18.3, 17.5, 17.2. HRMS calcd for ($C_{20}H_{26}O_4Na$) $[M + Na]^+$ 353.17233, found 353.1726.

[*cis*-(*2*-methyl-*1*-norcaran-7-yl-prop-*1*-enyl)] *2,2*-dimethylpropanoate (3p)

Starting materials: 168 mg **1a** (1 mmol) and 102 mg **2n** (1.25 mmol). dr (cis/trans): 100/0, ee%: 77/- (Amylose-1, Heptane/IPA, 99/1, 0.5 mL/min).

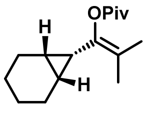

Yield (**3p**): 137 mg (0.550 mmol, 55%), yellow oil. ^{1}H NMR (500 MHz, DMSO-d_6) δ 1.78/1.46 (m+m, 4 H), 1.7/1.55 (s+s, 6 H), 1.57 (m, 1 H), 1.21 (s, 9 H), 1.16/1.1 (m+m, 4 H), 1.09 (m, 2 H). ^{13}C{1H} NMR (125 MHz, DMSO-d_6) δ 175.3, 141.6, 117.8, 38.4,

26.9, 23.2, 22.5, 20.7, 18.4, 17.4, 16.9. HRMS calcd for ($C_{16}H_{27}O_2$) $[M + H]^+$ 251.2006, found 251.2008.

[[*cis-2*-(*4*-acetylphenyl)cyclopropyl]-cyclohexylidene-methyl] *2,2*-dimethylpropanoate (3q) and [[*trans-2*-(*4*-acetylphenyl)cyclopropyl]-cyclohexylidene-methyl] *2,2*-dimethylpropanoate (4q)

Starting materials: 208 mg **1b** (1 mmol) and 182 mg **2o** (1.25 mmol). dr (cis/trans-mixture): 82/18, ee%: 80/46 (Amylose-3, Heptane/IPA, 97/3, 0.5 mL/min). Yield (**3q/4q**): 134 mg (0.430 mmol, 43%), yellow oil as diastereoisomers mixture.

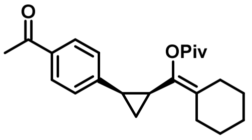

(**3q**) ^{1}H NMR (500 MHz, DMSO-d_6) δ 7.84 (m, 2 H), 7.16 (m, 2 H), 2.53 (s, 3 H), 2.41 (m, 1 H), 2.26 (m, 1 H), 2.05-0.34 (m, 10 H), 1.32/1.03 (m+m, 2 H), 1.18 (m, 9 H). ^{13}C{1H} NMR (125 MHz, DMSO-d_6) δ 197.4, 175.7, 145.6, 134.6, 134.2, 129.5, 127.4, 127.2, 38.3, 28.1, 26.9, 26.8, 26.6, 26.3, 25.6, 23.8, 22.3, 12.3. HRMS calcd for ($C_{23}H_{31}O_3$) $[M + H]^+$ 355.2282, found 355.2271.

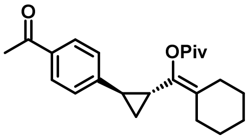

(**4q**) ^{1}H NMR (500 MHz, DMSO-d_6) δ 7.84 (m, 2 H), 7.24 (m, 2 H), 2.53 (s, 3 H), 2.27-0.92 (m, 10 H), 2.09 (m, 1 H), 2.04 (m, 1 H), 1.26/1.12 (m+m, 2 H), 1.22 (m, 9 H). ^{13}C{1H} NMR (125 MHz, DMSO-d_6) δ 197.2, 175.6, 147.9, 137.1, 134.4, 128.9, 128.3, 127.4, 125.5, 35.5, 28.4, 27.4, 26.9, 26.6, 26.4, 25.8, 23.9, 22.9, 15.5. HRMS calcd for ($C_{23}H_{31}O_3$) $[M + H]^+$ 355.2282, found 355.2269.

[*(Z)-1*-(*cis-2*-cyclopentylcyclopropyl)-*2*-phenyl-vinyl] 2,2-dimethylpropanoate (3r) and [*(Z)-1*-(*trans-2*-cyclopentylcyclopropyl)-*2*-phenyl-vinyl] 2,2-dimethylpropanoate (4r)

Starting materials: 216 mg **1c** (1 mmol) and 120 mg **2p** (1.25 mmol). dr (cis/trans): 67/33, ee%: 4/7 (Cellulose-5, Heptane/IPA, 99/1, 0.5 mL/min).

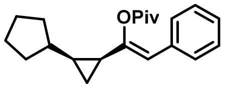

Yield (**3r**): 135 mg (0.435 mmol, 65%), yellow oil. ^{1}H NMR (500 MHz, DMSO-d_6) δ 7.37-7.16 (m, 5 H), 6.13 (brs, 1 H), 1.89/1.25 (m, 8 H), 1.73 (m, 1 H), 1.44 (m, 1 H), 1.22 (s, 9 H), 0.91 (m, 1 H), 0.88/0.59 (m+m, 2 H). ^{13}C{1H} NMR (125 MHz, DMSO-d_6) δ. 175.1, 148.6, 134.1, 128.2, 128.1, 126.9, 116.2, 39.4, 32.6, 31.9, 26.8, 24.9, 24.7, 24.6, 19.8, 9.3. HRMS calcd for ($C_{21}H_{29}O_2$) $[M + H]^+$ 313.2176, found 313.2171.

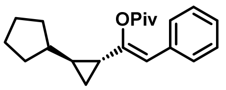

Yield (**4r**): 54 mg (0.175 mmol, 53%), yellow oil. ^{1}H NMR (500 MHz, DMSO-d_6) δ. 7.36-7.15 (m, 5 H), 6.11 (s, 1 H), 1.76-1.1 (m, 8 H), 1.46 (m, 1 H), 1.38 (m, 1 H), 1.21 (s, 9 H), 0.91 (m, 1 H), 0.82/0.62 (m+m, 2 H). ^{13}C{1H} NMR (125 MHz, DMSO-d_6) δ. 174.9, 149.9, 134.2, 128.2, 127.9, 126.7, 113.8, 43.4, 39.4, 31.4, 31.3, 26.8, 24.6, 24.5, 21.0, 11.1. HRMS calcd for ($C_{21}H_{29}O_2$) $[M + H]^+$ 313.2176, found 313.2163.

***Tert*-butyl *3*-[*(Z)-2*-acetoxy-*2*-[*cis-2*-(*3*-bromophenyl)cyclopropyl]vinyl]benzoate (3s) and *Tert*-butyl *3*-[*(Z)-2*-acetoxy-*2*-[*trans-2*-(*3*-bromophenyl)cyclopropyl]vinyl]benzoate (4s)**

Starting materials: 274 mg **1d** (1 mmol) and 228 mg **2f** (1.25 mmol). dr (cis/trans-mixture): 65/34, ee%: 0/0 (Amylose-3, Heptane/IPA, 9/1, 0.5 mL/min). Yield (**3s/4s**): 251 mg (0.550 mmol, 55%), yellow oil.

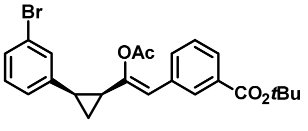

(**3s**): ^{1}H NMR (500 MHz, DMSO-d_6) δ 7.94-7.02 (m, 6 H), 7.44 (t, J = 7.4, 1 H), 7.24 (dm, 1 H), 6.21 (brs, 1 H), 2.46 (m, 1 H), 2.26 (m, 1 H), 2.06 (s, 3 H), 1.58/1.34 (m, 2 H), 1.51 (s, 9 H). ^{13}C{1H} NMR (125 MHz, DMSO-d_6) δ. 168.0, 164.7, 146.8, 140.8, 134.1, 132.3, 131.4, 130.9, 129.6, 128.7, 128.0, 127.5, 121.1, 116.5, 80.7, 54.8, 27.7, 24.2, 23.2, 20.7, 9.9. HRMS calcd for ($C_{24}H_{25}BrO_4Na$) $[M + Na]^+$ 479.0828, found 479.0829.

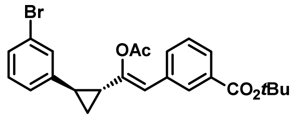

(**4s**): ^{1}H NMR (500 MHz, DMSO-d_6) δ 7.94-7.01 (m, 6 H), 7.23 (t, J = 7.4, 1 H), 7.04 (dm, 1 H), 6.3 (brs, 1 H), 2.53 (m, 1 H), 2.5 (m, 1 H), 2.09 (s, 3 H), 1.57 (s, 9 H), 1.36/1.32 (m+m, 2 H). ^{13}C{1H} NMR (125 MHz, DMSO-d_6) δ 168.6, 164.7, 147.1, 140.5, 134.3, 132.7, 131.2, 130.5, 128.6, 128.5, 127.4, 126.2, 121.0, 80.7, 54.8, 27.7, 24.4, 22.7, 20.5, 11.3. HRMS calcd for ($C_{24}H_{25}BrO_4Na$) [M + Na]$^+$ 479.0828, found 479.0827.

***Tert*-butyl *3*-[*(Z)-2*-acetoxy-*2*-[*cis-2*-(*2*-iodophenyl)cyclopropyl]vinyl]benzoate (3t) and *Tert*-butyl *3*-[*(Z)-2*-acetoxy-*2*-[*trans-2*-(*2*-iodophenyl)cyclopropyl]vinyl]benzoate (4t)**

Starting materials: 274 mg **1d** (1 mmol) and 228 mg **2q**(1.25 mmol). dr (cis/trans-mixture): 78/22, ee%: 6/28 (Amylose-3, Heptane/IPA, 4/1, 0.5 mL/min). Yield (**3t/4t**): 126 mg (0.250 mmol, 25%), yellow oil.

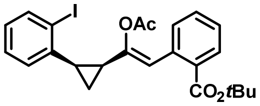

(**3t**): ^{1}H NMR (500 MHz, DMSO-d_6) δ 7.99-6.89 (m, 7 H), 7.19 (dm, 1 H), 6.14 (brs, 1 H), 2.38 (m, 1 H), 2.37 (m, 1 H), 2.06 (s, 3 H), 1.63/1.36 (m+m, 2 H), 1.51 (s, 9 H). ^{13}C{1H} NMR (125 MHz, DMSO-d_6) δ. 168.0, 164.6, 147.2, 140.0, 138.4, 134.2, 132.1, 131.3, 130.4, 128.8, 128.7, 128.1, 127.9, 127.5, 127.4, 116.1, 103.8, 80.7, 29.8, 27.7, 23.6, 20.9, 9.8. HRMS calcd for ($C_{24}H_{25}IO_4Na$) [M + Na]$^+$ 527.0689, found 527.0690.

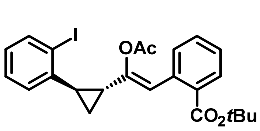

(**4t**): ^{1}H NMR (500 MHz, DMSO-d_6) δ. 7.99-6.88 (m, 7 H), 7.09 (dm, 1 H), 6.28 (brs, 1 H), 2.57 (m, 1 H), 2.48 (m, 1 H), 1.85 (s, 3 H), 1.55 (s, 9 H), 1.38/1.27 (m+m, 2 H). ^{13}C{1H} NMR (125 MHz, DMSO-d_6) δ. 168.3, 164.7, 147.6, 140.3, 138.5, 134.5, 132.9, 131.4, 129.2, 128.6, 128.1, 127.7, 127.4, 120.2, 103.2, 80.8, 30.1, 27.8, 21.4, 20.6, 11.9. HRMS calcd for ($C_{24}H_{25}IO_4Na$) [M + Na]$^+$ 527.0689, found 527.0693.

9. Comparison of the catalytic activity and selectivity of selected gold(I)-carbene complexes in the reaction of 1a and 2a under the original and optimised conditions

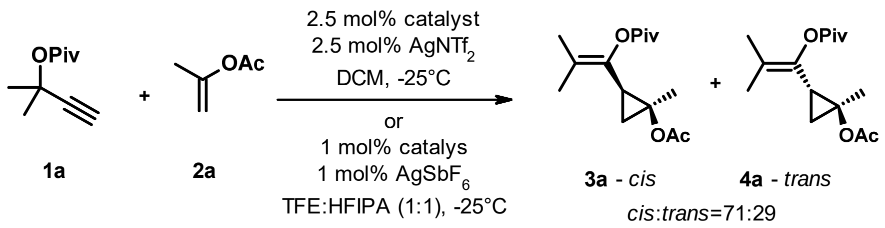

complex	original conditions			optimal conditions		
	**3a** *ee* (%)	**4a** *ee* (%)	Time (d)[1]	**3a** *ee* (%)	**4a** *ee* (%)	Time (d)[1]
**C3**	36[2]	32	6	22[2]	24	2
**C4**	14	14[2]	8	20	10[2]	1
**C15**	74[2]	64	10	84[2]	82	2
**C16**	44	42[2]	9	60	60[2]	2
**C21**	30[2]	28	10	42[2]	32	6
**C22**	52	48[2]	10	66	54[2]	10
**C25**	70[2]	64	8	82[2]	76	2
**C26**	36	42[2]	9	70	68[2]	2
**C31**	58[2]	56	7	82[2]	78	2
**C32**	62	58[2]	7	70	52[2]	2

[1] Time needed to reach full conversion. In reaction incomplete then conversion value is indicated in parenthesis

[2] The enantiomer eluting second under the applied chromatographic conditions is the major one.

10. References for Known Compound

1. Szabo, Z.; Timari, M.; Kassai, R.; Szolok, B.; Benyei, C. A.; Gáti, T.; Paczal, A.; Kotschy, A. Modular Synthesis of Chiral NHC Precursors and Their Silver and Gold Complexes. *Organometallics* **2020**, *39*, 3572−3589.
2. Szabo, Z.; Paczal, A.; Kovács, T.; Mándi, A.; Kotschy, A.; Kurtán, T. Synthesis and Vibrational Circular Dichroism Analysis of N-Heterocyclic Carbene Precursors Containing Remote Chirality Centers. *Int. J. Mol. Sci.* **2022**, *23*, 3471.
3. Paczal, A.; Bényei, A. C.; Kotschy, A. Modular synthesis of heterocyclic carbene precursors. *J. Org. Chem.* **2006**, *71*, 16, 5969–5979.

11.NMR Spectra (^{1}H and ^{13}C{1H})

Some of the complexes cotain their diastereoismer as a minor component. Signals belonging to these minor components are labelled with an asterisk(*).

(4*S*)-4-*terc*-Butyl-3-(2',6'-diisopropylphenyl)-1-[(1*R*)-2'-naphtylethyl]imidazolidin-2-ylidene-silver-chloride (B6)

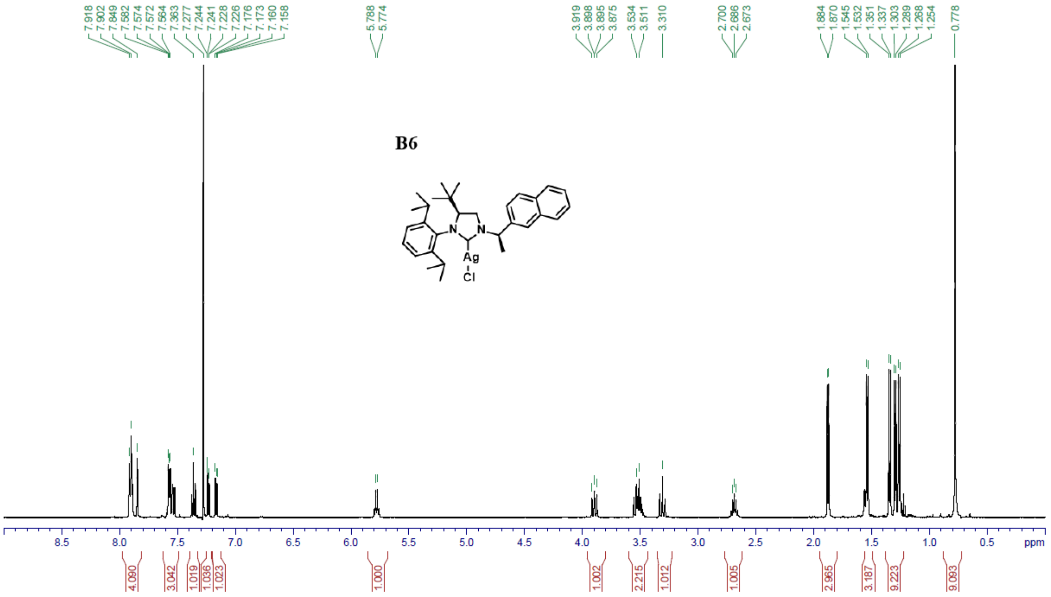

Figure S1. ^{1}H NMR (500 MHz) spectrum of **B6** in $CDCl_3$

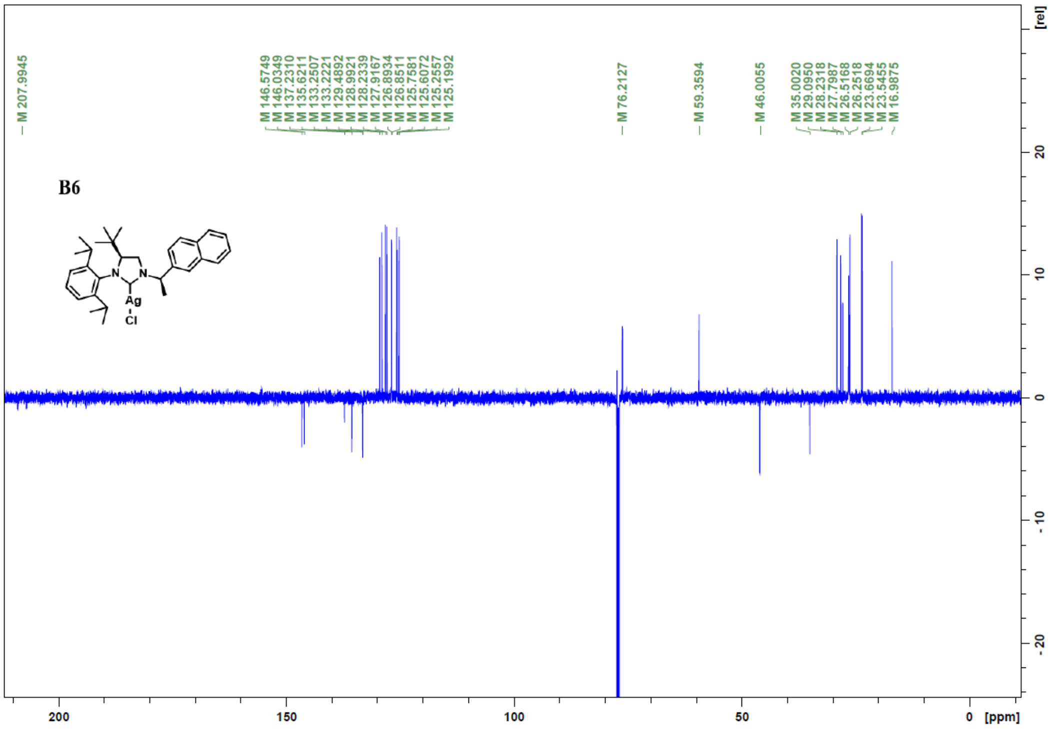

Figure S2. ^{13}C{1H} NMR (125 MHz) spectrum of **B6** in $CDCl_3$

(4*S*)-4-*terc*-Butyl-3-(2',6'-diisopropylphenyl)-1-[(1*R*)-1-(4-trifluoromethyl)-2-phenylethyl]imidazolidin-2-ylidene-silver-chloride (B15)

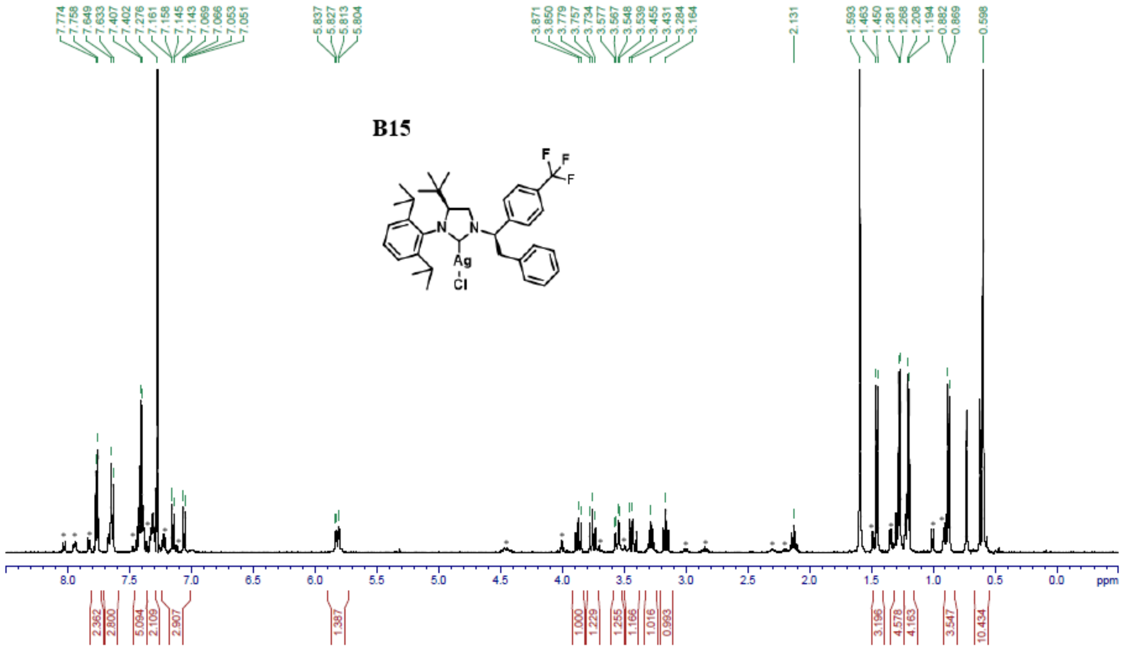

Figure S3. ^{1}H NMR (500 MHz) spectrum of **B15** in $CDCl_3$

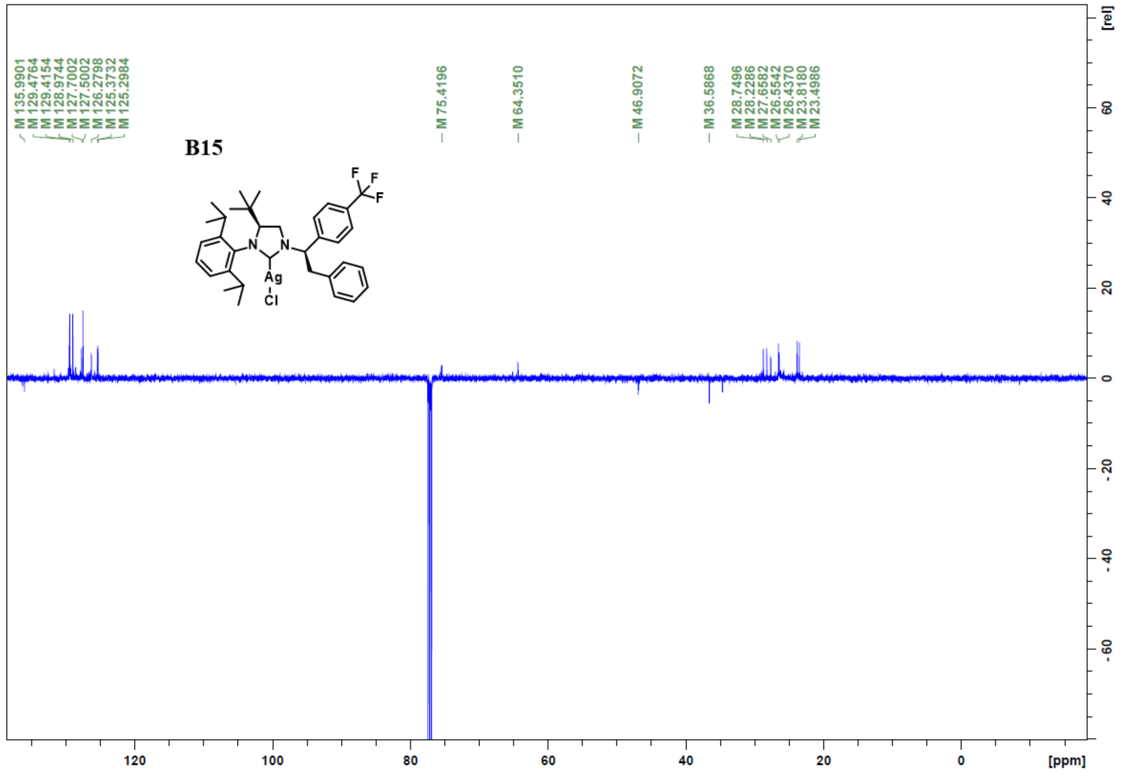

Figure S4. $^{13}C\{1H\}$ NMR (125 MHz) spectrum of **B15** in $CDCl_3$

(*4S*)-*4*-*terc*-Butyl-*3*-(*2'*,*6'*-diisopropylphenyl)-*1*-[(*1S*)-*1*-(*4*-trifluoromethyl)-*2*-phenylethyl]imidazolidin-2-ylidene-silver-chloride (B16)

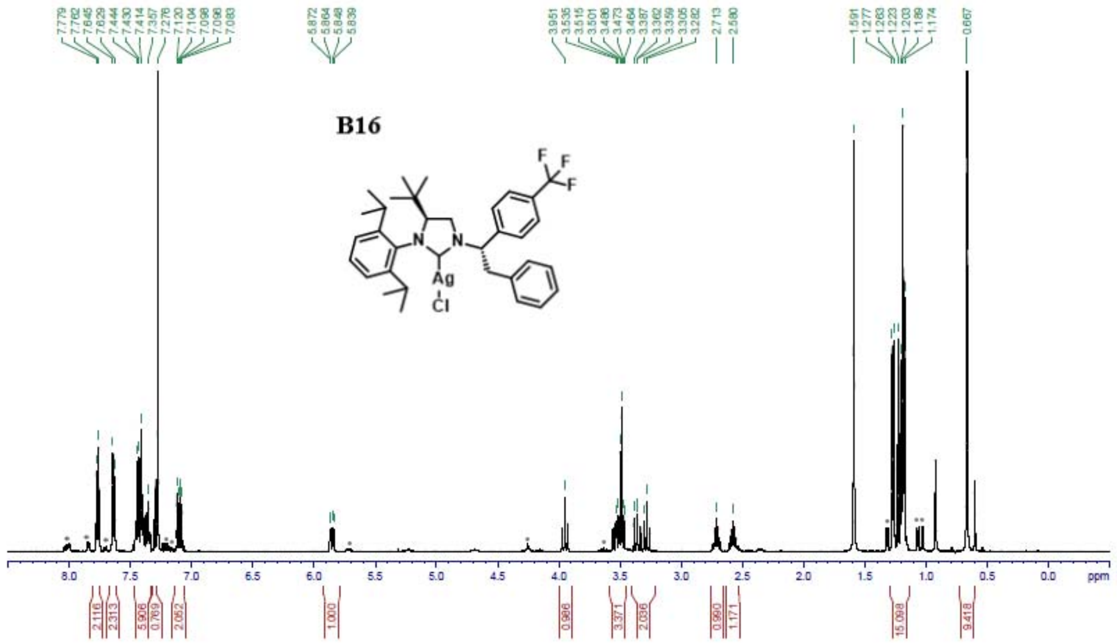

Figure S5. ^{1}H NMR (500 MHz) spectrum of **B16** in $CDCl_3$

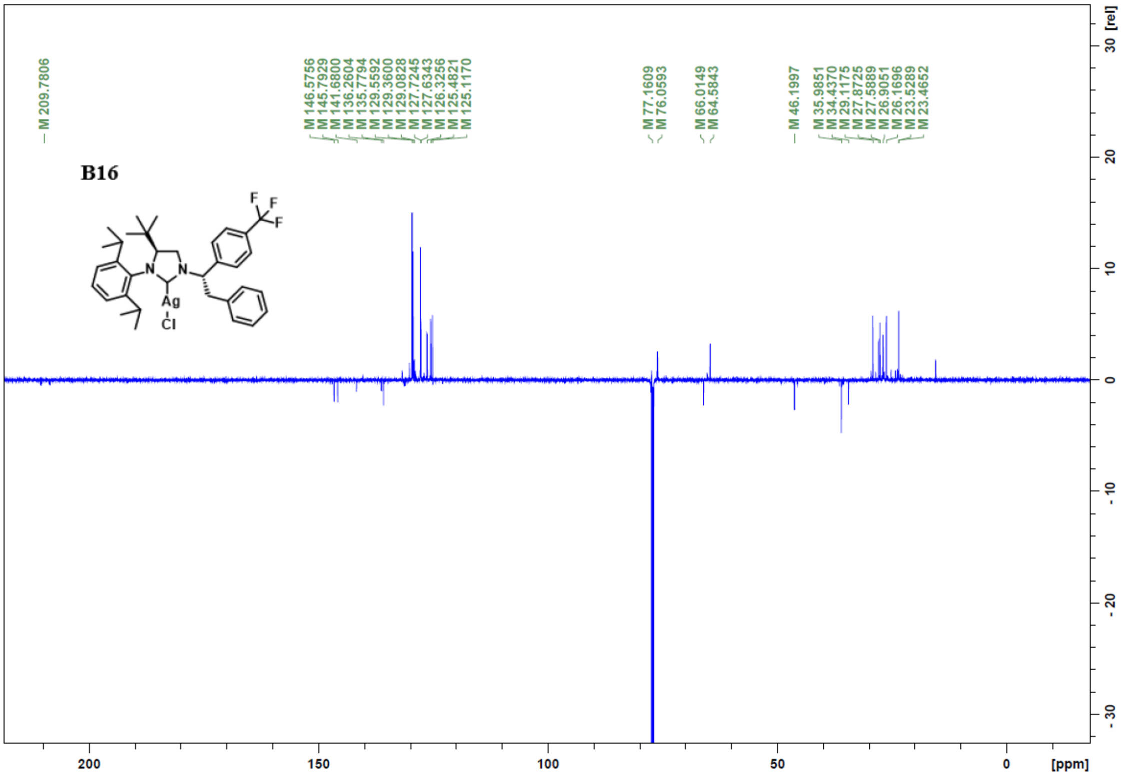

Figure S6. $^{13}C\{1H\}$ NMR (125 MHz) spectrum of **B16** in $CDCl_3$

(4S)-4-terc-Butyl-3-(2’,6’-diisopropylphenyl)-1-[(1R)-1-(4-methoxyphenyl)-2-phenylethyl]imidazolidin-2-ylidene-silver-chloride (B17)

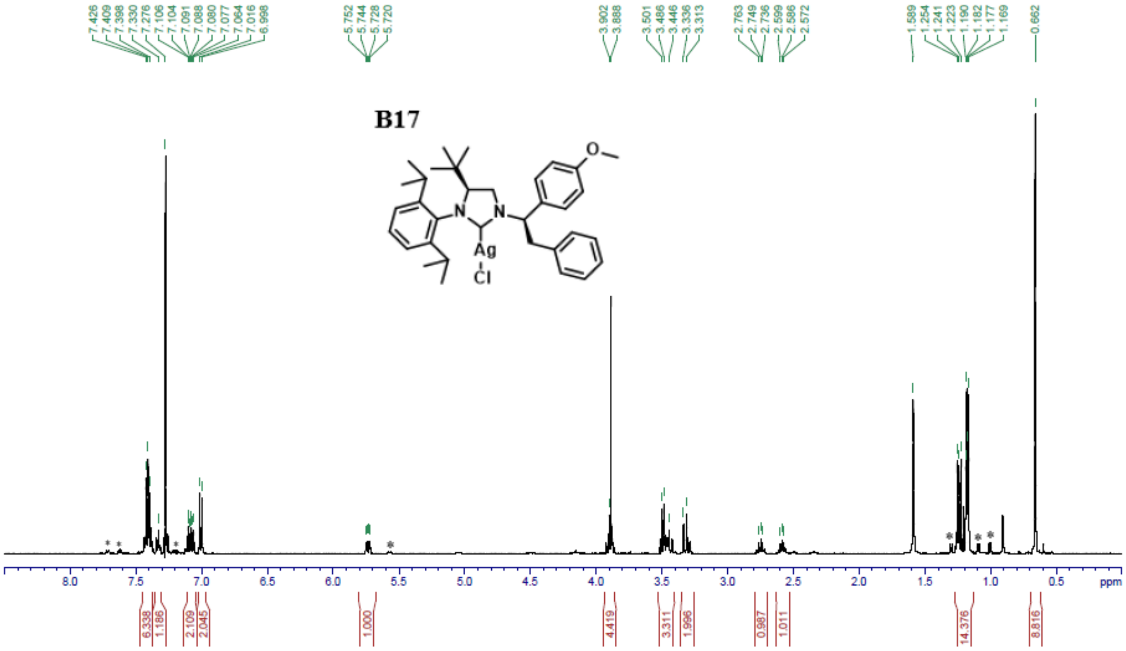

Figure S7. ^{1}H NMR (500 MHz) spectrum of **B17** in $CDCl_3$

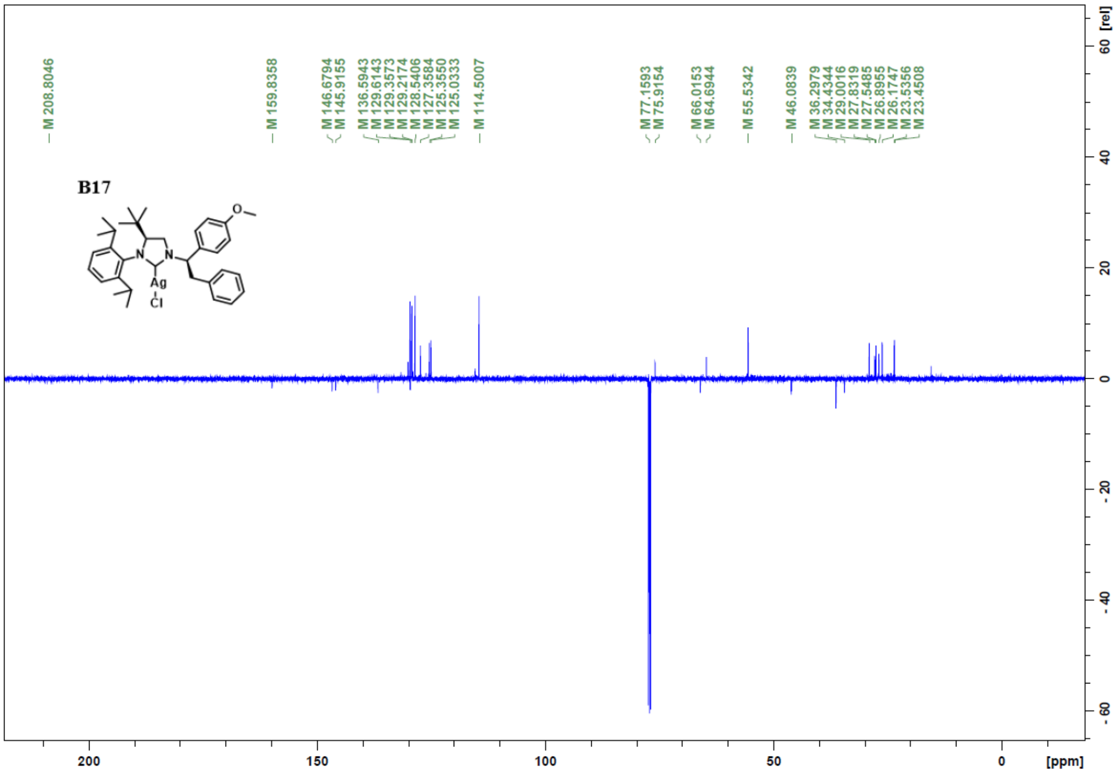

Figure S8. ^{13}C{1H} NMR (125 MHz) spectrum of **B17** in $CDCl_3$

(4*S*)-4-*terc*-Butyl-3-(2',6'-diisopropylphenyl)-1-[(1*S*)-1-(4-methoxyphenyl)-2-phenylethyl]imidazolidin-2-ylidene-silver-chloride (B18)

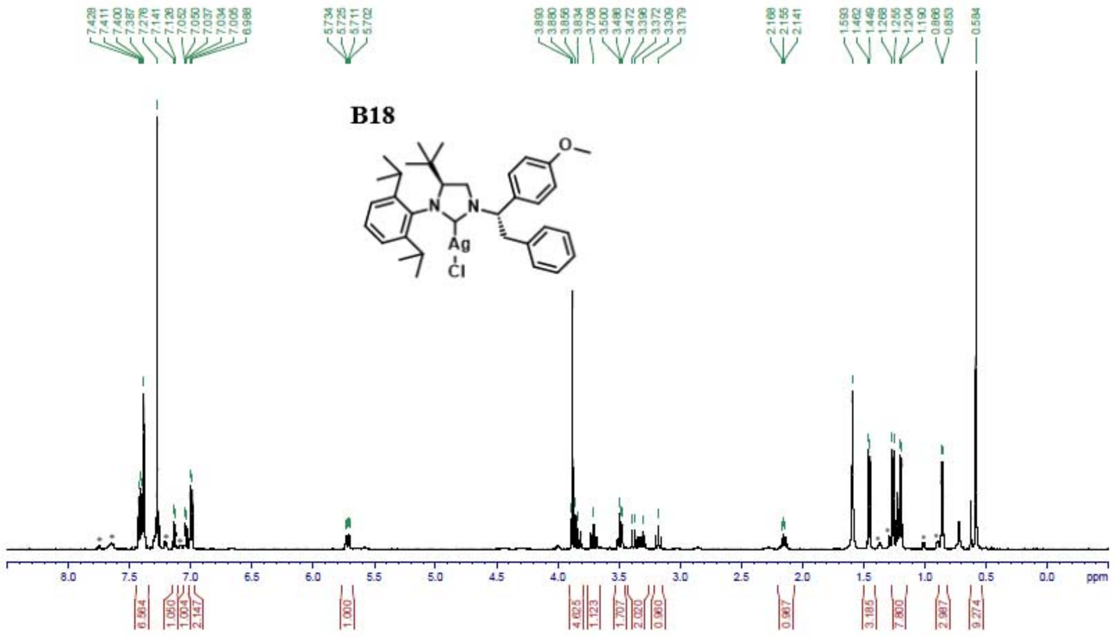

Figure S9. ^{1}H NMR (500 MHz) spectrum of **B18** in $CDCl_3$

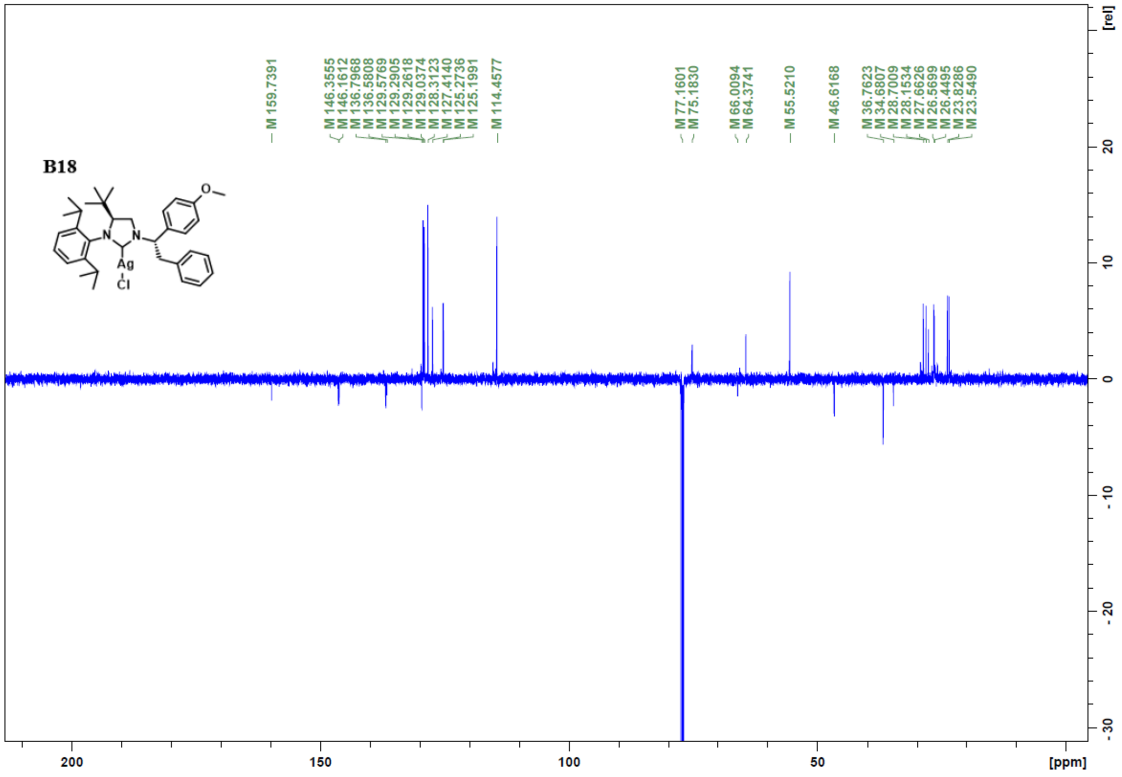

Figure S10. $^{13}C\{1H\}$ NMR (125 MHz) spectrum of **B18** in $CDCl_3$

(4*S*)-4-*terc*-Butyl-3-(2',6'-diisopropylphenyl)-1-[(1*R*)-1-(*p*-tolyl)-2-phenylethyl]imidazolidin-2-ylidene-silver-chloride (B19)

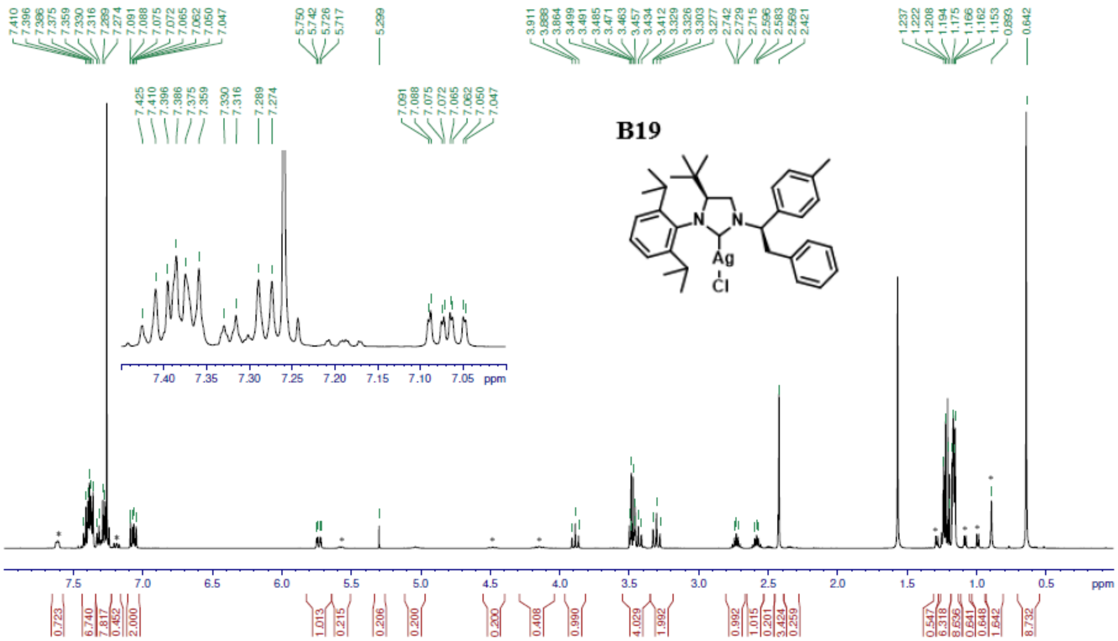

Figure S11. ^{1}H NMR (500 MHz) spectrum of **B19** in $CDCl_3$

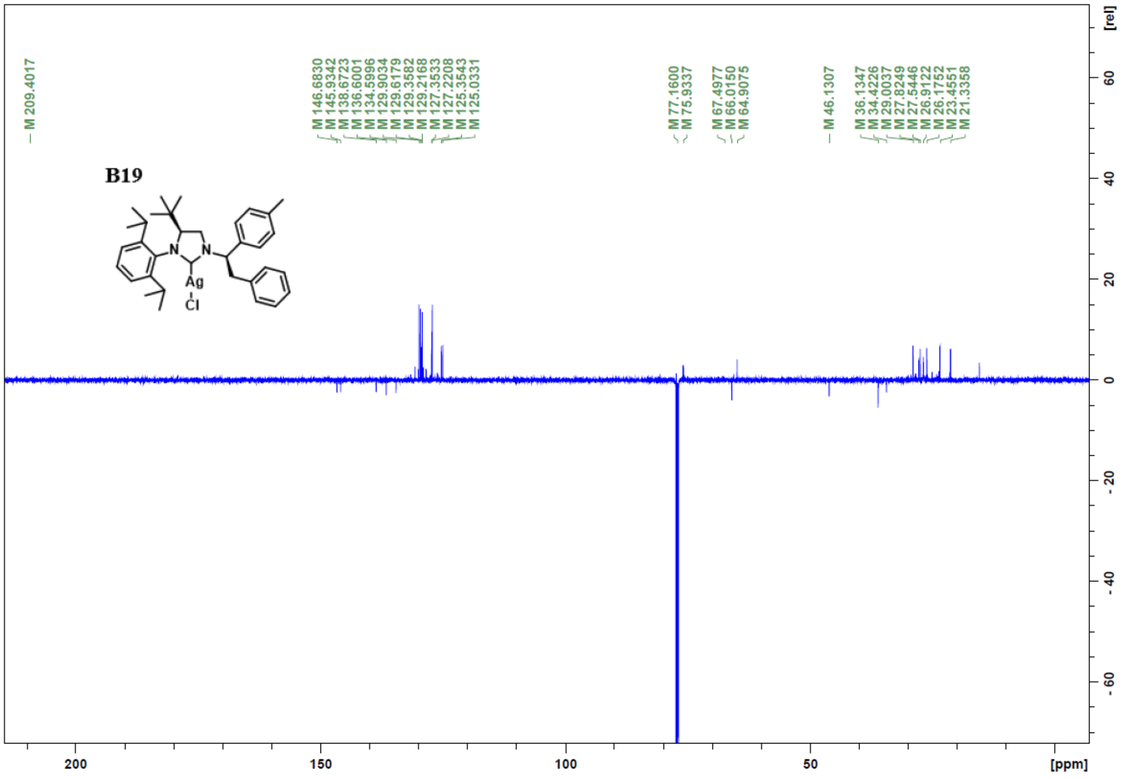

Figure S12. ^{13}C{1H} NMR (125 MHz) spectrum of **B19** in $CDCl_3$

(4*S*)-*4-terc*-Butyl-*3*-(*2',6'*-diisopropylphenyl)-*1*-[(*1S*)-*1*-(*p*-tolyl)-*2*-phenylethyl]imidazolidin-2-ylidene-silver-chloride (B20)

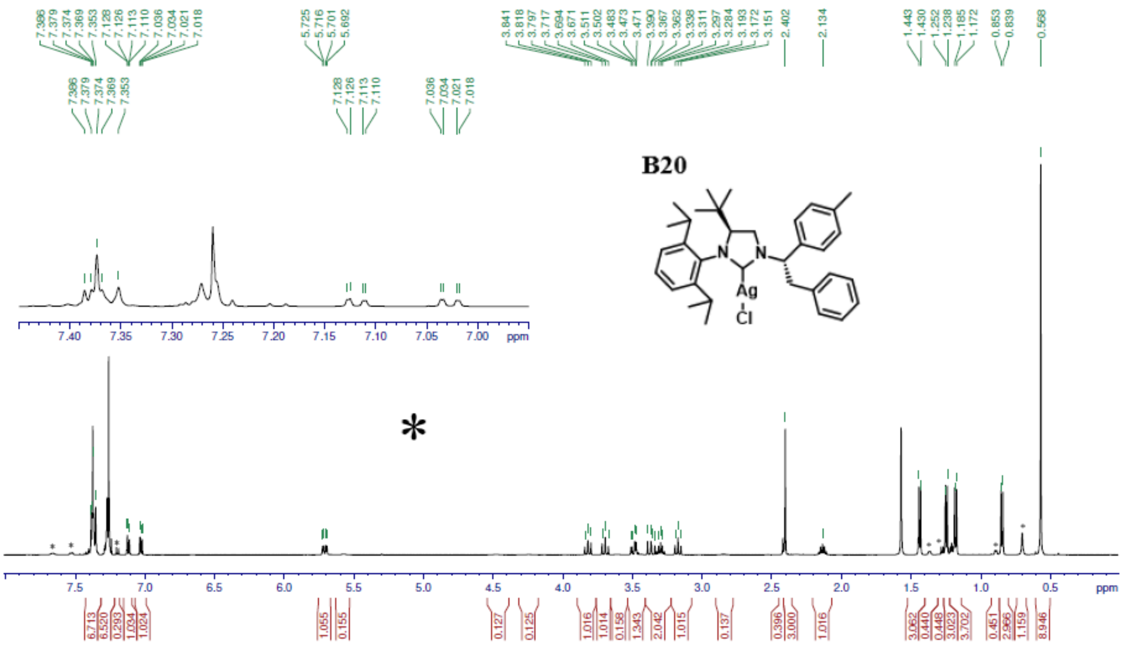

Figure S13. ^{1}H NMR (500 MHz) spectrum of **B20** in $CDCl_3$

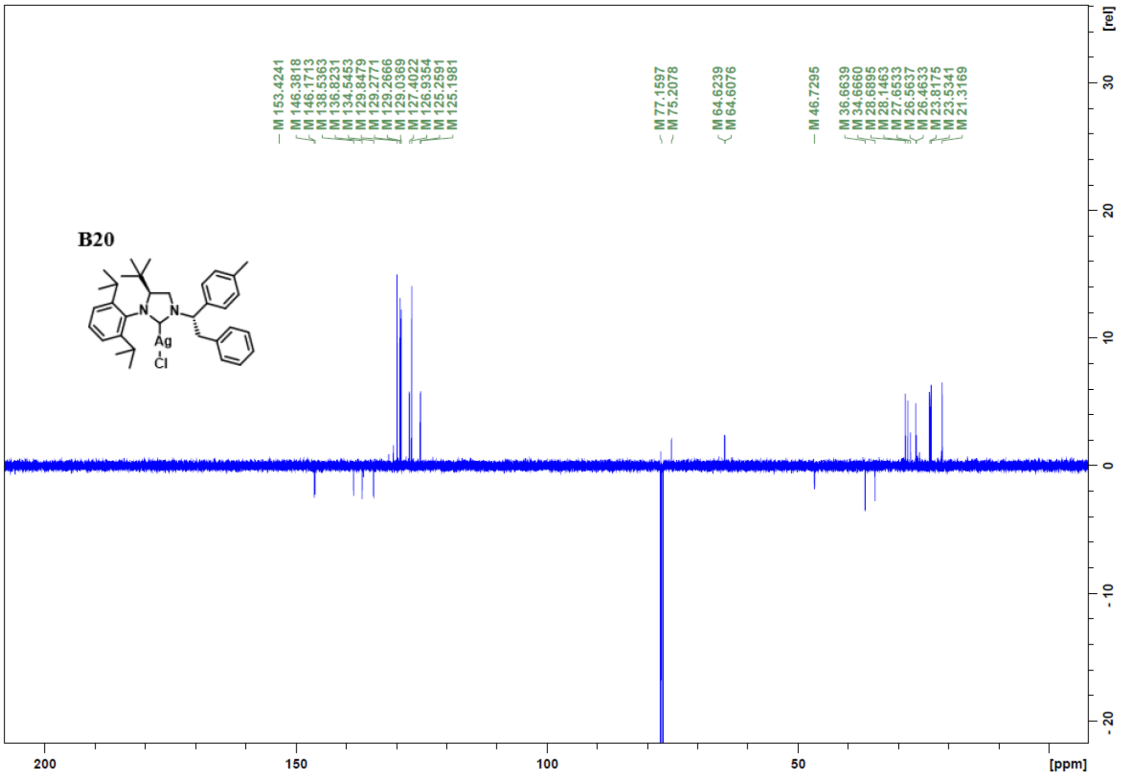

Figure S14. $^{13}C\{1H\}$ NMR (125 MHz) spectrum of **B20** in $CDCl_3$

(4*S*)-4-*terc*-Butyl-3-(2',6'-diisopropylphenyl)-1-[(1*R*)-1-(2-methoxyphenyl)-2-phenylethyl]imidazolidin-2-ylidene-silver-chloride (B21)

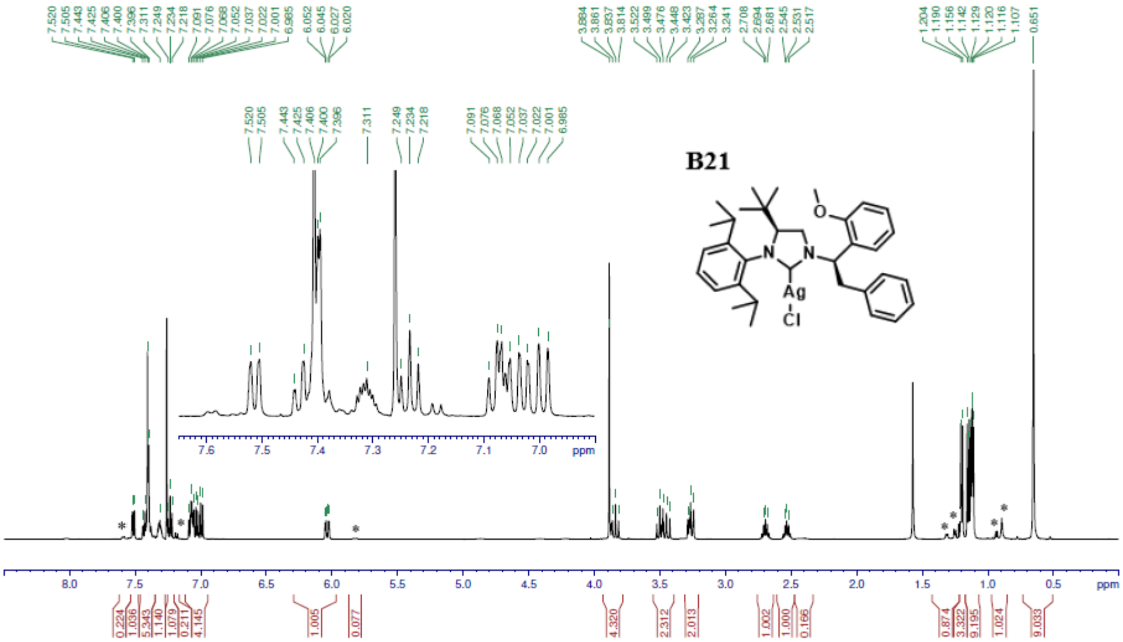

Figure S15. ^{1}H NMR (500 MHz) spectrum of **B21** in $CDCl_3$

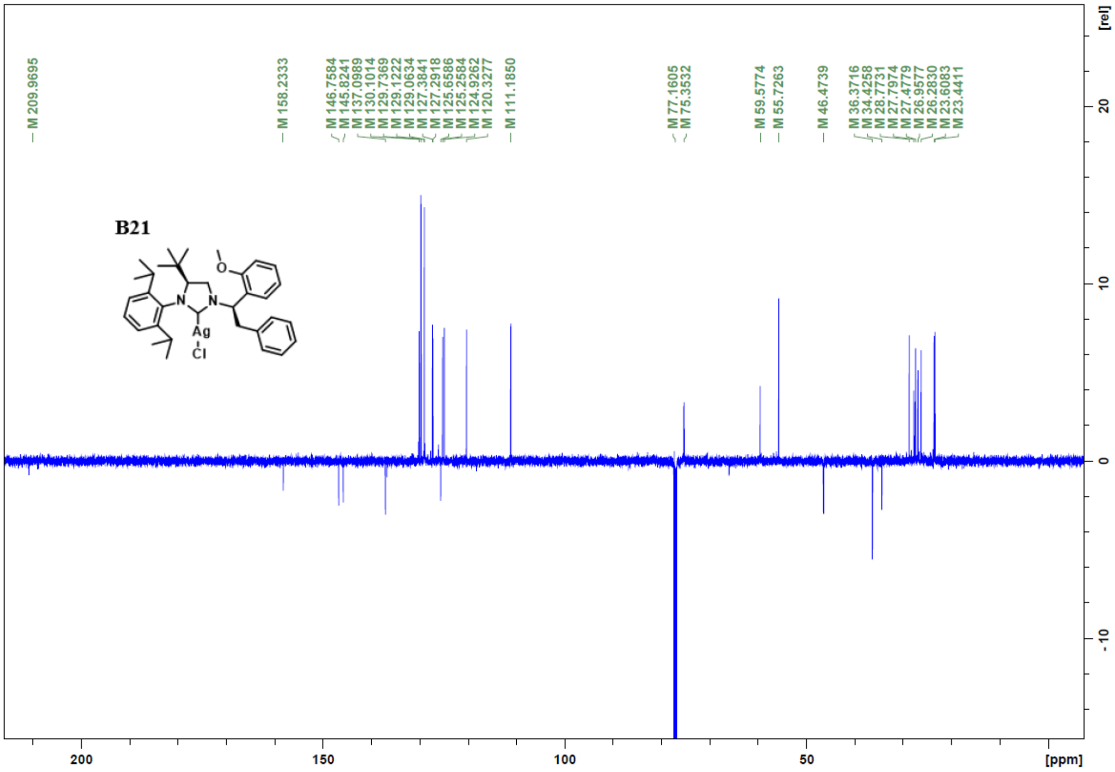

Figure S16. $^{13}C\{1H\}$ NMR (125 MHz) spectrum of **B21** in $CDCl_3$

(4*S*)-4-*terc*-Butyl-3-(2',6'-diisopropylphenyl)-1-[(1*S*)-1-(2-methoxyphenyl)-2-phenylethyl]imidazolidin-2-ylidene-silver-chloride (B22)

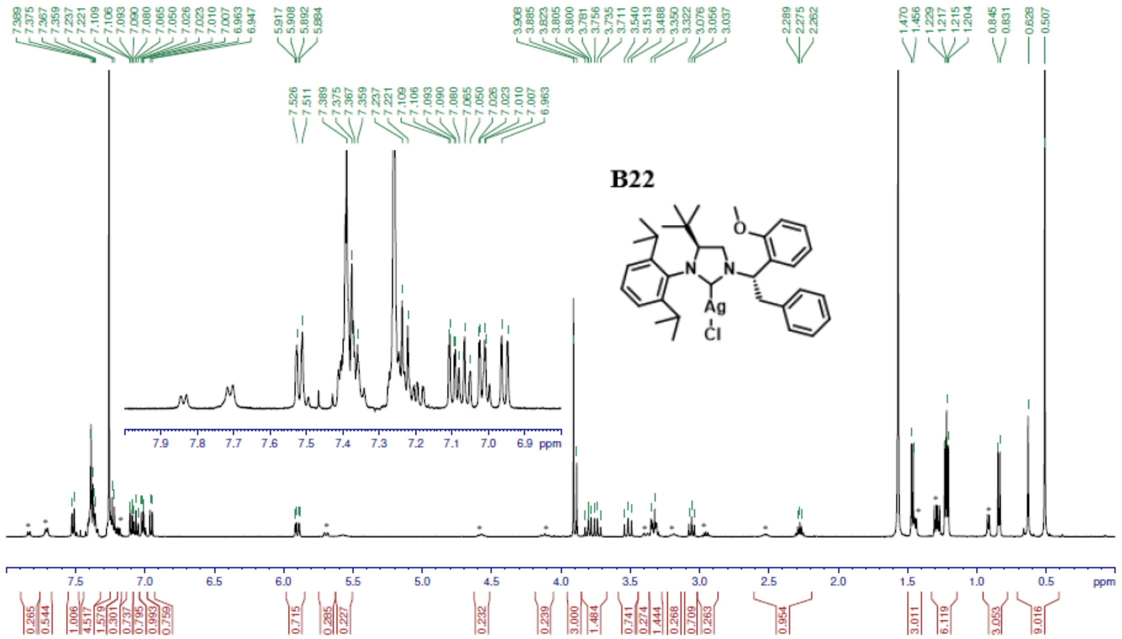

Figure S17. ^{1}H NMR (500 MHz) spectrum of **B22** in $CDCl_3$

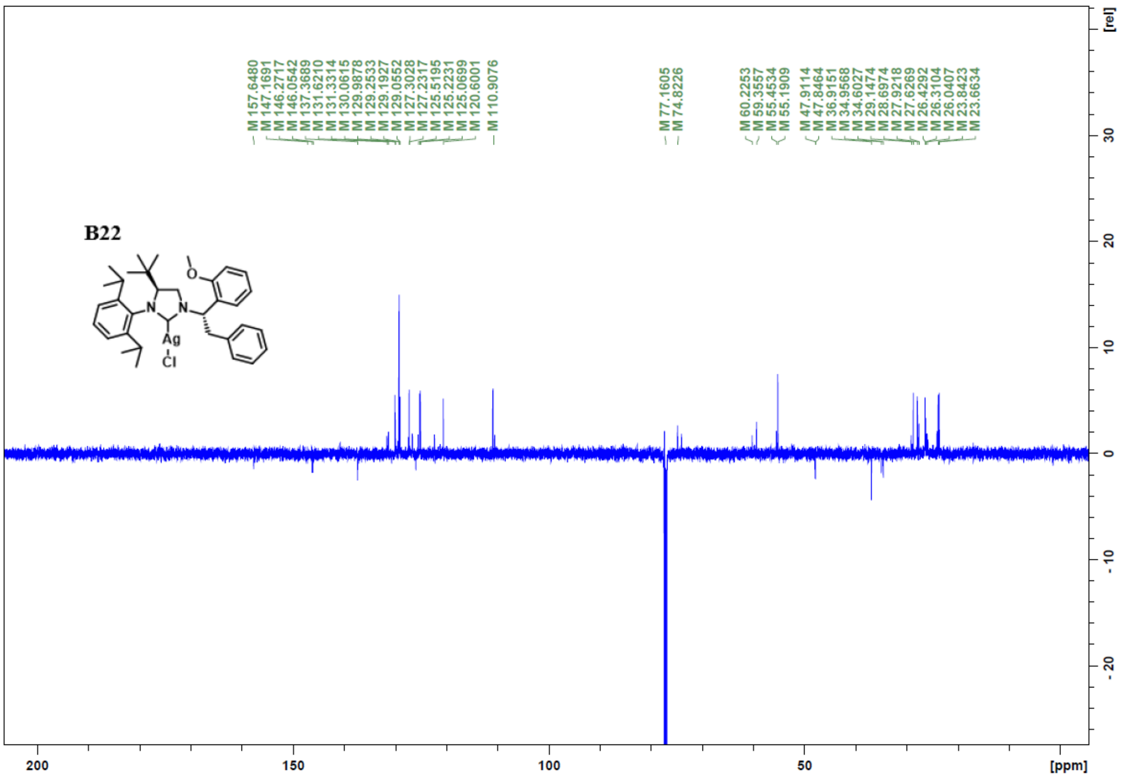

Figure S18. $^{13}C\{1H\}$ NMR (125 MHz) spectrum of **B22** in $CDCl_3$

(*4S*)-*4-terc*-Butyl-*3*-(*2',6'*-diisopropylphenyl)-*1*-[(*1R*)-*1*-(*o*-tolyl)-*2*-phenylethyl]imidazolidin-2-ylidene-silver-chloride (B23)

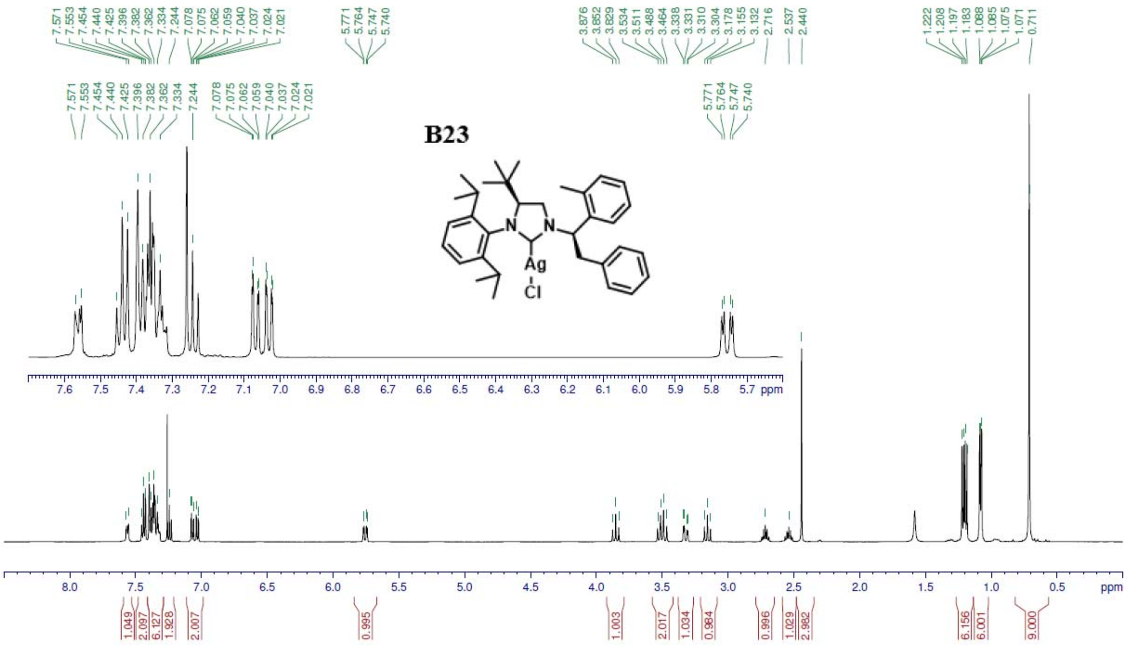

Figure S19. ^{1}H NMR (500 MHz) spectrum of **B23** in $CDCl_3$

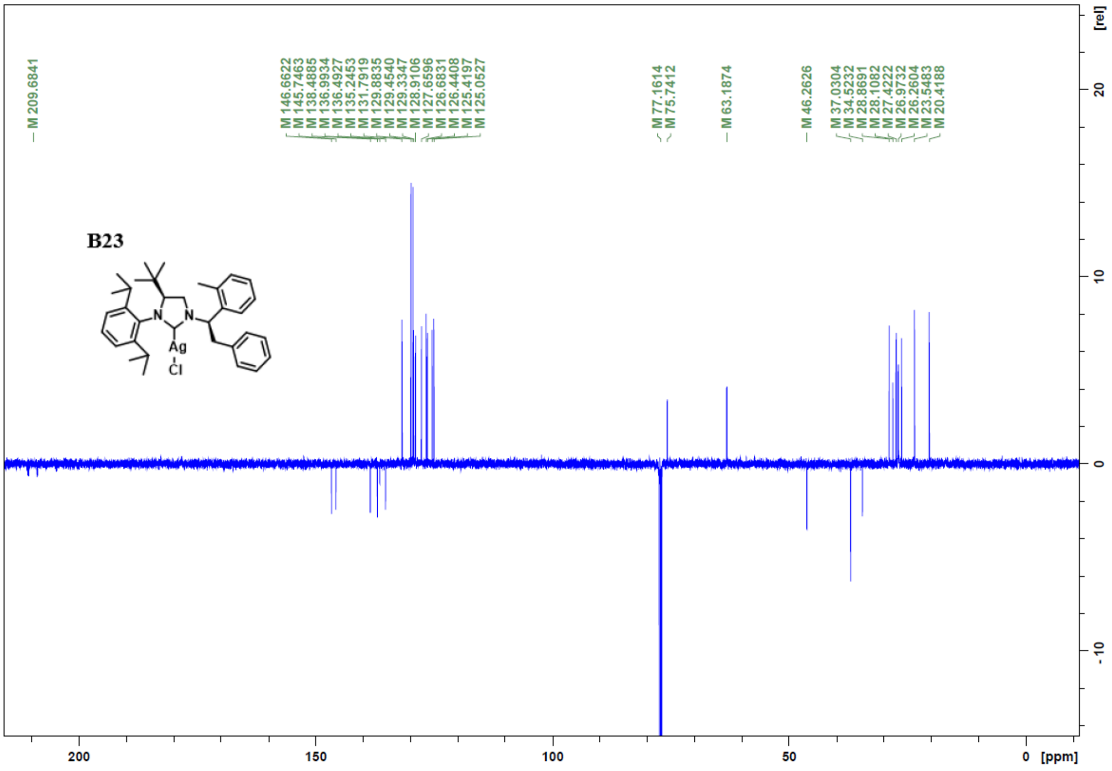

Figure S20. $^{13}C\{1H\}$ NMR (125 MHz) spectrum of **B23** in $CDCl_3$

(4*S*)-4-*terc*-Butyl-3-(2',6'-diisopropylphenyl)-1-[(1*S*)-1-(*o*-tolyl)-2-phenylethyl]imidazolidin-2-ylidene-silver-chloride (B24)

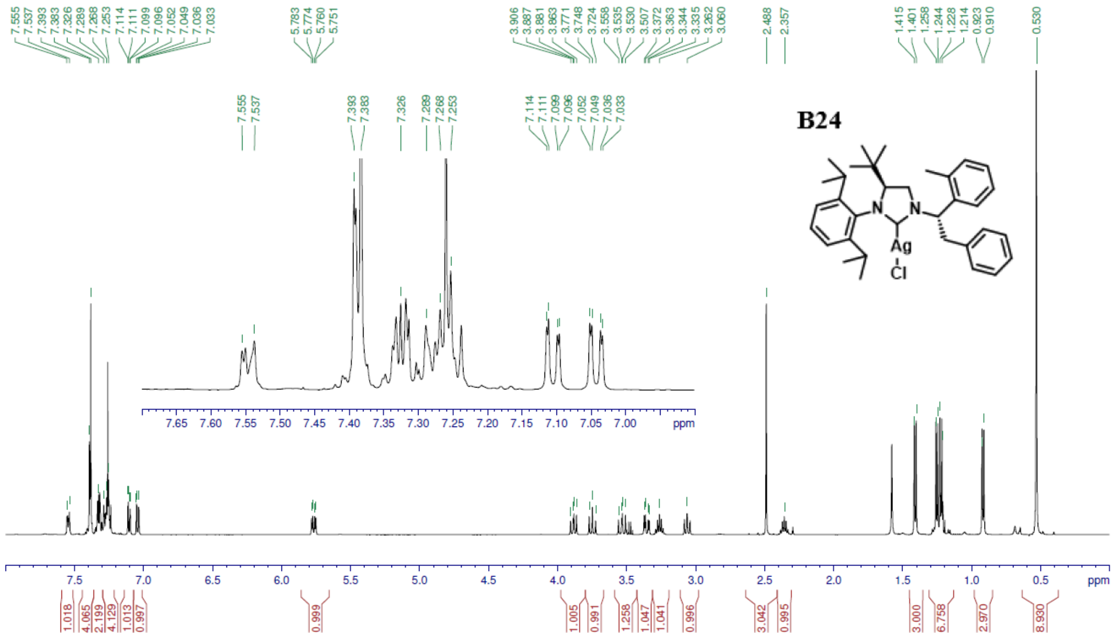

Figure S21. ^{1}H NMR (500 MHz) spectrum of **B24** in $CDCl_3$

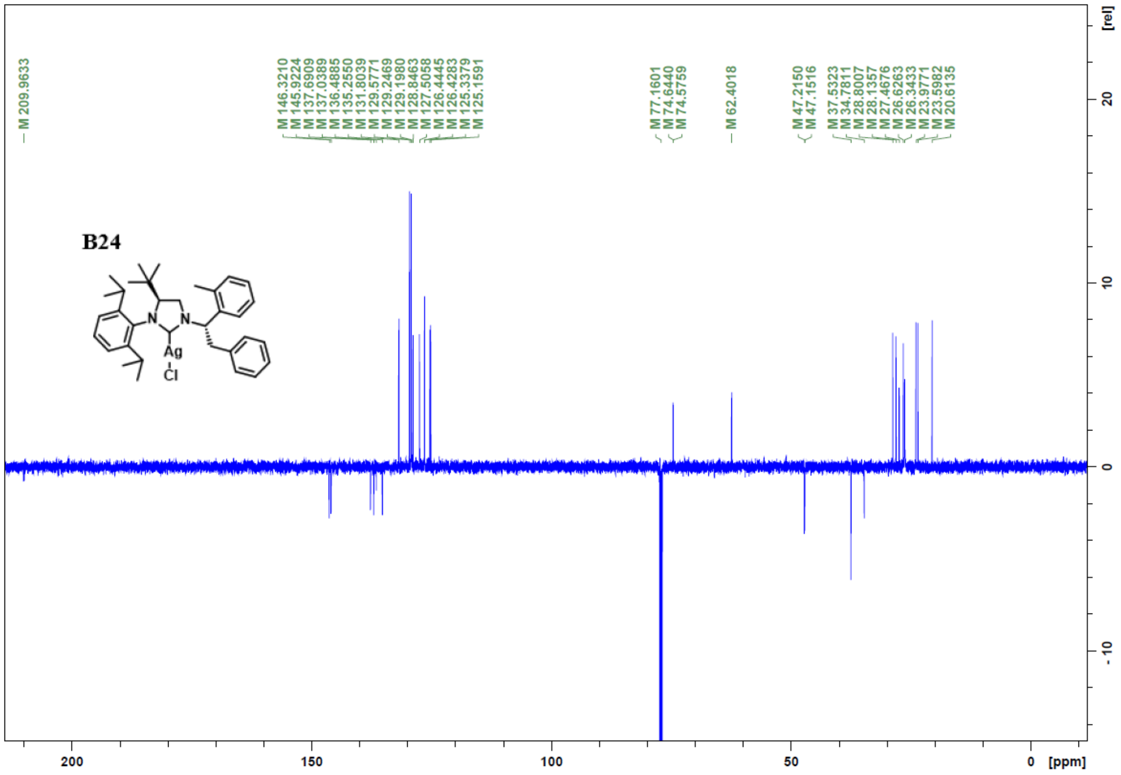

Figure S22. $^{13}C\{1H\}$ NMR (125 MHz) spectrum of **B24** in $CDCl_3$

(4S)-4-terc-Butyl-3-(2',6'-diisopropylphenyl)-1-[(1R)-1-(3-methoxyphenyl)-2-phenylethyl]imidazolidin-2-ylidene-silver-chloride (B25)

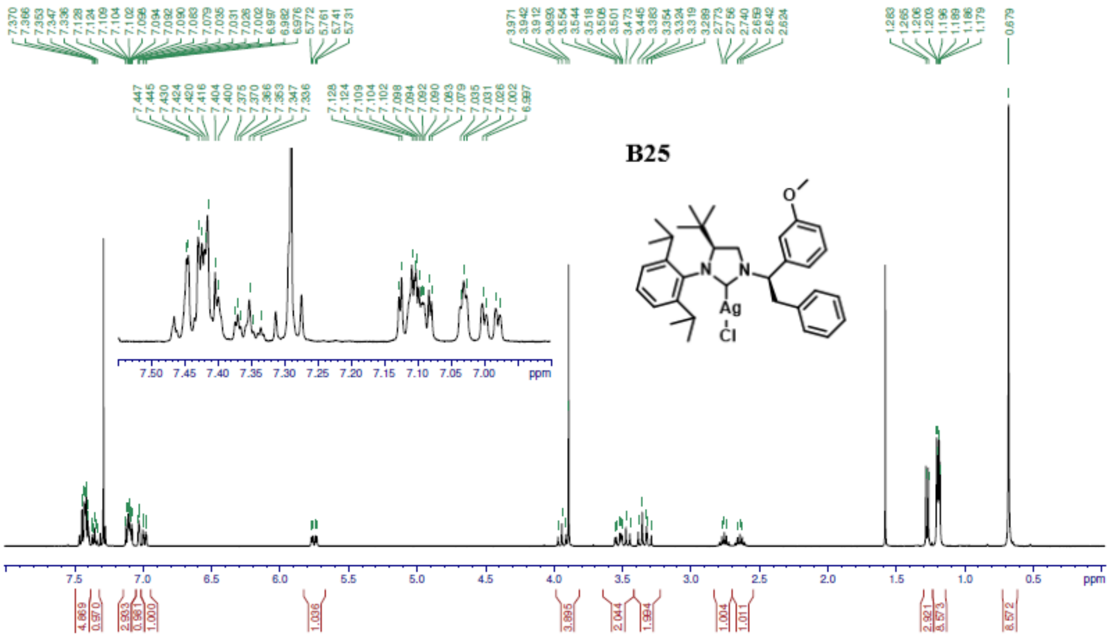

Figure S23. ^{1}H NMR (400 MHz) spectrum of **B25** in $CDCl_3$

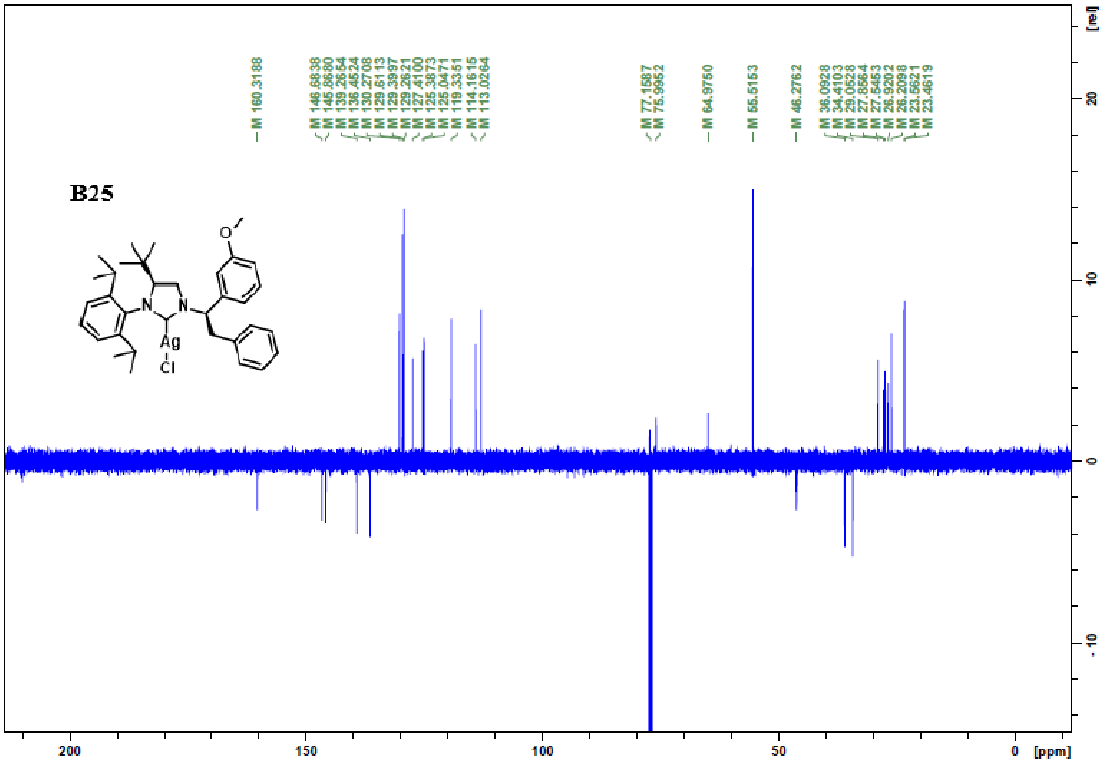

Figure S24. $^{13}C\{1H\}$ NMR (100 MHz) spectrum of **B25** in $CDCl_3$

(4*S*)-4-*terc*-Butyl-3-(2',6'-diisopropylphenyl)-1-[(1*S*)-1-(3-methoxyphenyl)-2-phenylethyl]imidazolidin-2-ylidene-silver-chloride (B26)

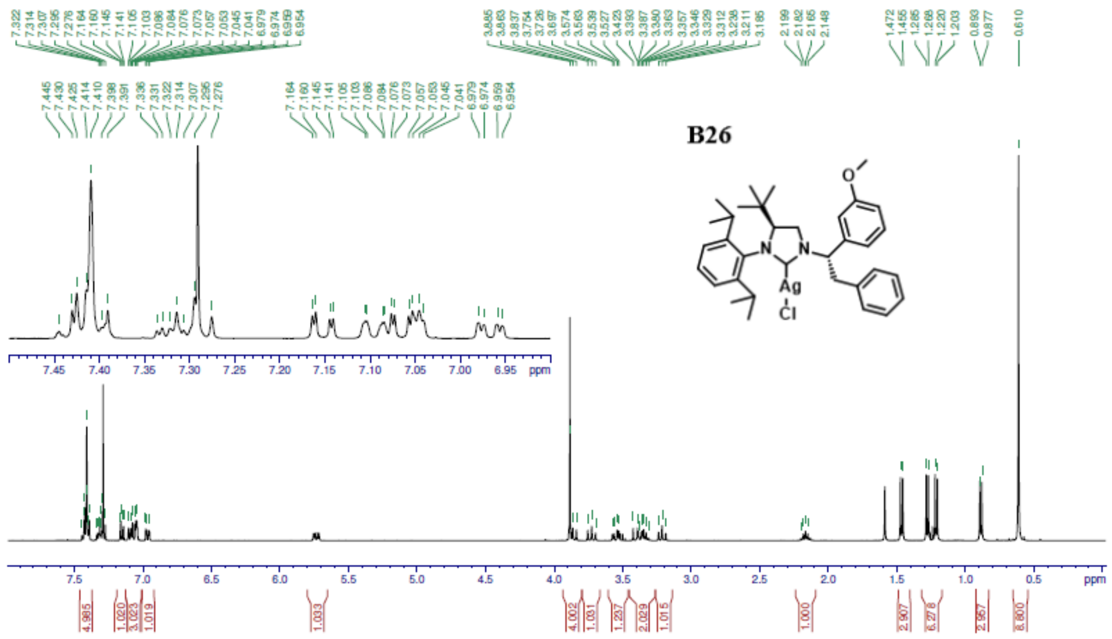

Figure S25. ^{1}H NMR (400 MHz) spectrum of **B26** in $CDCl_3$

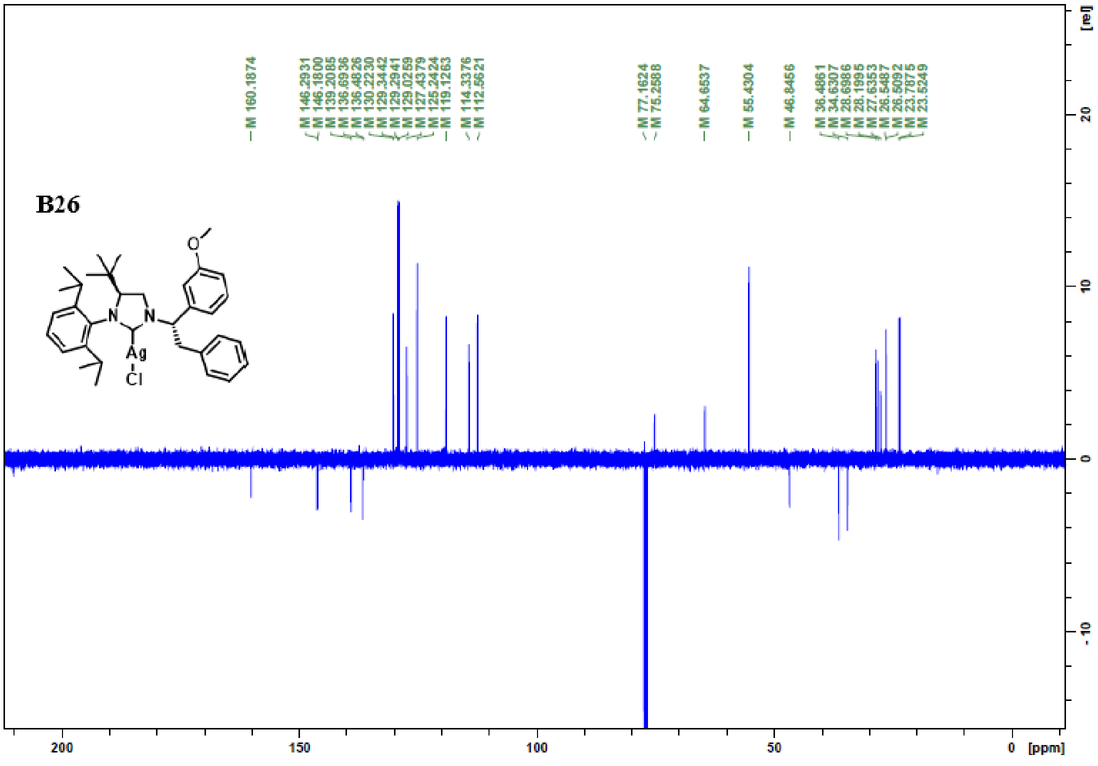

Figure S26. $^{13}C\{1H\}$ NMR (100 MHz) spectrum of **B26** in $CDCl_3$

(*4S*)-*4-terc*-Butyl-*3*-(*2',6'*-diisopropylphenyl)-*1*-[(*1R*)-*2*-(*p*-tolyl)-*1*-phenylethyl]imidazolidin-2-ylidene-silver-chloride (B27)

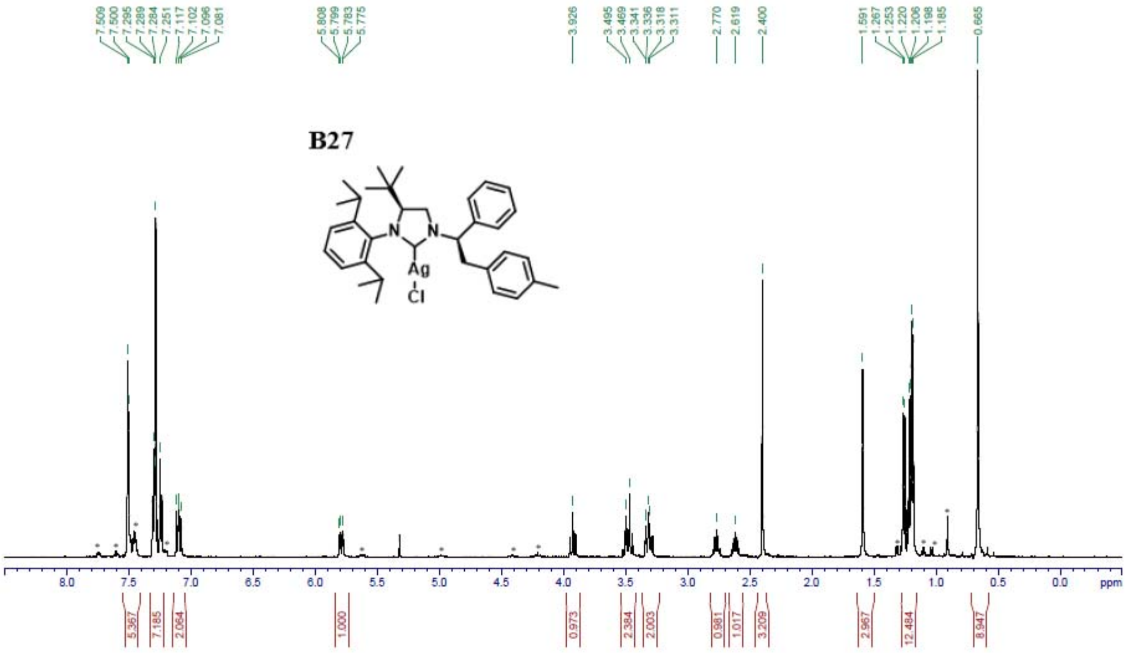

Figure S27. ^{1}H NMR (500 MHz) spectrum of **B27** in $CDCl_3$

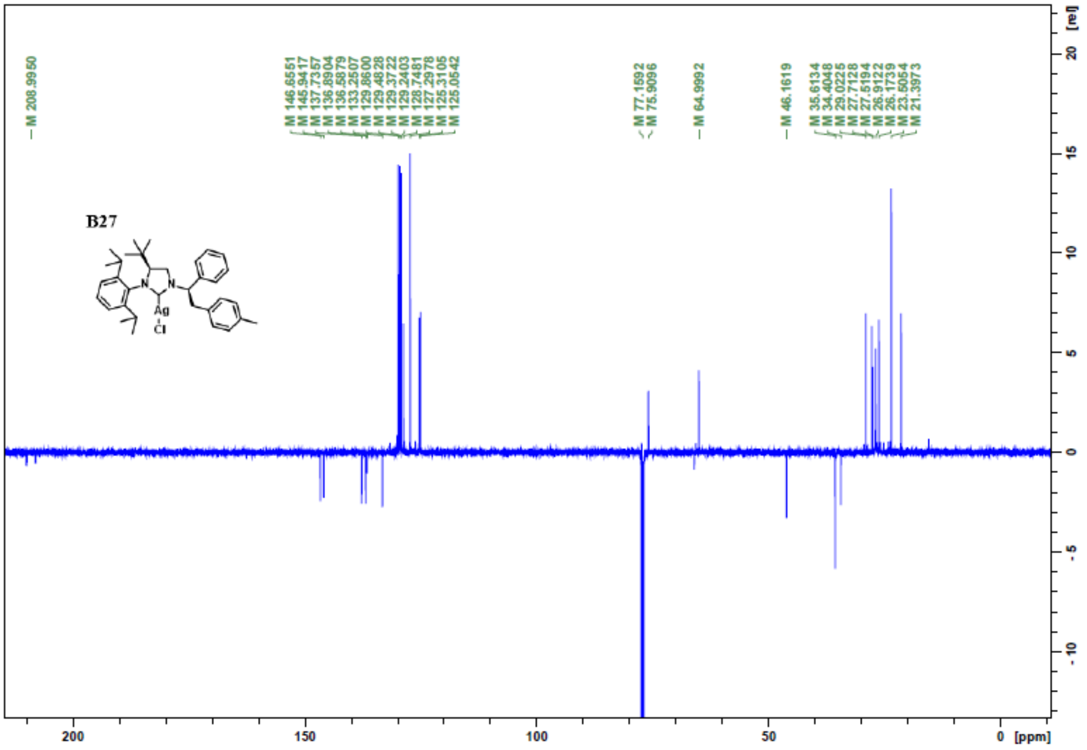

Figure S28. $^{13}C\{1H\}$ NMR (125 MHz) spectrum of **B27** in $CDCl_3$

(*4S*)-*4*-*terc*-Butyl-*3*-(*2'*,*6'*-diisopropylphenyl)-*1*-[(*1S*)-*2*-(*p*-tolyl)-*1*-phenylethyl]imidazolidin-2-ylidene-silver-chloride (B28)

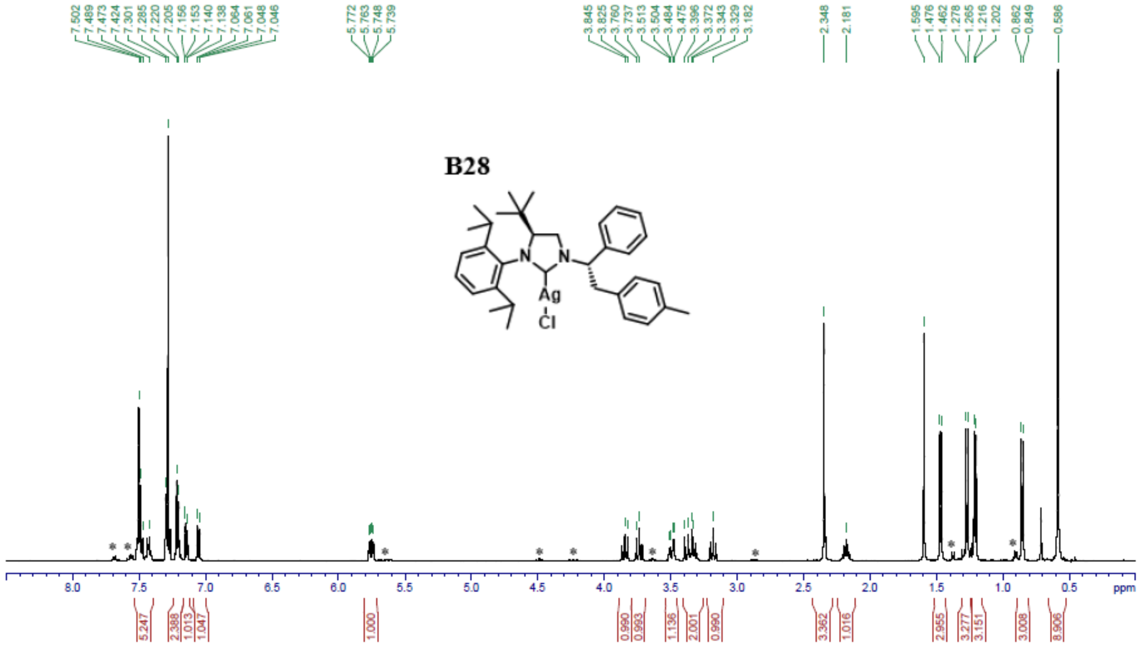

Figure S29. ^{1}H NMR (500 MHz) spectrum of **B28** in $CDCl_3$

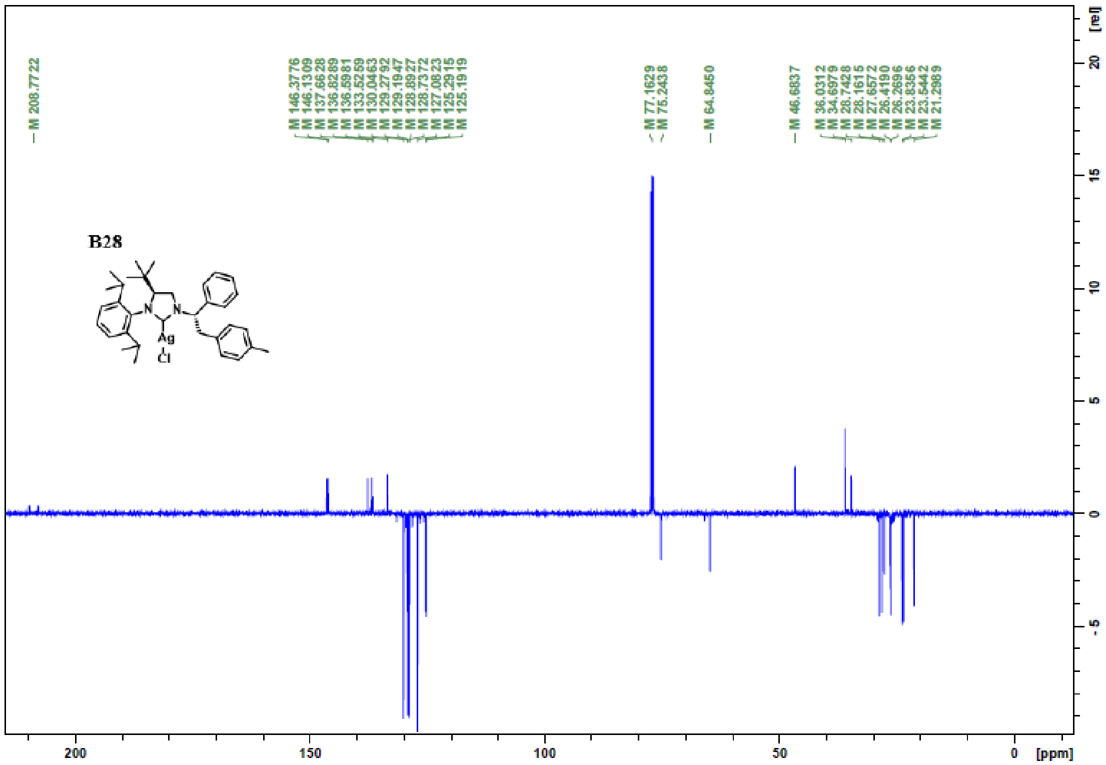

Figure S30. $^{13}C\{1H\}$ NMR (125 MHz) spectrum of **B28** in $CDCl_3$

(4*S*)-4-*terc*-Butyl-3-(2',6'-diisopropylphenyl)-1-[(1*R*)-2-(4-chlorophenyl)-1-phenylethyl]imidazolidin-2-ylidene-silver-chloride (B29)

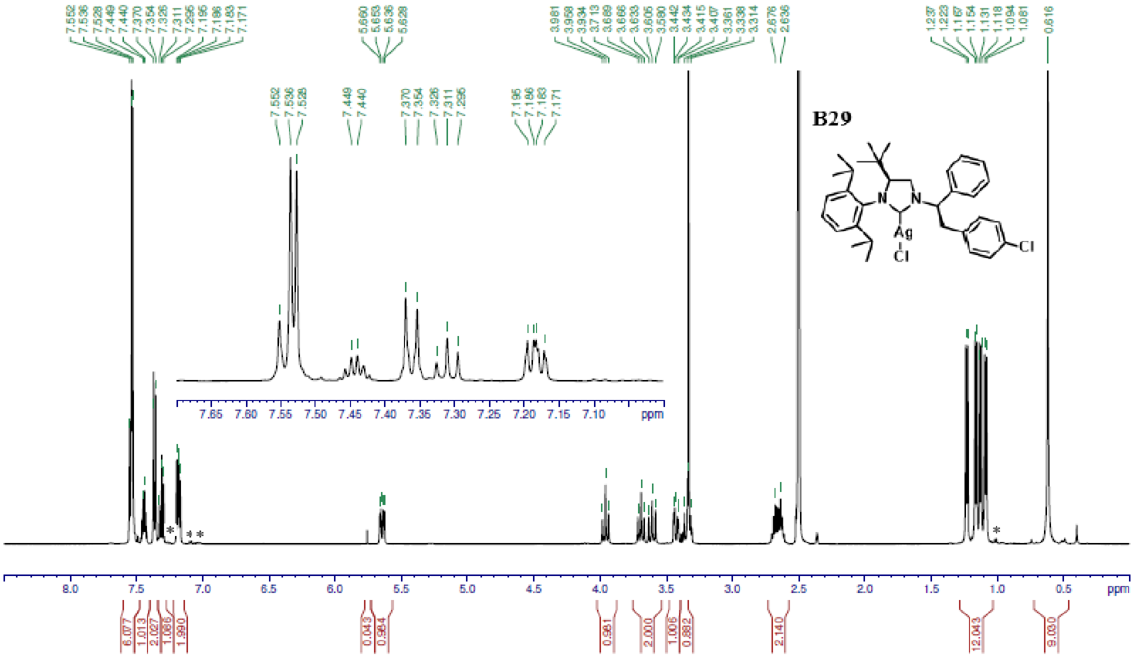

Figure S31. ^{1}H NMR (500 MHz) spectrum of **B29** in $CDCl_3$

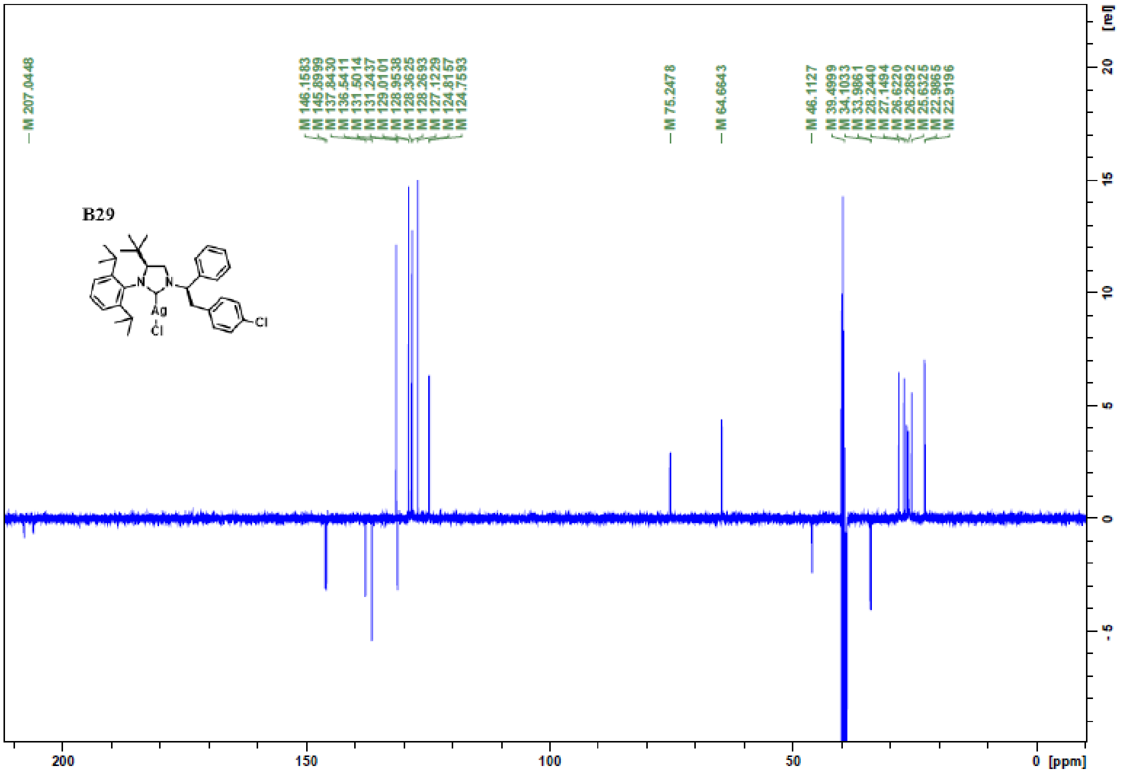

Figure S32. ^{13}C{1H} NMR (125 MHz) spectrum of **B29** in $CDCl_3$

(*4S*)-*4*-*terc*-Butyl-*3*-(*2'*,*6'*-diisopropylphenyl)-*1*-[(*1S*)-*2*-(*4*-chlorophenyl)-*1*-phenylethyl]imidazolidin-2-ylidene-silver-chloride (B30)

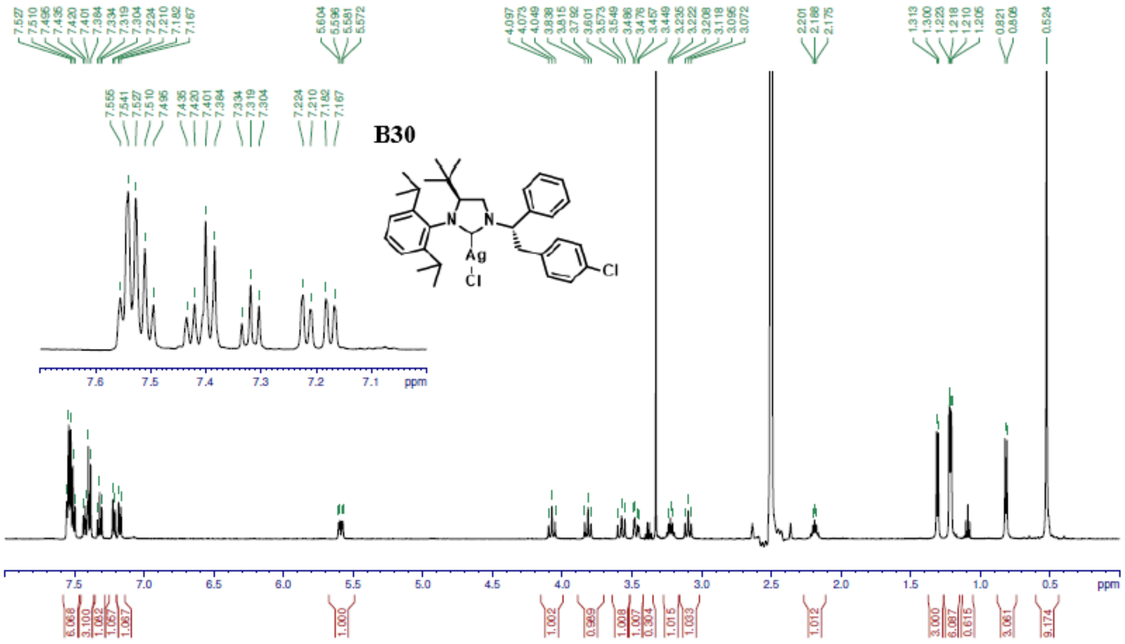

Figure S33. ^{1}H NMR (500 MHz) spectrum of **B30** in $CDCl_3$

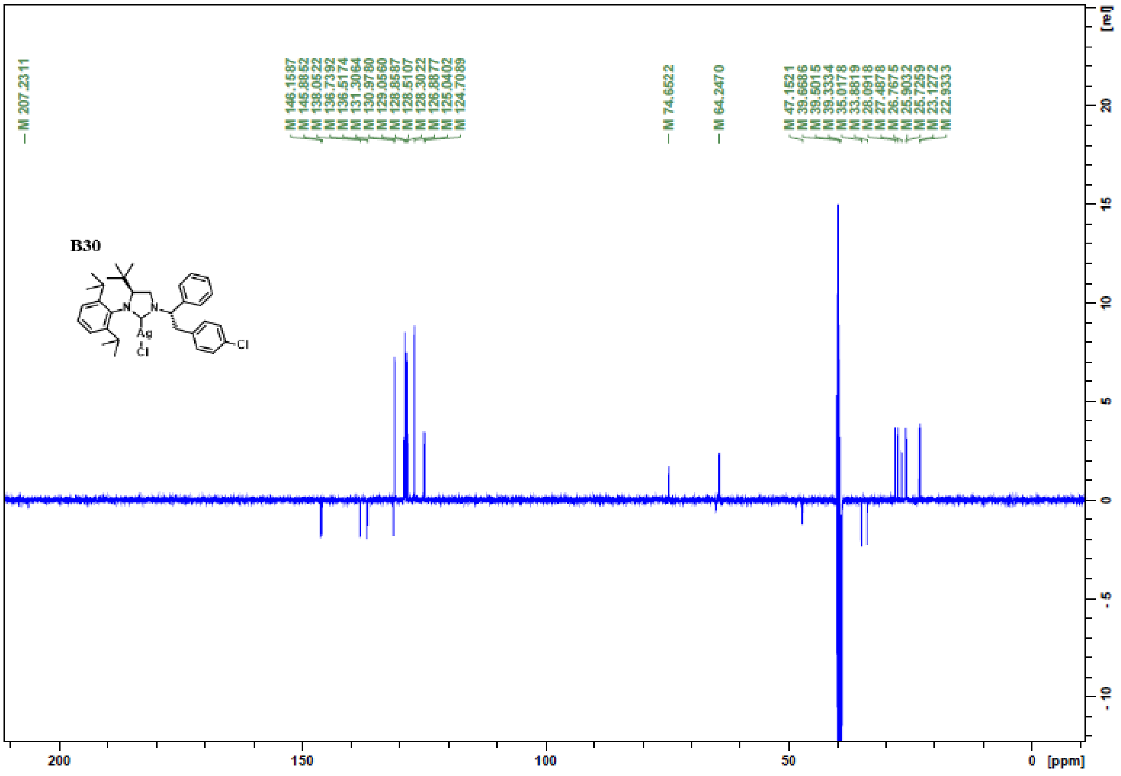

Figure S34. ^{13}C{1H} NMR (125 MHz) spectrum of **B30** in $CDCl_3$

(*4S*)-*4*-*terc*-Butyl-*3*-(*2'*,*6'*-diisopropylphenyl)-*1*-[(*1R*)-*2*-(*2*-chlorophenyl)-*1*-phenylethyl]imidazolidin-2-ylidene-silver-chloride (B31)

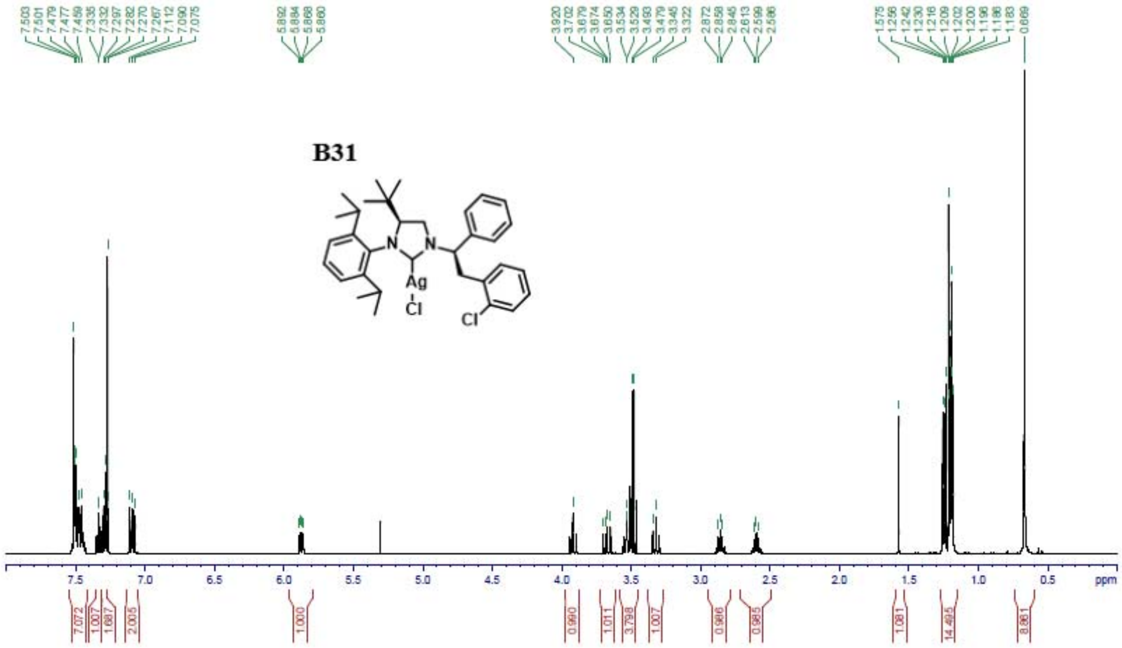

Figure S35. ^{1}H NMR (500 MHz) spectrum of **B31** in $CDCl_3$

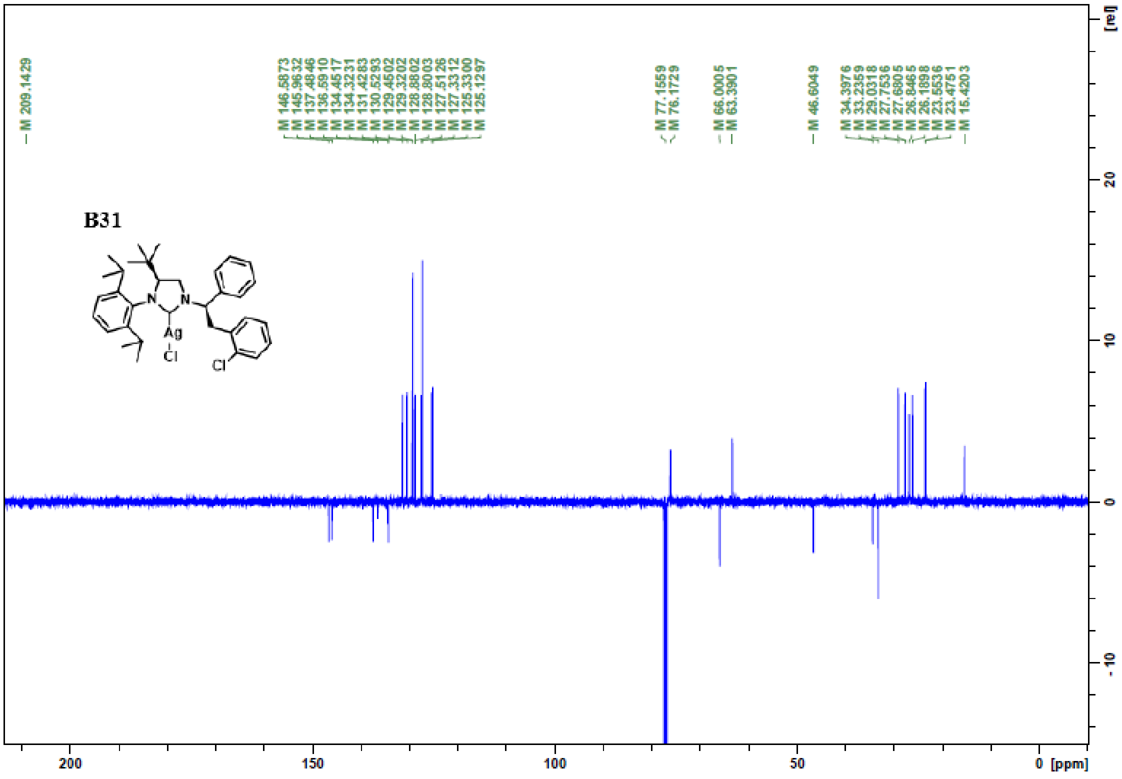

Figure S36. ^{13}C{1H} NMR (125 MHz) spectrum of **B31** in $CDCl_3$

(*4S*)-*4-terc*-Butyl-*3*-(*2',6'*-diisopropylphenyl)-*1*-[(*1S*)-*2*-(*2*-chlorophenyl)-*1*-phenylethyl]imidazolidin-2-ylidene-silver-chloride (B32)

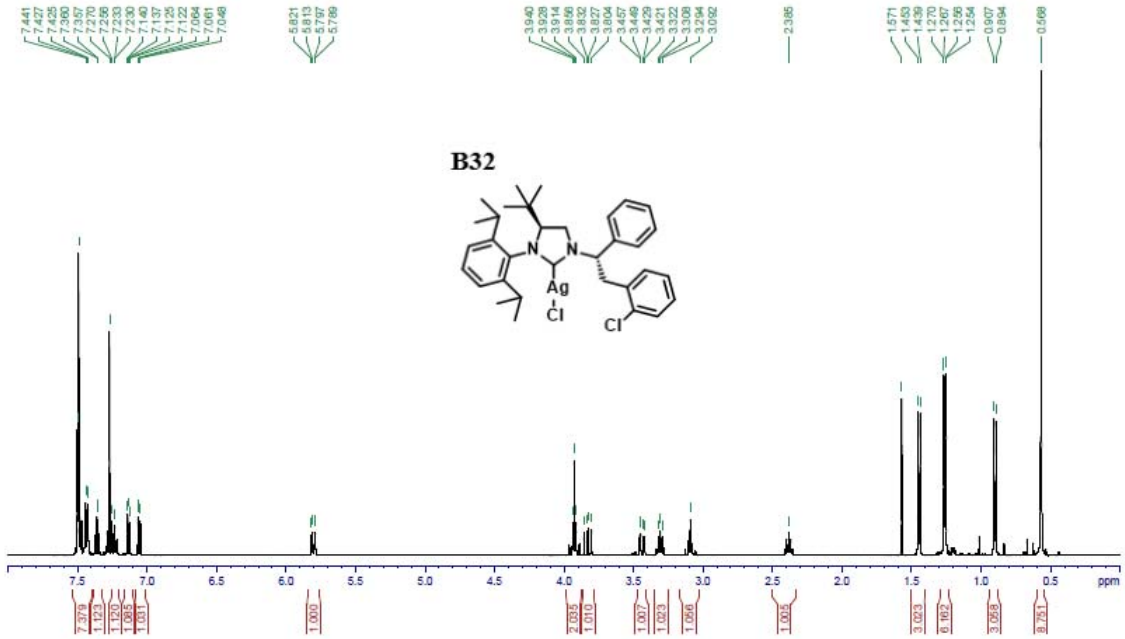

Figure S37. ^{1}H NMR (500 MHz) spectrum of **B32** in $CDCl_3$

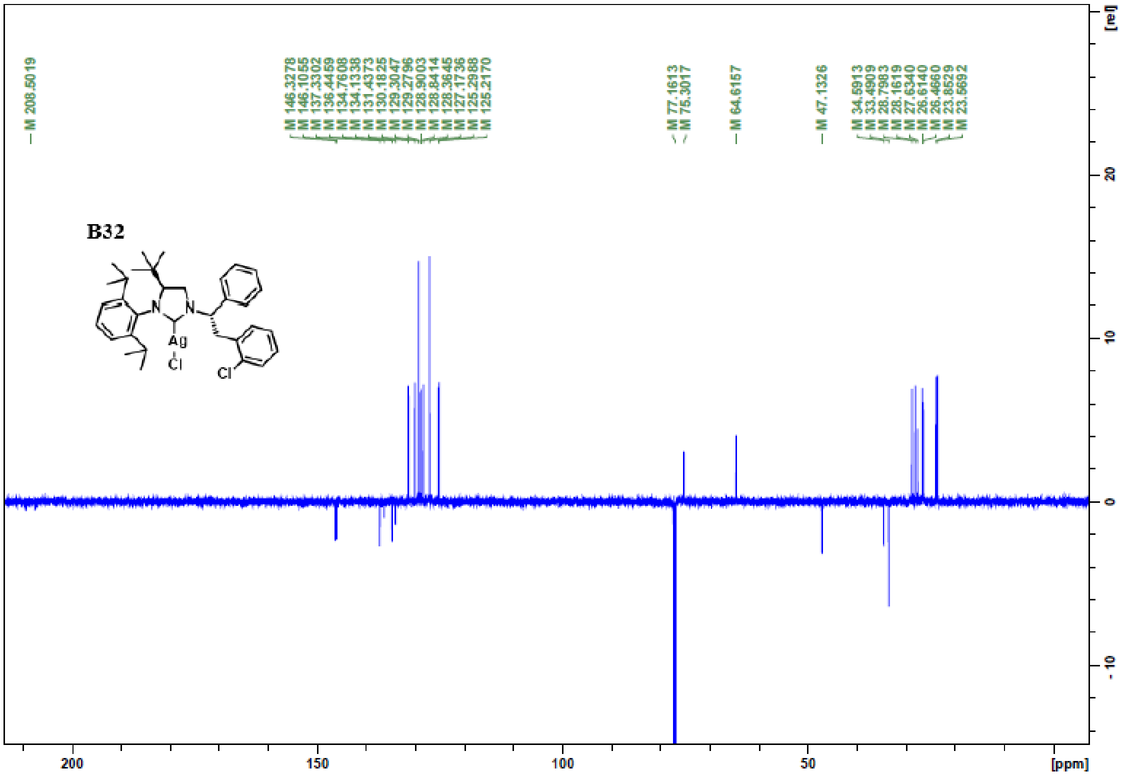

Figure S38. $^{13}C\{1H\}$ NMR (125 MHz) spectrum of **B32** in $CDCl_3$

(*4R*)-*4-terc*-Butyl-*3*-(*2',6'*-diisopropylphenyl)-*1*-[(*1R*)-*2*-(*3*-chlorophenyl)-*1*-phenylethyl]imidazolidin-2-ylidene-silver-chloride (B35)

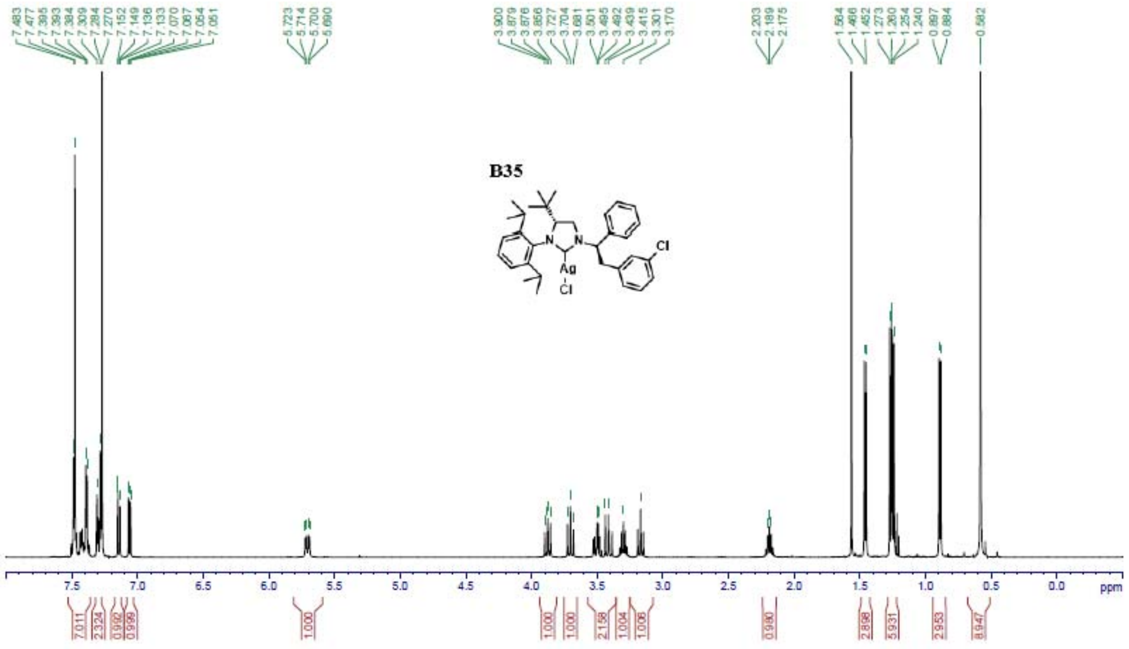

Figure S39. ^{1}H NMR (500 MHz) spectrum of **B35** in $CDCl_3$

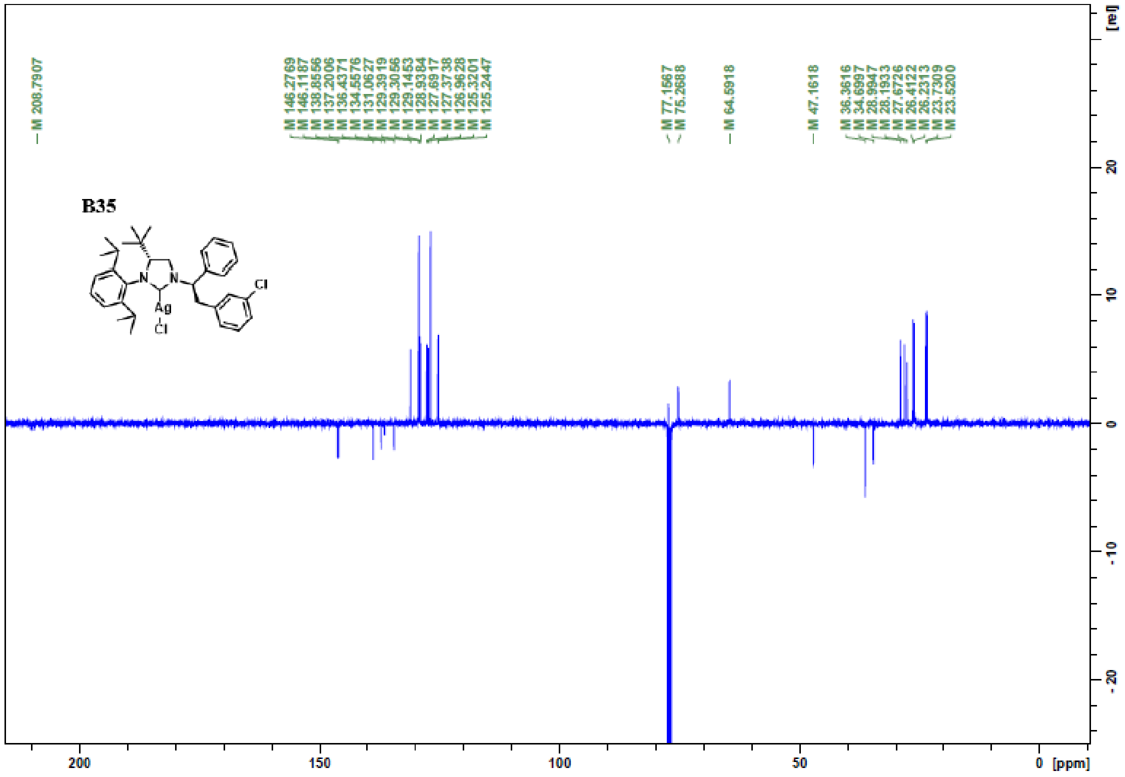

Figure S40. $^{13}C\{1H\}$ NMR (125 MHz) spectrum of **B35** in $CDCl_3$

(*4R*)-*4-terc*-Butyl-*3*-(*2',6'*-diisopropylphenyl)-*1*-[(*1S*)-*2*-(*3*-chlorophenyl)-*1*-phenylethyl]imidazolidin-2-ylidene-silver-chloride (B36)

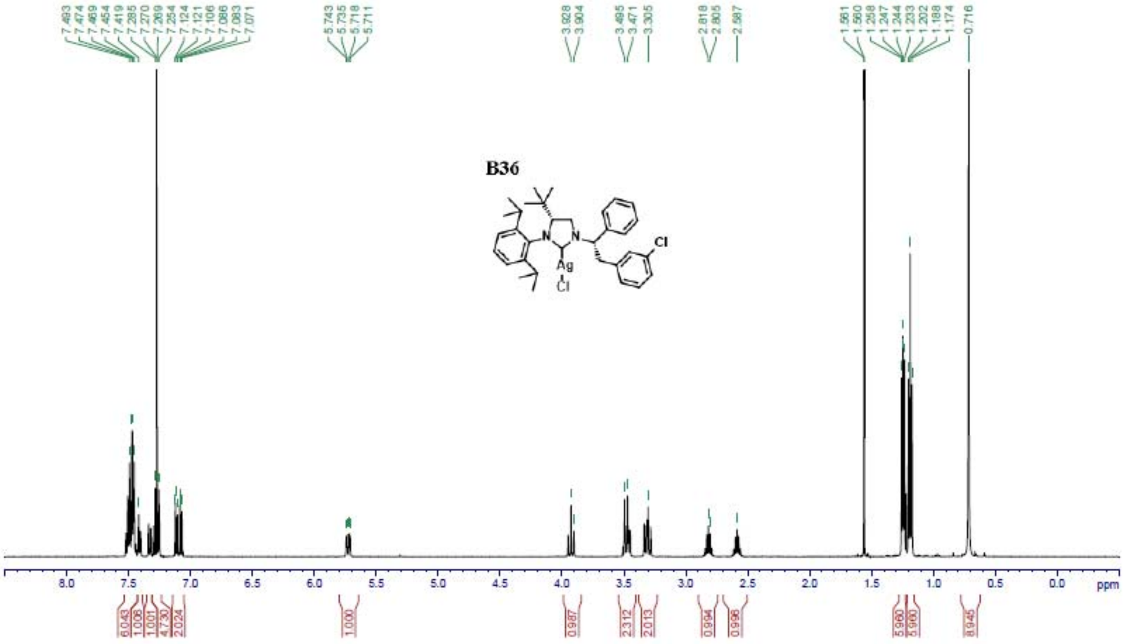

Figure S41. ^{1}H NMR (500 MHz) spectrum of **B36** in $CDCl_3$

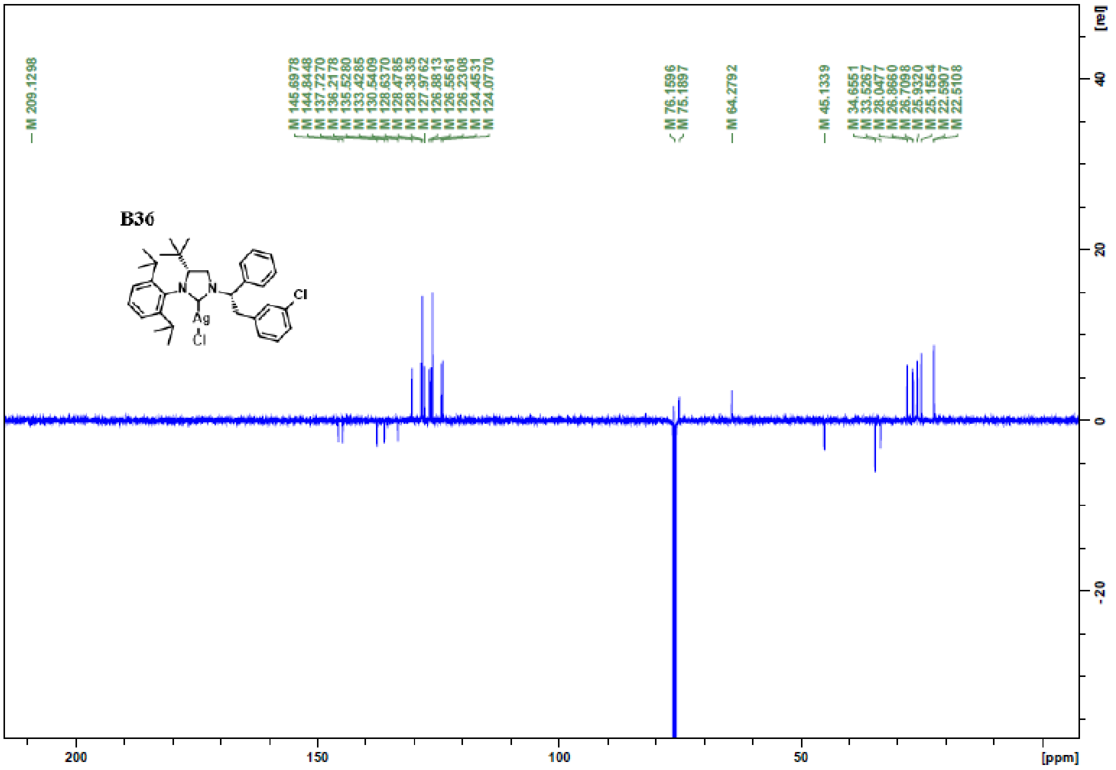

Figure S42. $^{13}C\{1H\}$ NMR (125 MHz) spectrum of **B36** in $CDCl_3$

(*4R*)-*4*-*terc*-Butyl-*3*-(*2’*,*6’*-diisopropylphenyl)-*1*-[(*1R*)-*1*-(*3*-trifluoromethyl)-*2*-phenylethyl]imidazolidin-2-ylidene-silver-chloride (B37)

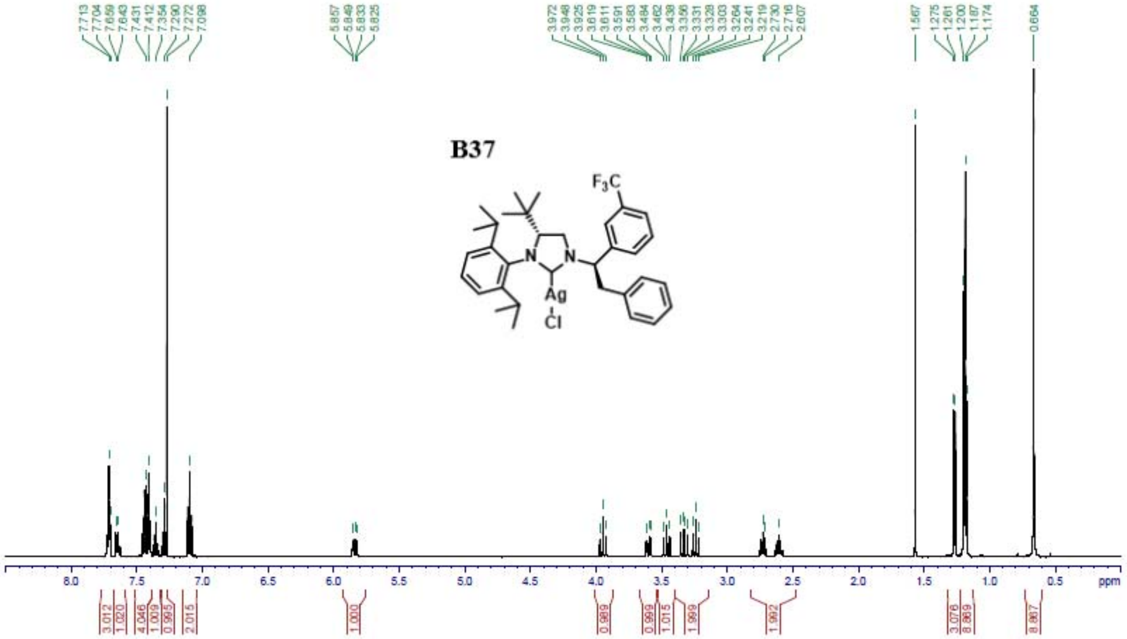

Figure S43. ^{1}H NMR (500 MHz) spectrum of **B37** in $CDCl_3$

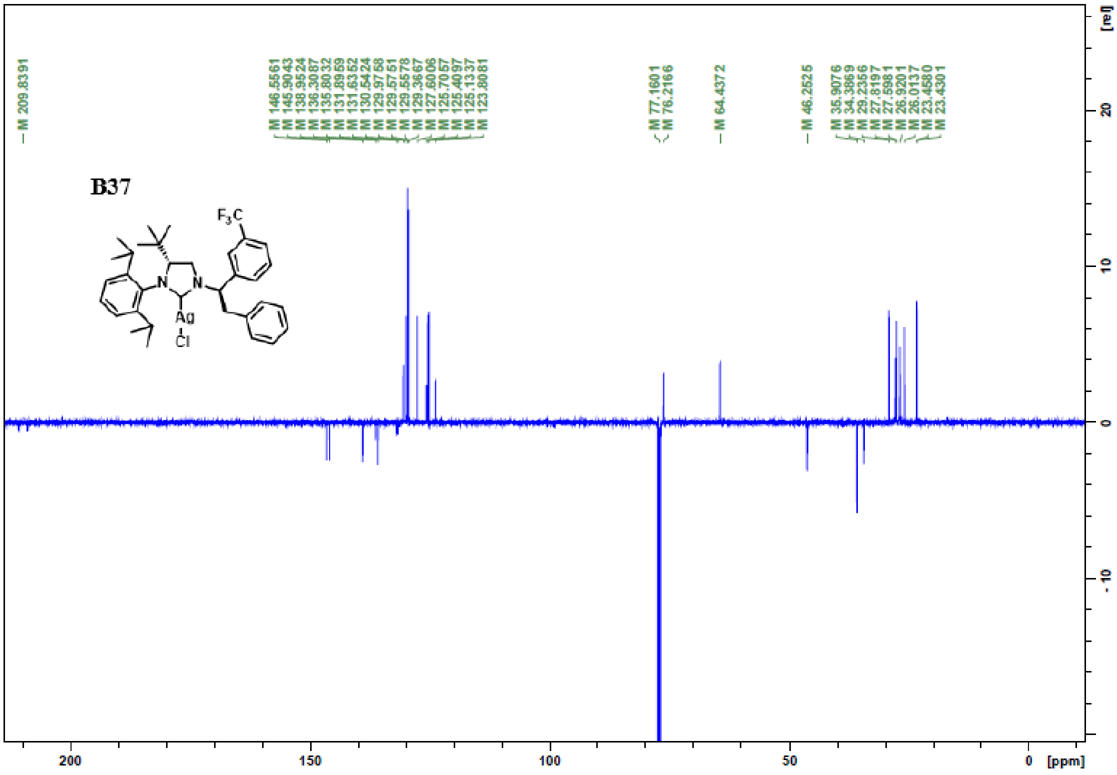

Figure S44. ^{13}C{1H} NMR (125 MHz) spectrum of **B37** in $CDCl_3$

(*4R*)-*4-terc*-Butyl-*3*-(*2’,6’*-diisopropylphenyl)-*1*-[(*1S*)-*1*-(*3*-trifluoromethyl)-*2*-phenylethyl]imidazolidin-2-ylidene-silver-chloride (B38)

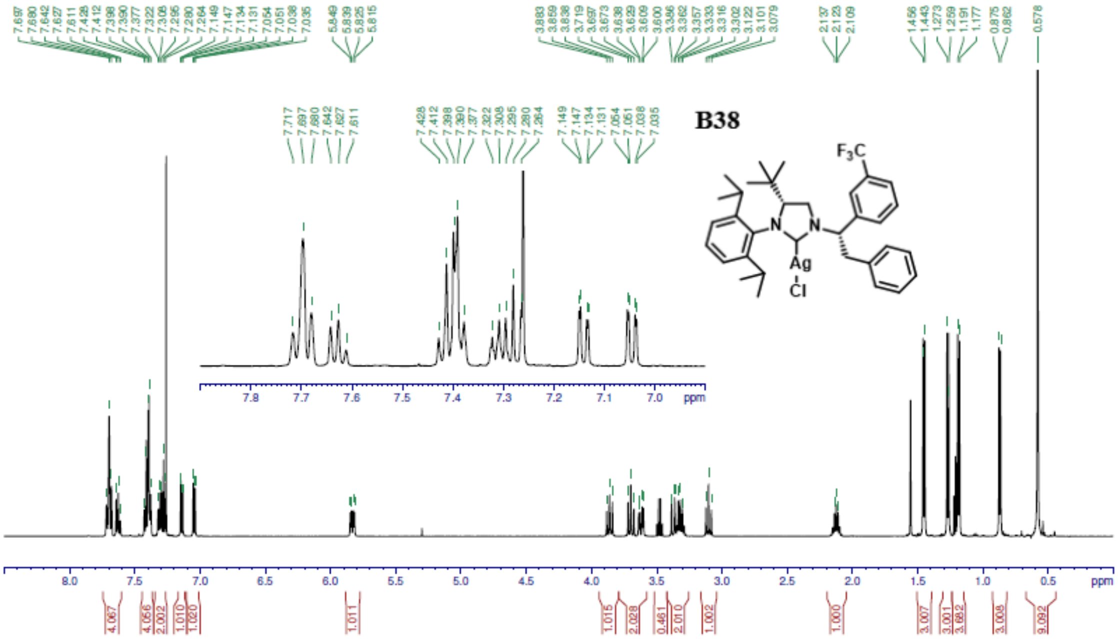

Figure S45. ^{1}H NMR (500 MHz) spectrum of **B38** in $CDCl_3$

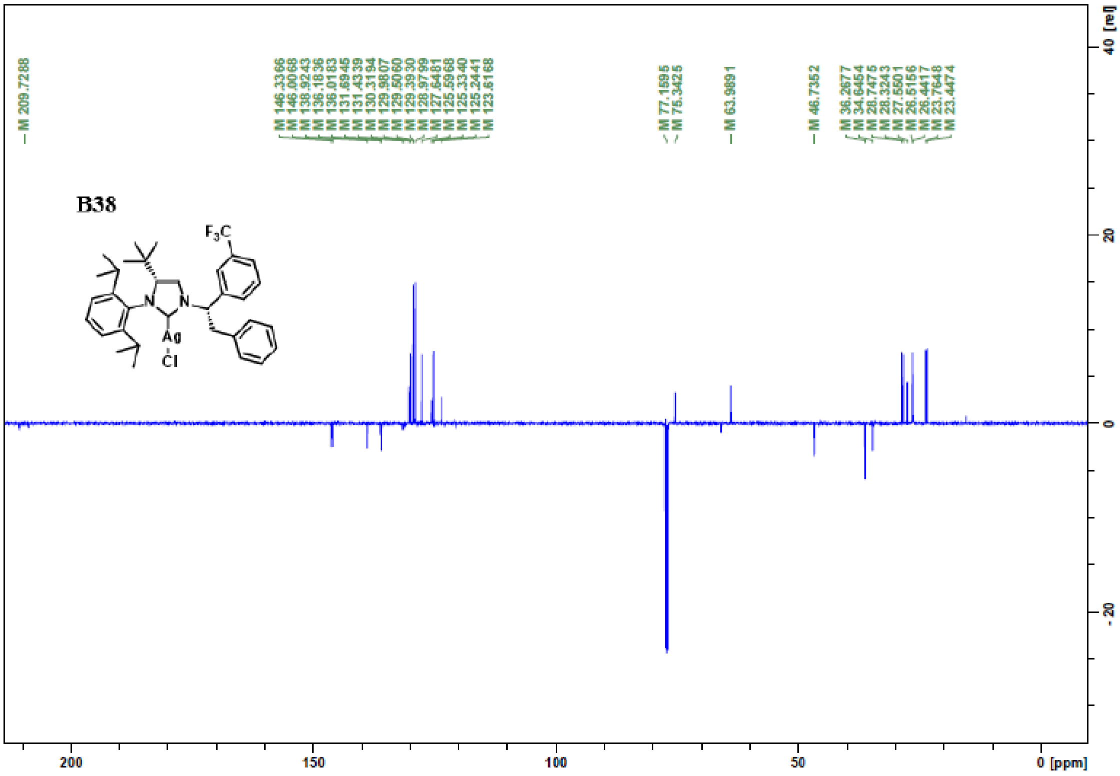

Figure S46. ^{13}C{1H} NMR (125 MHz) spectrum of **B38** in $CDCl_3$

***3*-(*2'*,*6'*-diisopropylphenyl)-*1*-[(*1R*)-*2*-(*4*-chlorophenyl)-*1*-phenylethyl]imidazolidin-2-ylidene-silver-chloride (B39)**

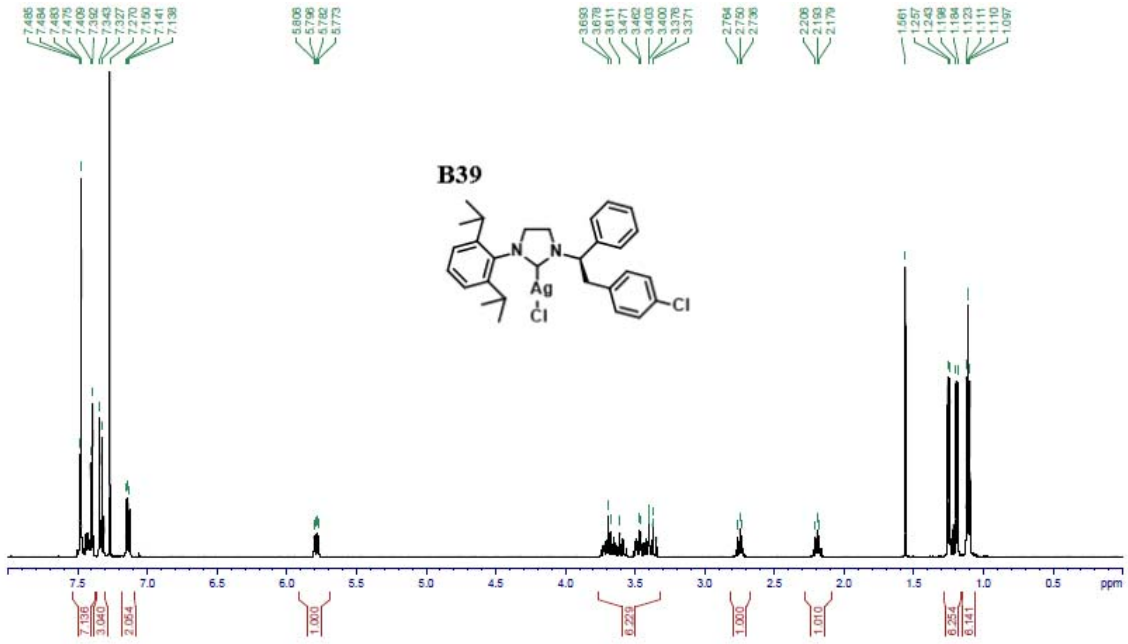

Figure S47. ^{1}H NMR (500 MHz) spectrum of **B39** in $CDCl_3$

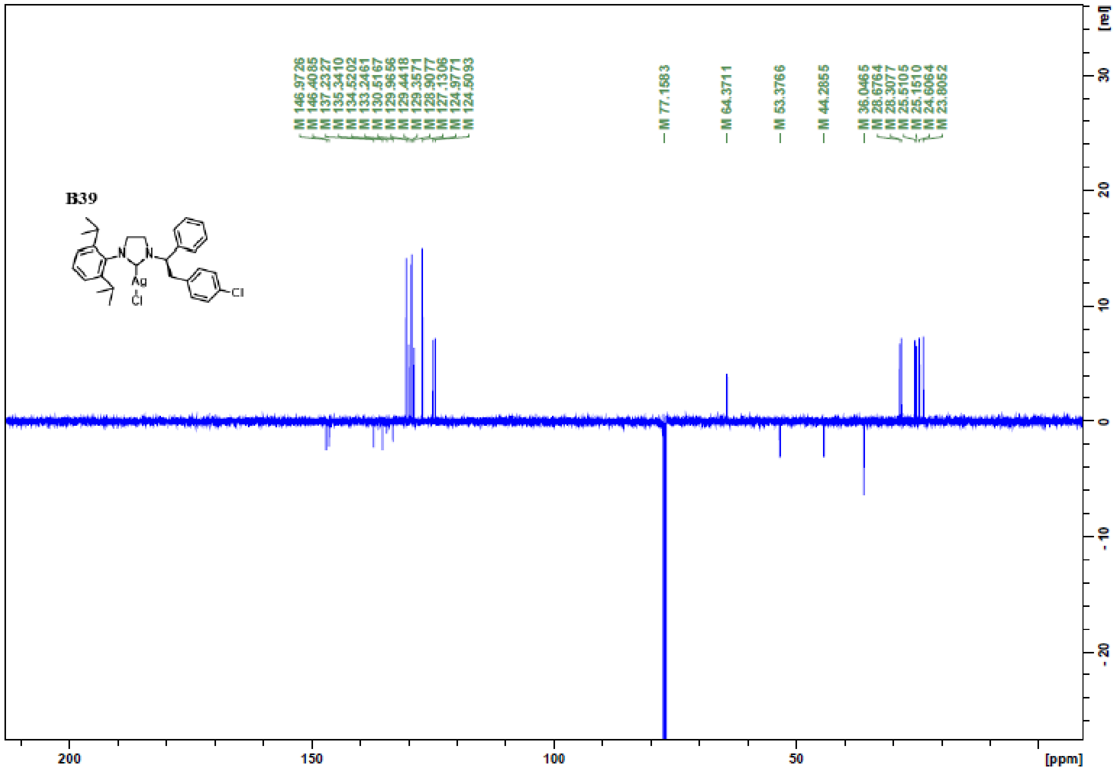

Figure S48. ^{13}C{1H} NMR (125 MHz) spectrum of **B39** in $CDCl_3$

***3*-(*2’*,*6’*-diisopropylphenyl)-*1*-[(*1S*)-*2*-(*4*-chlorophenyl)-*1*-phenylethyl]imidazolidin-2-ylidene-silver-chloride (B40)**

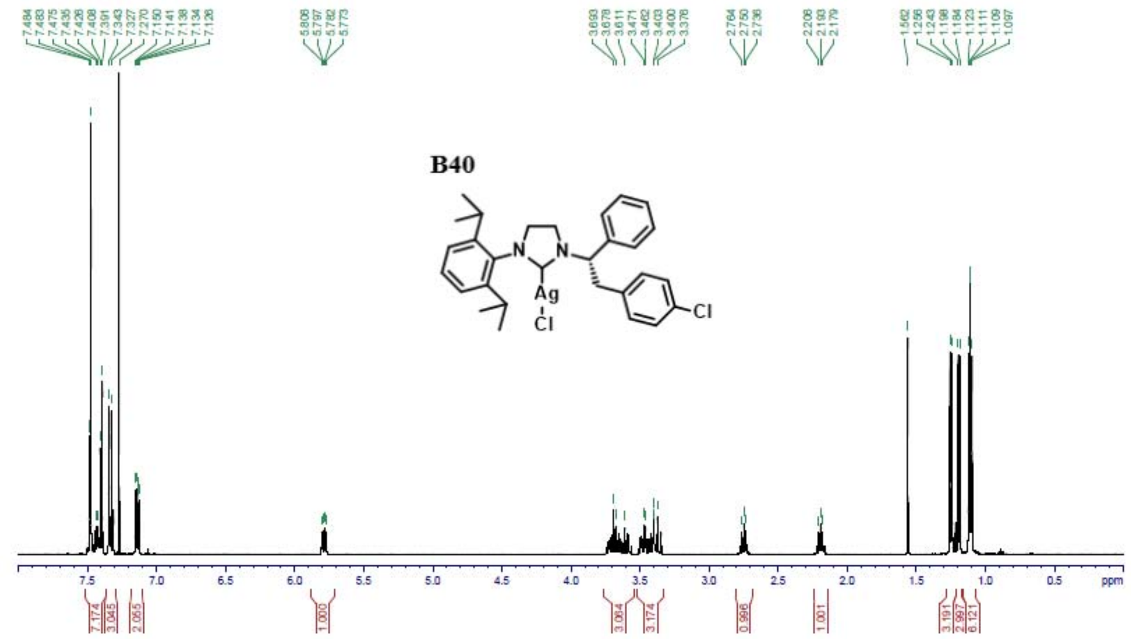

Figure S49. ^{1}H NMR (500 MHz) spectrum of **B40** in $CDCl_3$

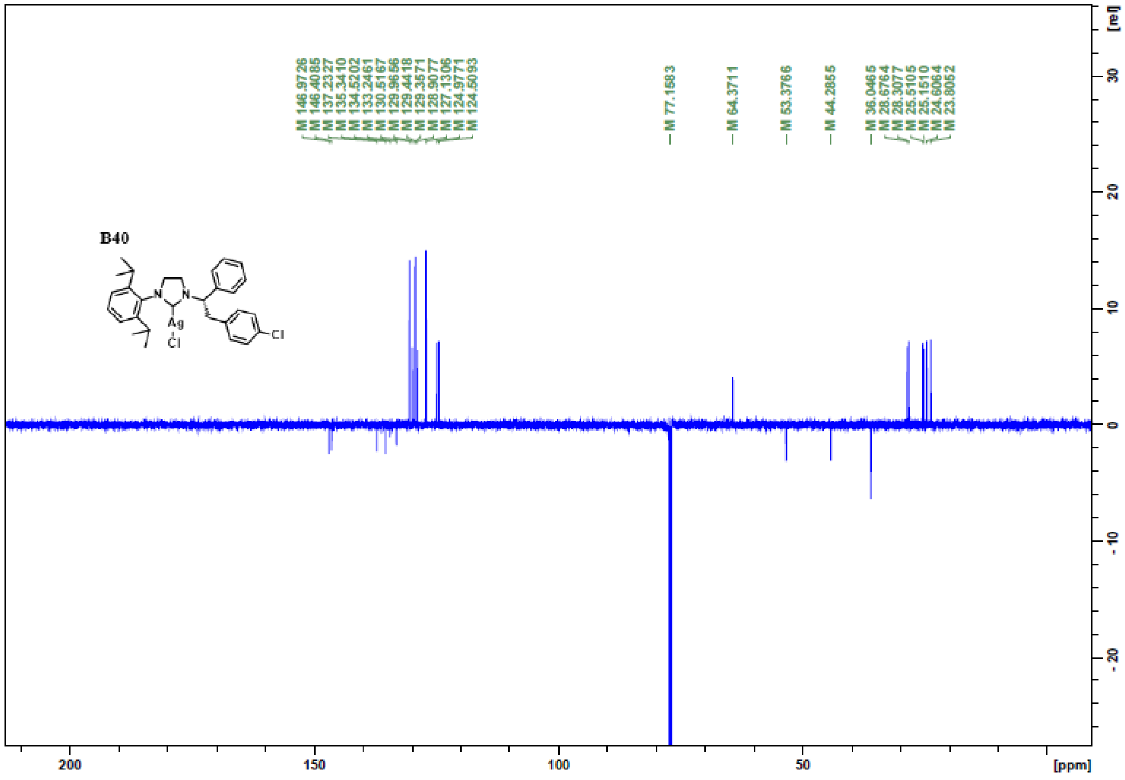

Figure S50. ^{13}C{1H} NMR (125 MHz) spectrum of **B40** in $CDCl_3$

(*4S*)-*4*-*tert*-Butyl-*3*-(*2',6'*-diisopropylphenyl)-*1*-[(*1S*)-*2'*-naphtylethyl]imidazolidin-2-ylidene-gold-chloride (C5)

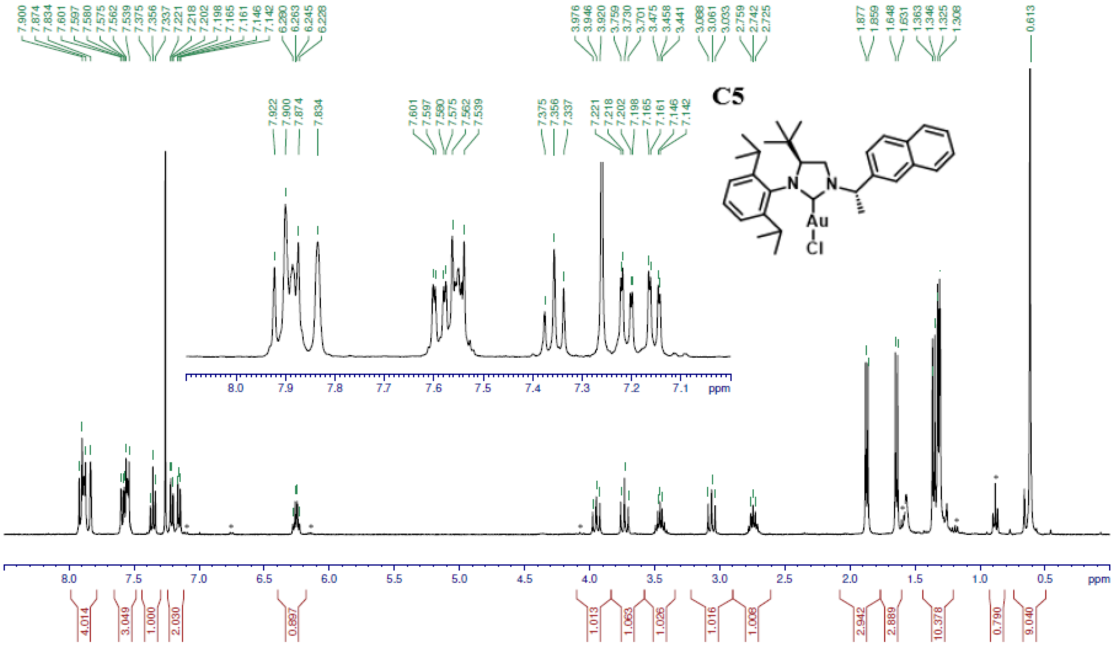

Figure S51. ^{1}H NMR (400 MHz) spectrum of **C5** in $CDCl_3$

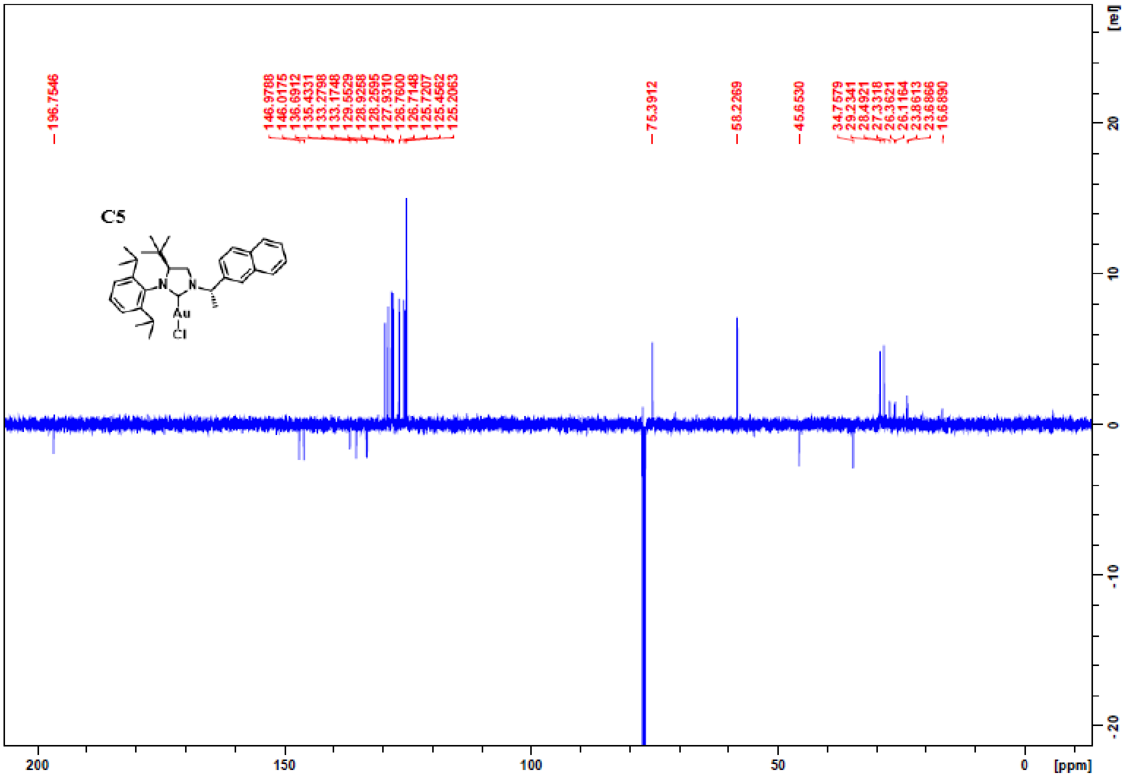

Figure S52. $^{13}C\{1H\}$ NMR (100 MHz) spectrum of **C5** in $CDCl_3$

(*4S*)-*4*-*tert*-Butyl-*3*-(*2',6'*-diisopropylphenyl)-*1*-[(*1R*)-*2'*-naphtylethyl]imidazolidin-2-ylidene-gold-chloride (C6)

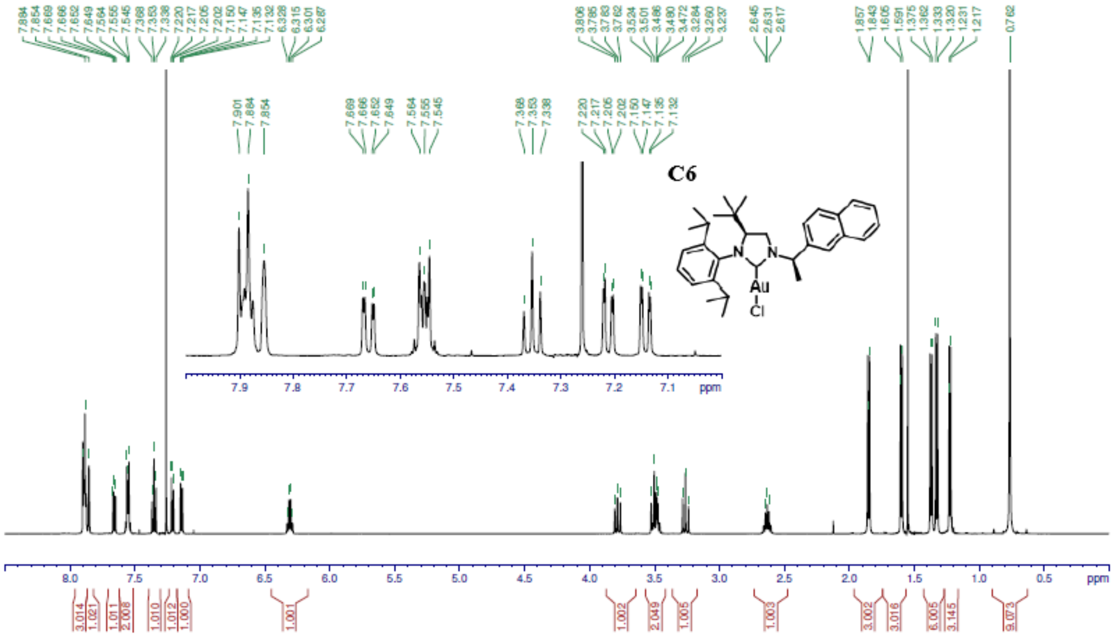

Figure S53. ^{1}H NMR (500 MHz) spectrum of **C6** in $CDCl_3$

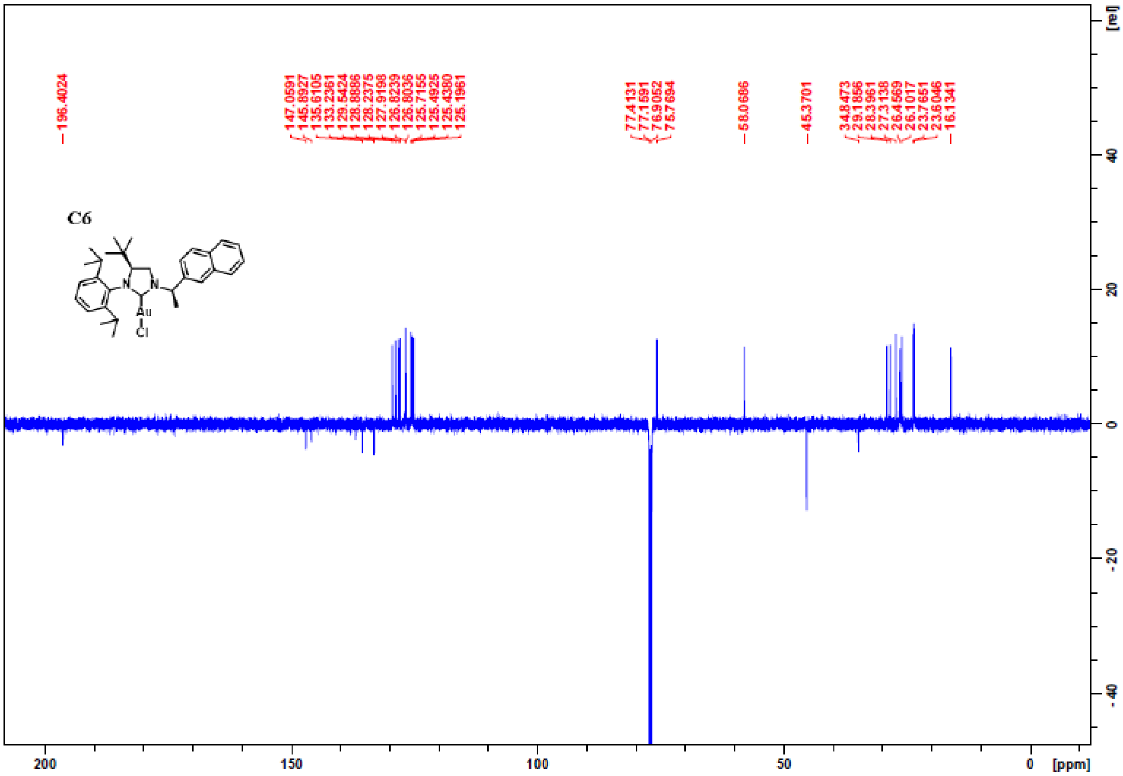

Figure S54. ^{13}C{1H} NMR (125 MHz) spectrum of **C6** in $CDCl_3$

(*4S*)-*4-tert*-Butyl-*3*-(*2’,6’*-diisopropylphenyl)-*1*-[(*1R*)-*1*-(*4*-trifluoromethyl)-*2*-phenylethyl]imidazolidin-2-ylidene-gold-chloride (C15)

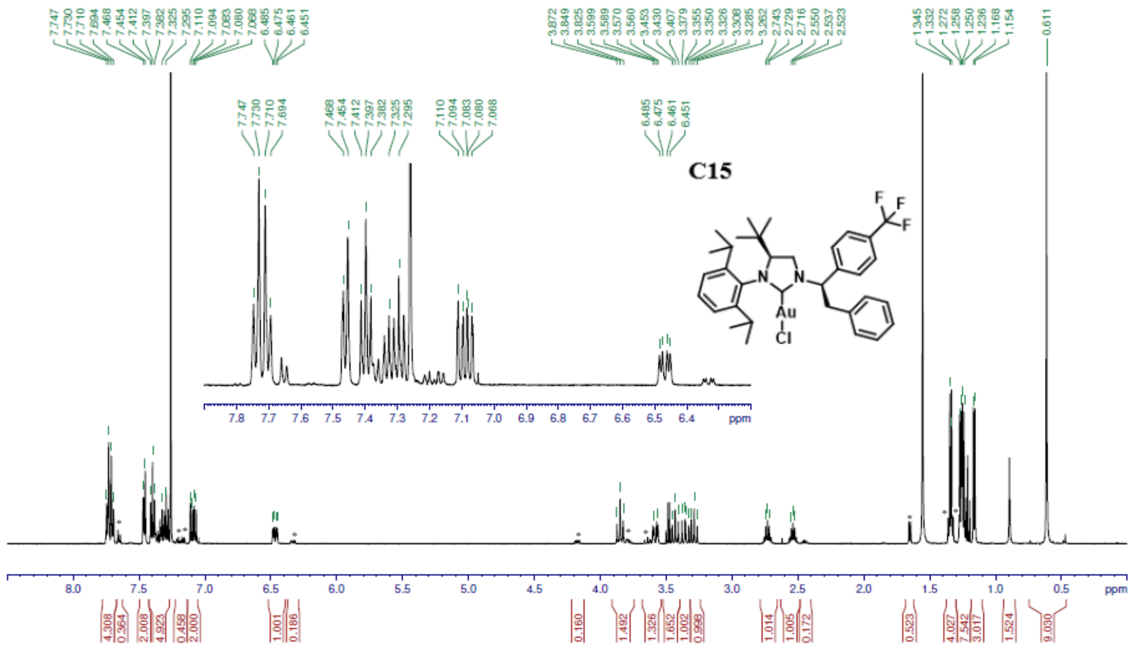

Figure S55. ^{1}H NMR (500 MHz) spectrum of **C15** in $CDCl_3$

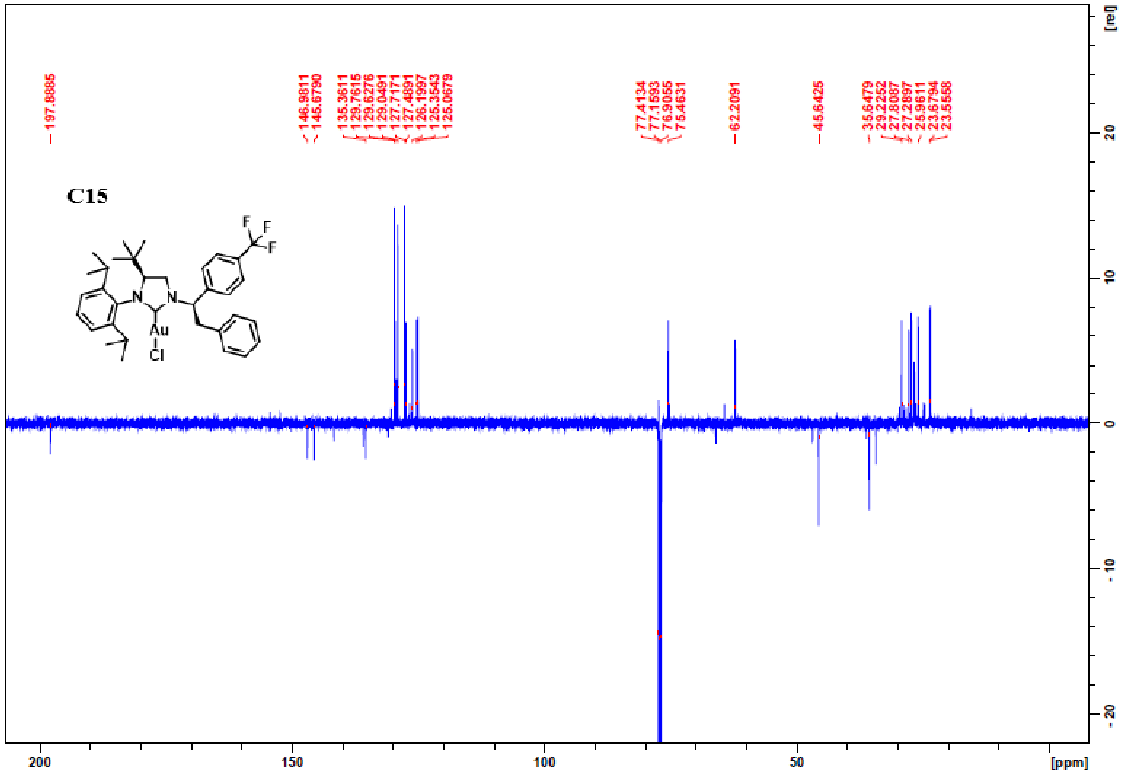

Figure S56. $^{13}C\{1H\}$ NMR (125 MHz) spectrum of **C15** in $CDCl_3$

(*4S*)-*4*-*tert*-Butyl-*3*-(*2'*,*6'*-diisopropylphenyl)-*1*-[(*1S*)-*1*-(*4*-trifluoromethyl)-*2*-phenylethyl]imidazolidin-2-ylidene-gold-chloride (C16)

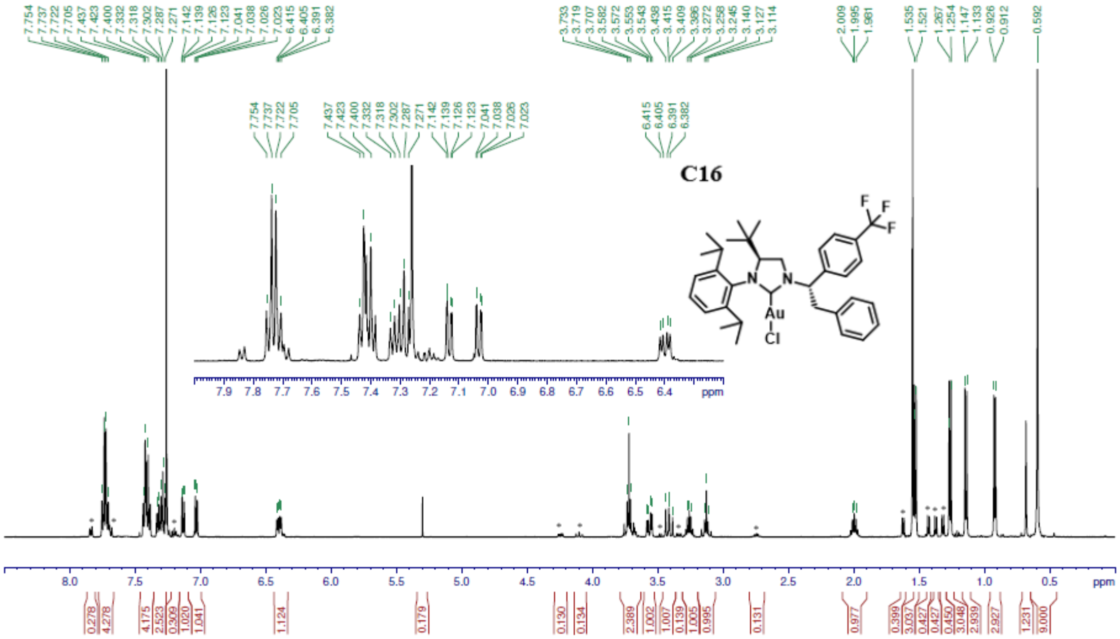

Figure S57. ^{1}H NMR (500 MHz) spectrum of **C16** in $CDCl_3$

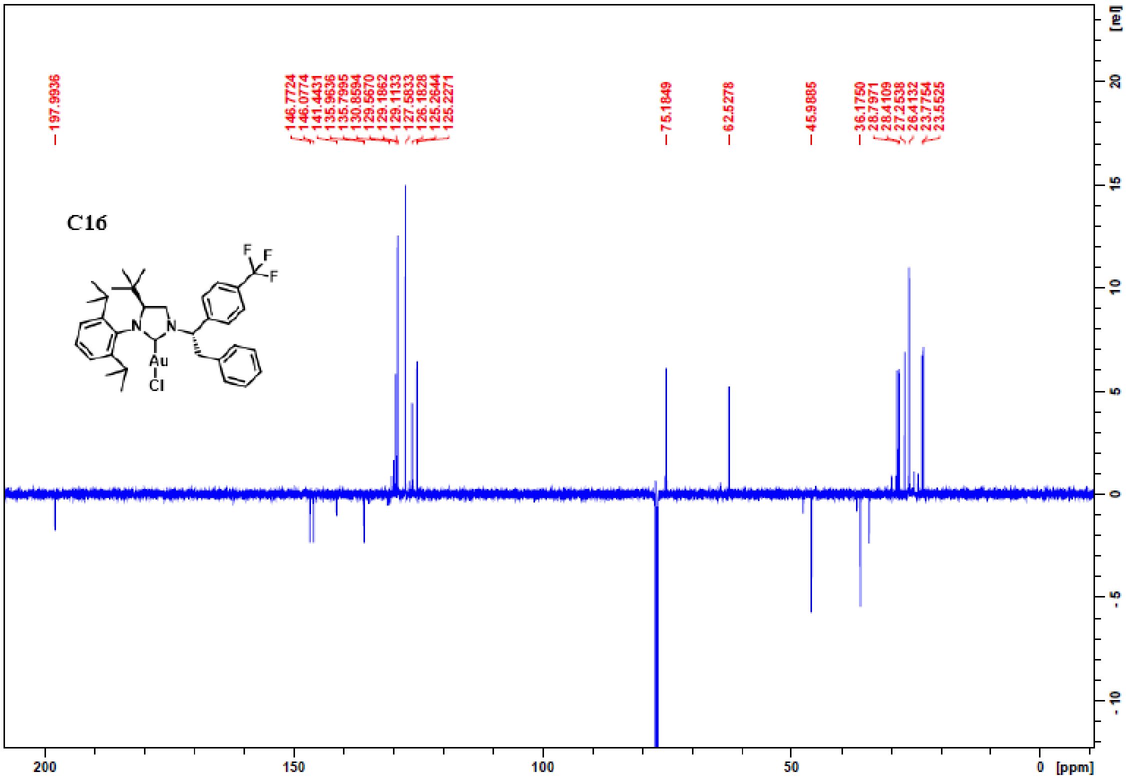

Figure S58. ^{13}C{1H} NMR (125 MHz) spectrum of **C16** in $CDCl_3$

(*4S*)-*4*-*tert*-Butyl-*3*-(*2'*,*6'*-diisopropylphenyl)-*1*-[(*1R*)-*1*-(*4*-methoxyphenyl)-*2*-phenylethyl]imidazolidin-2-ylidene-gold-chloride (C17)

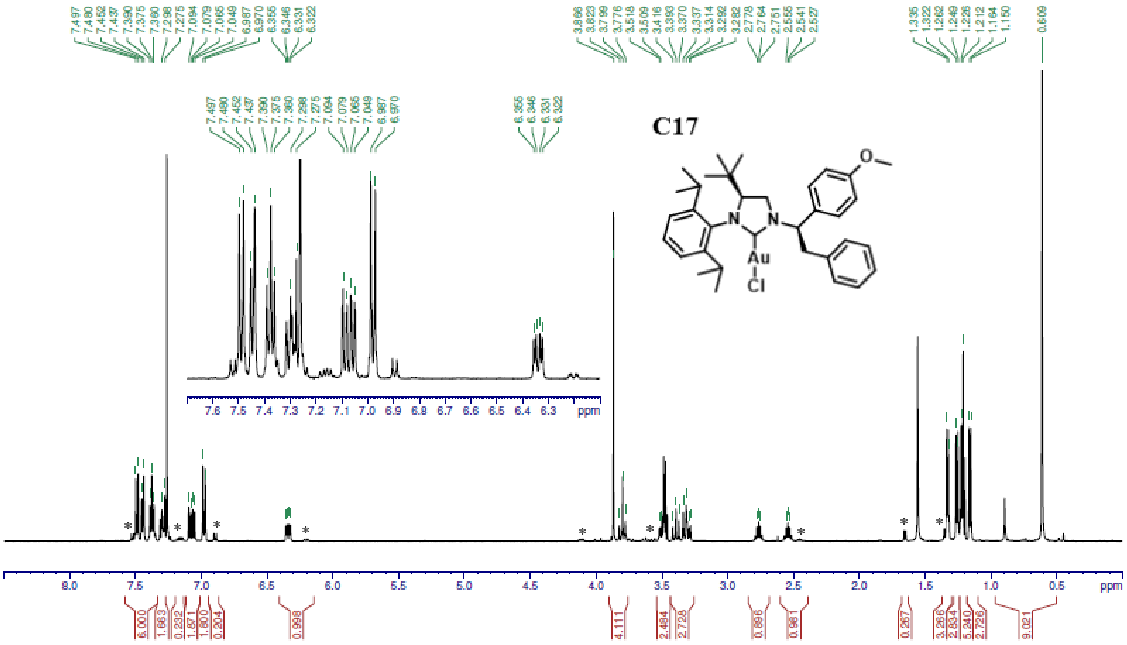

Figure S59. ^{1}H NMR (500 MHz) spectrum of **C17** in $CDCl_3$

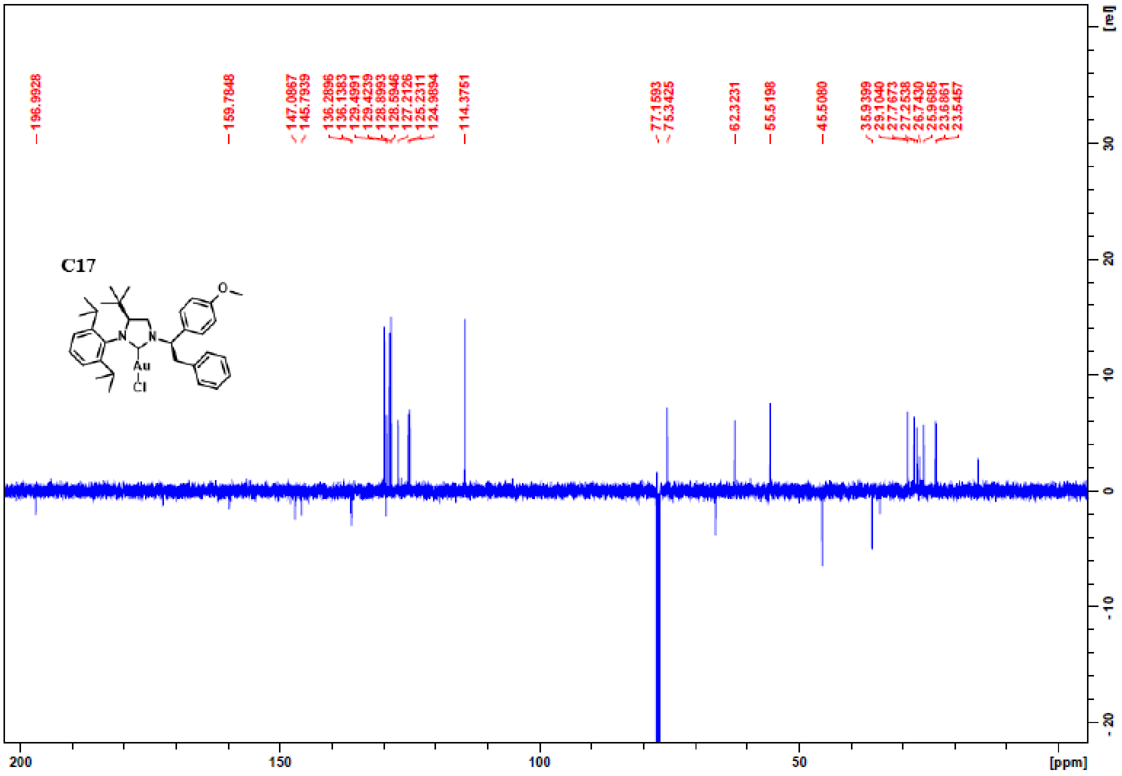

Figure S60. $^{13}C\{1H\}$ NMR (125 MHz) spectrum of **C17** in $CDCl_3$

(4*S*)-4-*tert*-Butyl-3-(2',6'-diisopropylphenyl)-1-[(1*S*)-1-(4-methoxyphenyl)-2-phenylethyl]imidazolidin-2-ylidene-gold-chloride (C18)

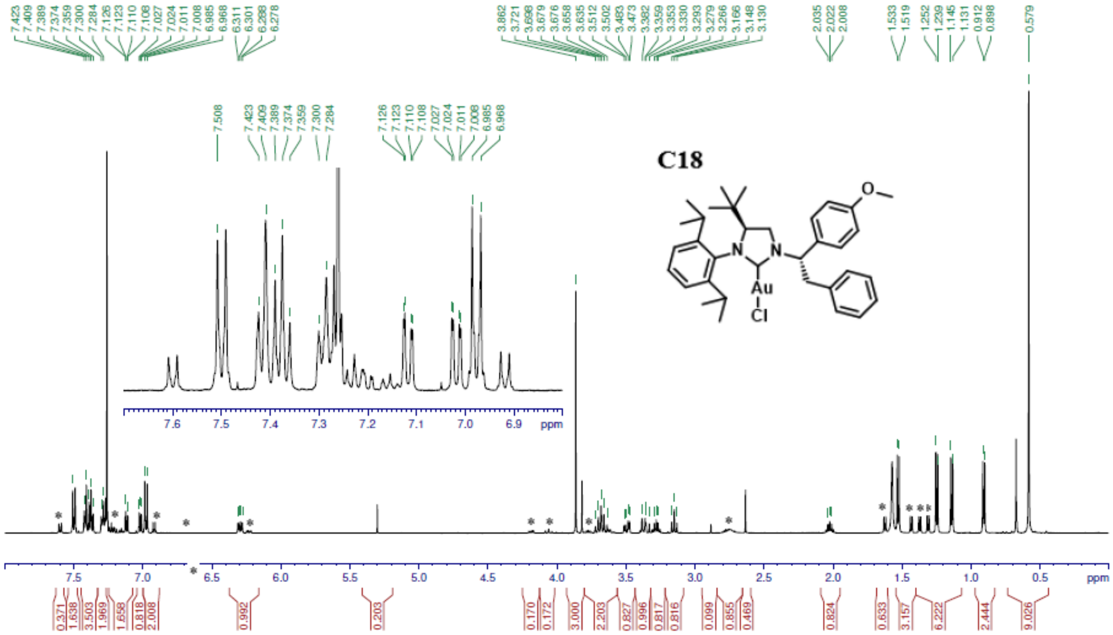

Figure S61. ^{1}H NMR (500 MHz) spectrum of **C18** in $CDCl_3$

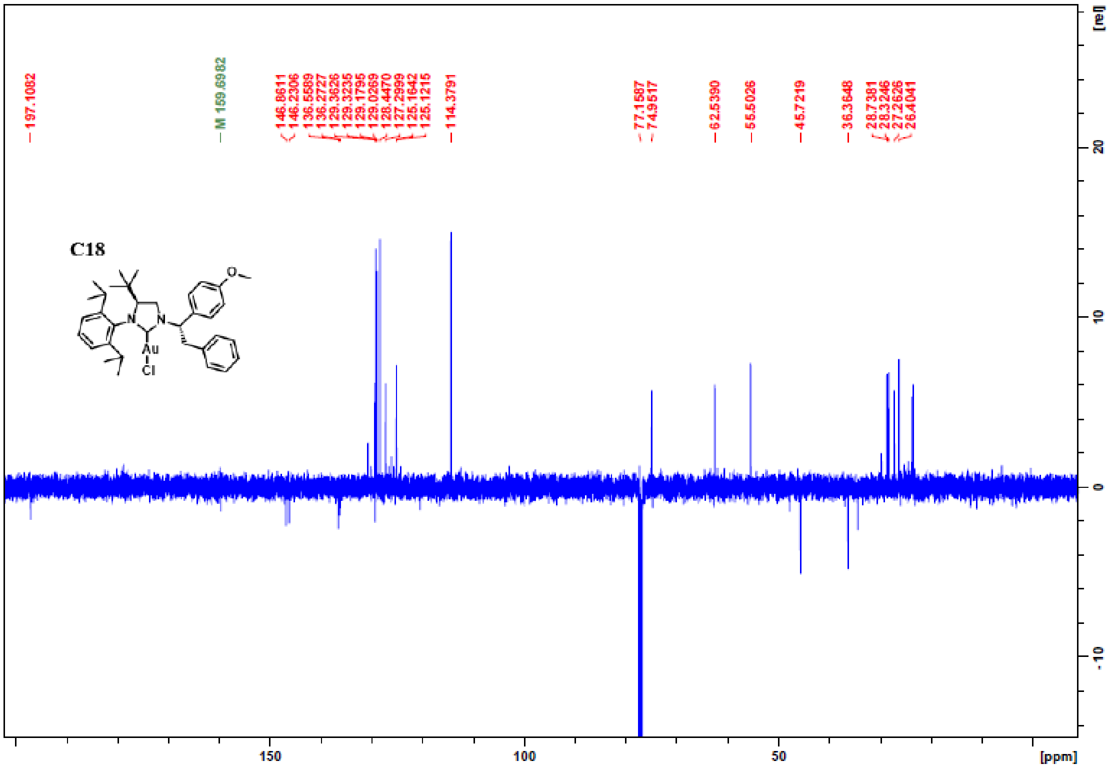

Figure S62. ^{13}C{1H} NMR (125 MHz) spectrum of **C18** in $CDCl_3$

(4*S*)-4-*tert*-Butyl-3-(2',6'-diisopropylphenyl)-1-[(1*R*)-1-(*p*-tolyl)-2-phenylethyl]imidazolidin-2-ylidene-gold-chloride (C19)

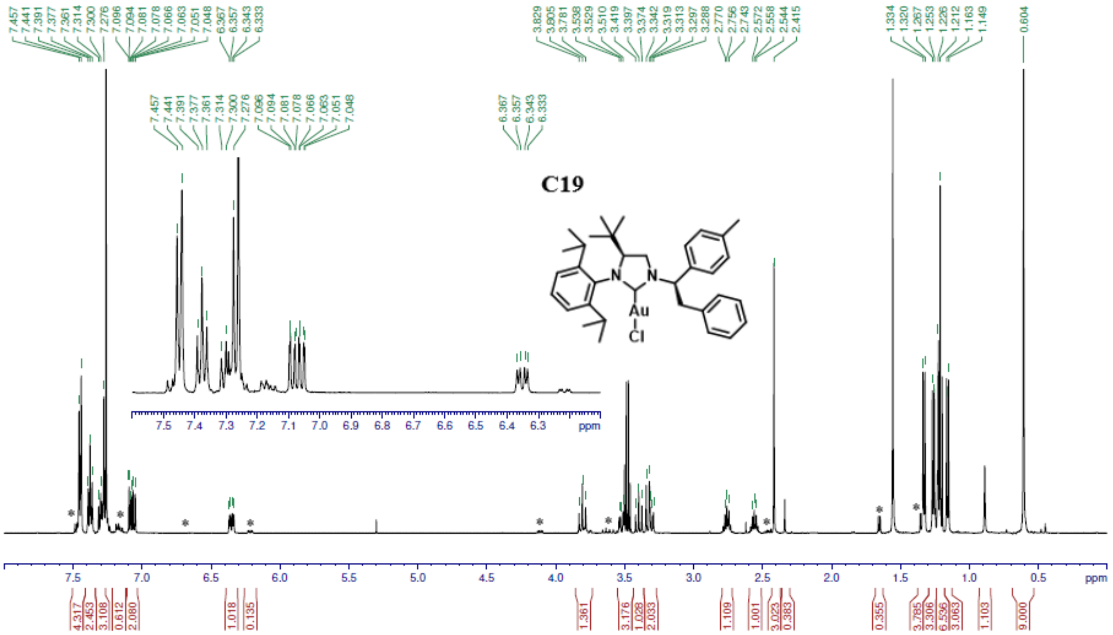

Figure S63. ^{1}H NMR (500 MHz) spectrum of **C19** in $CDCl_3$

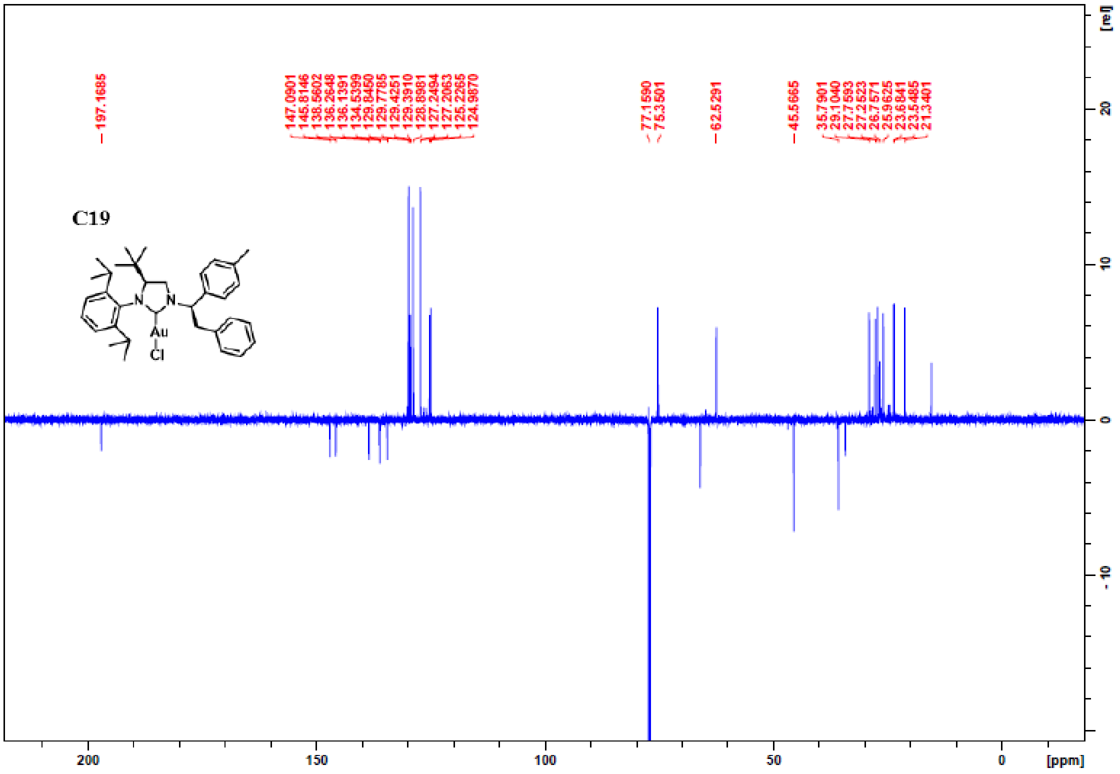

Figure S64. ^{13}C{1H} NMR (125 MHz) spectrum of **C19** in $CDCl_3$

(4*S*)-4-*tert*-Butyl-3-(2’,6’-diisopropylphenyl)-1-[(1*S*)-1-(*p*-tolyl)-2-phenylethyl]imidazolidin-2-ylidene-gold-chloride (C20)

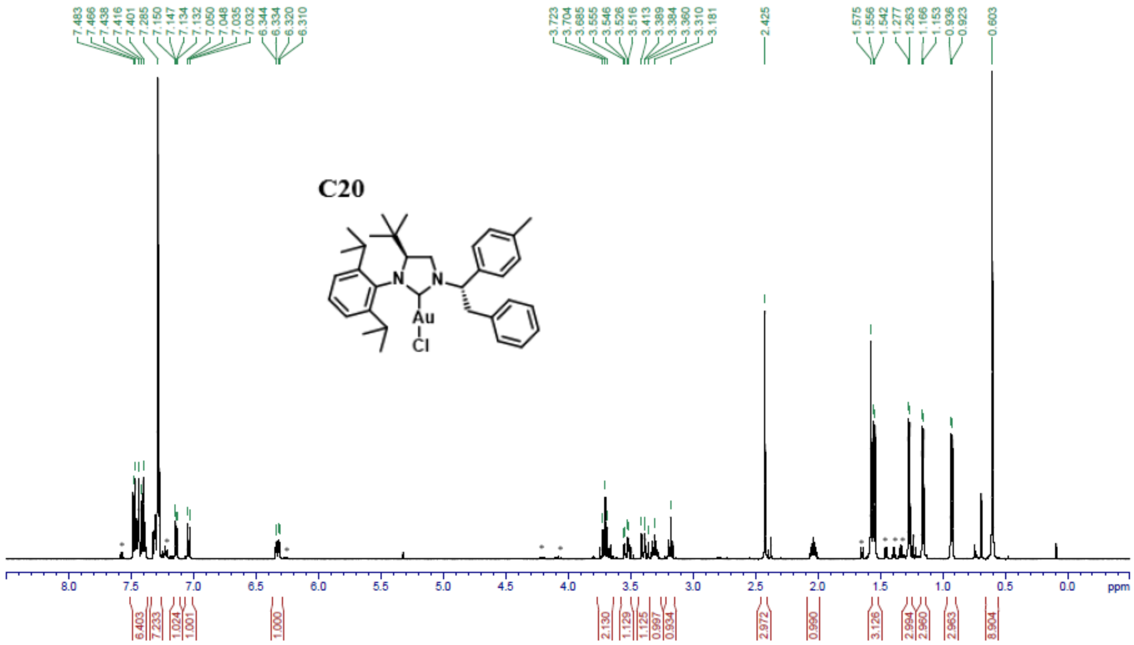

Figure S65. ^{1}H NMR (500 MHz) spectrum of **C20** in $CDCl_3$

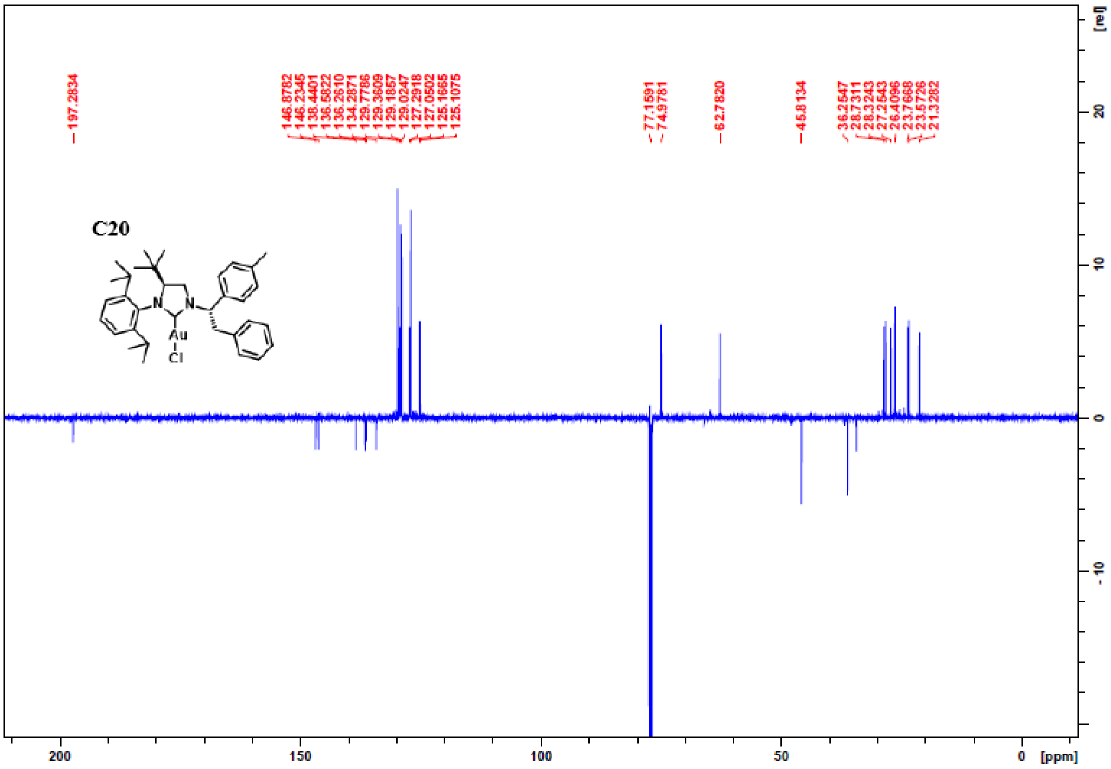

Figure S66. ^{13}C{1H} NMR (125 MHz) spectrum of **C20** in $CDCl_3$

(*4S*)-*4-tert*-Butyl-*3*-(*2',6'*-diisopropylphenyl)-*1*-[(*1R*)-*1*-(*2*-methoxyphenyl)-*2*-phenylethyl]imidazolidin-2-ylidene-gold-chloride (C21)

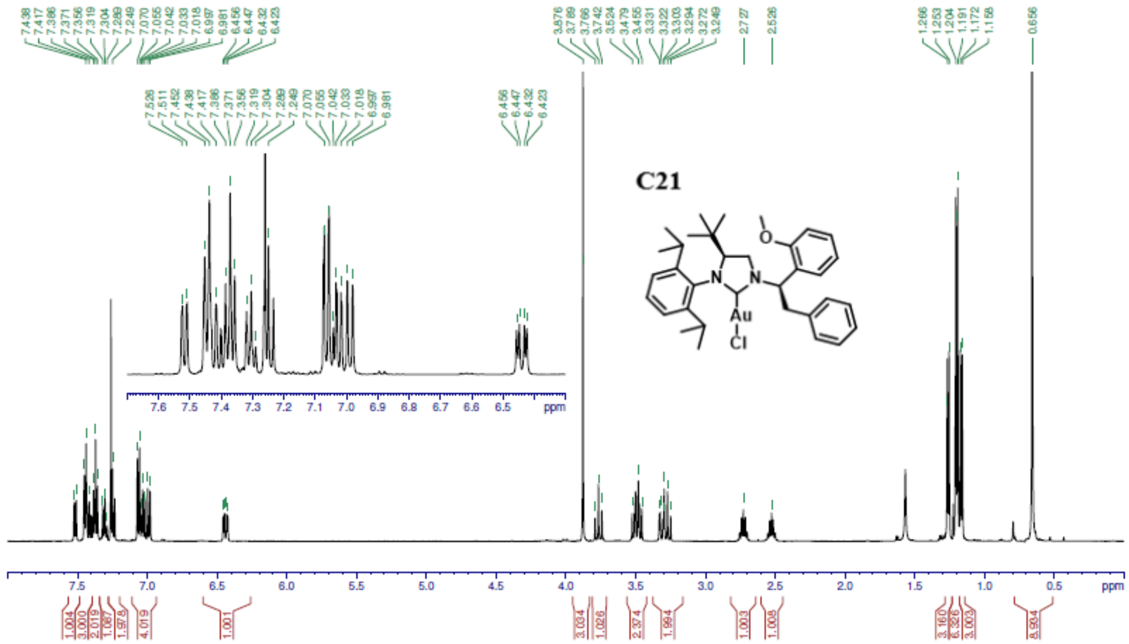

Figure S67. ^{1}H NMR (500 MHz) spectrum of **C21** in $CDCl_3$

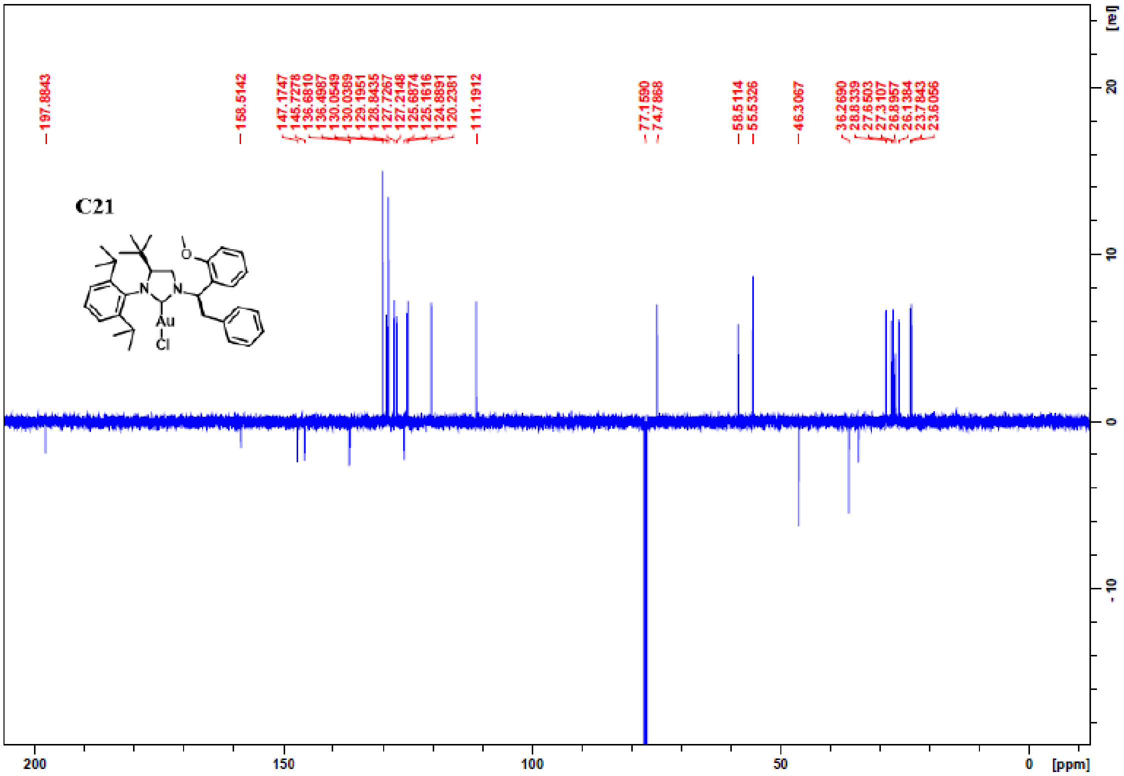

Figure S68. $^{13}C\{1H\}$ NMR (125 MHz) spectrum of **C21** in $CDCl_3$

(*4S*)-*4-tert*-Butyl-*3*-(*2',6'*-diisopropylphenyl)-*1*-[(*1S*)-*1*-(*2*-methoxyphenyl)-*2*-phenylethyl]imidazolidin-2-ylidene-gold-chloride (C22)

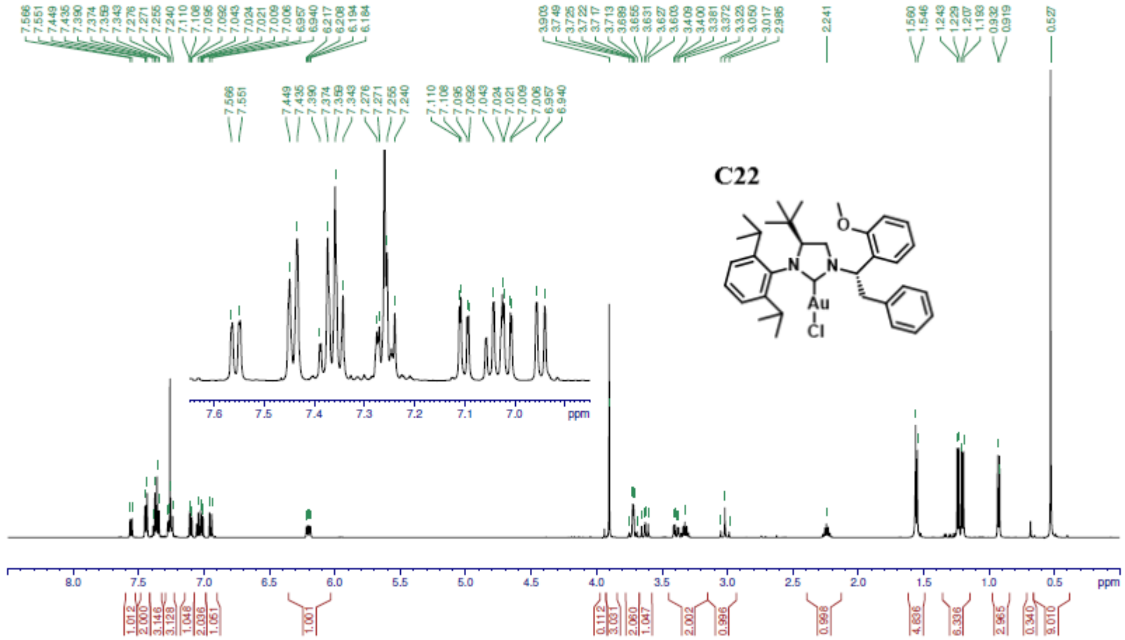

Figure S69. ^{1}H NMR (500 MHz) spectrum of **C22** in $CDCl_3$

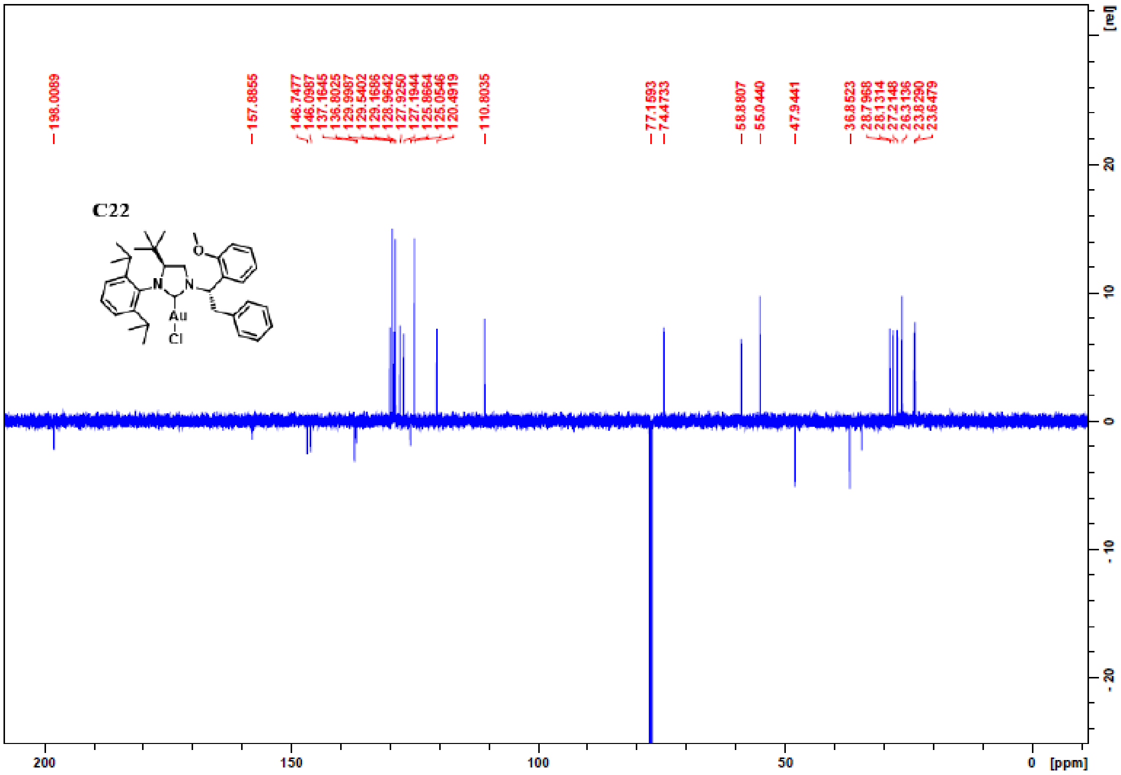

Figure S70. $^{13}C\{1H\}$ NMR (125 MHz) spectrum of **C22** in $CDCl_3$

(4*S*)-4-*tert*-Butyl-3-(2',6'-diisopropylphenyl)-1-[(1*R*)-1-(*o*-tolyl)-2-phenylethyl]imidazolidin-2-ylidene-gold-chloride (C23)

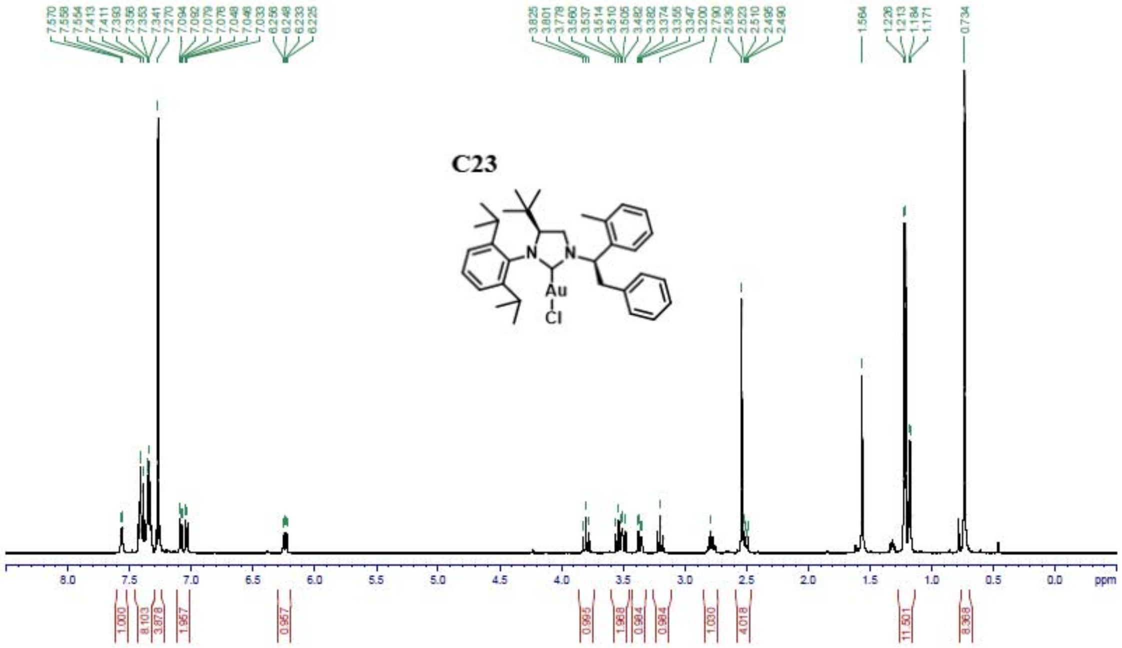

Figure S71. ^{1}H NMR (500 MHz) spectrum of **C23** in $CDCl_3$

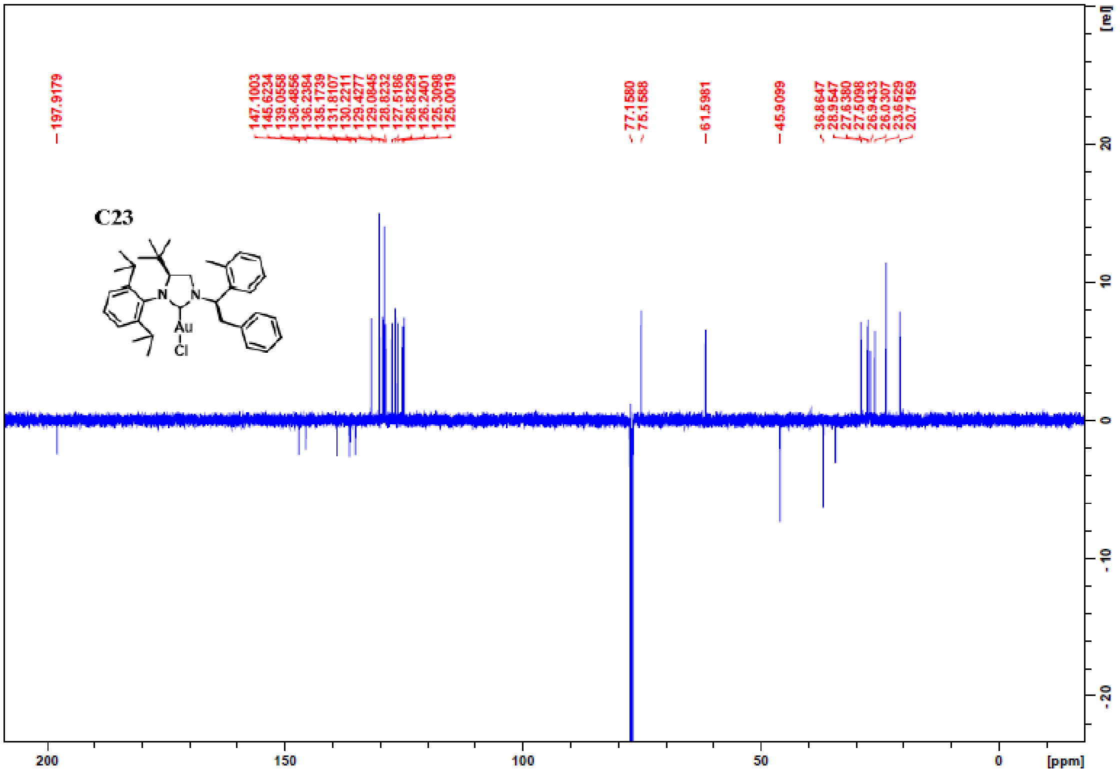

Figure S72. ^{13}C{1H} NMR (125 MHz) spectrum of **C23** in $CDCl_3$

(*4S*)-*4*-*tert*-Butyl-*3*-(*2'*,*6'*-diisopropylphenyl)-*1*-[(*1S*)-*1*-(*o*-tolyl)-*2*-phenylethyl]imidazolidin-2-ylidene-gold-chloride (C24)

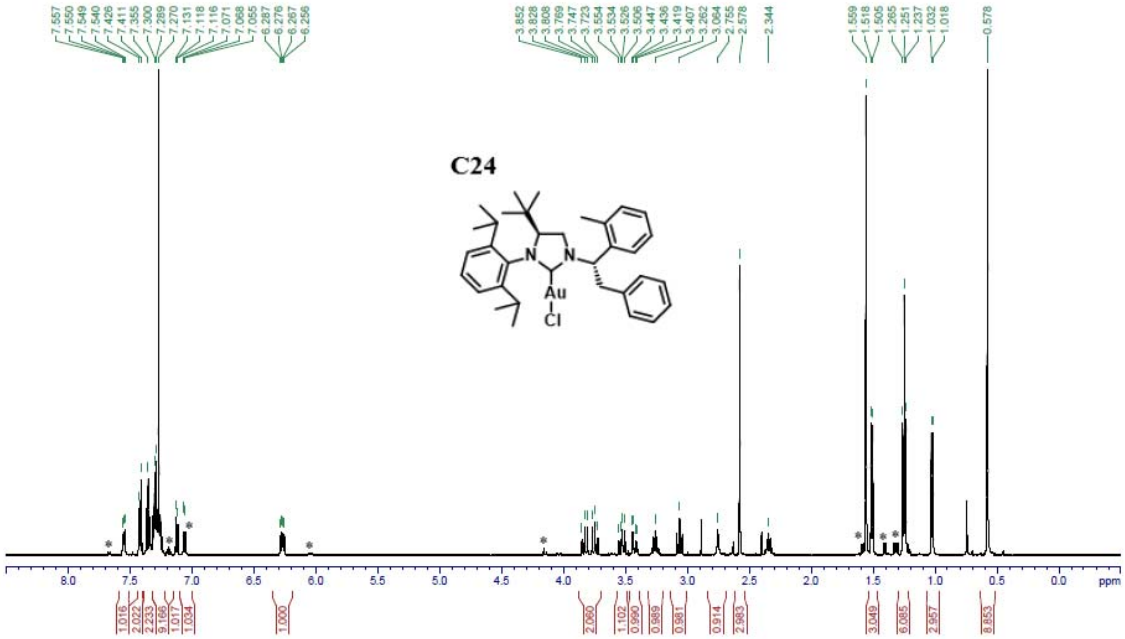

Figure S73. ^{1}H NMR (500 MHz) spectrum of **C24** in $CDCl_3$

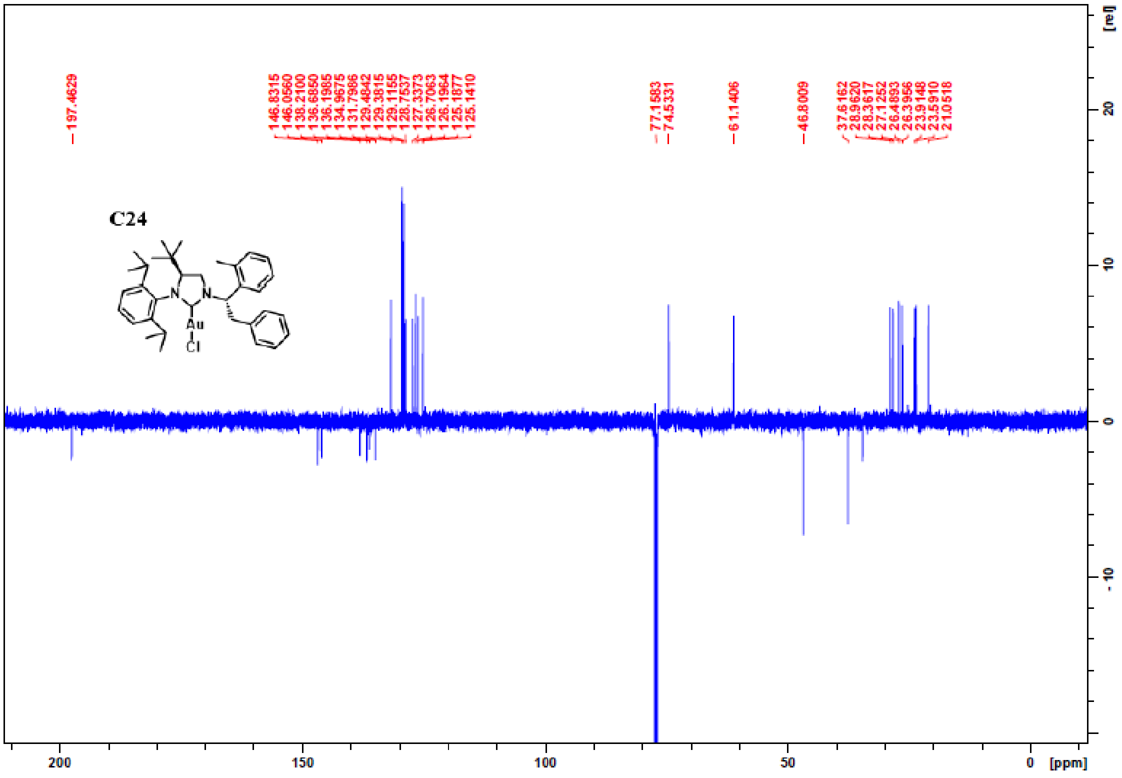

Figure S74. ^{13}C{1H} NMR (125 MHz) spectrum of **C24** in $CDCl_3$

(*4S*)-*4*-*tert*-Butyl-*3*-(*2'*,*6'*-diisopropylphenyl)-*1*-[(*1R*)-*1*-(*3*-methoxyphenyl)-*2*-phenylethyl]imidazolidin-2-ylidene-gold-chloride (C25)

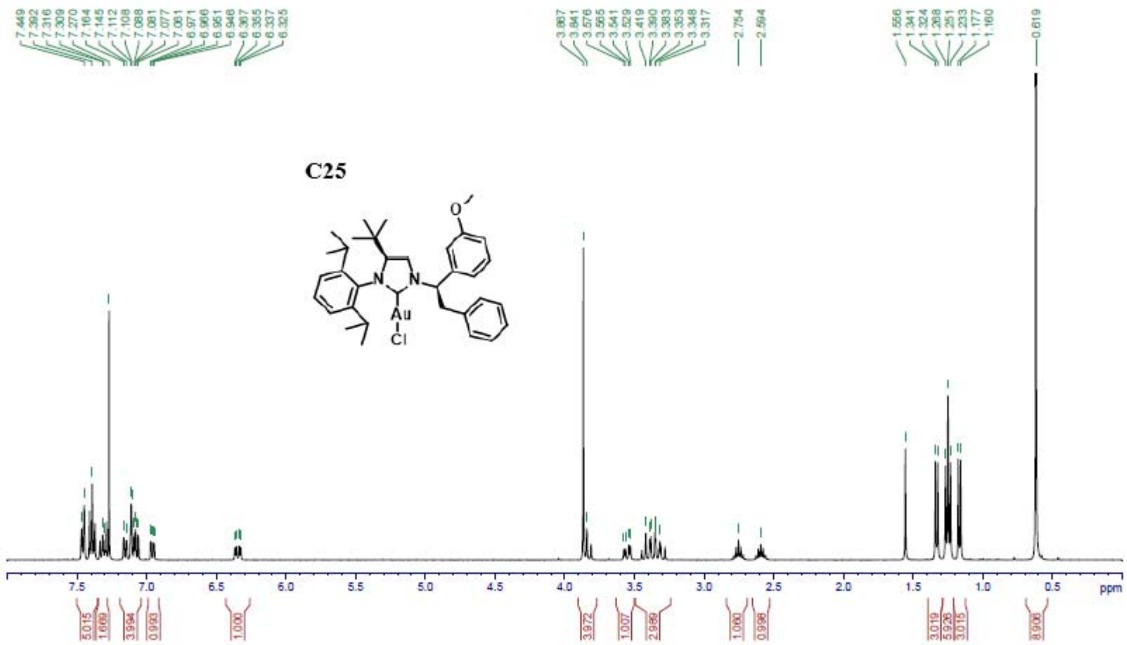

Figure S75. ^{1}H NMR (400 MHz) spectrum of **C25** in $CDCl_3$

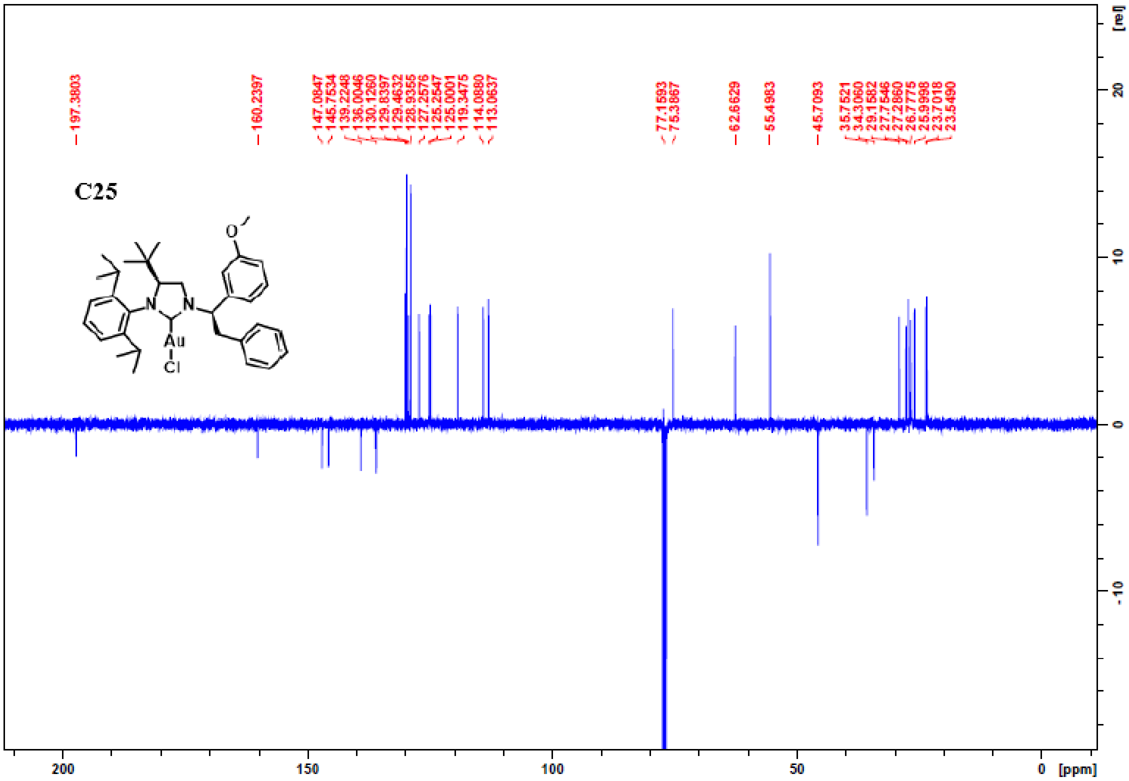

Figure S76. $^{13}C\{1H\}$ NMR (100 MHz) spectrum of **C25** in $CDCl_3$

(*4S*)-*4*-*tert*-Butyl-*3*-(*2'*,*6'*-diisopropylphenyl)-*1*-[(*1S*)-*1*-(*3*-methoxyphenyl)-*2*-phenylethyl]imidazolidin-2-ylidene-gold-chloride (C26)

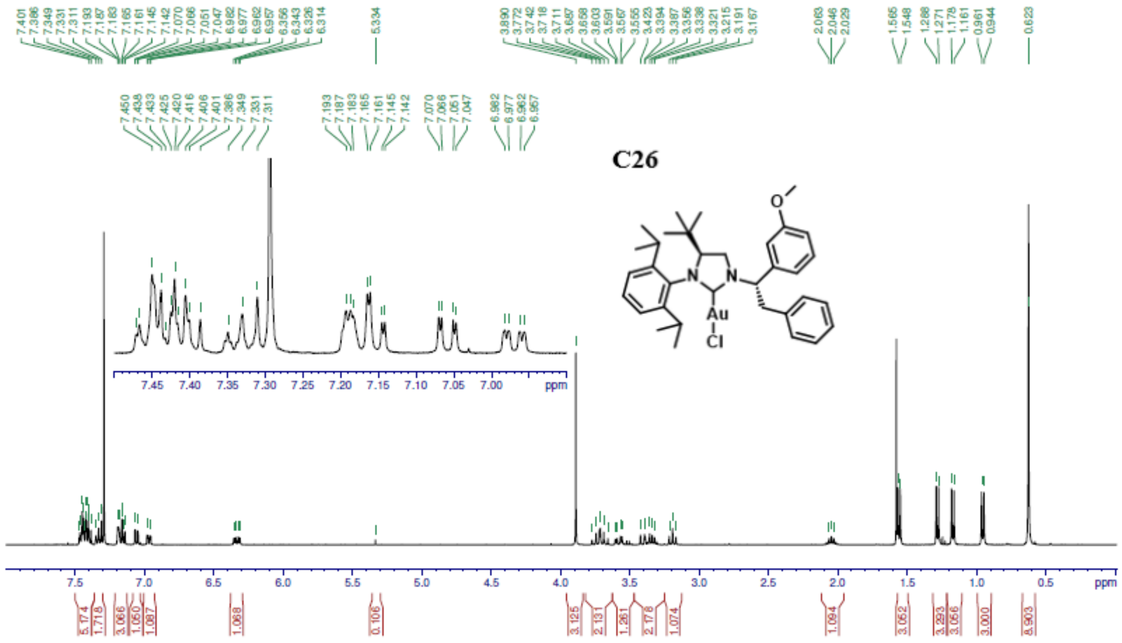

Figure S77. ^{1}H NMR (400 MHz) spectrum of **C26** in $CDCl_3$

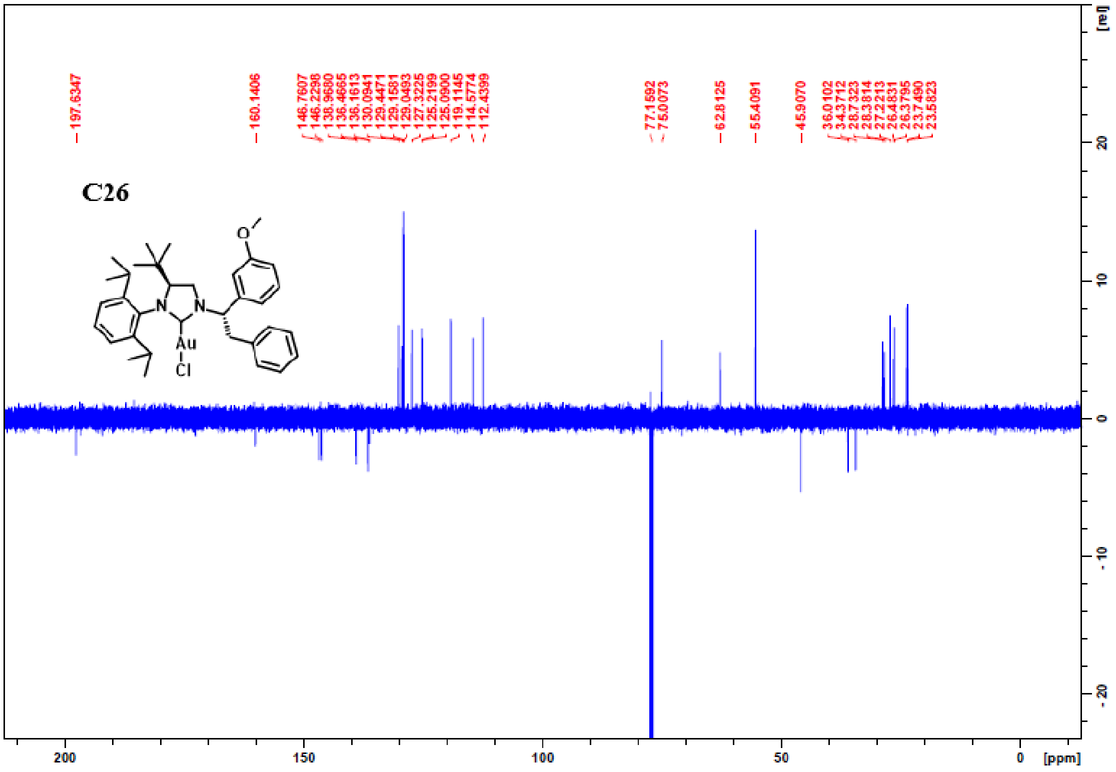

Figure S78. ^{13}C{1H} NMR (100 MHz) spectrum of **C26** in $CDCl_3$

(4*S*)-4-*tert*-Butyl-3-(2',6'-diisopropylphenyl)-1-[(1*R*)-2-(*p*-tolyl)-1-phenylethyl]imidazolidin-2-ylidene-gold-chloride (C27)

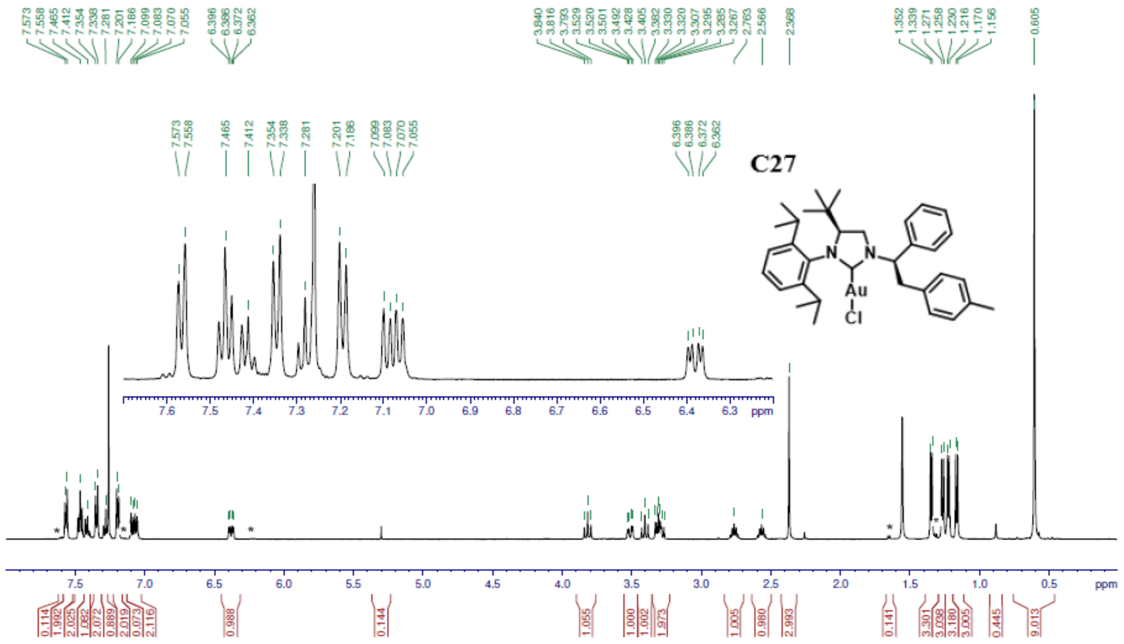

Figure S79. ^{1}H NMR (500 MHz) spectrum of **C27** in $CDCl_3$

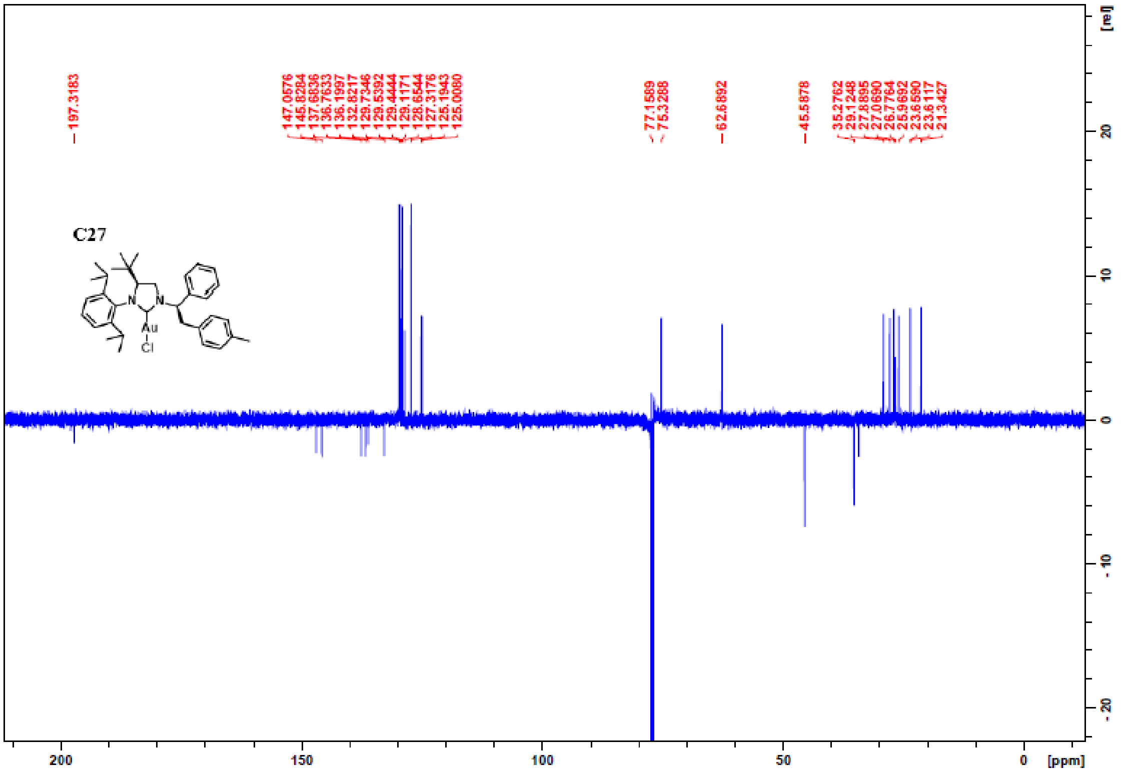

Figure S80. $^{13}C\{1H\}$ NMR (125 MHz) spectrum of **C27** in $CDCl_3$

(*4S*)-*4-tert*-Butyl-*3*-(*2’,6’*-diisopropylphenyl)-*1*-[(*1S*)-*2*-(*p*-tolyl)-*1*-phenylethyl]imidazolidin-2-ylidene-gold-chloride (C28)

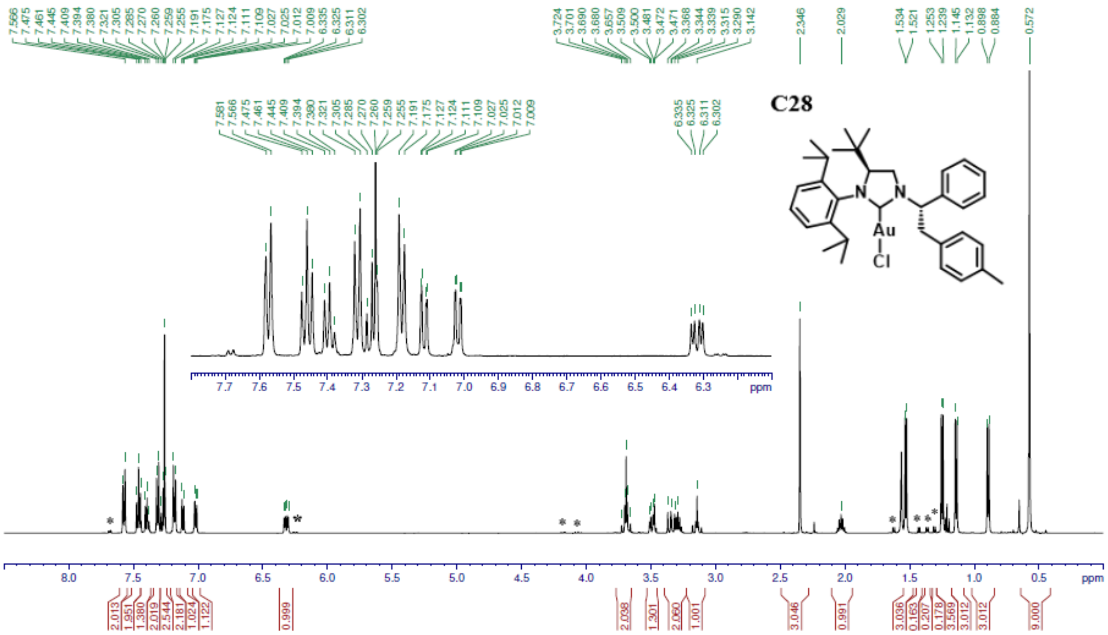

Figure S81. ^{1}H NMR (500 MHz) spectrum of **C28** in $CDCl_3$

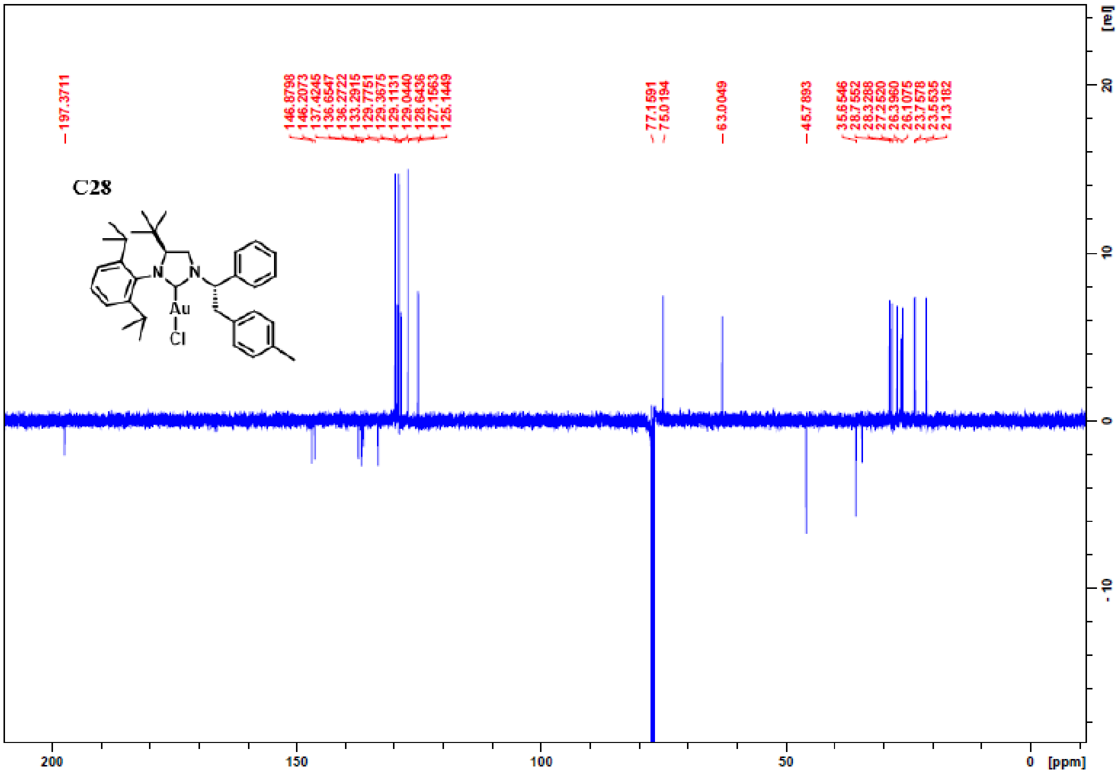

Figure S82. ^{13}C{1H} NMR (125 MHz) spectrum of **C28** in $CDCl_3$

(*4S*)-*4*-*tert*-Butyl-*3*-(*2'*,*6'*-diisopropylphenyl)-*1*-[(*1R*)-*2*-(*4*-chlorophenyl)-*1*-phenylethyl]imidazolidin-2-ylidene-gold-chloride (C29)

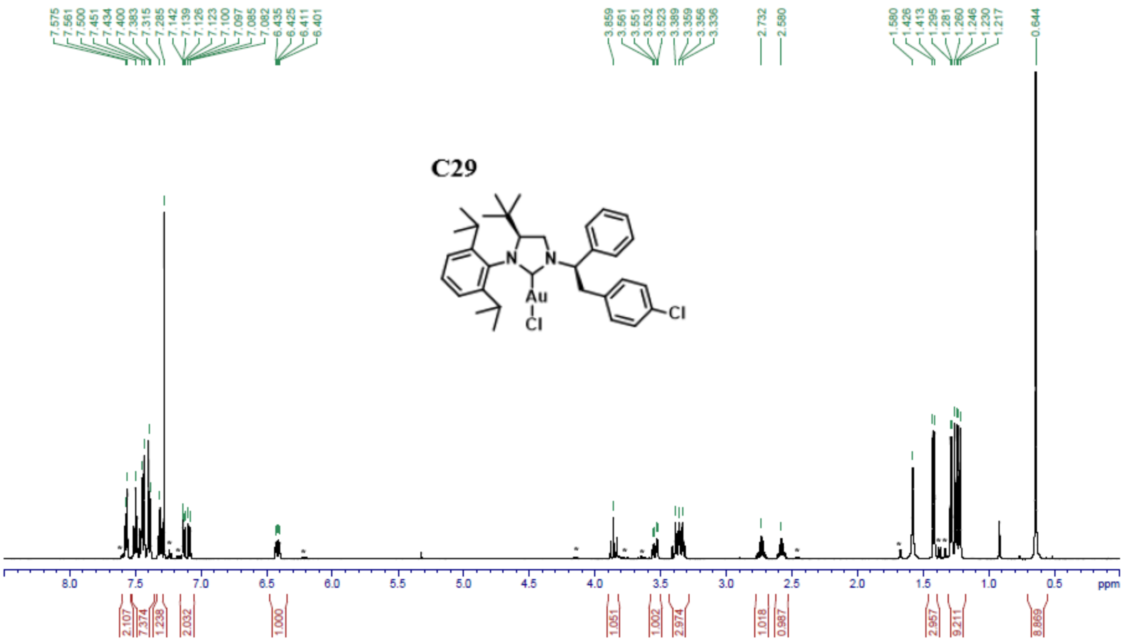

Figure S83. ^{1}H NMR (500 MHz) spectrum of **C29** in $CDCl_3$

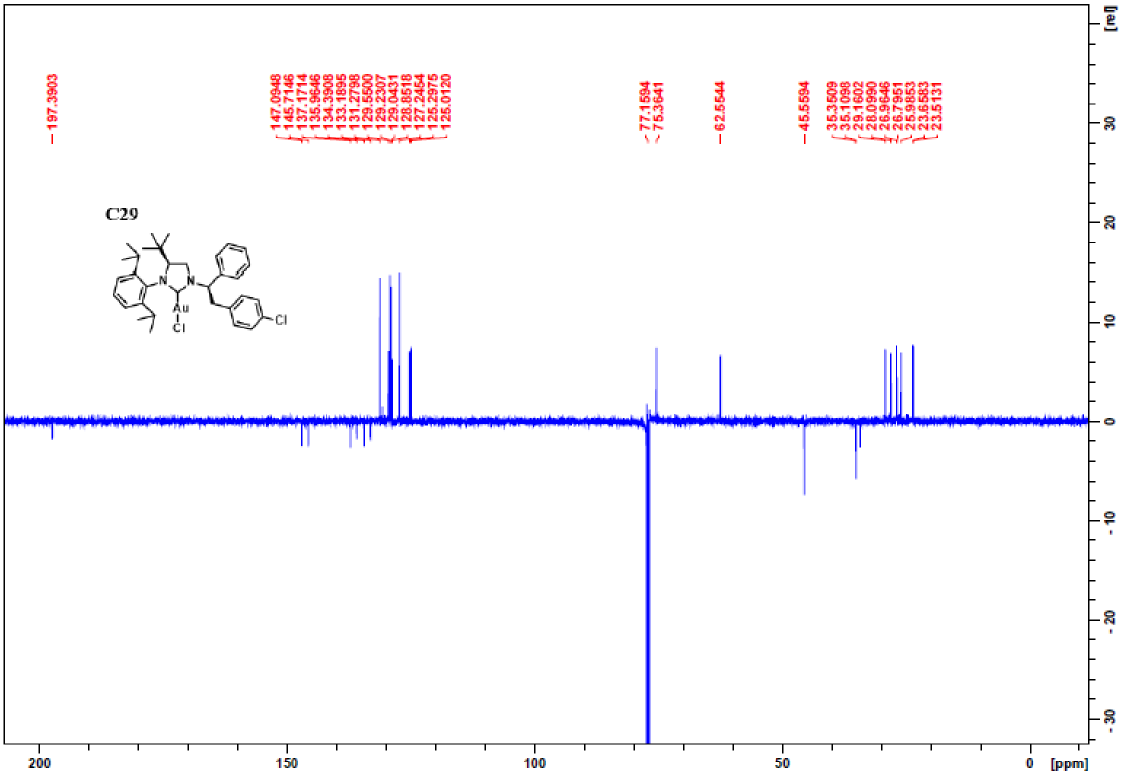

Figure S84. $^{13}C\{1H\}$ NMR (125 MHz) spectrum of **C29** in $CDCl_3$

(*4S*)-*4*-*tert*-Butyl-*3*-(*2',6'*-diisopropylphenyl)-*1*-[(*1S*)-*2*-(*4*-chlorophenyl)-*1*-phenylethyl]imidazolidin-2-ylidene-gold-chloride (C30)

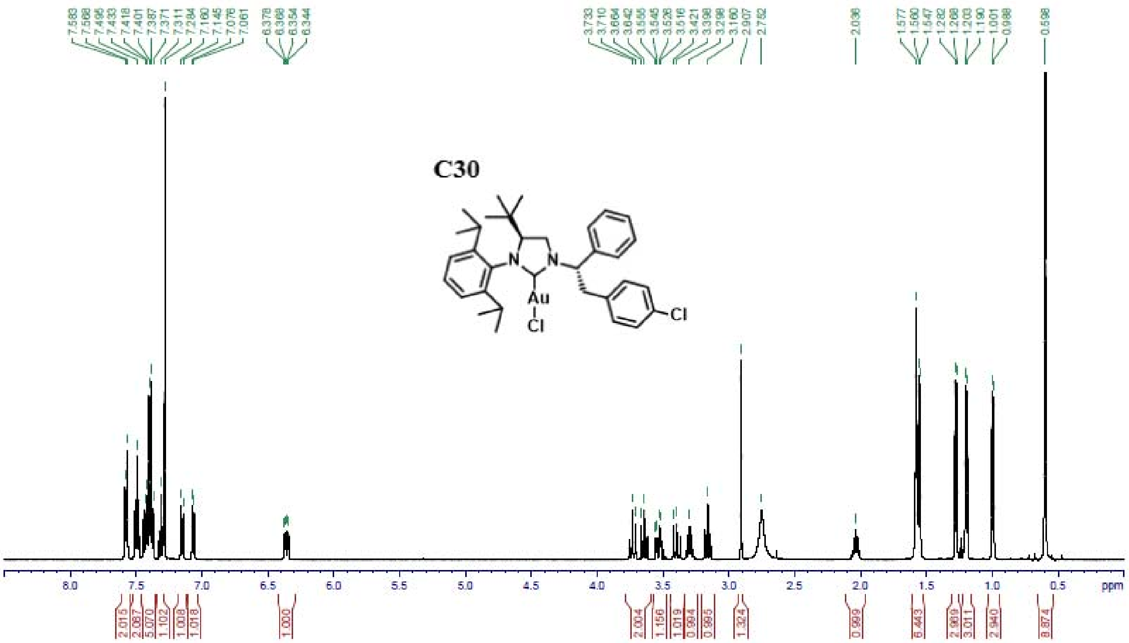

Figure S85. ^{1}H NMR (500 MHz) spectrum of **C30** in $CDCl_3$

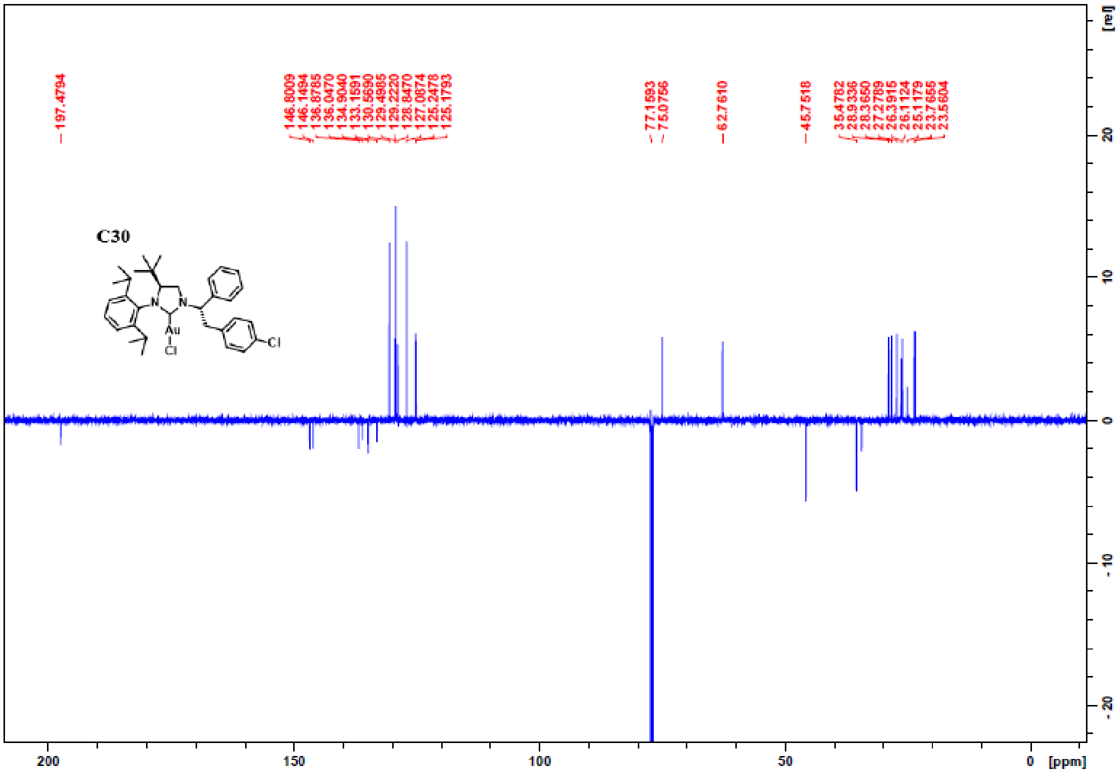

Figure S86. $^{13}C\{1H\}$ NMR (125 MHz) spectrum of **C30** in $CDCl_3$

(*4S*)-*4-tert*-Butyl-*3*-(*2',6'*-diisopropylphenyl)-*1*-[(*1R*)-*2*-(*2*-chlorophenyl)-*1*-phenylethyl]imidazolidin-2-ylidene-gold-chloride (C31)

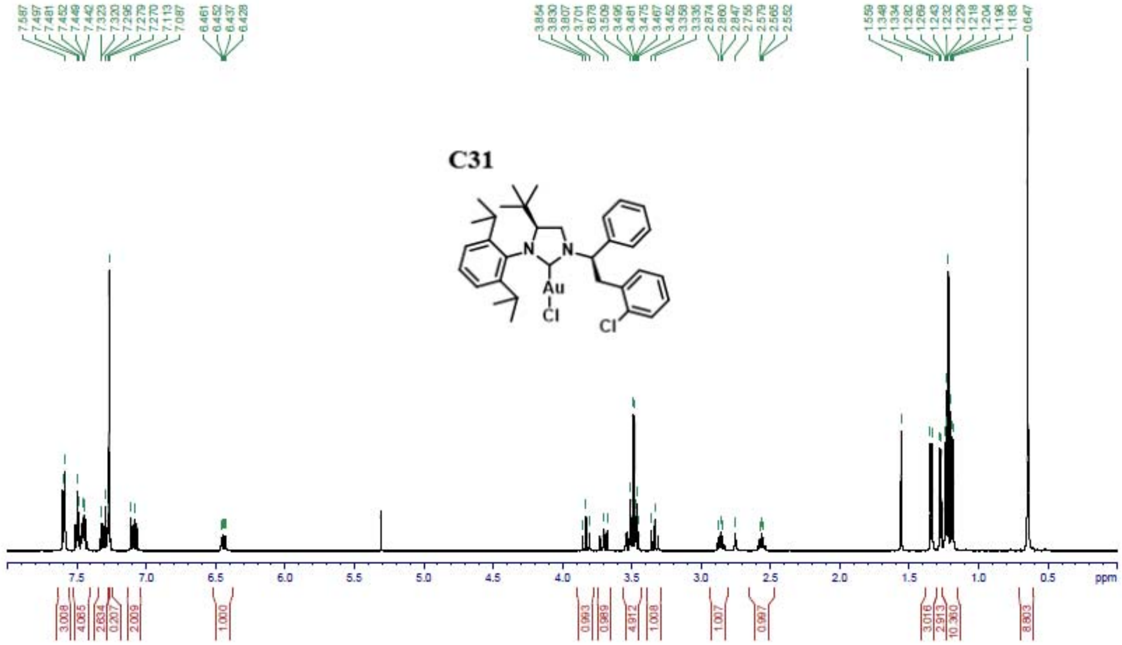

Figure S87. ^{1}H NMR (500 MHz) spectrum of **C31** in $CDCl_3$

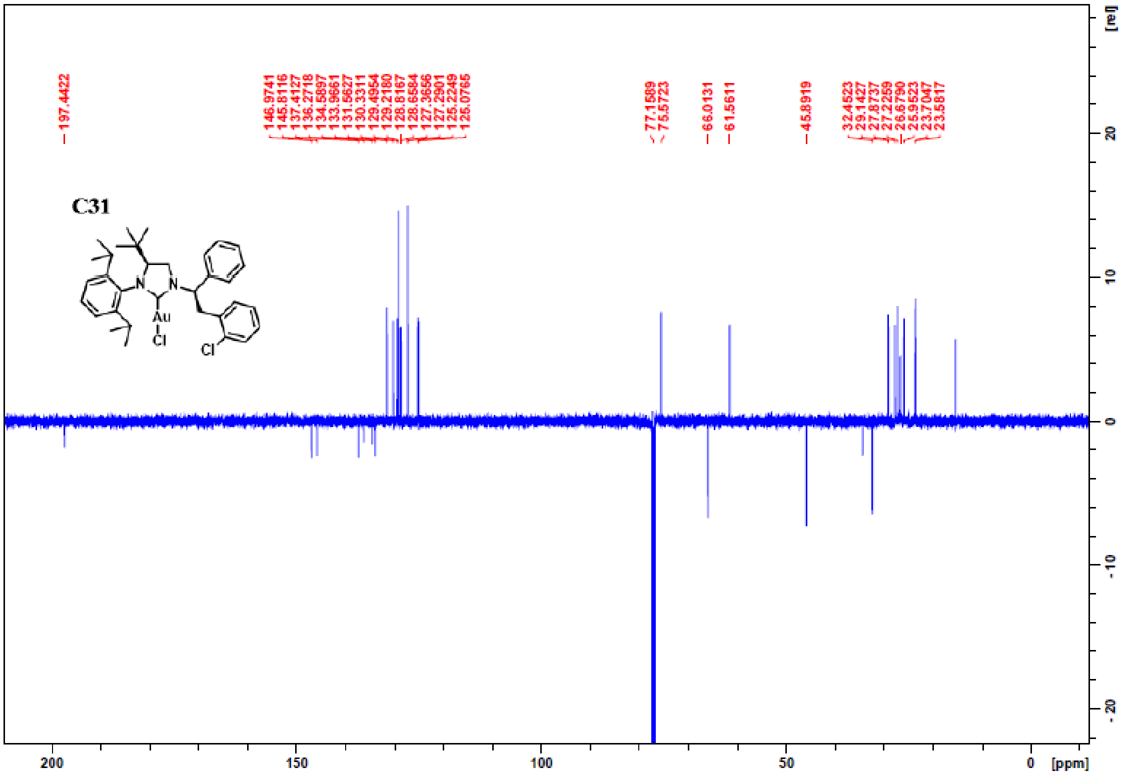

Figure S88. ^{13}C{1H} NMR (125 MHz) spectrum of **C31** in $CDCl_3$

(*4S*)-*4-tert*-Butyl-*3*-(*2',6'*-diisopropylphenyl)-*1*-[(*1S*)-*2*-(*2*-chlorophenyl)-*1*-phenylethyl]imidazolidin-2-ylidene-gold-chloride (C32)

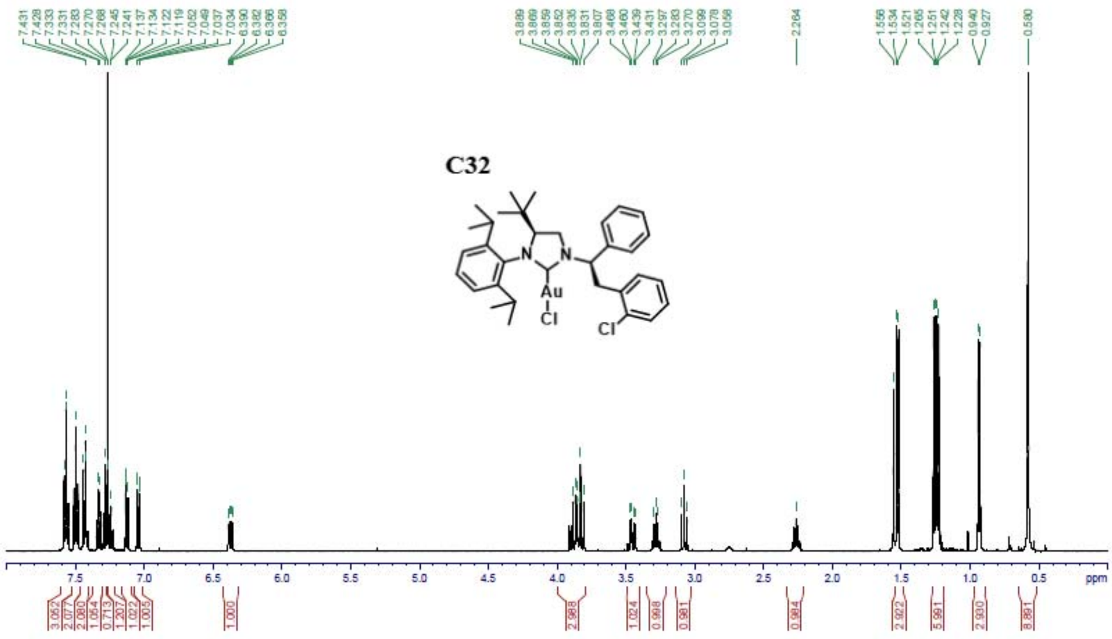

Figure S89. ^{1}H NMR (500 MHz) spectrum of **C32** in $CDCl_3$

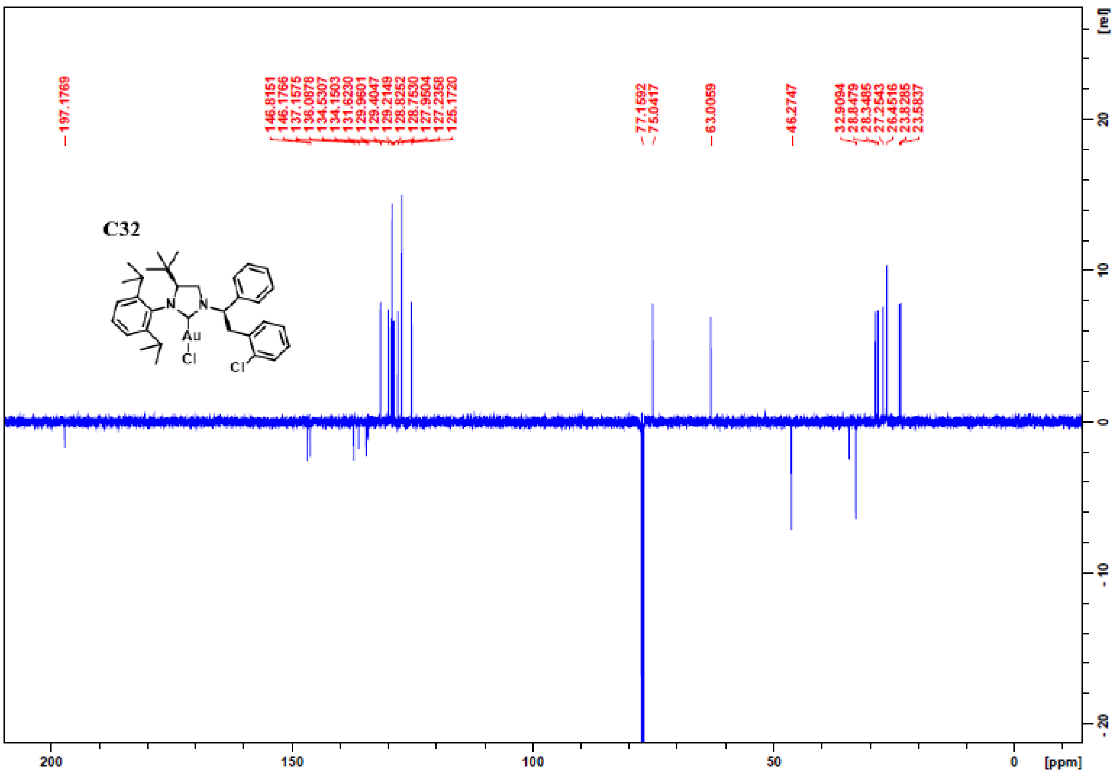

Figure S90. $^{13}C\{1H\}$ NMR (125 MHz) spectrum of **C32** in $CDCl_3$

(*4R*)-*4*-*tert*-Butyl-*3*-(*2'*,*6'*-diisopropylphenyl)-*1*-[(*1R*)-*2*-(*3*-chlorophenyl)-*1*-phenylethyl]imidazolidin-2-ylidene-gold-chloride (C35)

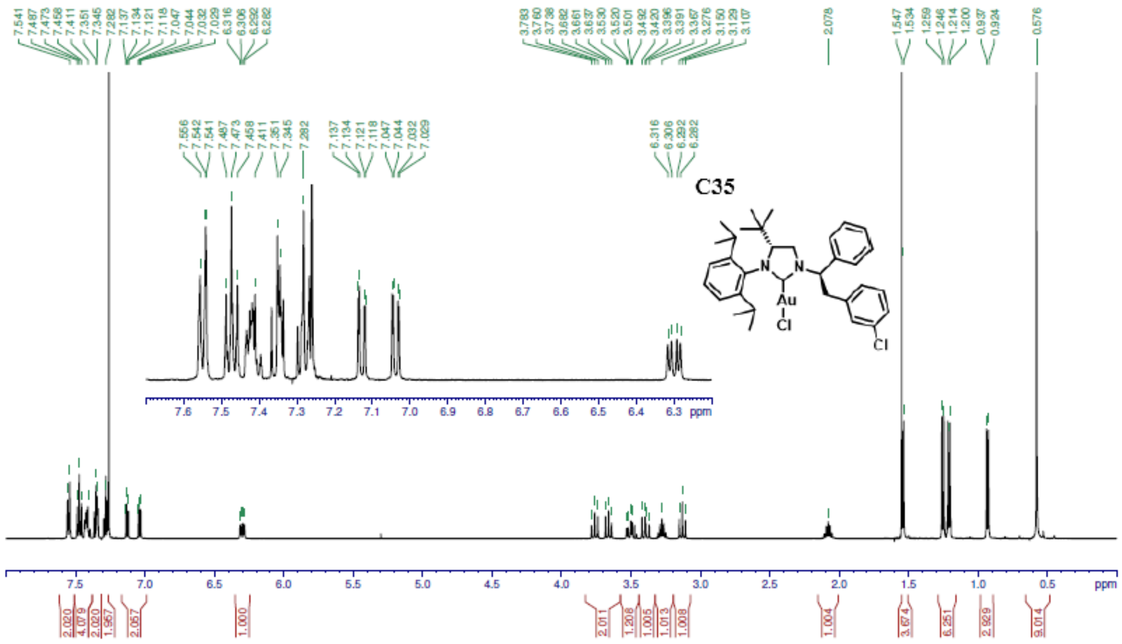

Figure S91. ^{1}H NMR (500 MHz) spectrum of **C35** in $CDCl_3$

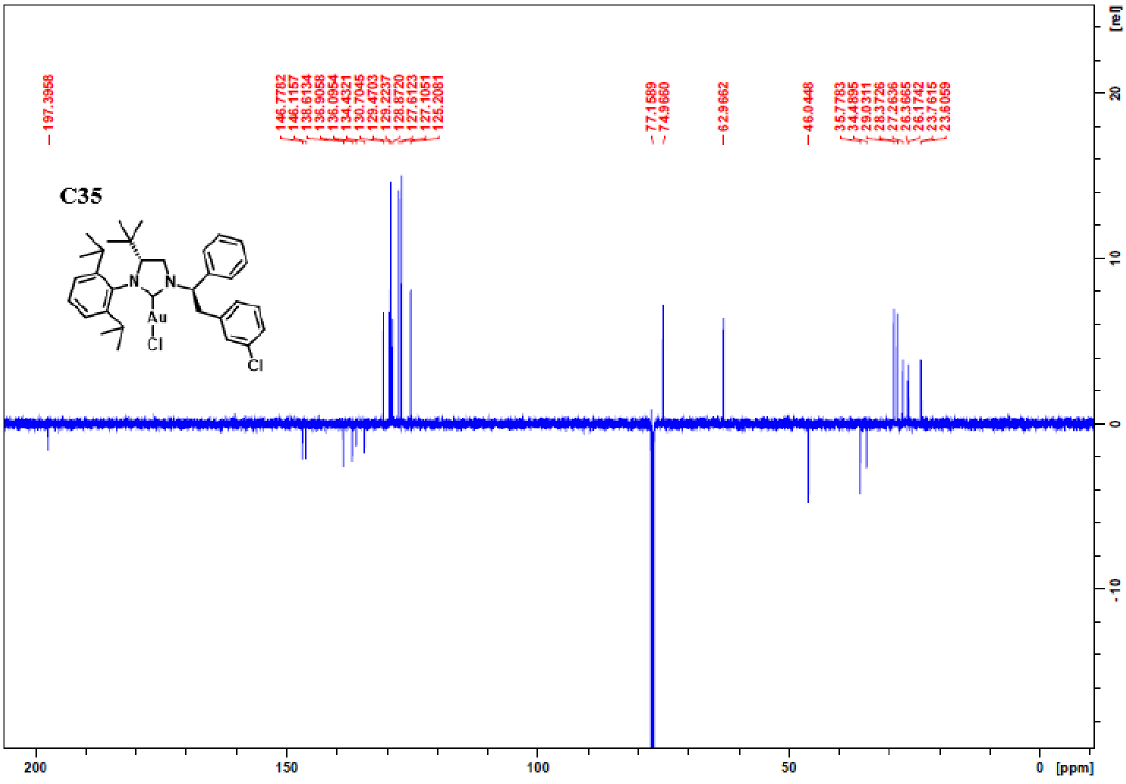

Figure S92. ^{13}C{1H} NMR (125 MHz) spectrum of **C35** in $CDCl_3$

(*4R*)-*4-tert*-Butyl-*3*-(*2',6'*-diisopropylphenyl)-*1*-[(*1S*)-*2*-(*3*-chlorophenyl)-*1*-phenylethyl]imidazolidin-2-ylidene-gold-chloride (C36)

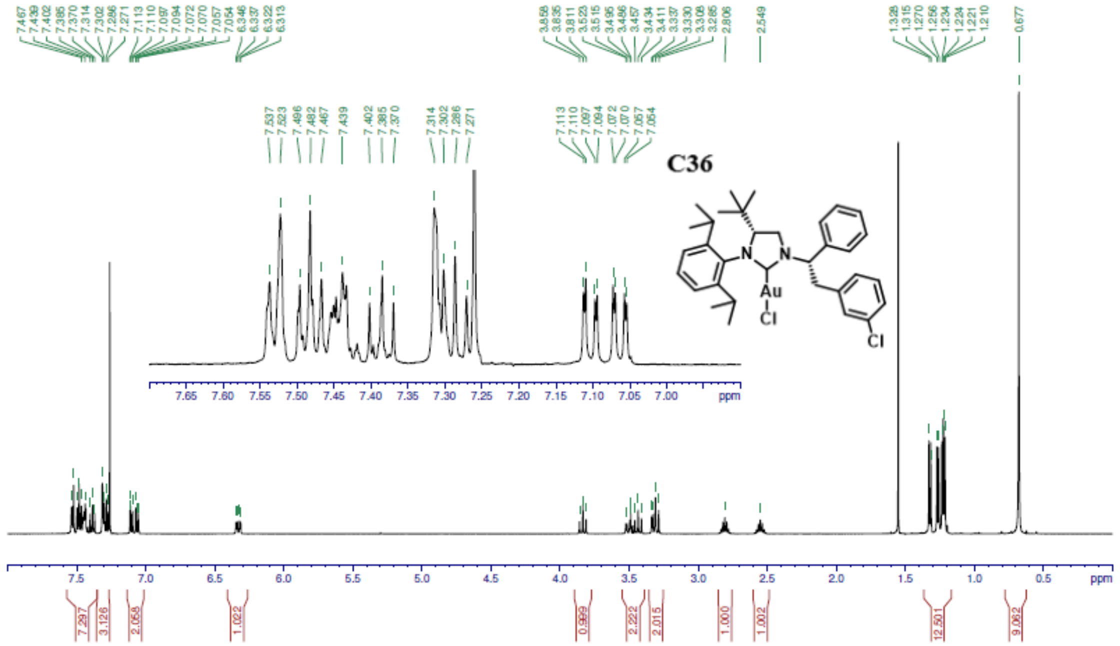

Figure S93. ^{1}H NMR (500 MHz) spectrum of **C36** in $CDCl_3$

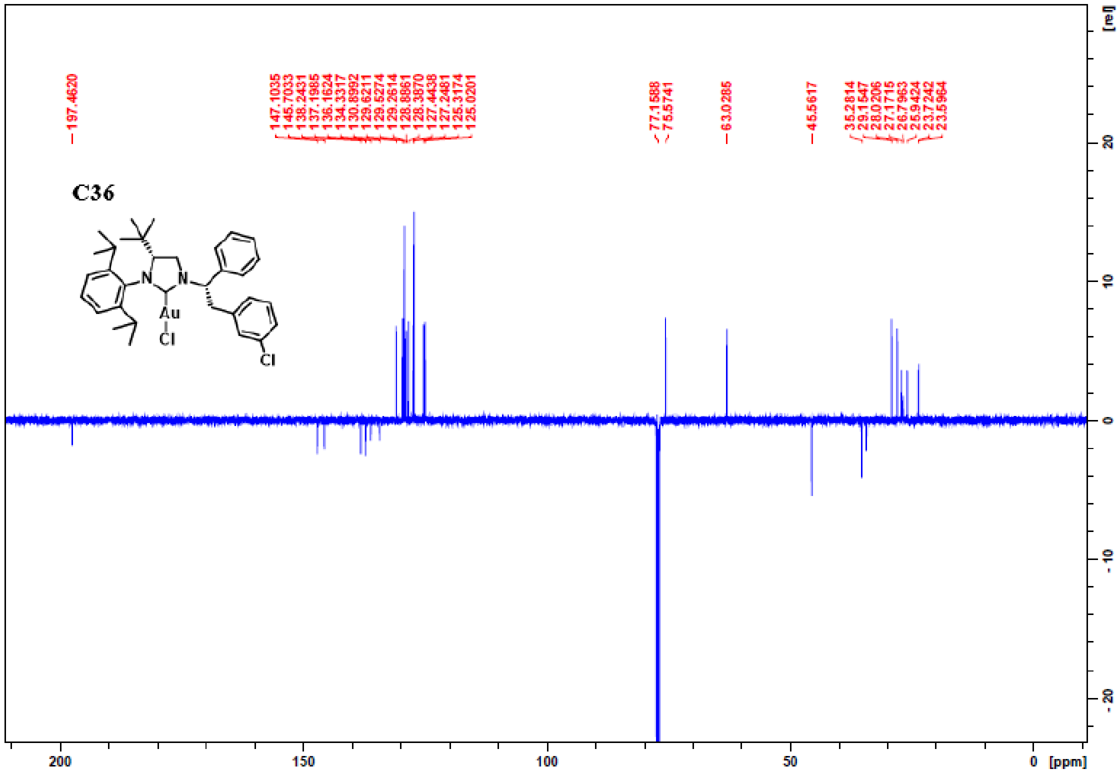

Figure S94. ^{13}C{1H} NMR (125 MHz) spectrum of **C36** in $CDCl_3$

(*4R*)-*4*-*tert*-Butyl-*3*-(*2',6'*-diisopropylphenyl)-*1*-[(*1R*)-*1*-(*3*-trifluoromethyl)-*2*-phenylethyl]imidazolidin-2-ylidene-gold-chloride (C37)

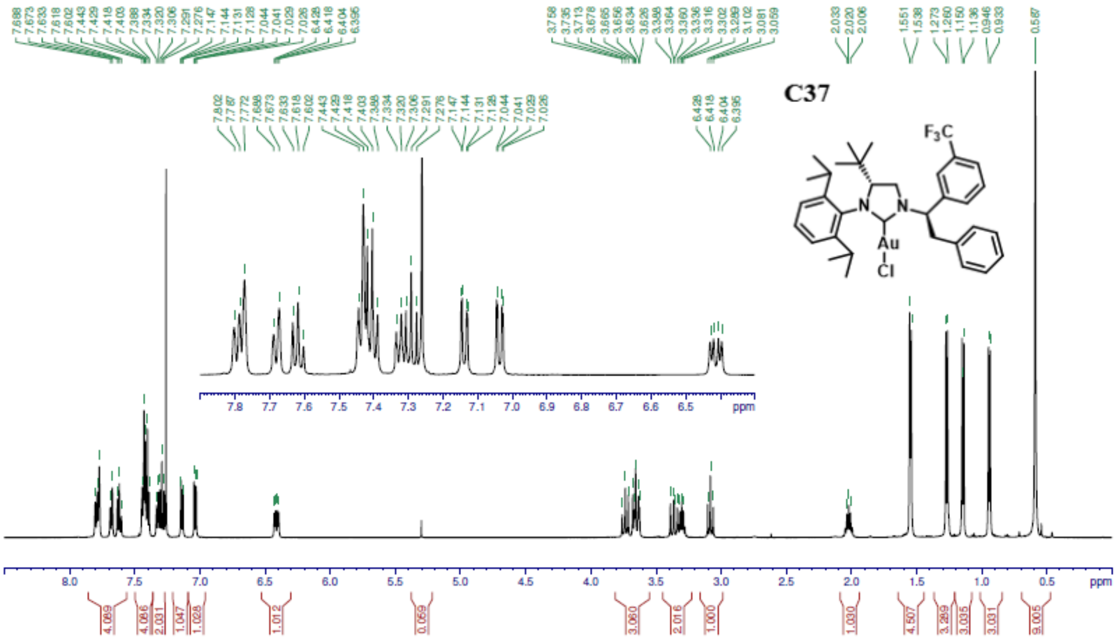

Figure S95. ^{1}H NMR (500 MHz) spectrum of **C37** in $CDCl_3$

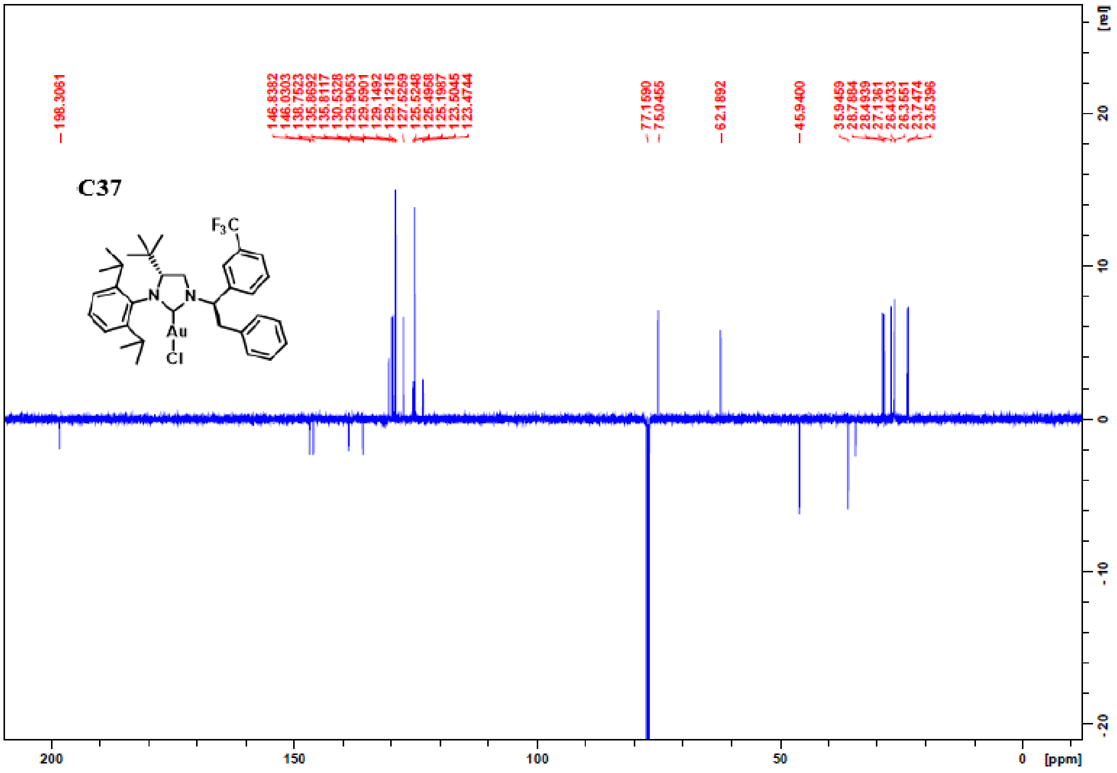

Figure S96. $^{13}C\{1H\}$ NMR (125 MHz) spectrum of **C37** in $CDCl_3$

(*4R*)-*4-tert*-Butyl-*3*-(*2',6'*-diisopropylphenyl)-*1*-[(*1S*)-*1*-(*3*-trifluoromethyl)-*2*-phenylethyl]imidazolidin-2-ylidene-gold-chloride (C38)

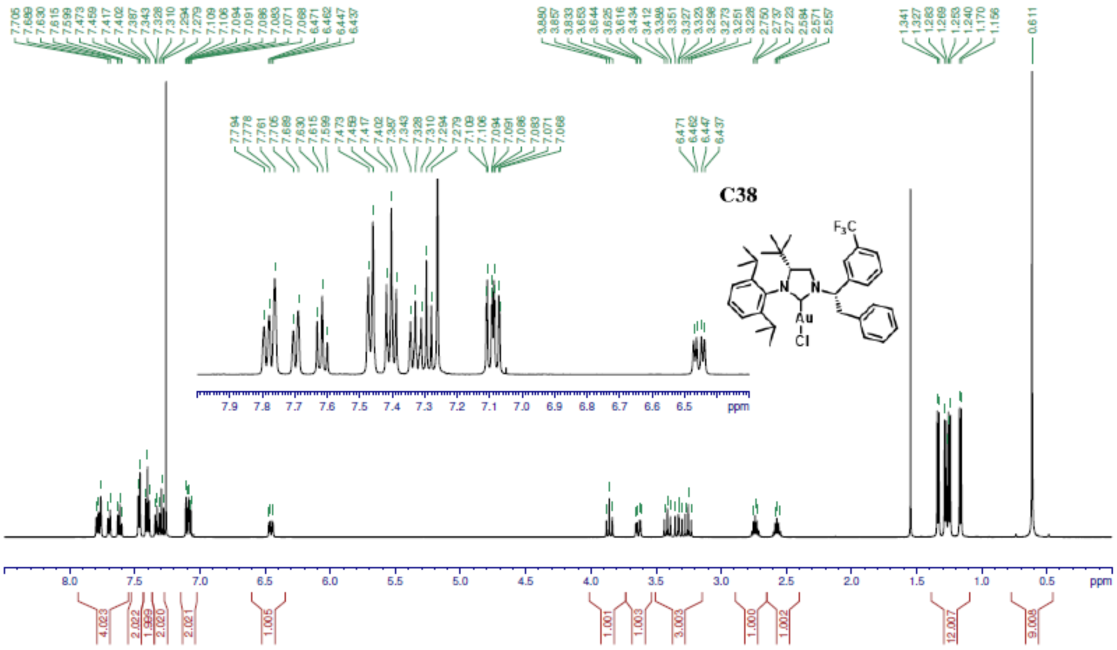

Figure S97. ^{1}H NMR (500 MHz) spectrum of **C38** in $CDCl_3$

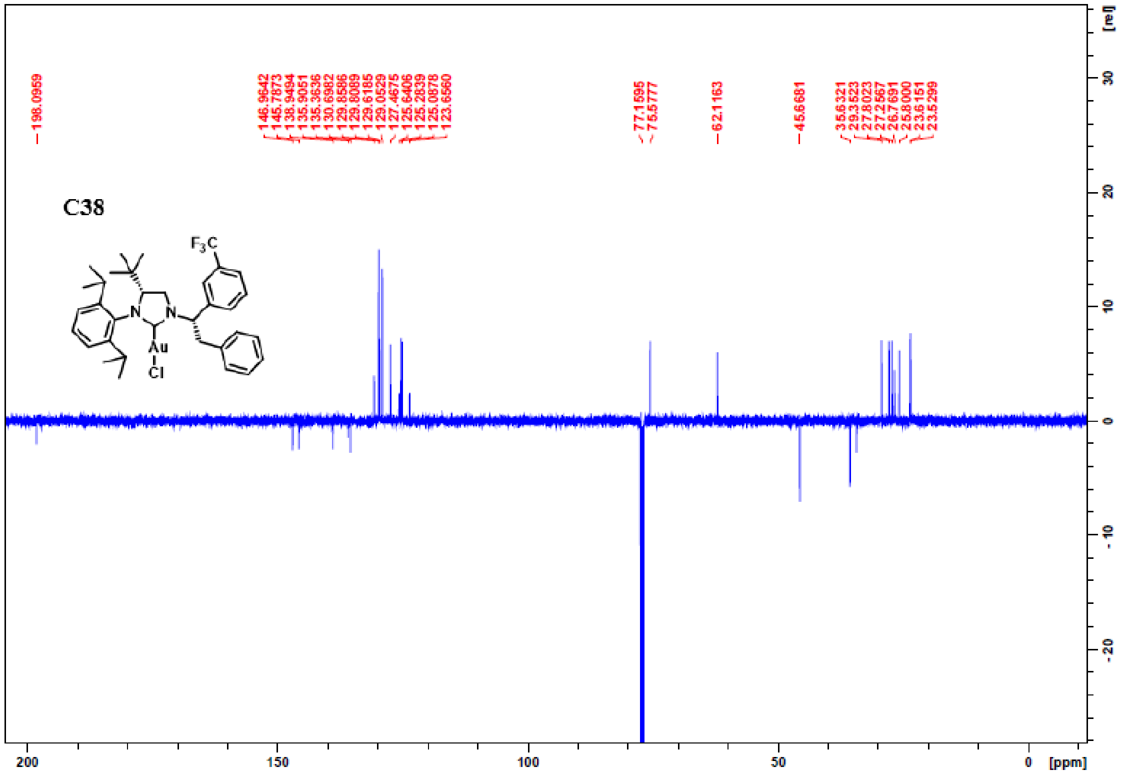

Figure S98. $^{13}C\{1H\}$ NMR (125 MHz) spectrum of **C38** in $CDCl_3$

***3*-(*2’*,*6’*-diisopropylphenyl)-*1*-[(*1R*)-*2*-(*4*-chlorophenyl)-*1*-phenylethyl]imidazolidin-2-ylidene-gold-chloride (C39)**

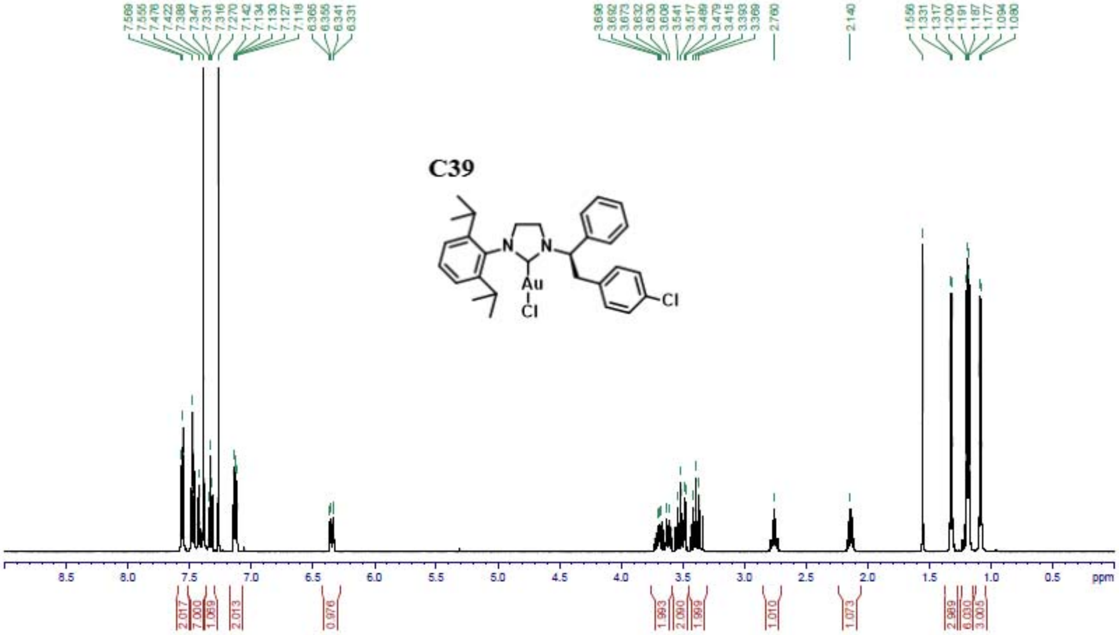

Figure S99. ^{1}H NMR (500 MHz) spectrum of **C39** in $CDCl_3$

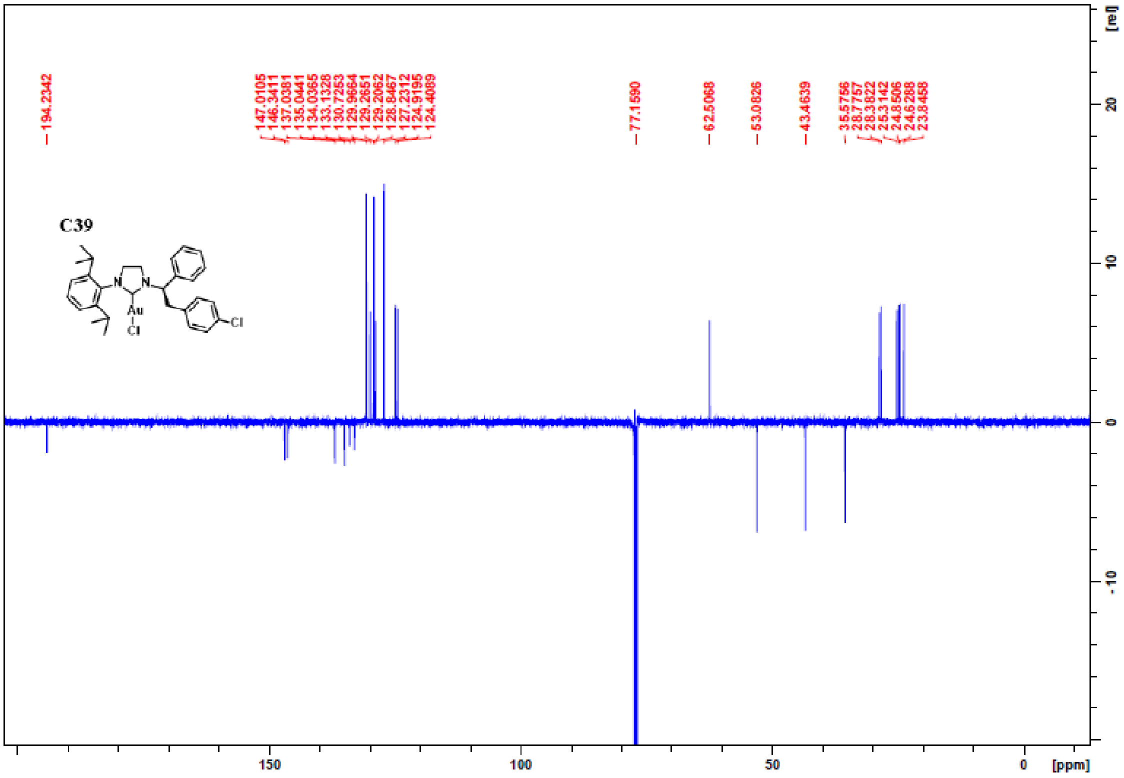

Figure S100. $^{13}C\{1H\}$ NMR (125 MHz) spectrum of **C39** in $CDCl_3$

***3*-(*2'*,*6'*-diisopropylphenyl)-*1*-[(*1S*)-*2*-(*4*-chlorophenyl)-*1*-phenylethyl]imidazolidin-2-ylidene-gold-chloride (C40)**

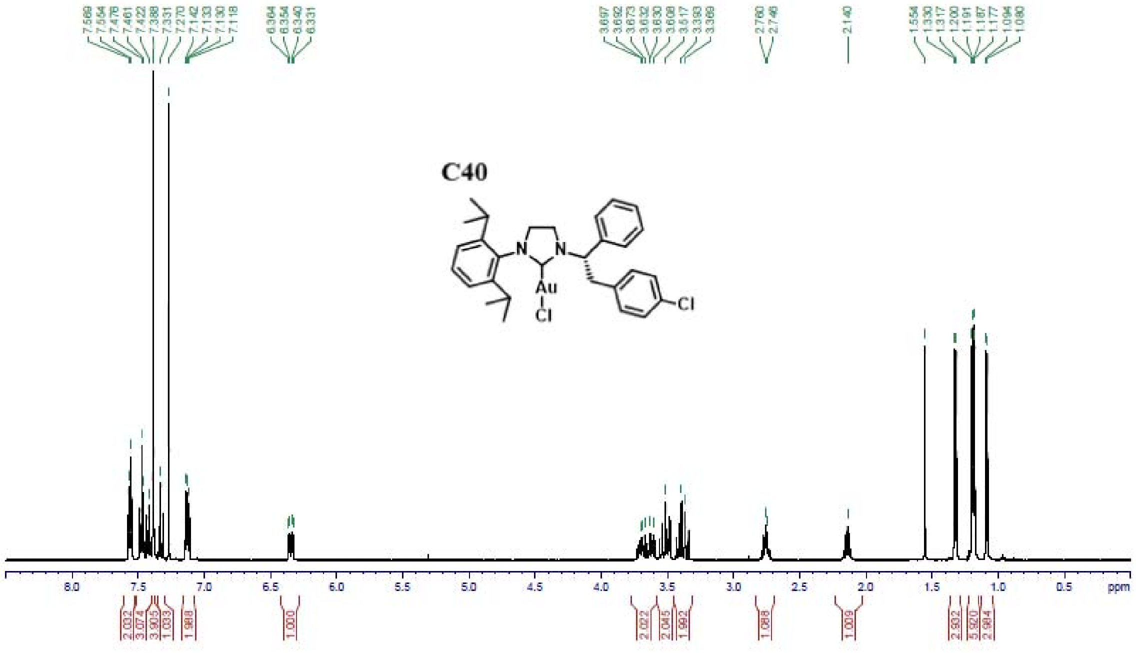

Figure S101. ^{1}H NMR (500 MHz) spectrum of **C40** in $CDCl_3$

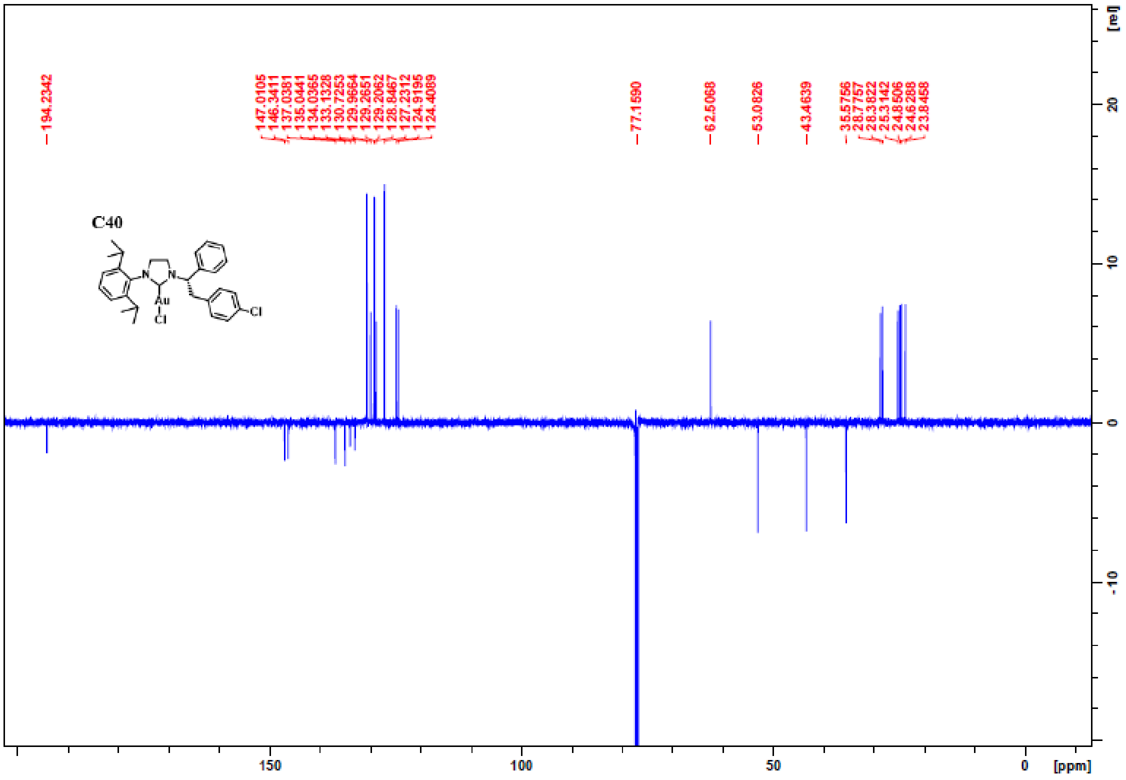

Figure S102. $^{13}C\{1H\}$ NMR (125 MHz) spectrum of **C40** in $CDCl_3$

***3*-(*2'*,*6'*-diisopropylphenyl)-*1*-[(*1S*)-*1*-benzyl-*2*-hydroxiethyl]imidazolidin-2-ylidene-gold-chloride (C41)**

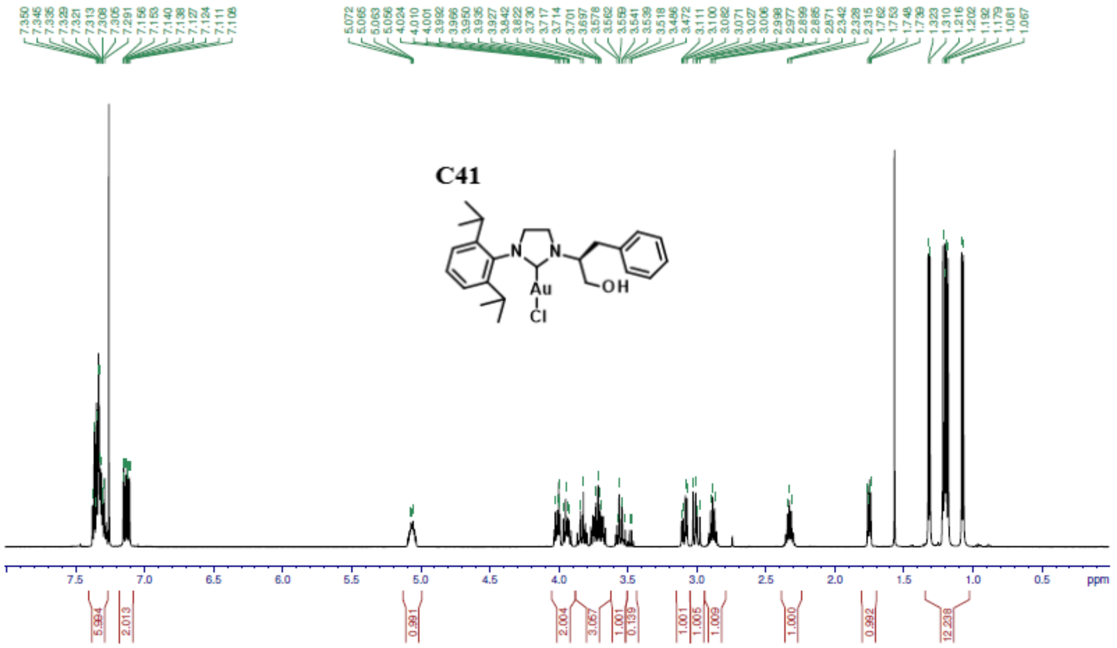

Figure S103. ^{1}H NMR (500 MHz) spectrum of **C41** in $CDCl_3$

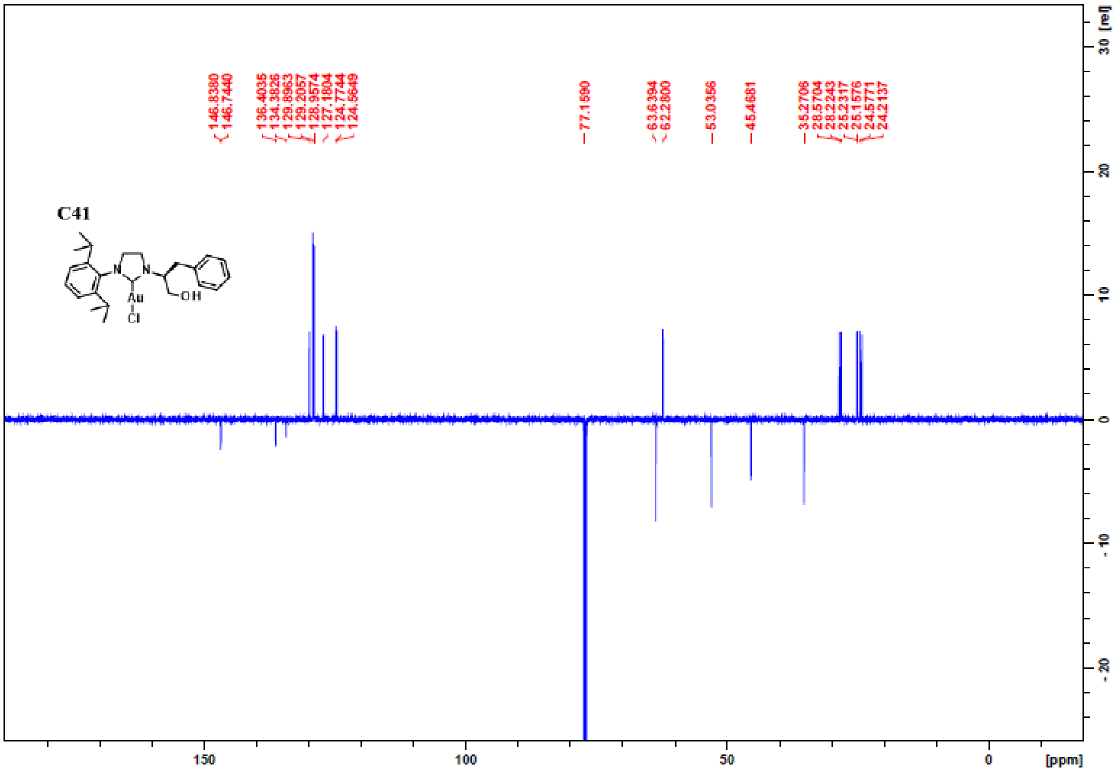

Figure S104. $^{13}C\{1H\}$ NMR (125 MHz) spectrum of **C41** in $CDCl_3$

(*4S*)-*4-tert*-Butyl-*3*-(*2',6'*-diisopropylphenyl)-*1*-[(*1S*)-*1*-benzyl-*2*-hydroxiethyl]imidazolidin-2-ylidene-gold-chloride (C42)

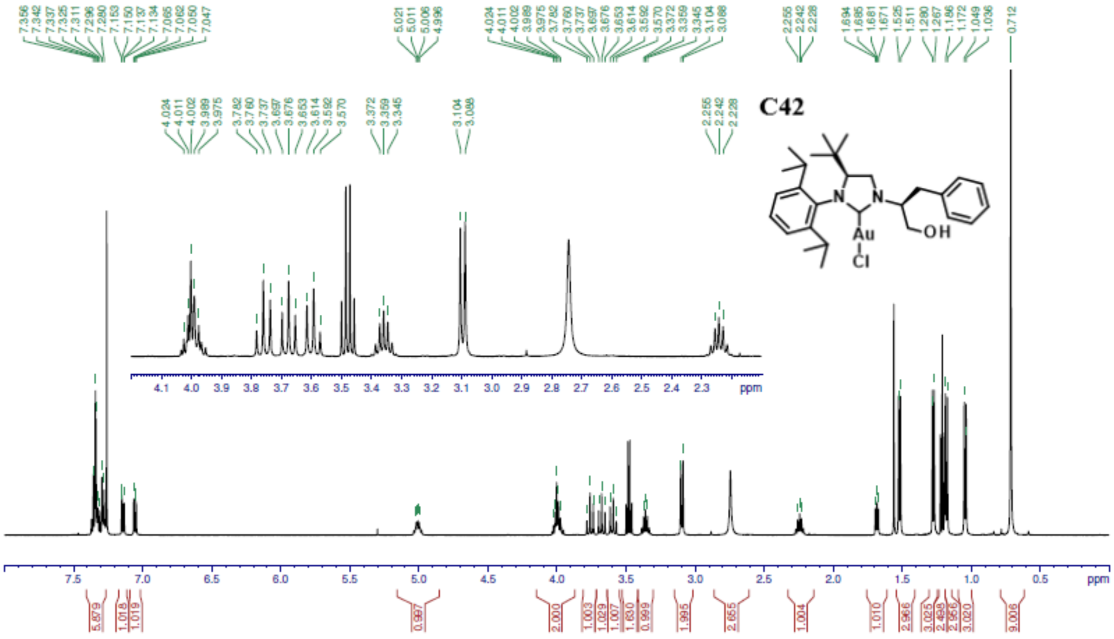

Figure S105. ^{1}H NMR (500 MHz) spectrum of **C42** in $CDCl_3$

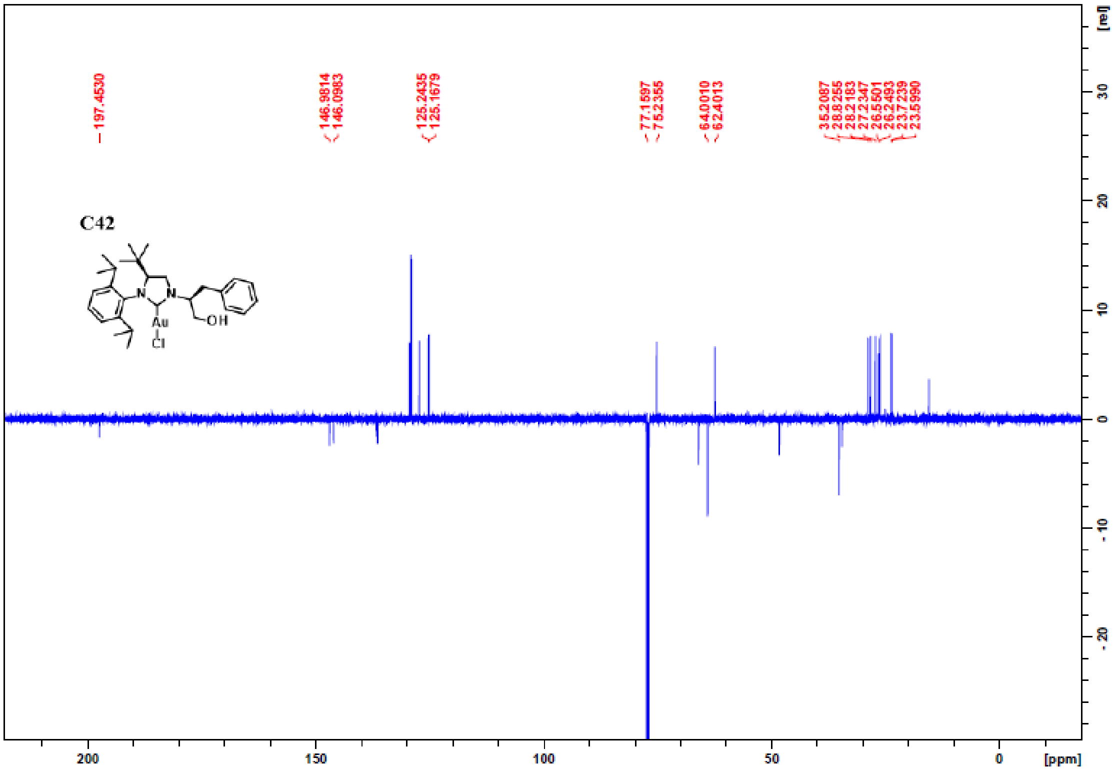

Figure S106. $^{13}C\{1H\}$ NMR (125 MHz) spectrum of **C42** in $CDCl_3$

***1,1*-dimethylprop-*2*-ynyl-*2,2*-dimethylpropanoate (1a)**

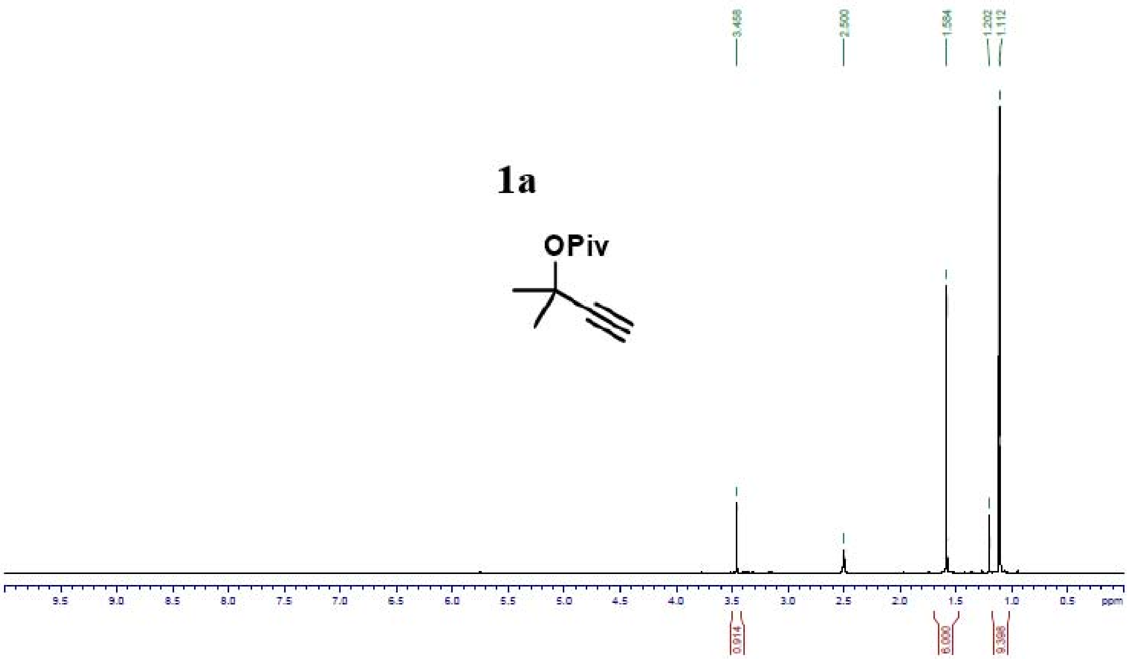

Figure S107. ^{1}H NMR (500 MHz) spectrum of **1a** in DMSO-d_6

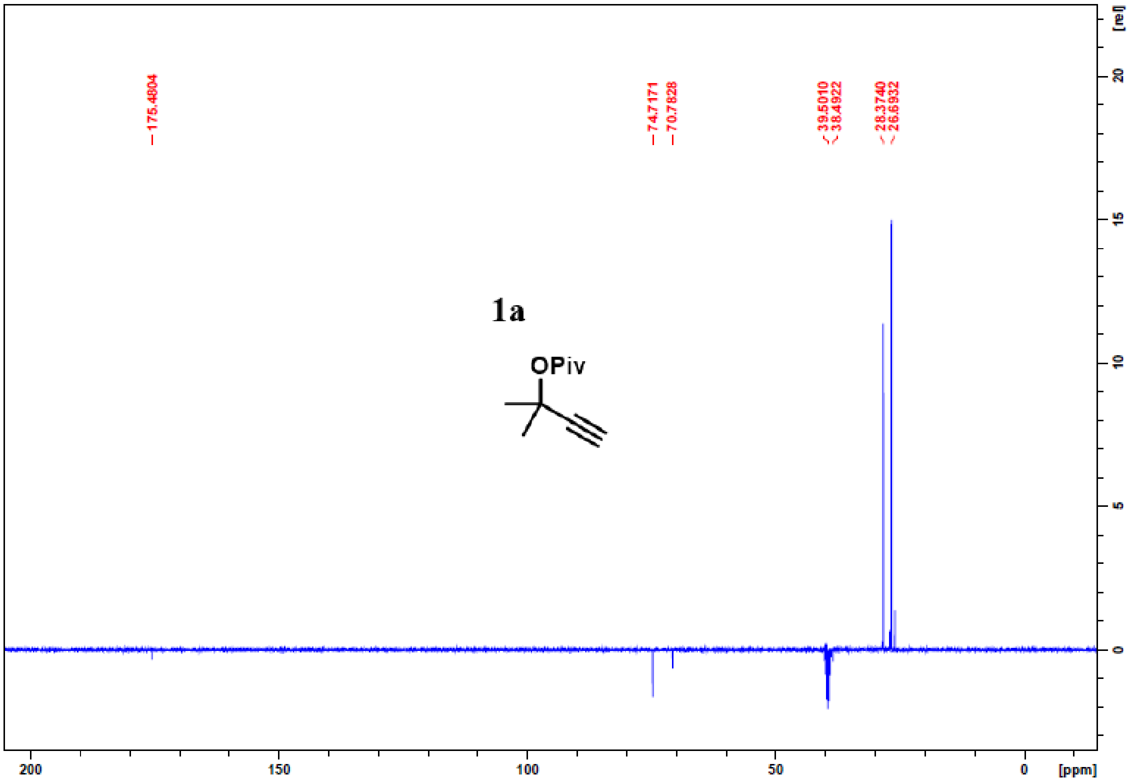

Figure S108. $^{13}C\{1H\}$ NMR (125 MHz) spectrum of **1a** in DMSO-d_6

(*1*-ethynylcyclohexyl)-*2*,*2*-dimethylpropanoate (1b)

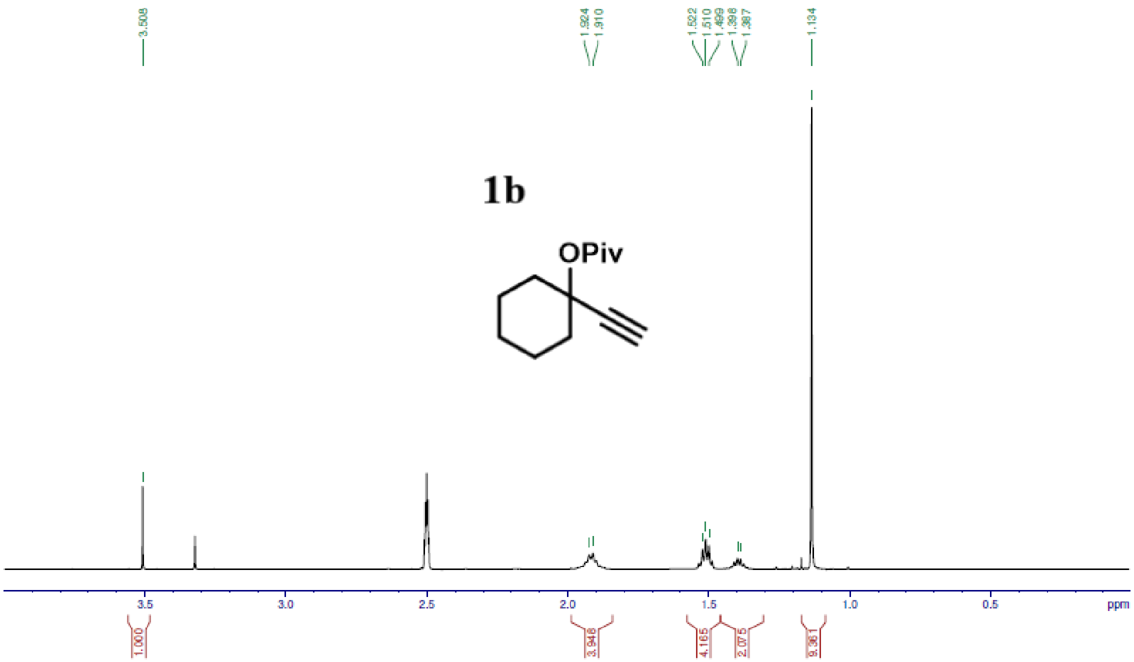

Figure S109. ^{1}H NMR (500 MHz) spectrum of **1b** in DMSO-d_6

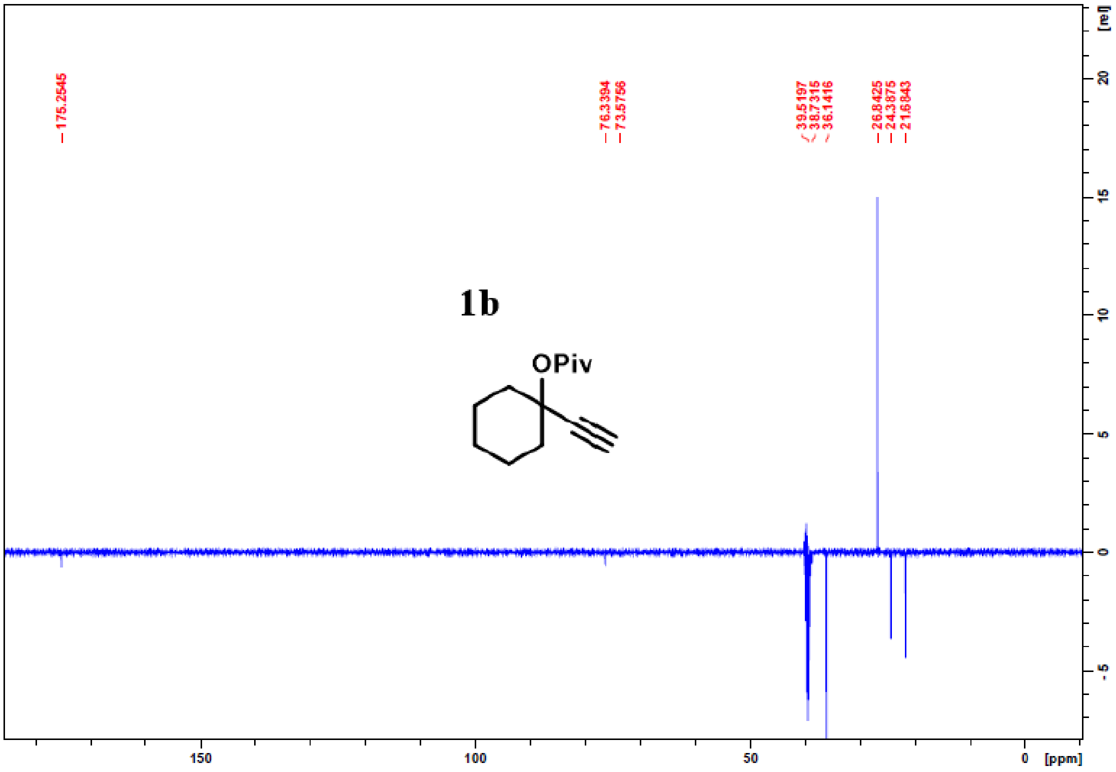

Figure S110. ^{13}C{1H} NMR (125 MHz) spectrum of **1b** in DMSO-d_6

***1*-phenylprop-*2*-ynyl-*2*,*2*-dimethylpropanoate (1c)**

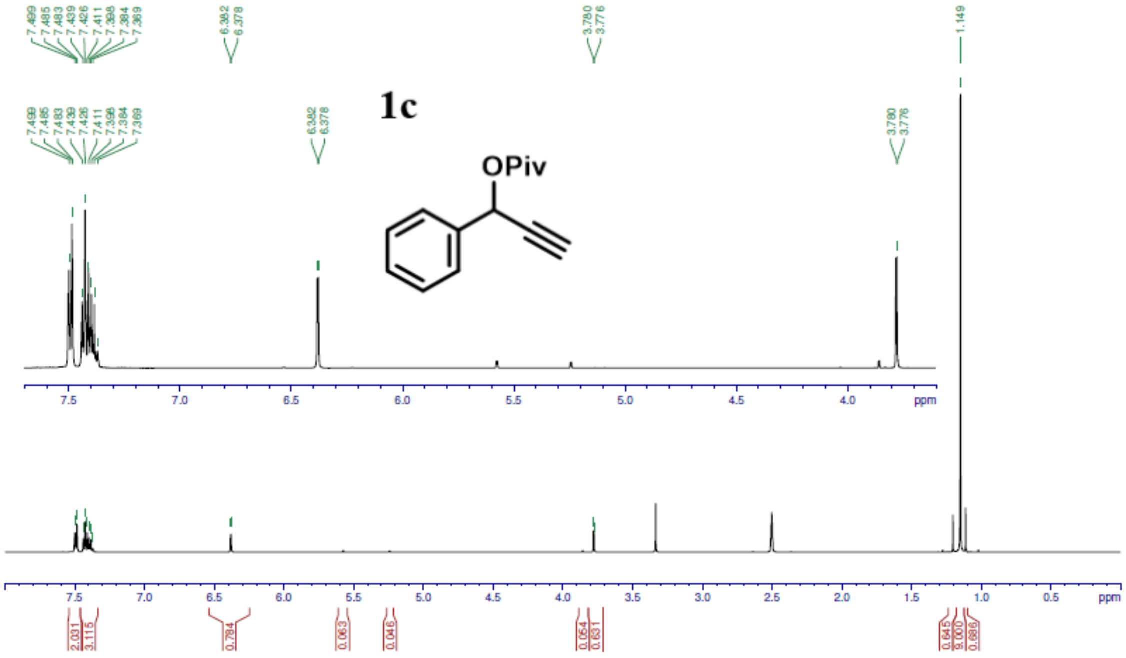

Figure S111. ^{1}H NMR (500 MHz) spectrum of **1c** in DMSO-d_6

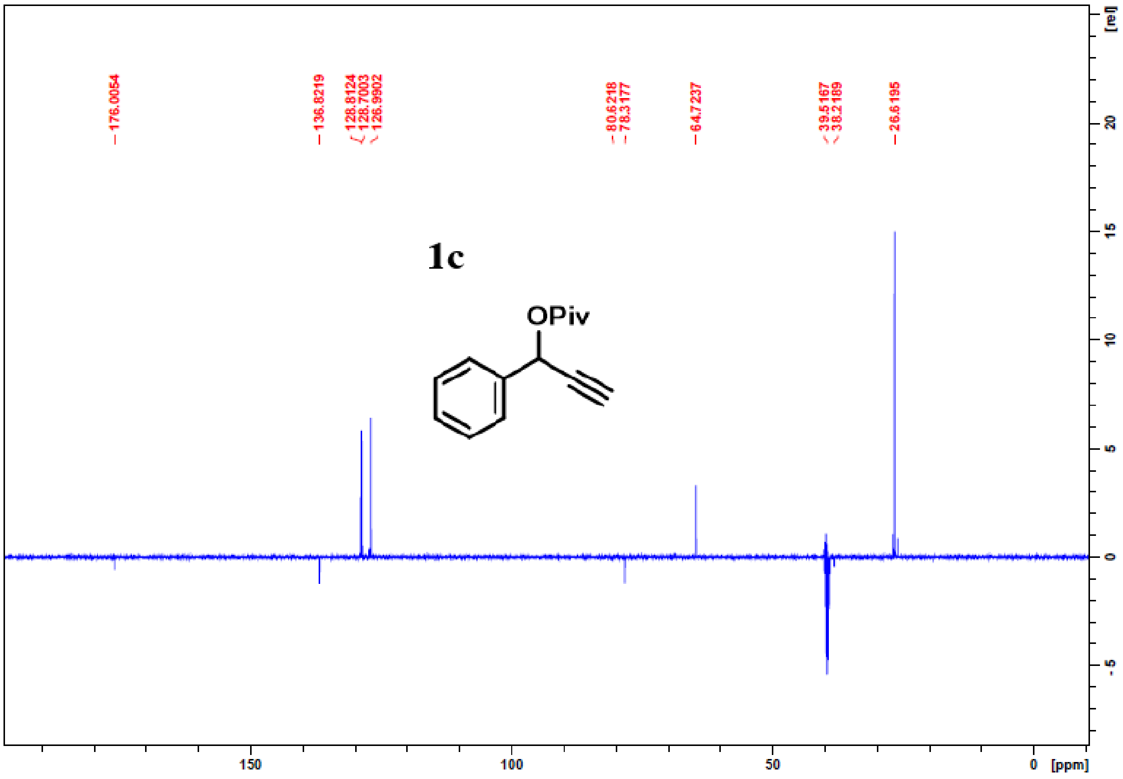

Figure S112. ^{13}C{1H} NMR (125 MHz) spectrum of **1c** in DMSO-d_6

***tert*-butyl 3-(1-acetoxyprop-2-ynyl)benzoate (1d)**

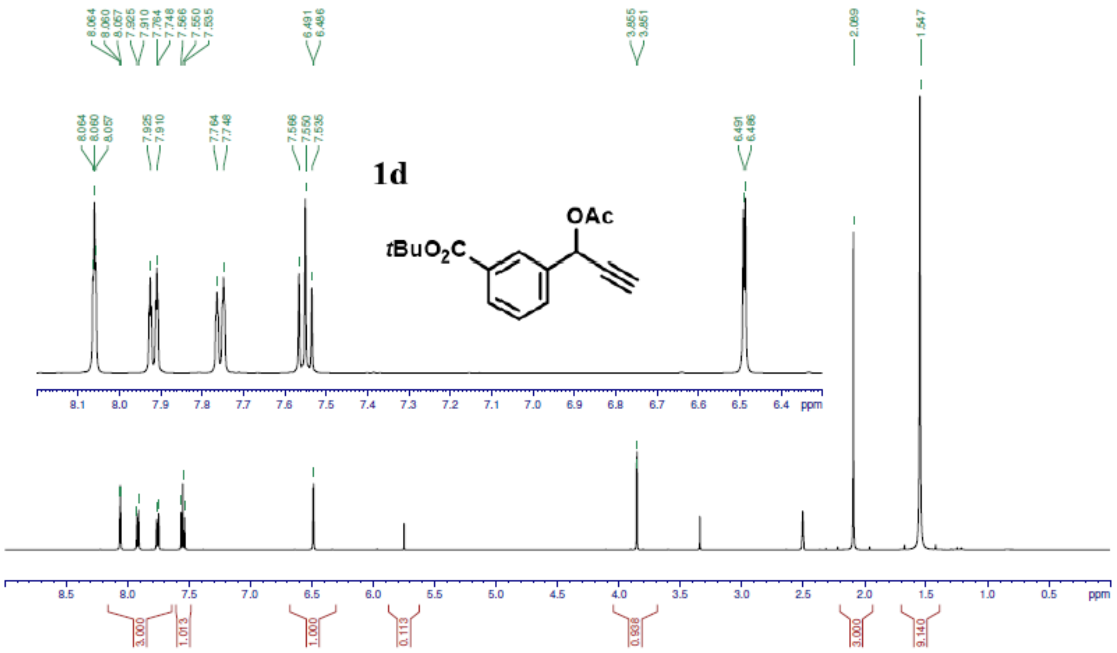

Figure S113. ^{1}H NMR (500 MHz) spectrum of **1d** in DMSO-d_6

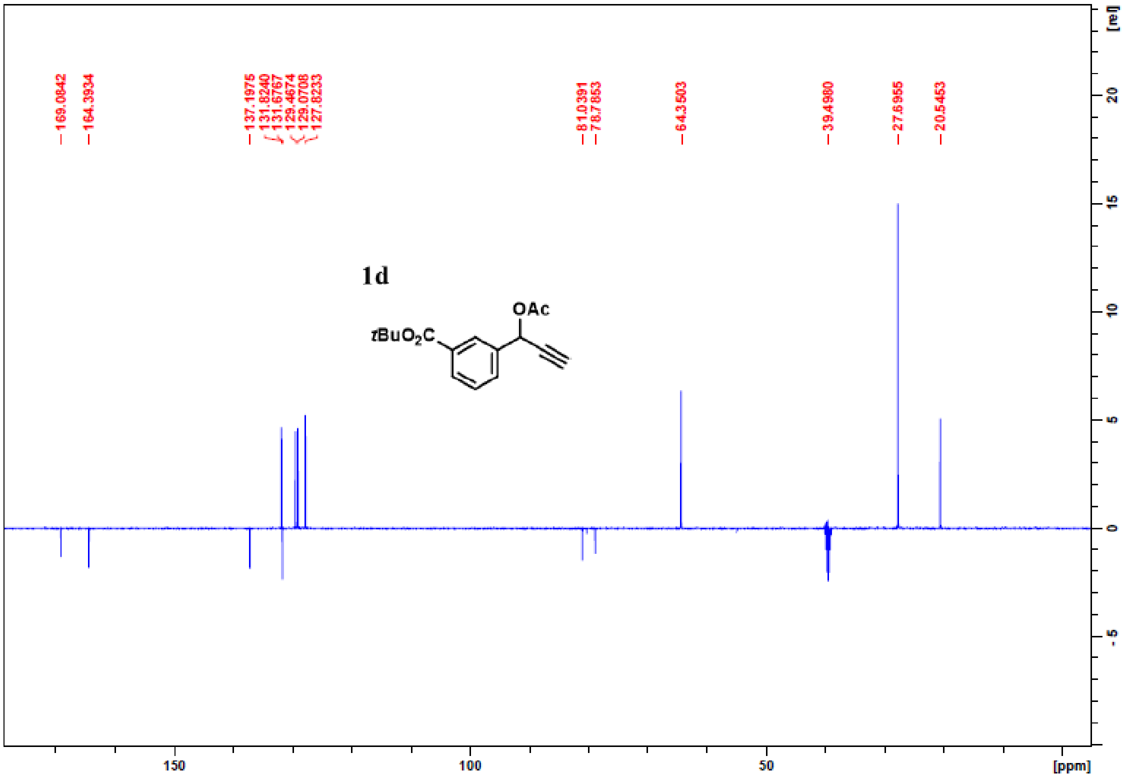

Figure S114. ^{13}C{1H} NMR (125 MHz) spectrum of **1d** in DMSO-d_6

1-(*cis-2*-acetoxy-*2*-methylcyclopropyl)-*2*-methylprop-*1*-enyl *2*,*2*-dimethylpropanoate (3a)

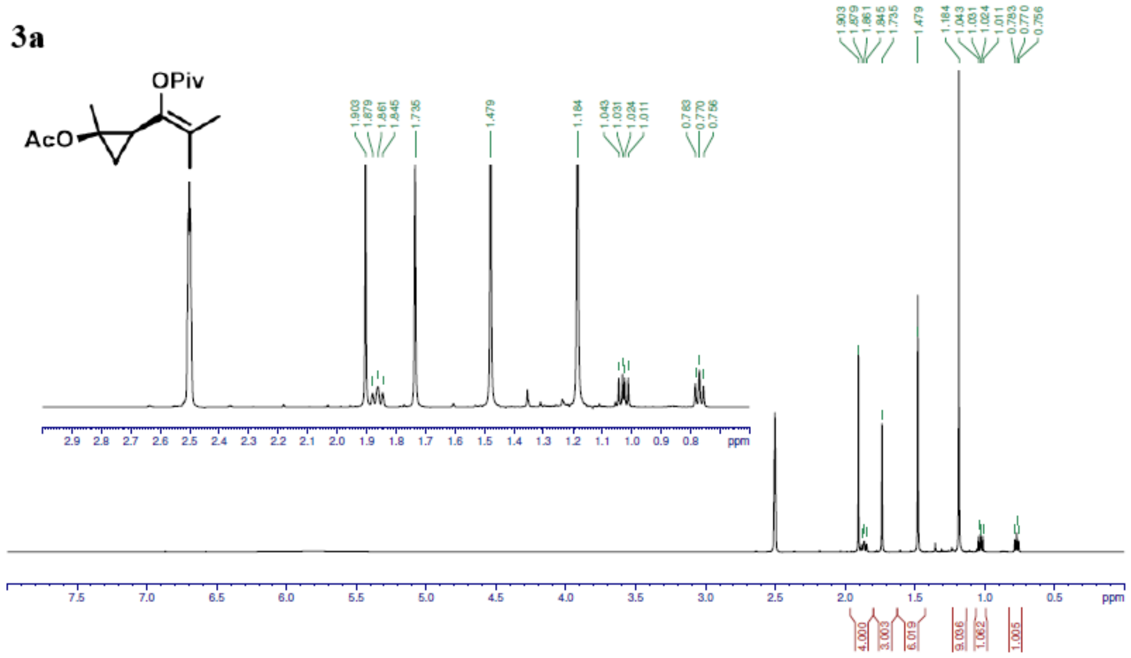

Figure S115. ^{1}H NMR (500 MHz) spectrum of **3a** in DMSO-d_6

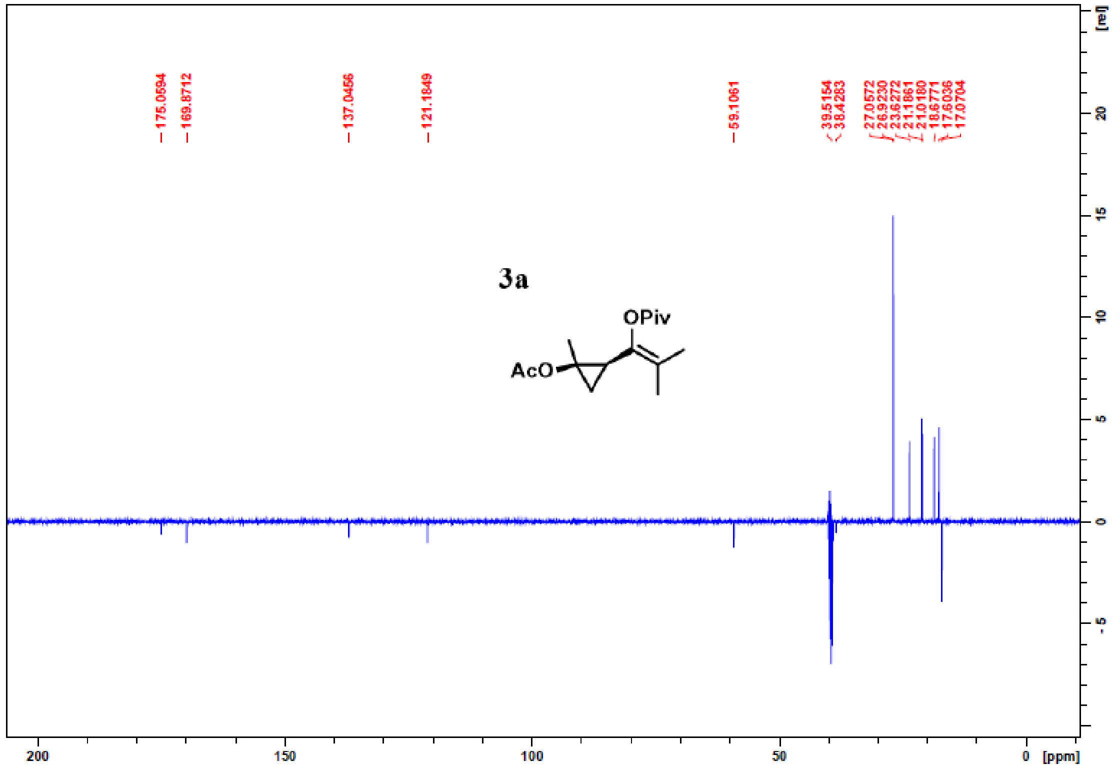

Figure S116. $^{13}C\{1H\}$ NMR (125 MHz) spectrum of **3a** in DMSO-d_6

1-(*trans*-2-acetoxy-2-methylcyclopropyl)-2-methylprop-1-enyl 2,2-dimethylpropanoate (4a)

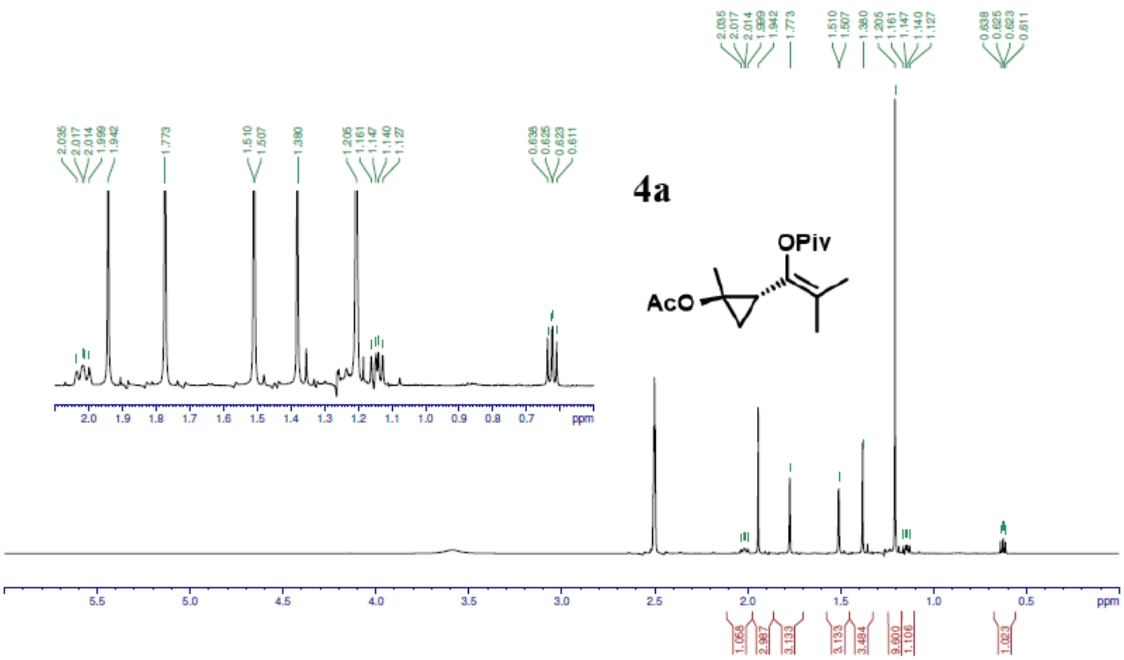

Figure S117. ^{1}H NMR (500 MHz) spectrum of **4a** in DMSO-d_6

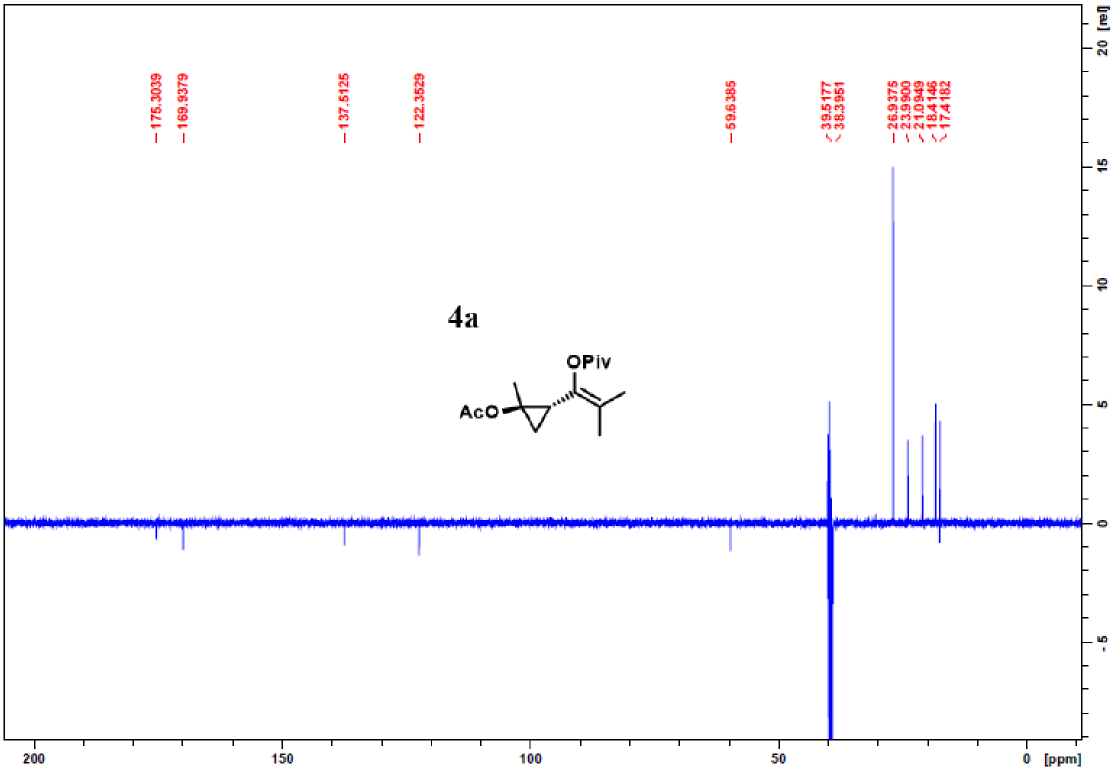

Figure S118. $^{13}C\{1H\}$ NMR (125 MHz) spectrum of **4a** in DMSO-d_6

***1*-(*cis*-*2*-acetoxy-*2*-methylcyclopropyl)-cyclohexylidenemethyl-*2*,*2*-dimethylpropanoate (3b)**

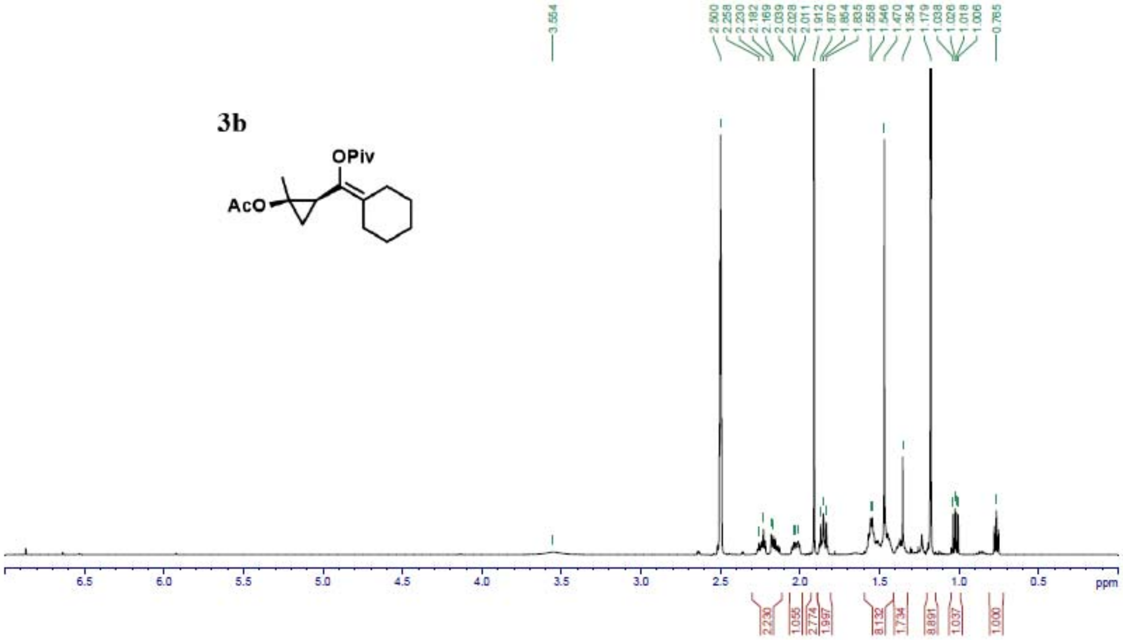

Figure S119. ^{1}H NMR (500 MHz) spectrum of **3b** in DMSO-d_6

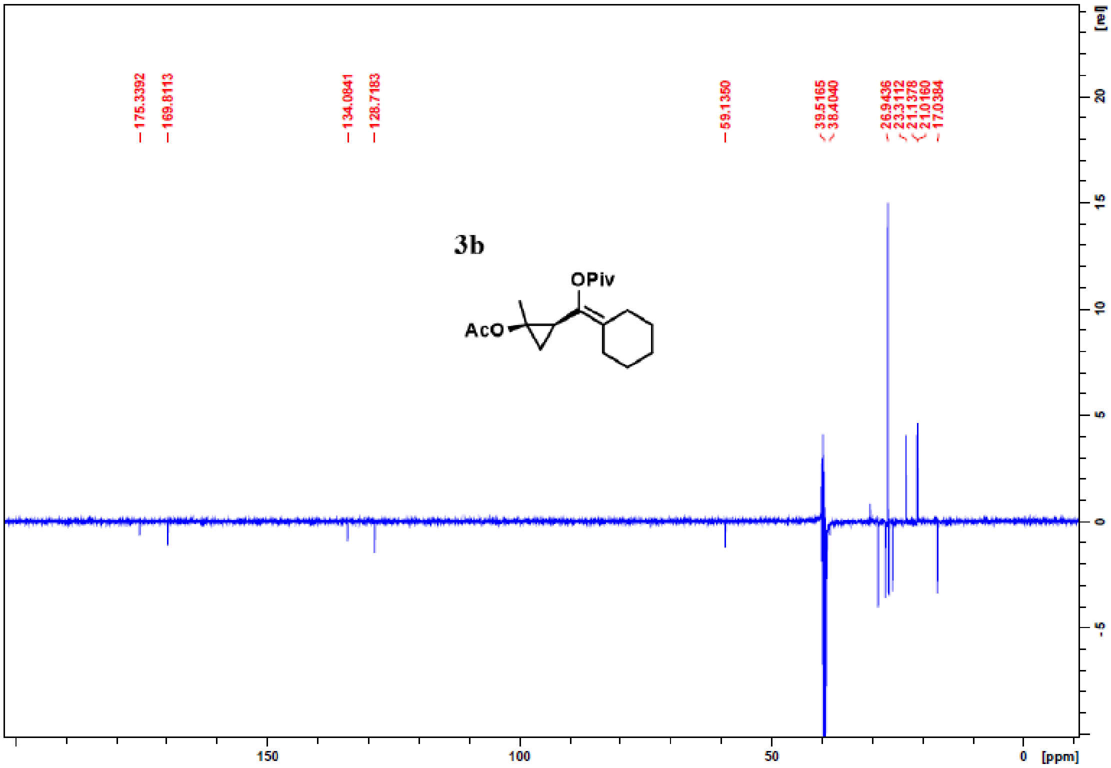

Figure S120. $^{13}C\{1H\}$ NMR (125 MHz) spectrum of **3b** in DMSO-d_6

***1*-(*trans*-*2*-acetoxy-*2*-methylcyclopropyl)-cyclohexylidenemethyl-*2*,*2*-dimethylpropanoate (4b)**

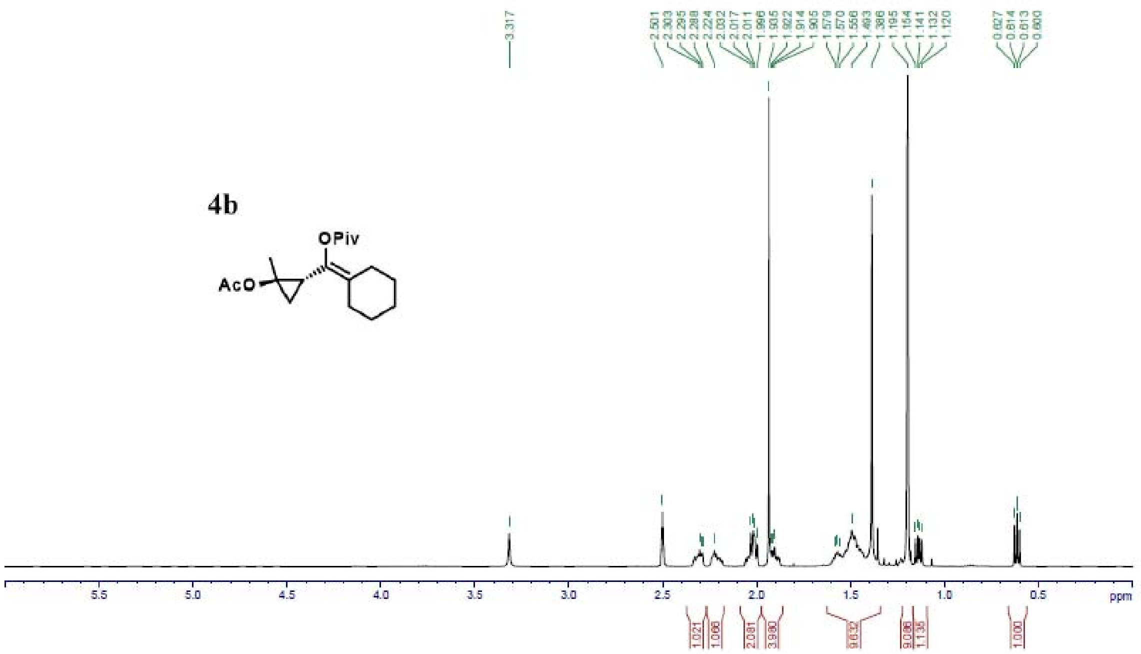

Figure S121. ^{1}H NMR (500 MHz) spectrum of **4b** in DMSO-d_6

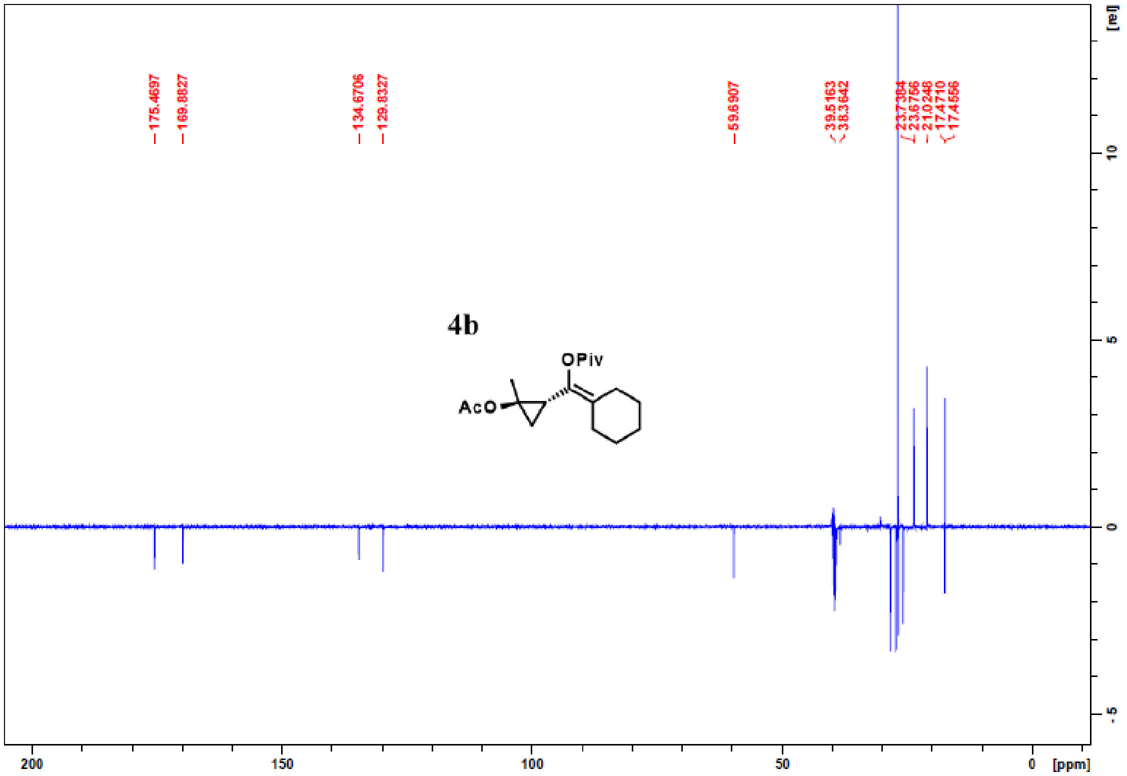

Figure S122. $^{13}C\{1H\}$ NMR (125 MHz) spectrum of **4b** in DMSO-d_6

***1*-[*cis*-*2*-acetoxy-*2*-methyl-cyclopropyl]-*2*-phenyl-vinyl-*2,2*-dimethylpropanoate (3c)**

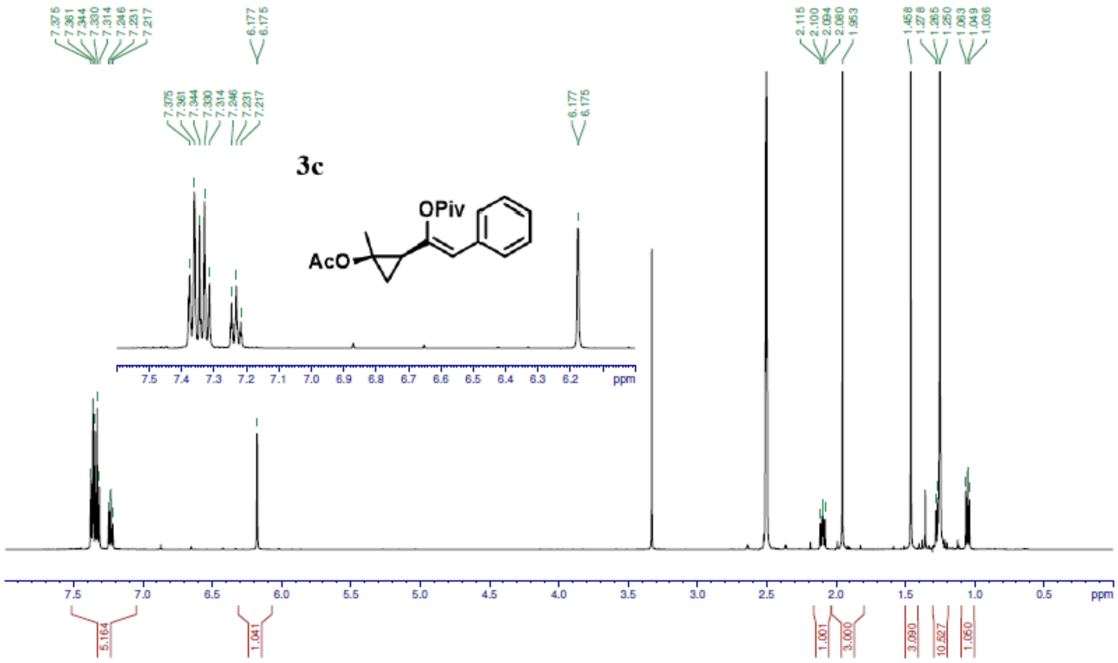

Figure S123. ^{1}H NMR (500 MHz) spectrum of **3c** in DMSO-d_6

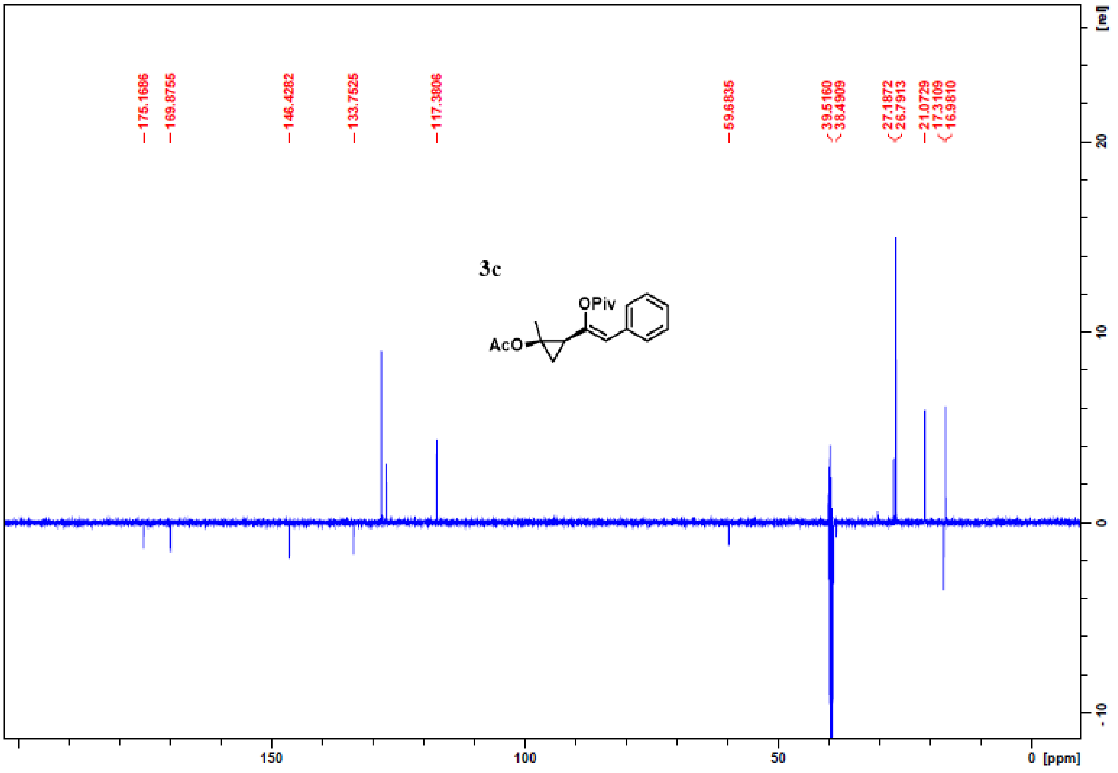

Figure S124. ^{13}C{1H} NMR (125 MHz) spectrum of **3c** in DMSO-d_6

***1*-[*cis*-*2*-acetoxy-*2*-methyl-cyclopropyl]-*2*-phenyl-vinyl-*2,2*-dimethylpropanoate (4c)**

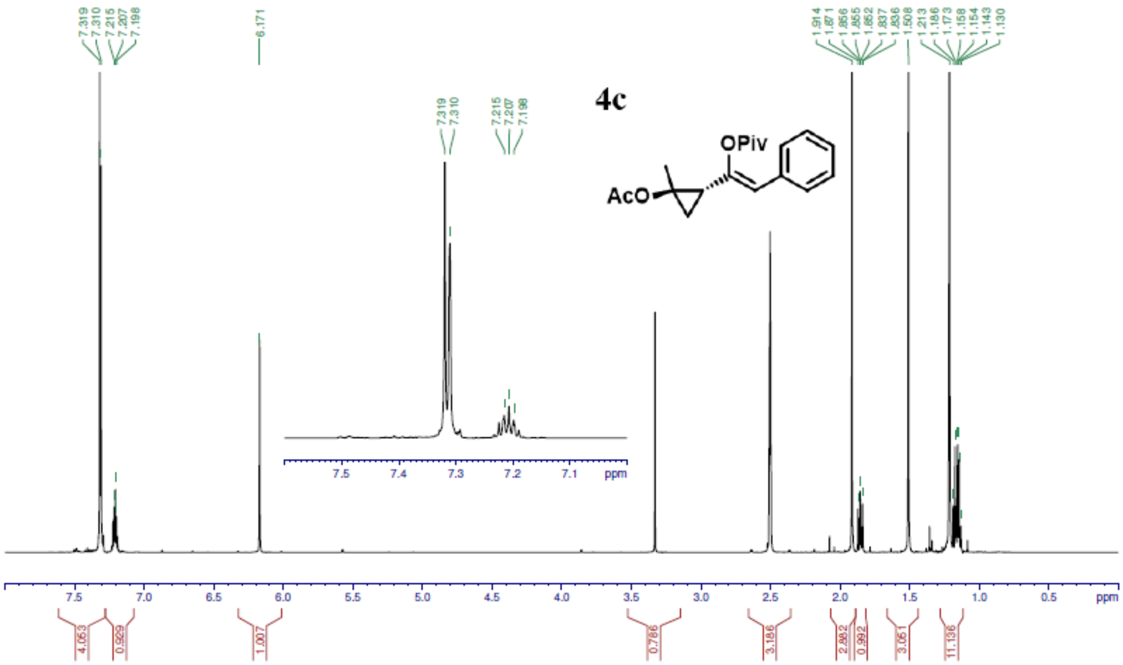

Figure S125. ^{1}H NMR (500 MHz) spectrum of **4c** in DMSO-d_6

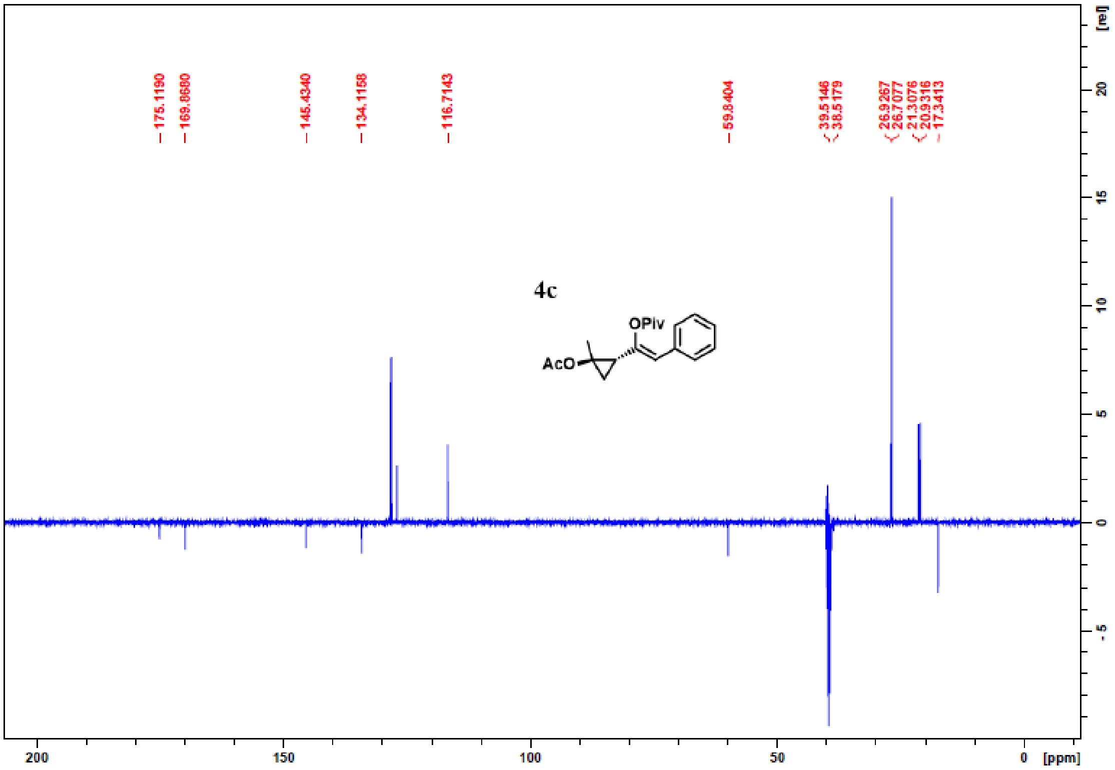

Figure S126. ^{13}C{1H} NMR (125 MHz) spectrum of **4c** in DMSO-d_6

***1*-[(*cis*-*2*-(*3*-methoxy-*3*-oxopropyl)cyclopropyl]-*2*-methylprop-*1*-enyl]-*2*,*2*-dimethylpropanoate (3d)**

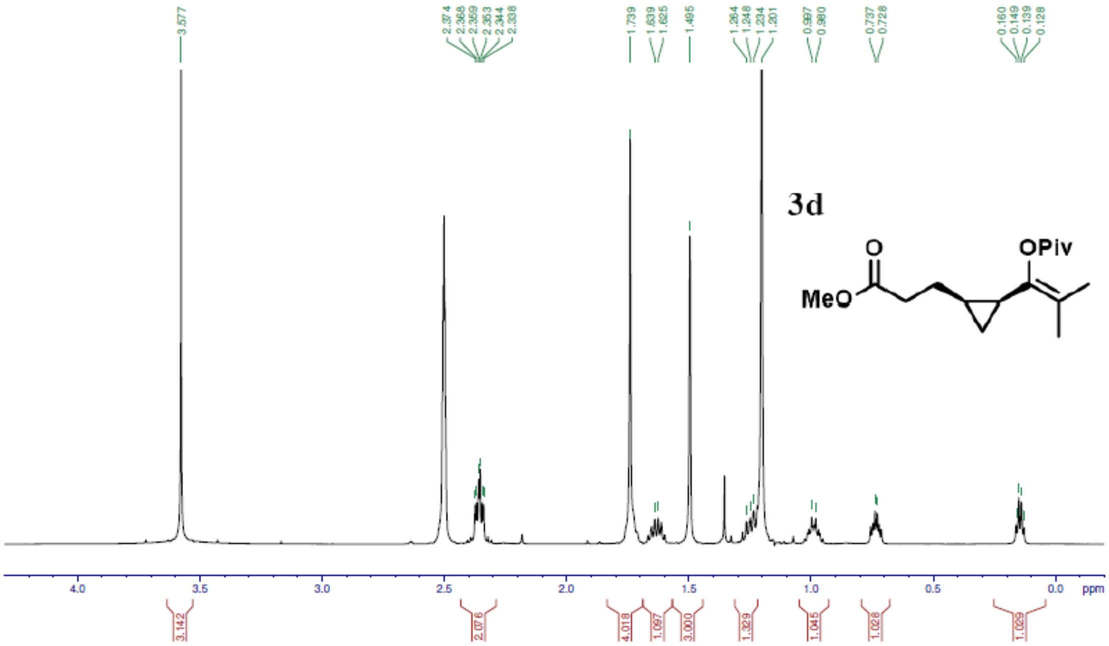

Figure S127. ^{1}H NMR (500 MHz) spectrum of **3d** in DMSO-d_6

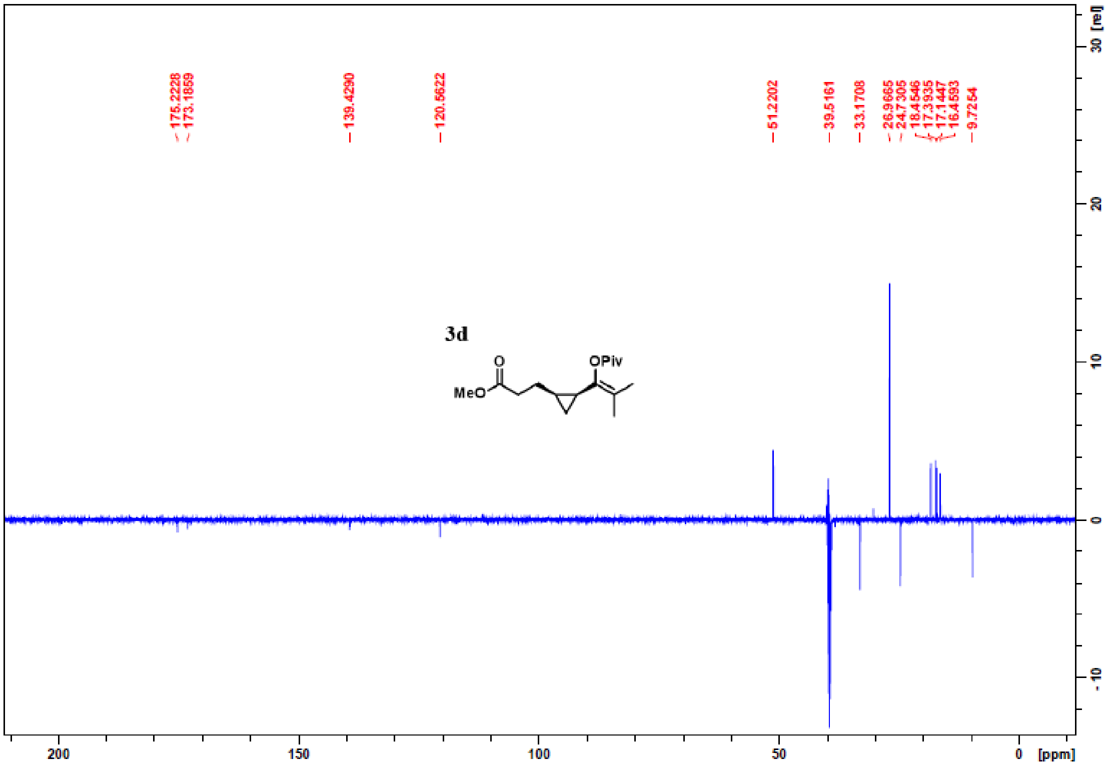

Figure S128. ^{13}C{1H} NMR (125 MHz) spectrum of **3d** in DMSO-d_6

***1*-[(*trans*-*2*-(*3*-methoxy-*3*-oxopropyl)cyclopropyl]-*2*-methylprop-*1*-enyl-*2*,*2*-dimethylpropanoate (4d)**

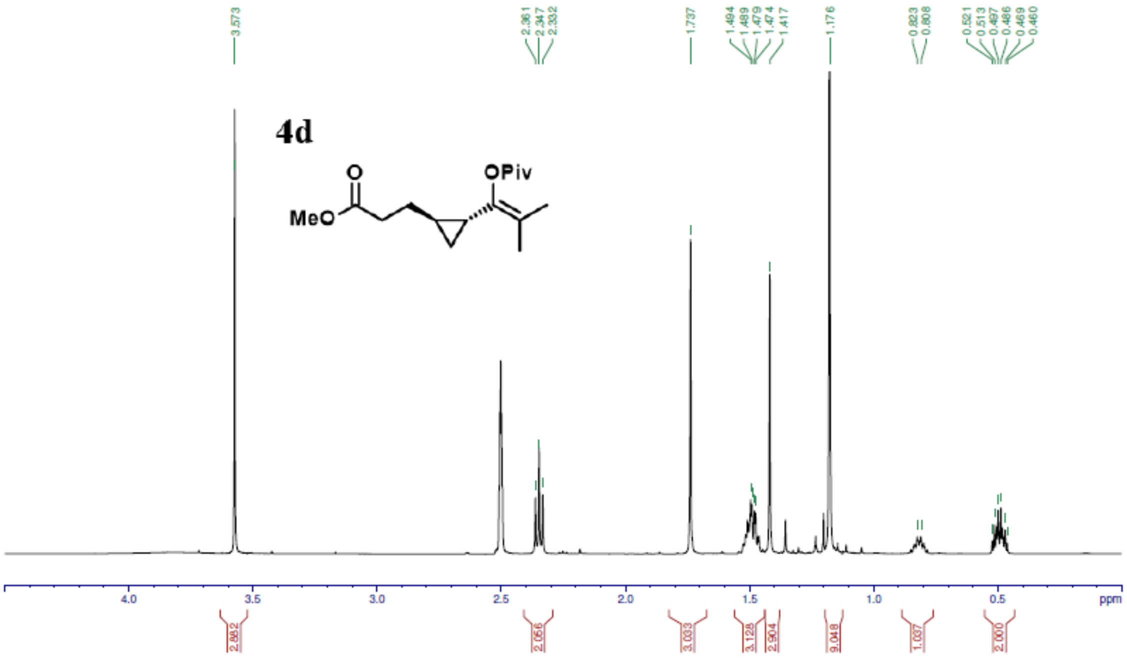

Figure S129. ^{1}H NMR (500 MHz) spectrum of **4d** in DMSO-d_6

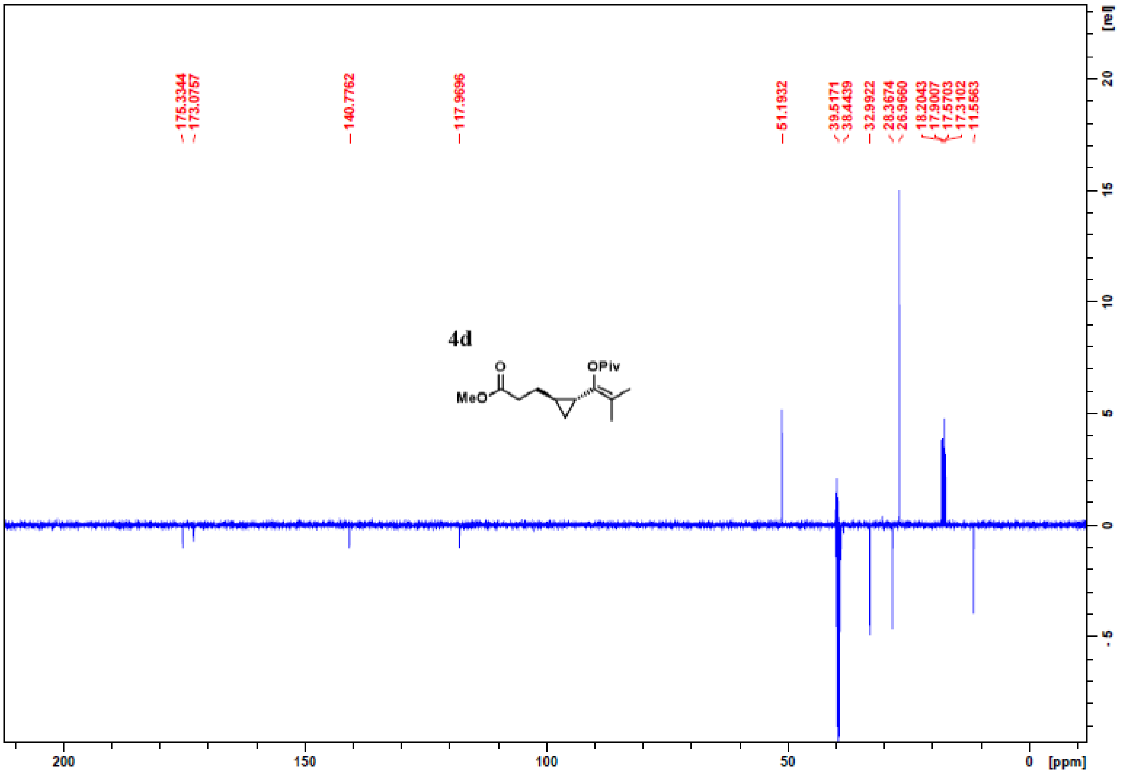

Figure S130. ^{13}C{1H} NMR (125 MHz) spectrum of **4d** in DMSO-d_6

***1*-[(*cis*-*2*-[*2*-(*1*,*3*-dioxoisoindolin-*2*-yl)ethyl]cyclopropyl]-*2*-methylprop-*1*-enyl-*2*,*2*-dimethylpropanoate (3e)**

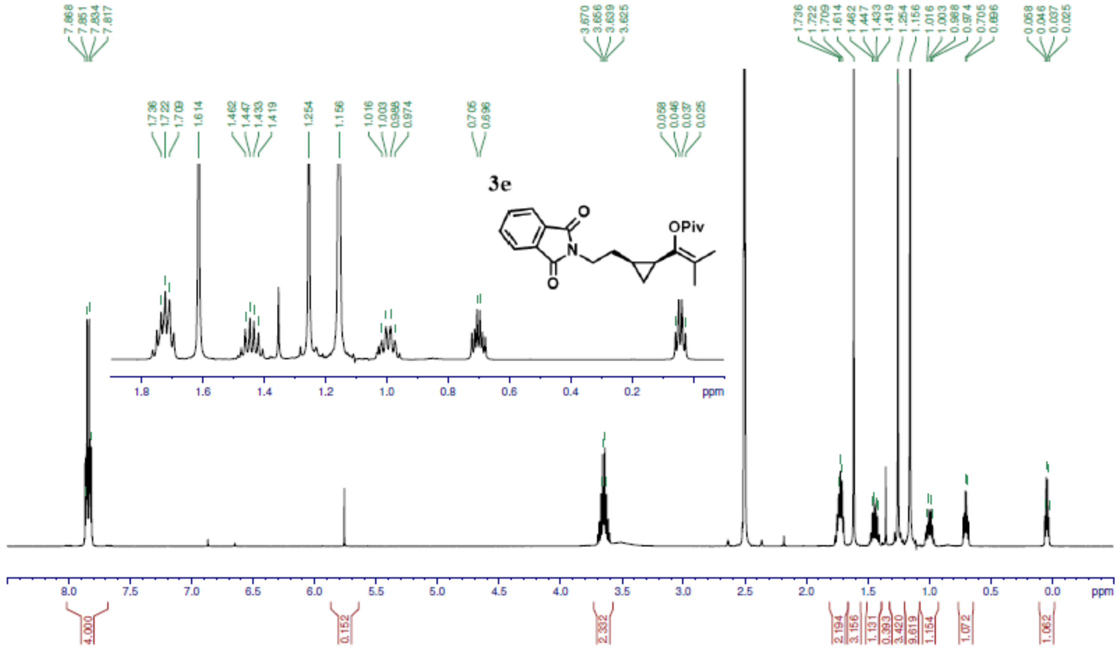

Figure S131. ^{1}H NMR (500 MHz) spectrum of **3e** in DMSO-d_6

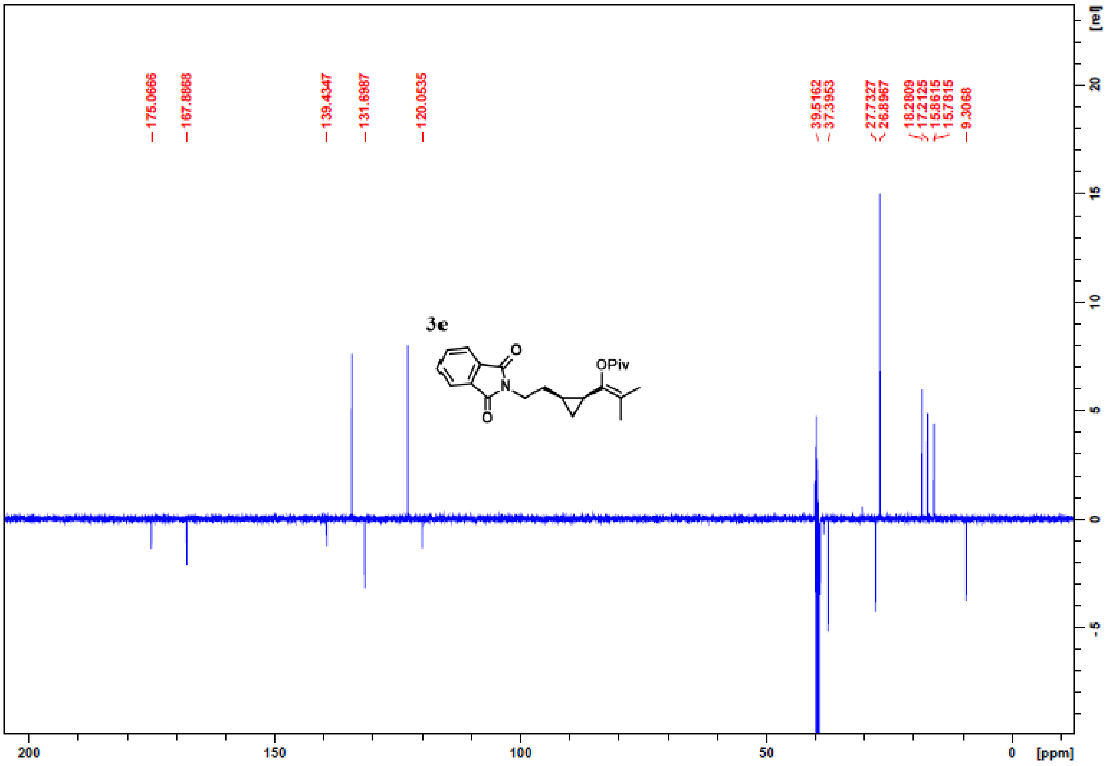

Figure S132. ^{13}C{1H} NMR (125 MHz) spectrum of **3e** in DMSO-d_6

***1*-[*trans*-*2*-[*2*-(*1*,*3*-dioxoisoindolin-*2*-yl)ethyl]cyclopropyl]-*2*-methylprop-*1*-enyl-*2*,*2*-dimethylpropanoate (4e)**

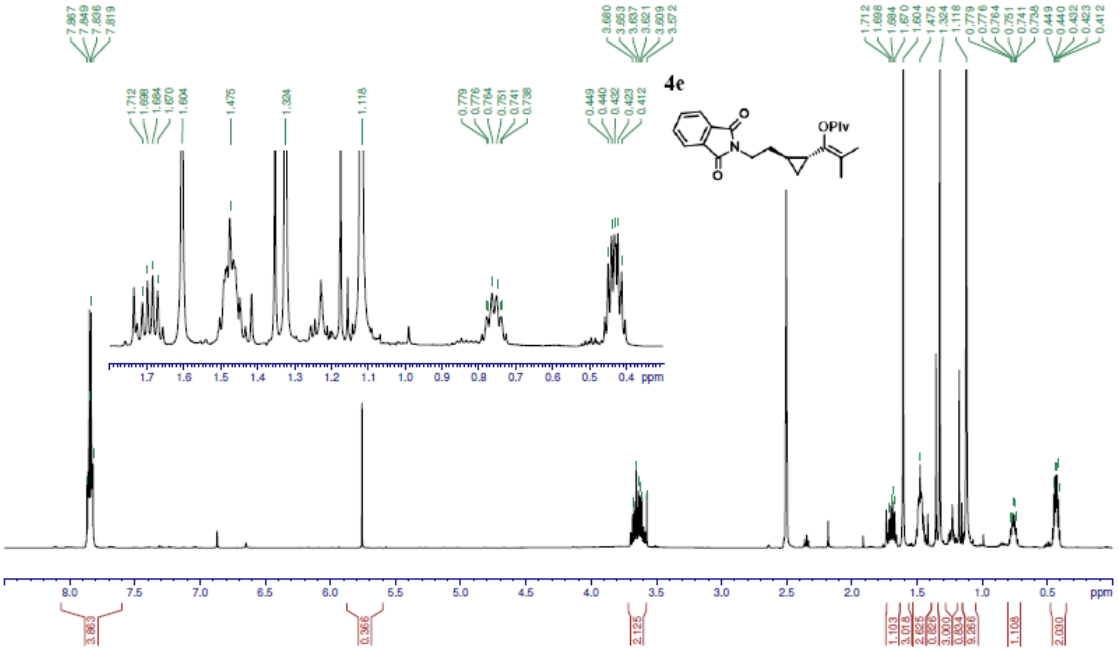

Figure S133. ^{1}H NMR (500 MHz) spectrum of **4e** in DMSO-d_6

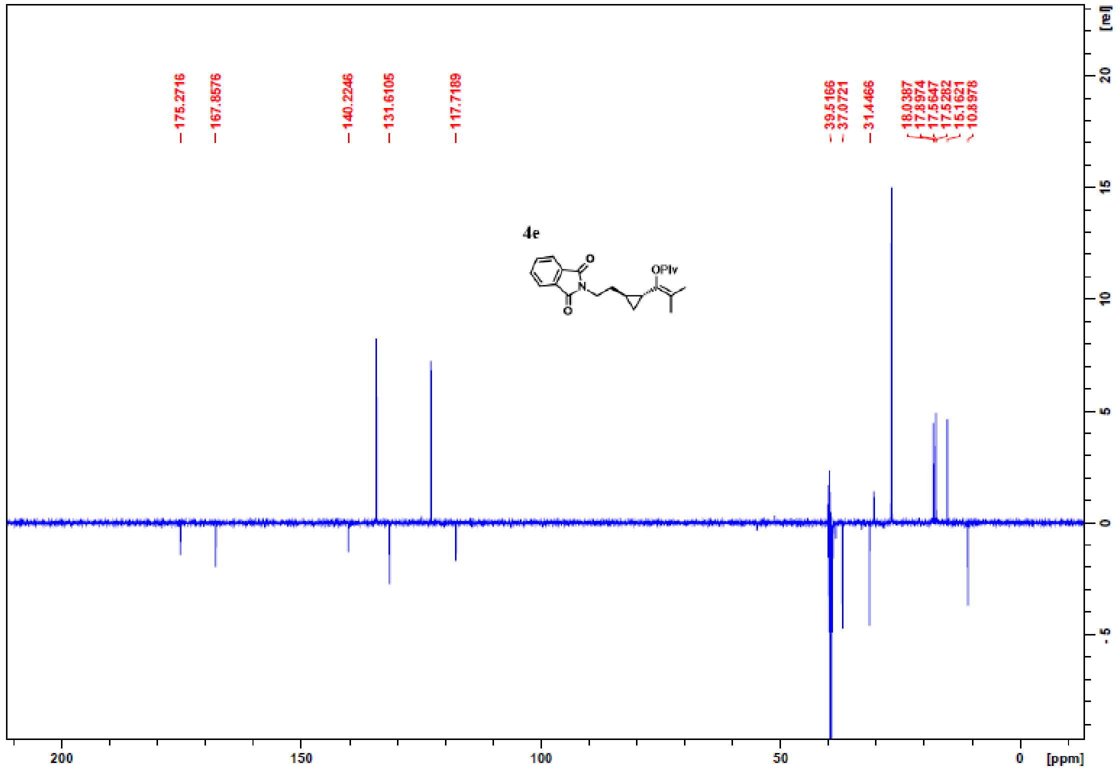

Figure S134. $^{13}C\{1H\}$ NMR (125 MHz) spectrum of **4e** in DMSO-d_6

***1*-[(*cis*-*2*-(*3*-thienyl)cyclopropyl]-*2*-methylprop-*1*-enyl-*2*,*2*-dimethylpropanoate (3f)**

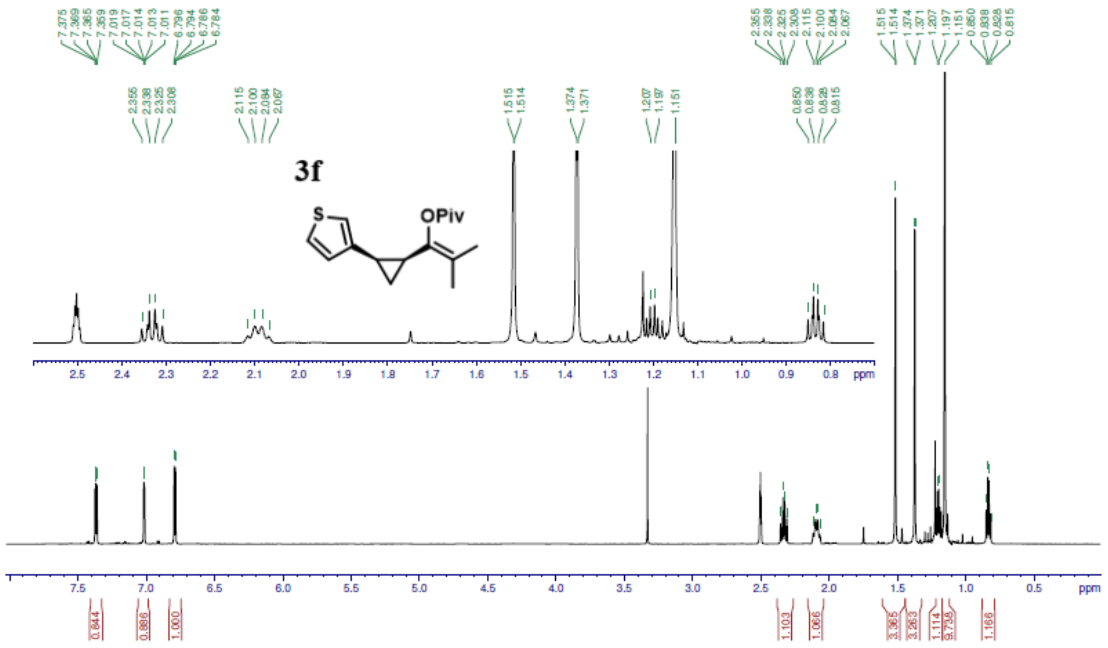

Figure S135. ^{1}H NMR (500 MHz) spectrum of **3f** in DMSO-d_6

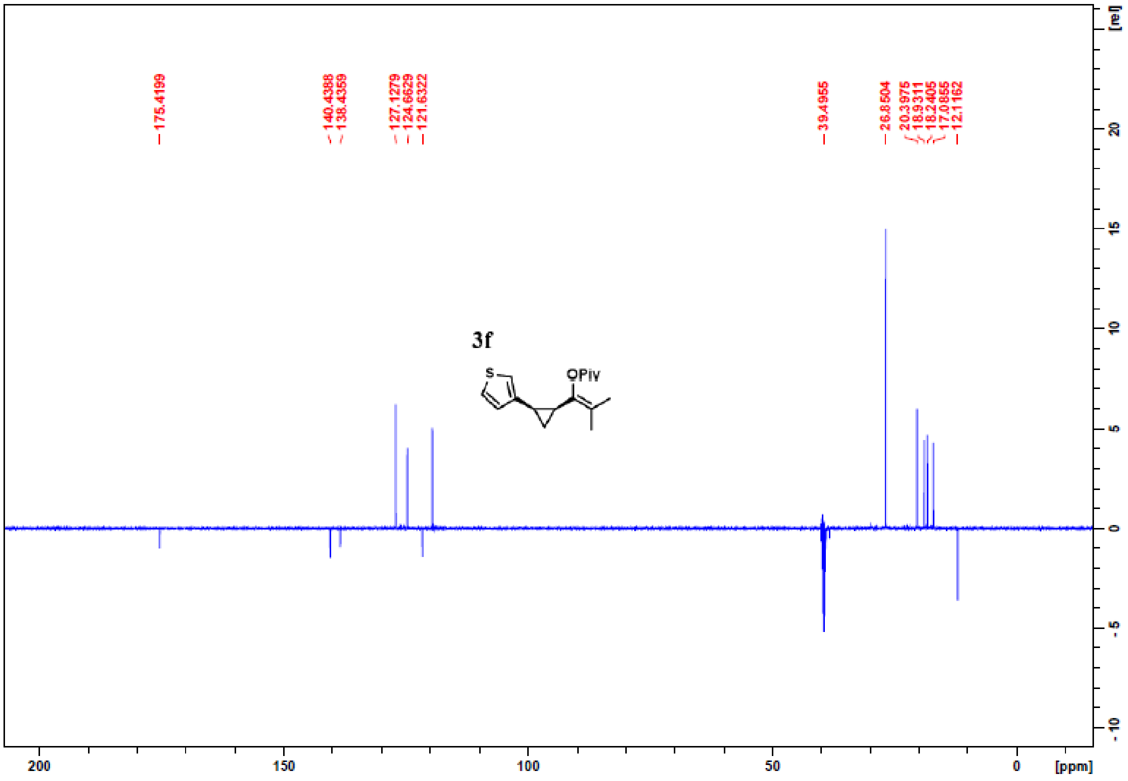

Figure S136. ^{13}C{1H} NMR (125 MHz) spectrum of **3f** in DMSO-d_6

***1*-[(*cis*-*2*-(*4*-chlorophenyl)cyclopropyl]-*2*-methylprop-*1*-enyl-*2*,*2*-dimethylpropanoate (3g)**

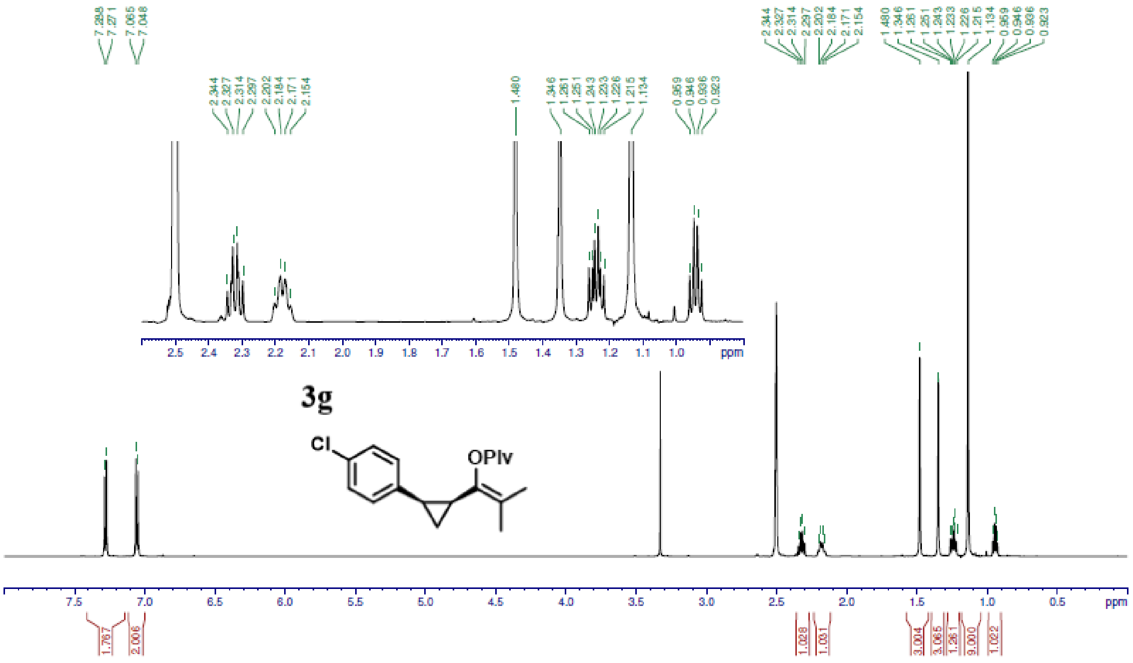

Figure S137. ^{1}H NMR (500 MHz) spectrum of **3g** in DMSO-d_6

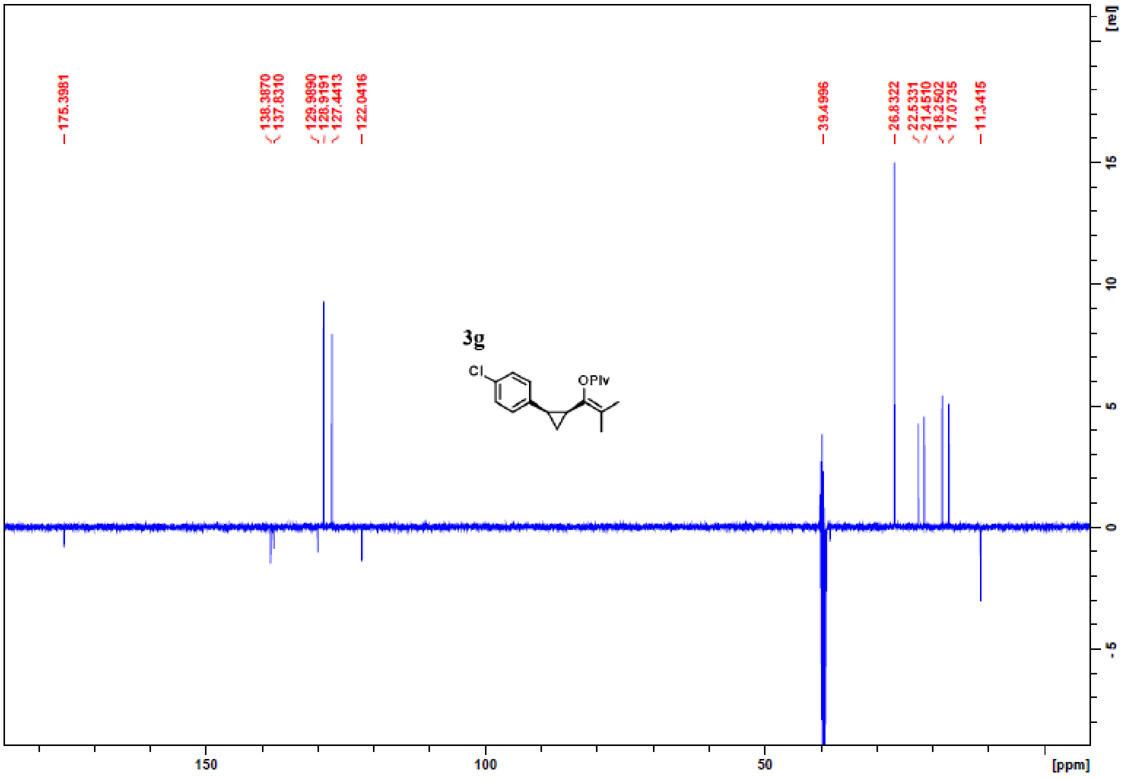

Figure S138. ^{13}C{1H} NMR (125 MHz) spectrum of **3g** in DMSO-d_6

***1*-[(*trans*-*2*-(*4*-chlorophenyl)cyclopropyl]-*2*-methylprop-*1*-enyl-*2*,*2*-dimethylpropanoate (4g)**

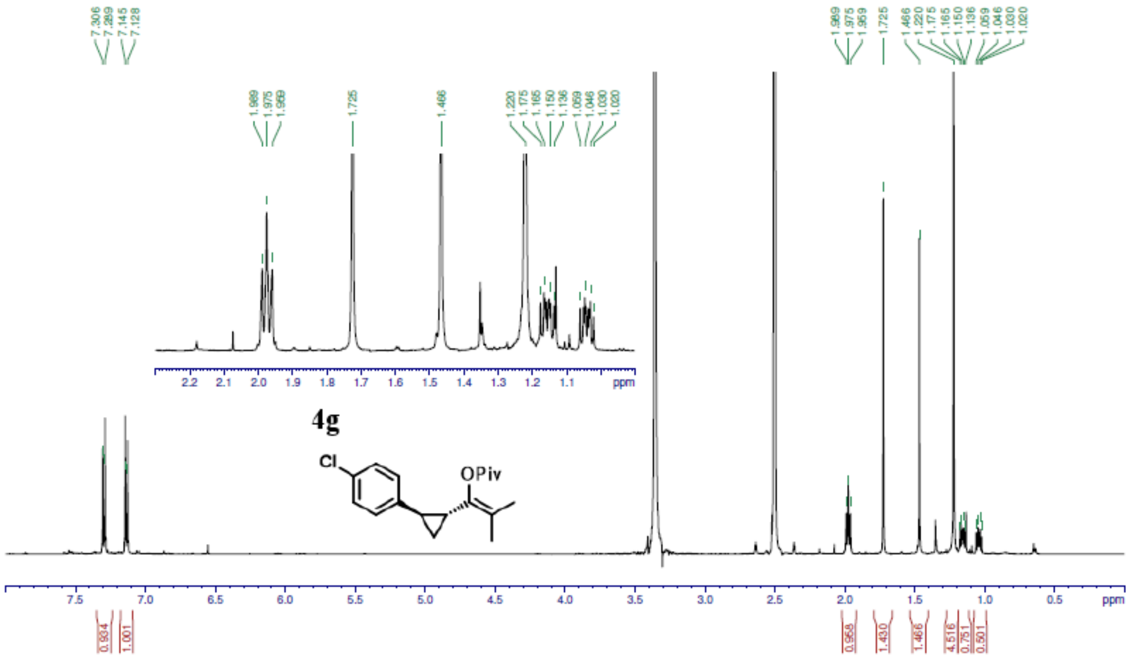

Figure S139. ^{1}H NMR (500 MHz) spectrum of **4g** in DMSO-d_6

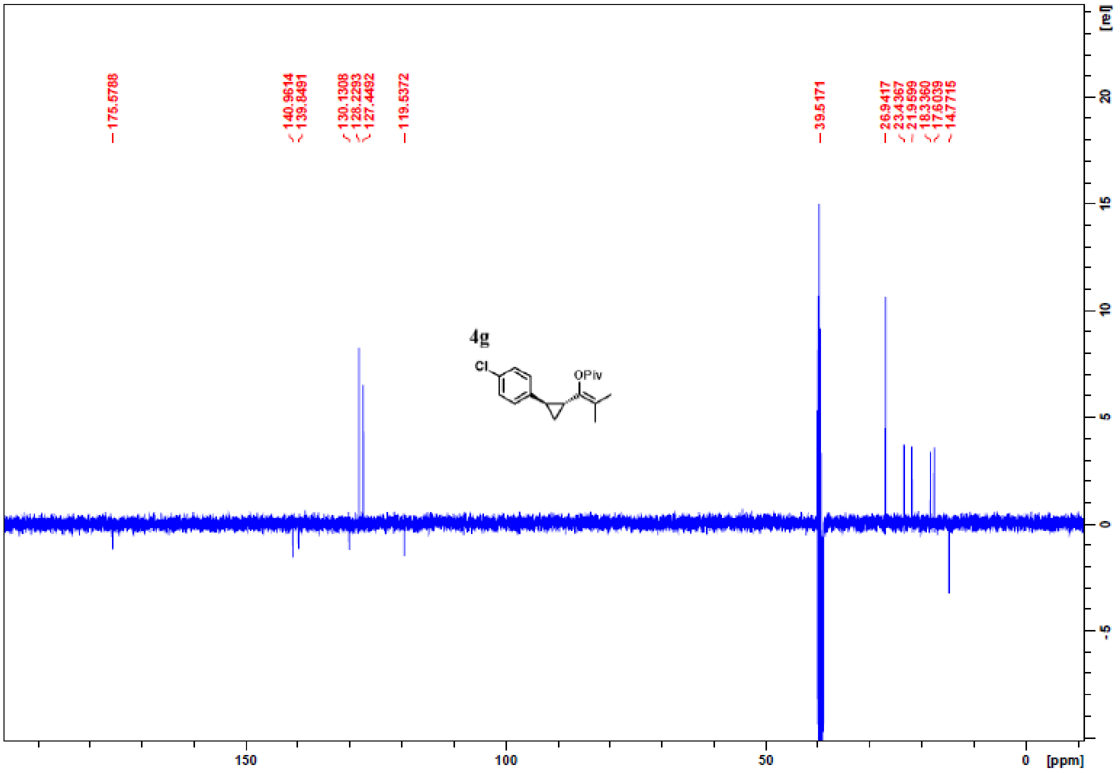

Figure S140. ^{13}C{1H} NMR (125 MHz) spectrum of **4g** in DMSO-d_6

***1*-[*cis*-*2*-(*3*-bromophenyl)cyclopropyl]-*2*-methylprop-*1*-enyl-*2*,*2*-dimethylpropanoate (3h)**

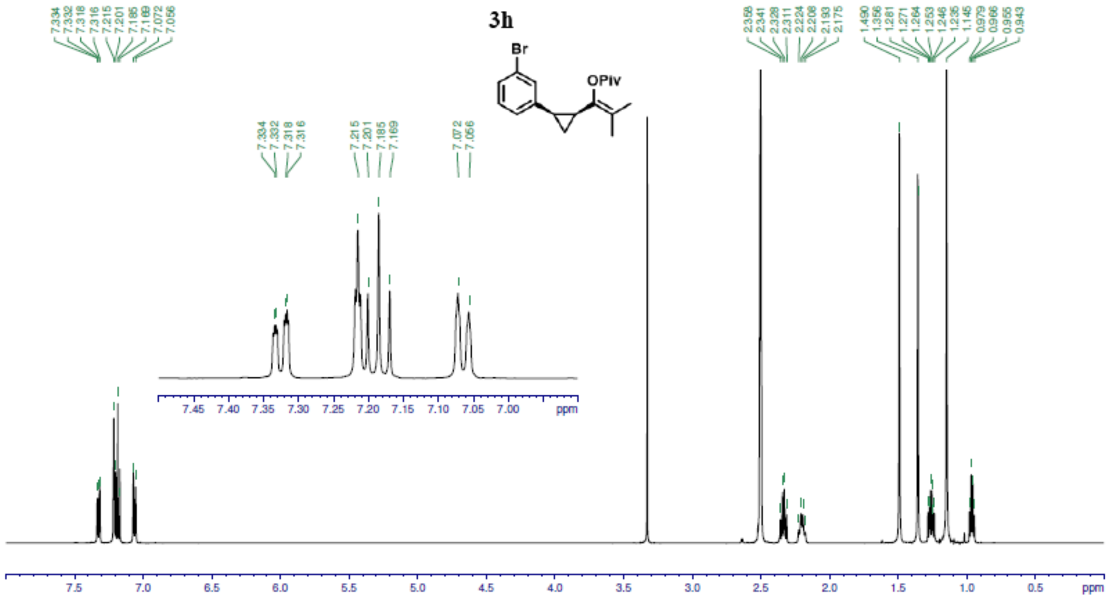

Figure S141. ^{1}H NMR (500 MHz) spectrum of **3h** in DMSO-d_6

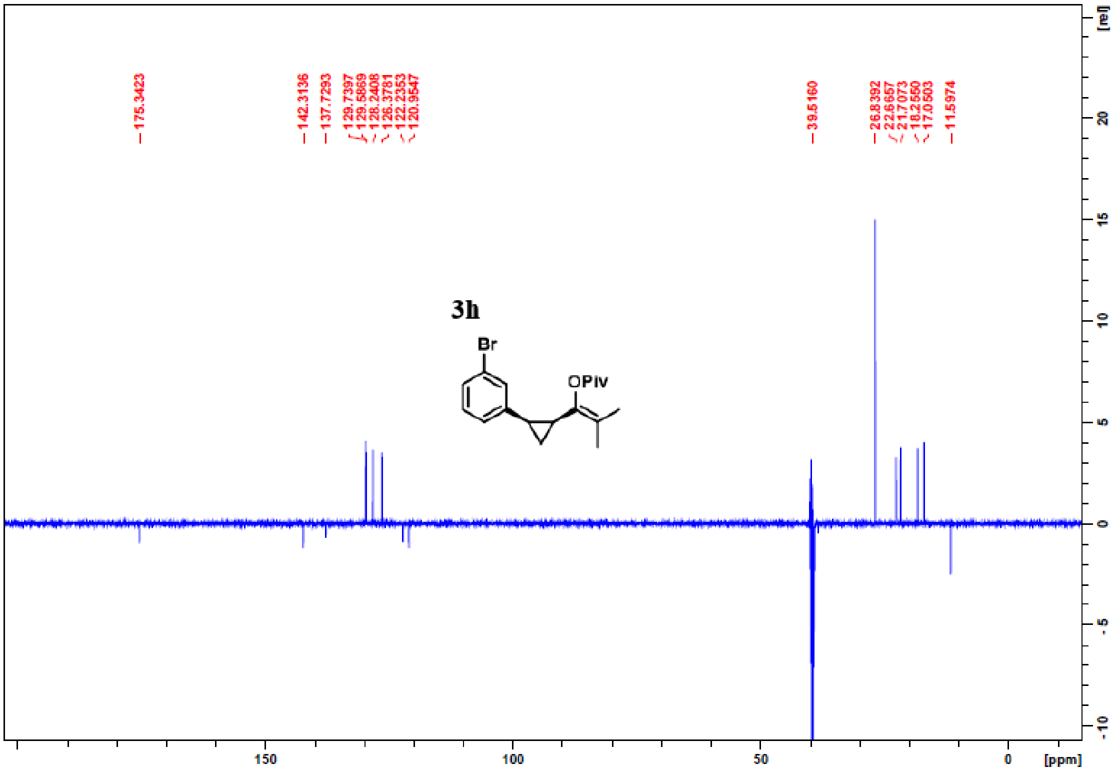

Figure S142. ^{13}C{1H} NMR (125 MHz) spectrum of **3h** in DMSO-d_6

***1*-[*trans*-*2*-(*3*-bromophenyl)cyclopropyl]-*2*-methylprop-*1*-enyl-*2*,*2*-dimethylpropanoate (4h)**

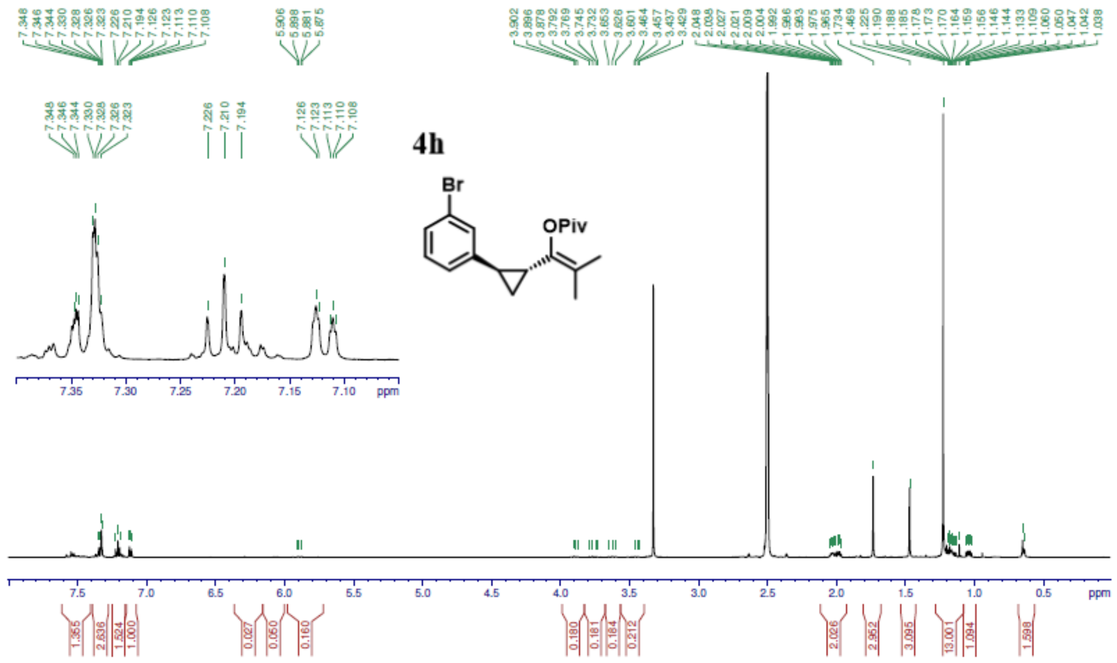

Figure S143. ^{1}H NMR (500 MHz) spectrum of **4h** in DMSO-d_6

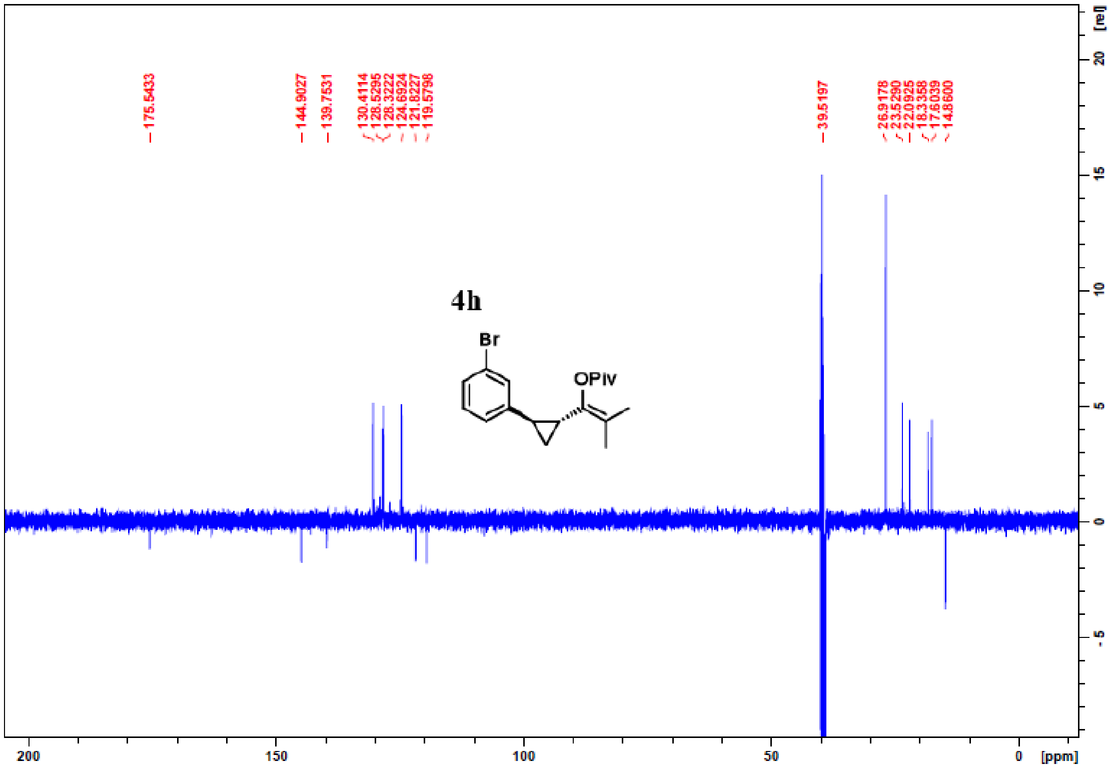

Figure S144. ^{13}C{1H} NMR (125 MHz) spectrum of **4h** in DMSO-d_6

***1*-[*cis*-*2*-(o-tolyl)cyclopropyl]-*2*-methylprop-*1*-enyl-*2*,*2*-dimethylpropanoate (3i)**

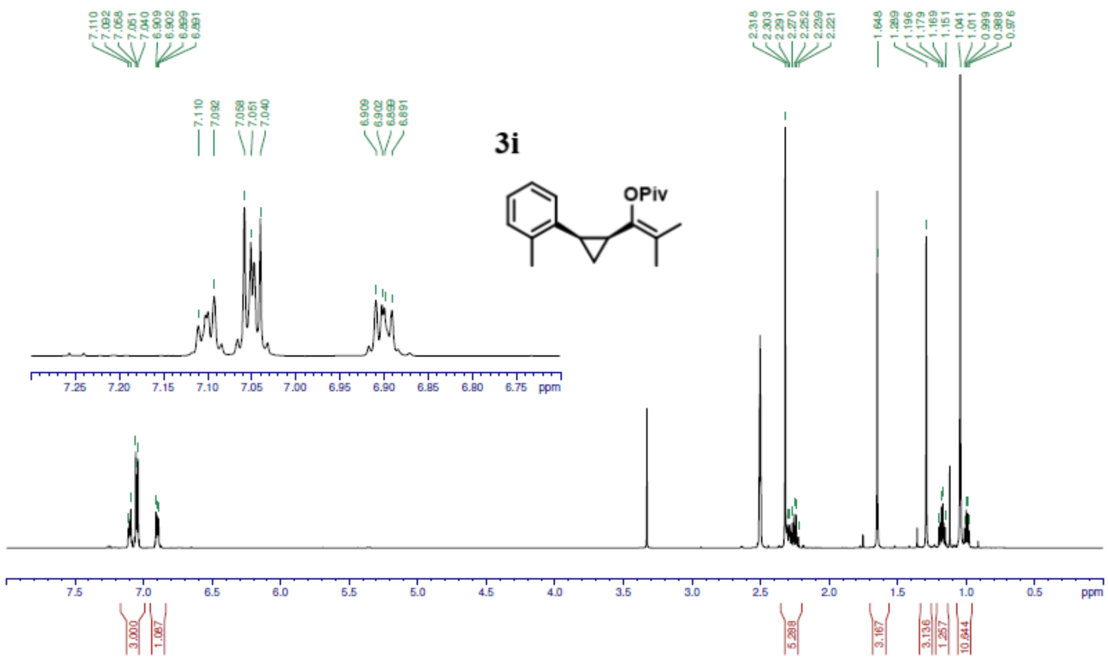

Figure S145. ^{1}H NMR (500 MHz) spectrum of **3i** in DMSO-d_6

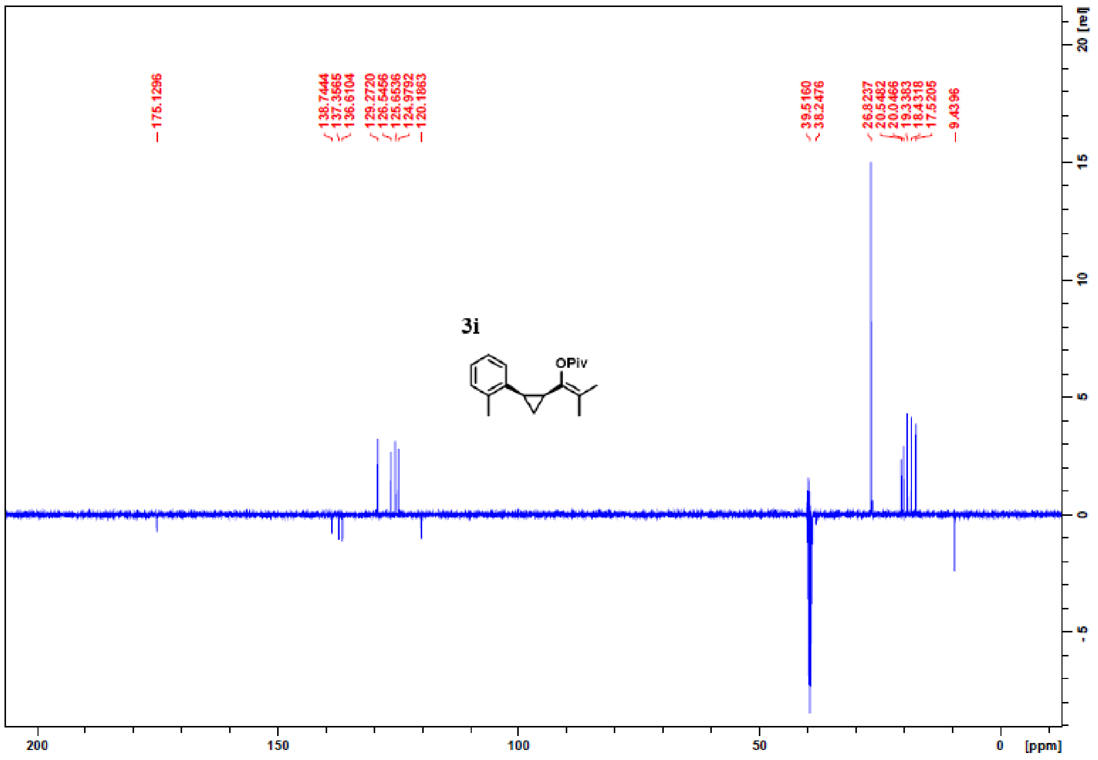

Figure S146. $^{13}C\{1H\}$ NMR (125 MHz) spectrum of **3i** in DMSO-d_6

***1*-[*trans*-*2*-(o-tolyl)cyclopropyl]-*2*-methylprop-*1*-enyl-*2*,*2*-dimethylpropanoate (4i)**

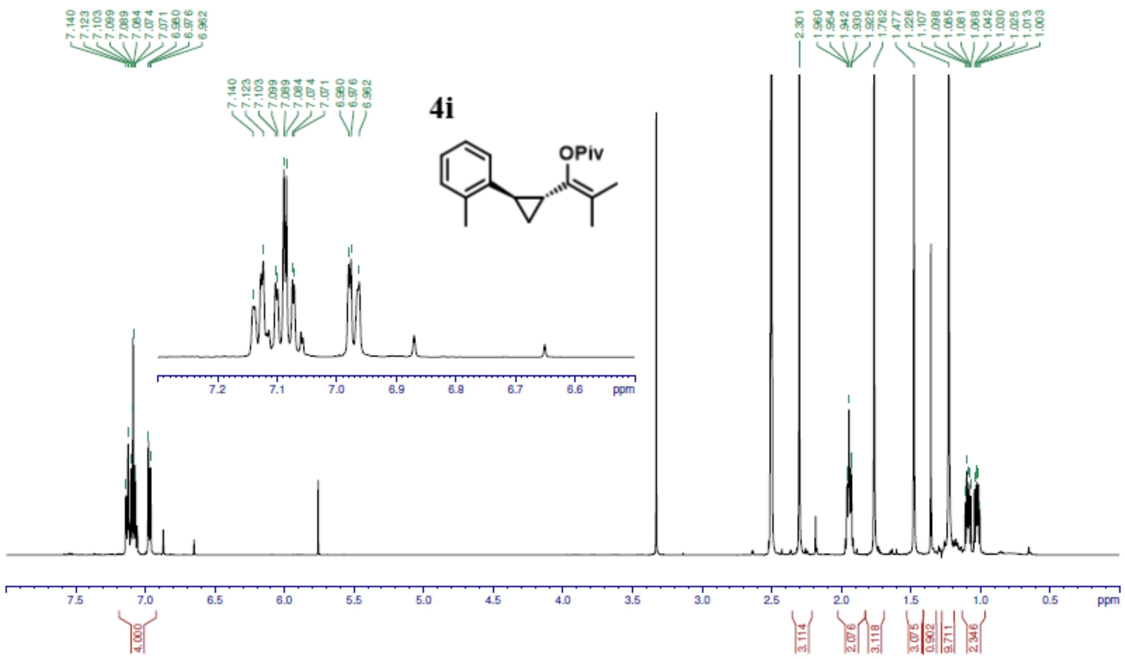

Figure S147. ^{1}H NMR (500 MHz) spectrum of **4i** in DMSO-d_6

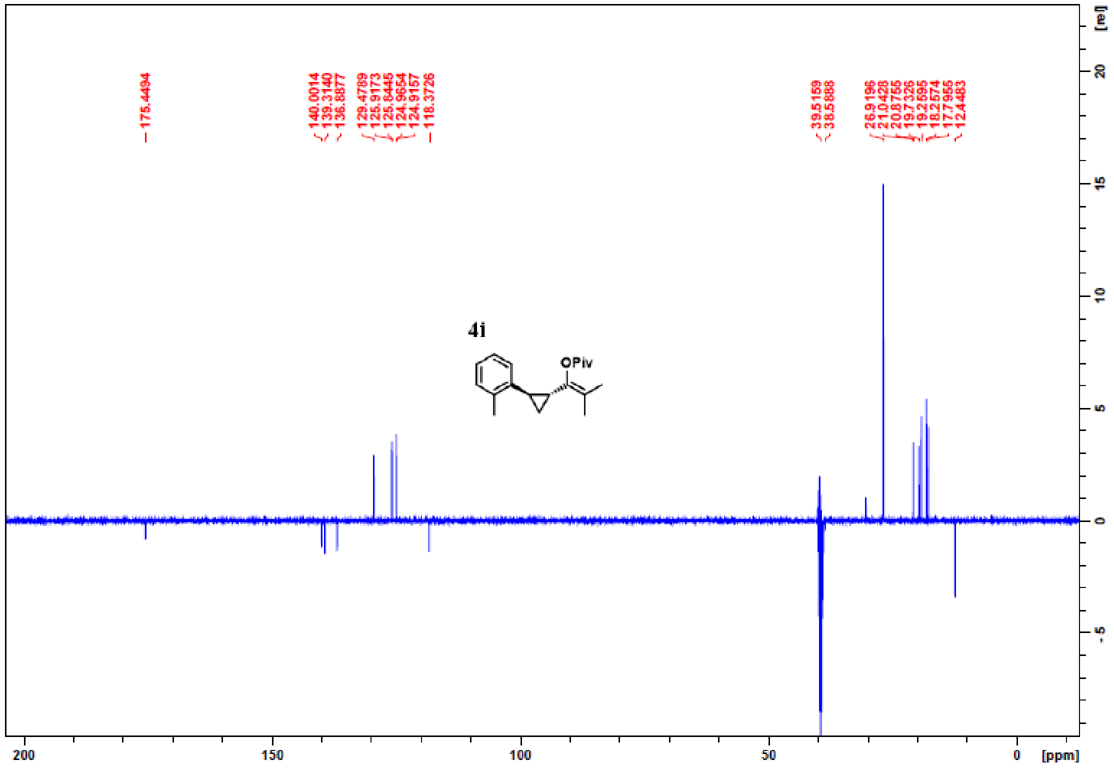

Figure S148. ^{13}C{1H} NMR (125 MHz) spectrum of **4i** in DMSO-d_6

***1*-[*cis*-*2*-(*4*-nitrophenyl)cyclopropyl]-*2*-methylprop-*1*-enyl-*2*,*2*-dimethylpropanoate (3j)**

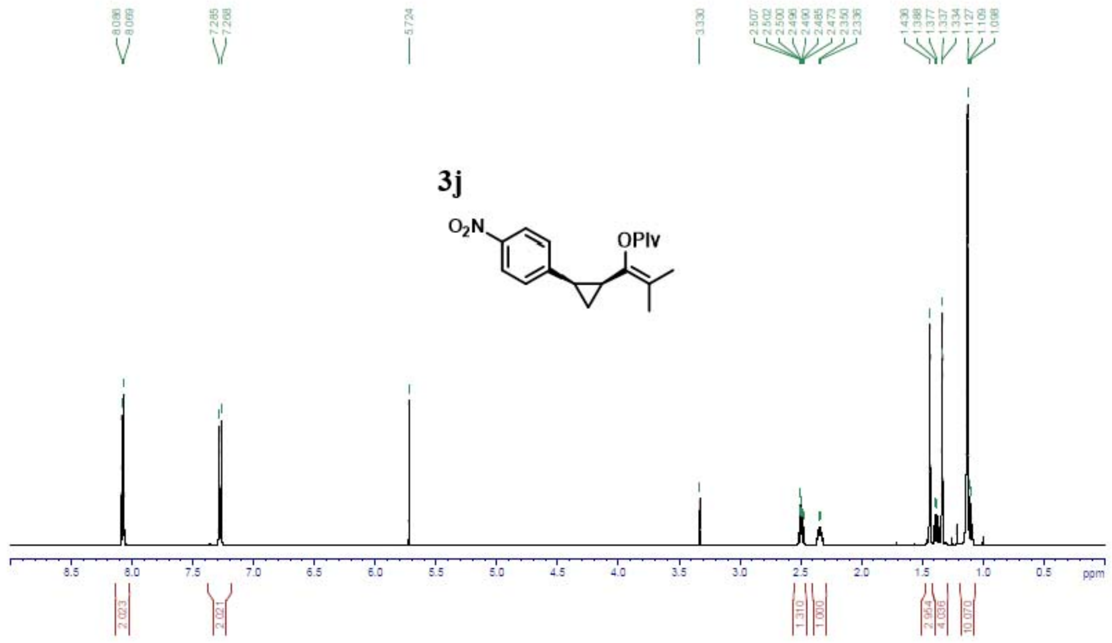

Figure S149. ^{1}H NMR (500 MHz) spectrum of **3j** in DMSO-d_6

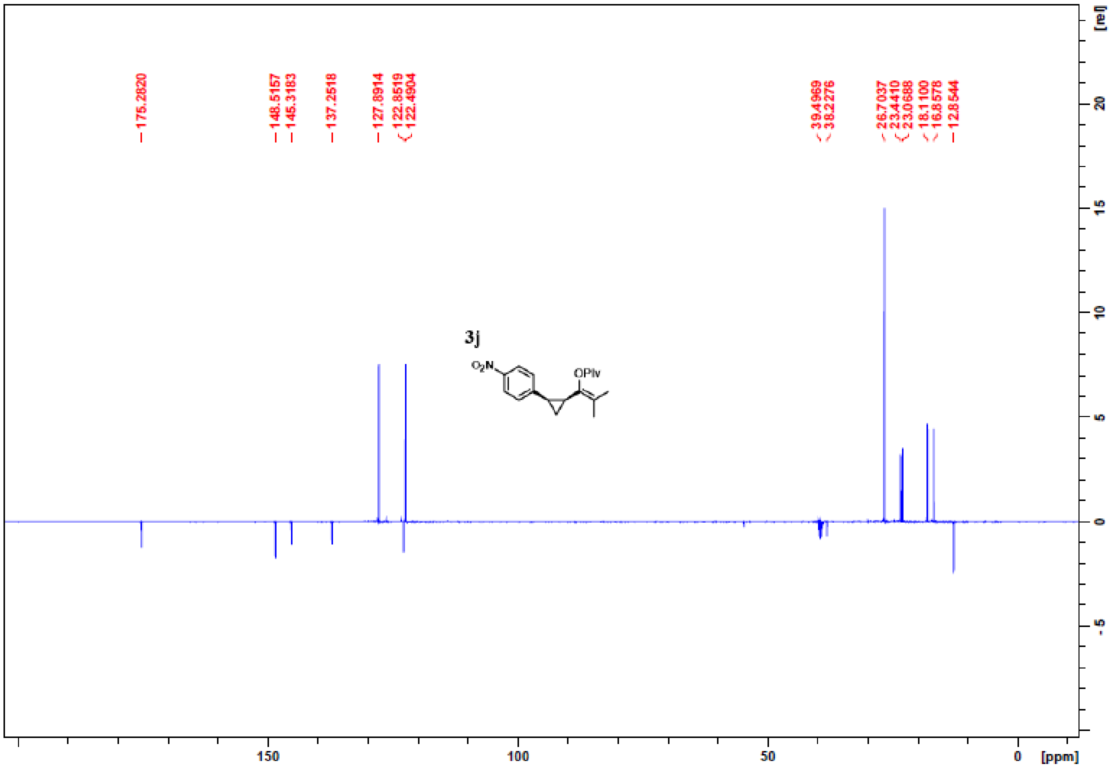

Figure S150. ^{13}C{1H} NMR (125 MHz) spectrum of **3j** in DMSO-d_6

***1*-[*trans*-*2*-(*4*-nitrophenyl)cyclopropyl]-*2*-methylprop-*1*-enyl-*2*,*2*-dimethylpropanoate (4j)**

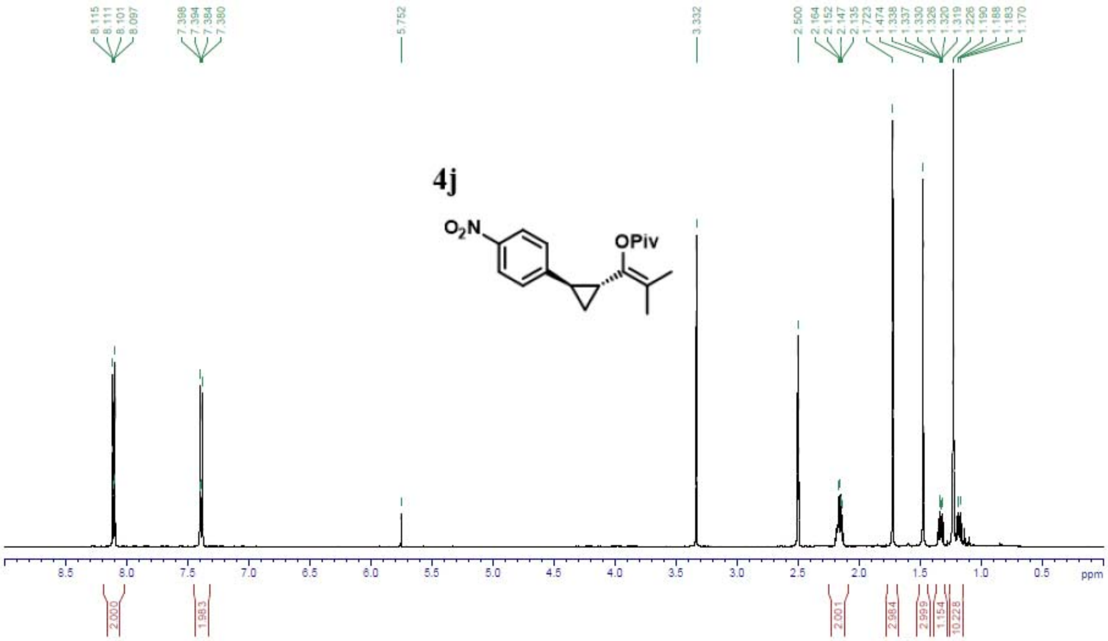

Figure S151. ^{1}H NMR (500 MHz) spectrum of **4j** in DMSO-d_6

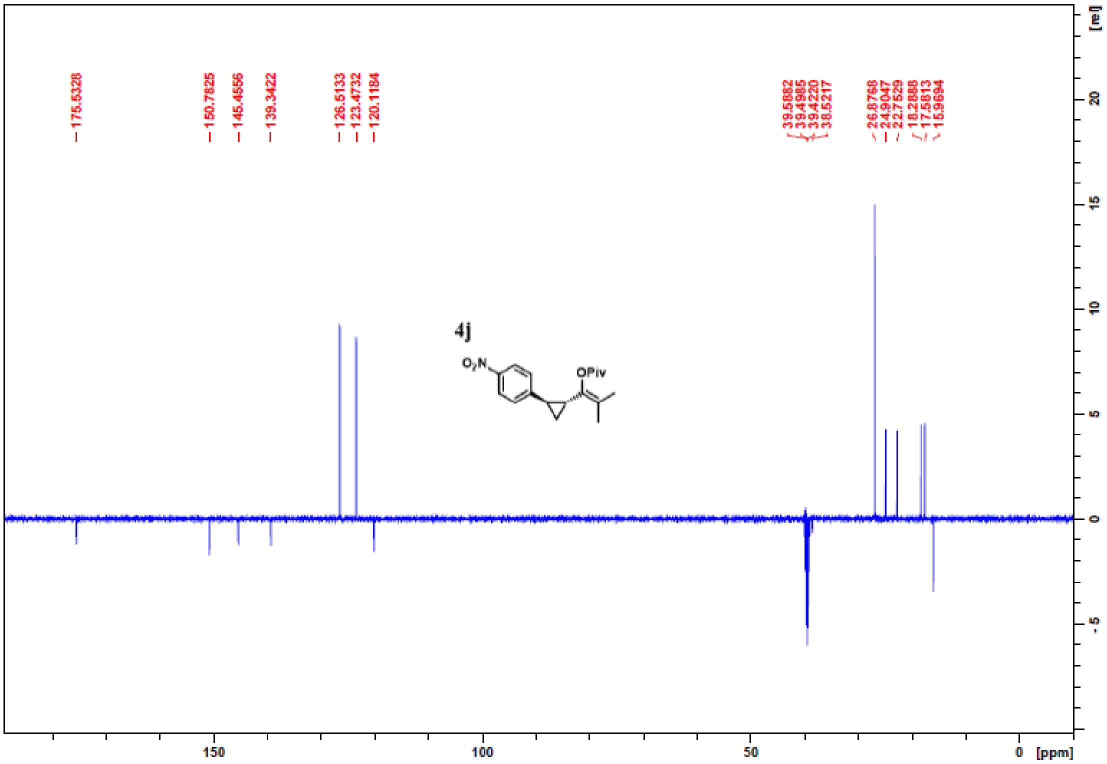

Figure S152. ^{13}C{1H} NMR (125 MHz) spectrum of **4j** in DMSO-d_6

***1*-[*cis*-*2*-phenylcyclopropyl]-*2*-methylprop-*1*-enyl-*2*,*2*-dimethylpropanoate (3k)**

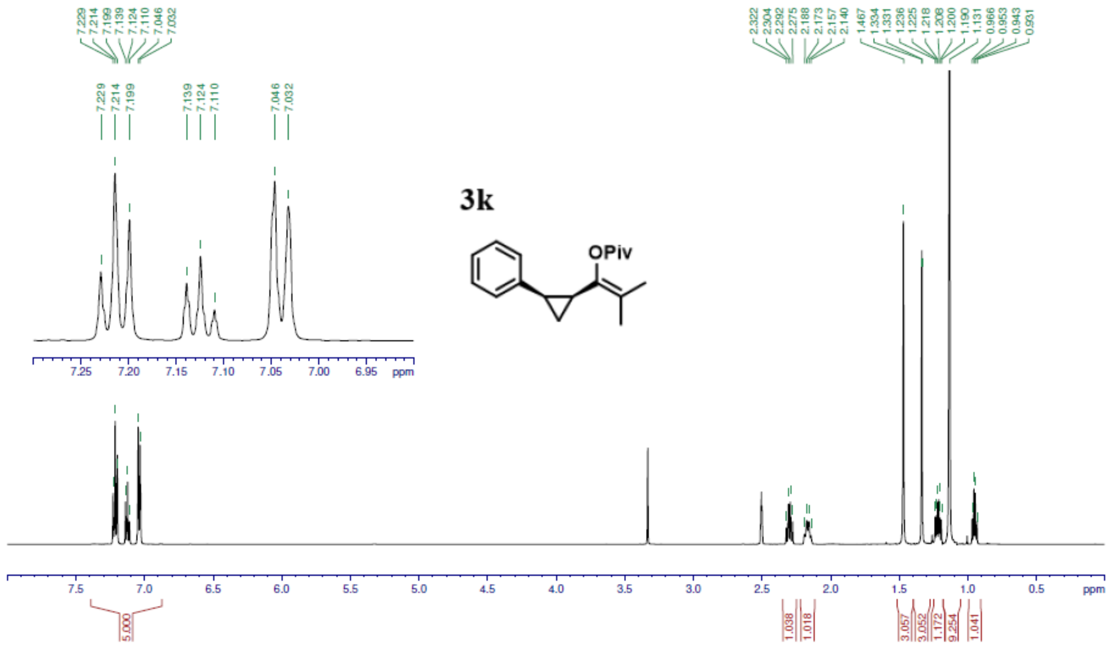

Figure S153. ^{1}H NMR (500 MHz) spectrum of **3k** in DMSO-d_6

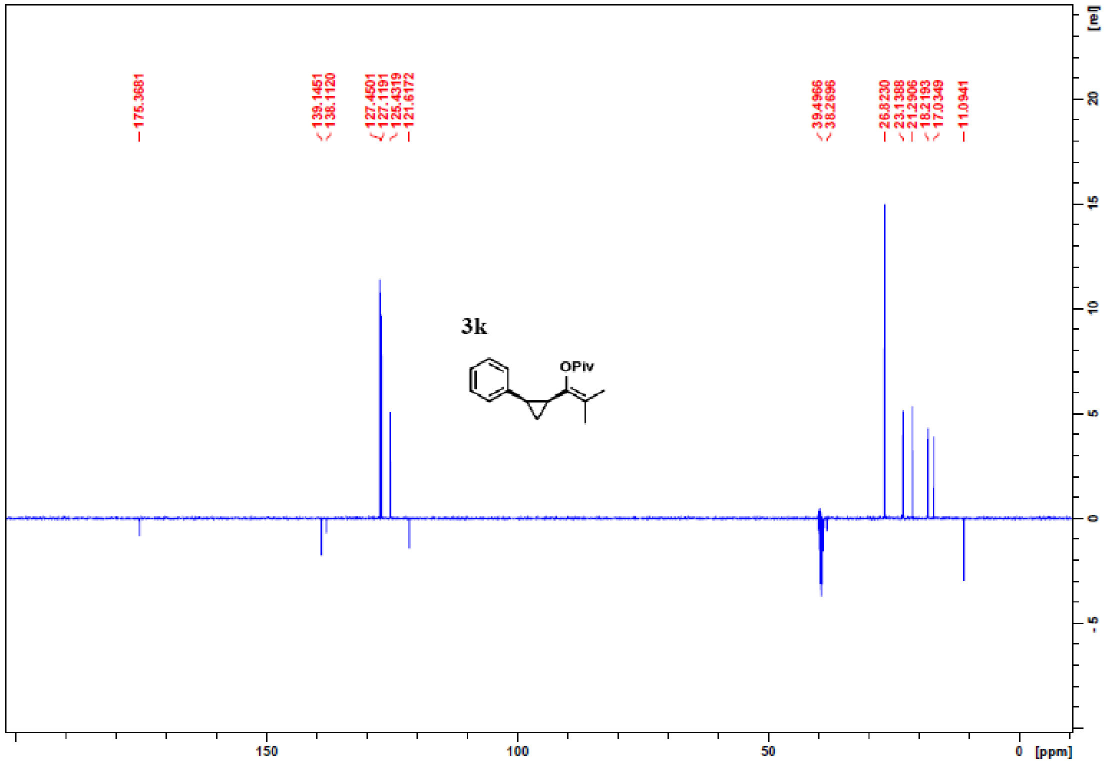

Figure S154. ^{13}C{1H} NMR (125 MHz) spectrum of **3k** in DMSO-d_6

1-[***cis***-***2***-methyl-***3***-phenylcyclopropyl]-***2***-methylprop-***1***-enyl-***2,2***-dimethylpropanoate (3l)**

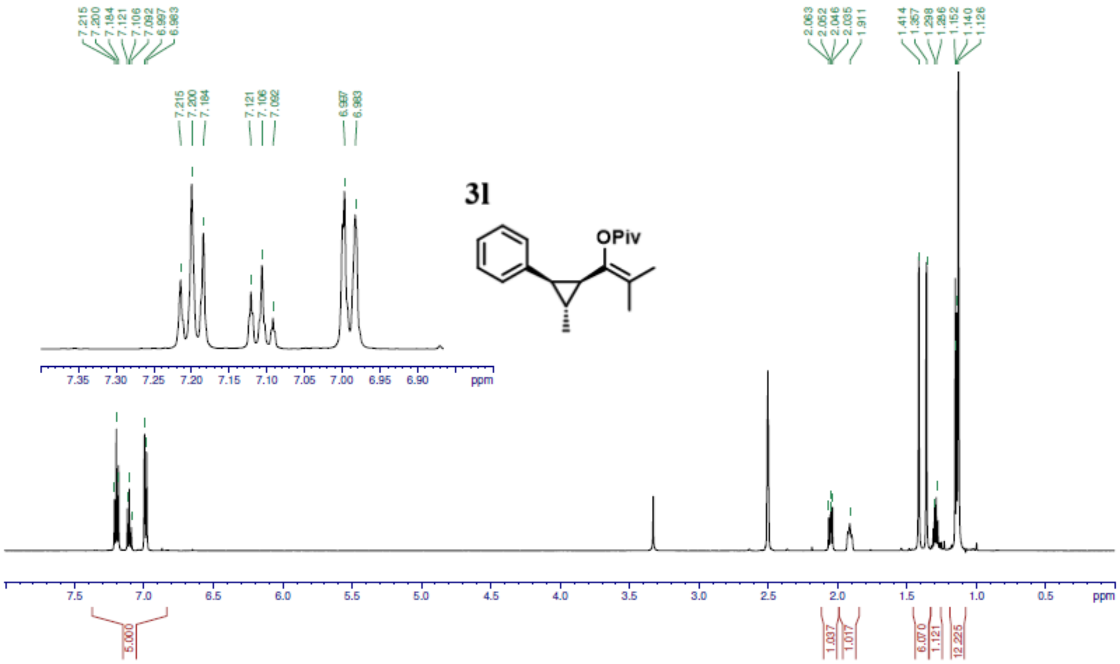

Figure S155. ^{1}H NMR (500 MHz) spectrum of **3l** in DMSO-d_6

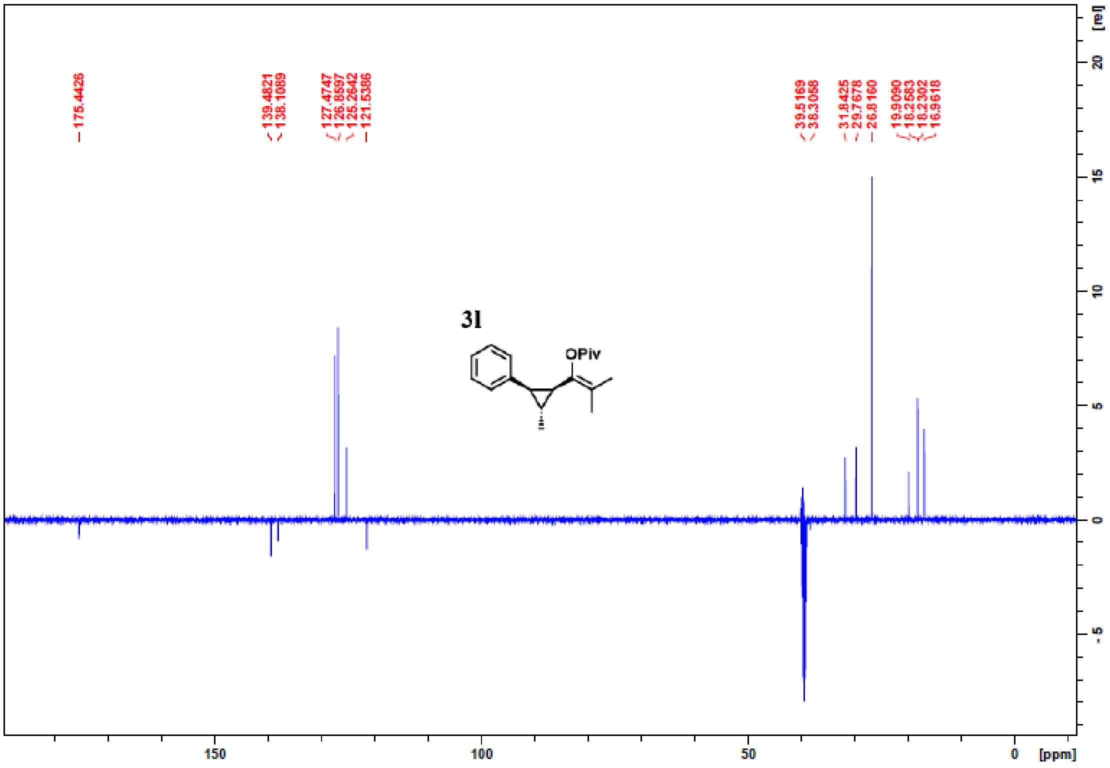

Figure S156. ^{13}C{1H} NMR (125 MHz) spectrum of **3l** in DMSO-d_6

***1*-[*trans*-*2*-methyl-*3*-phenylcyclopropyl]-*2*-methylprop-*1*-enyl]-*2*,*2*-dimethylpropanoate (4l)**

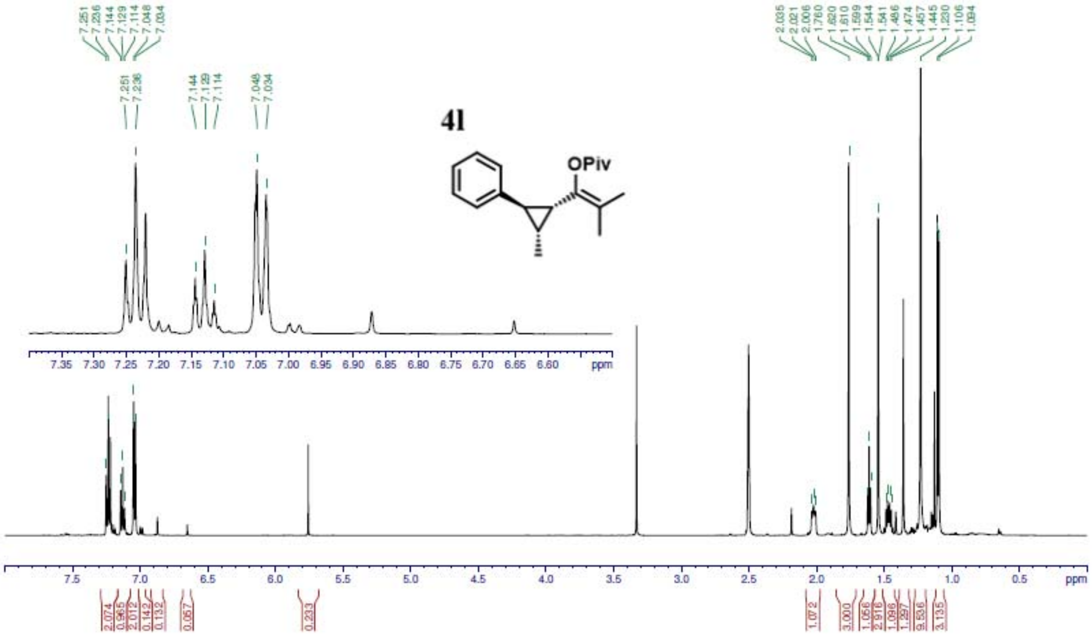

Figure S157. ^{1}H NMR (500 MHz) spectrum of **4l** in DMSO-d_6

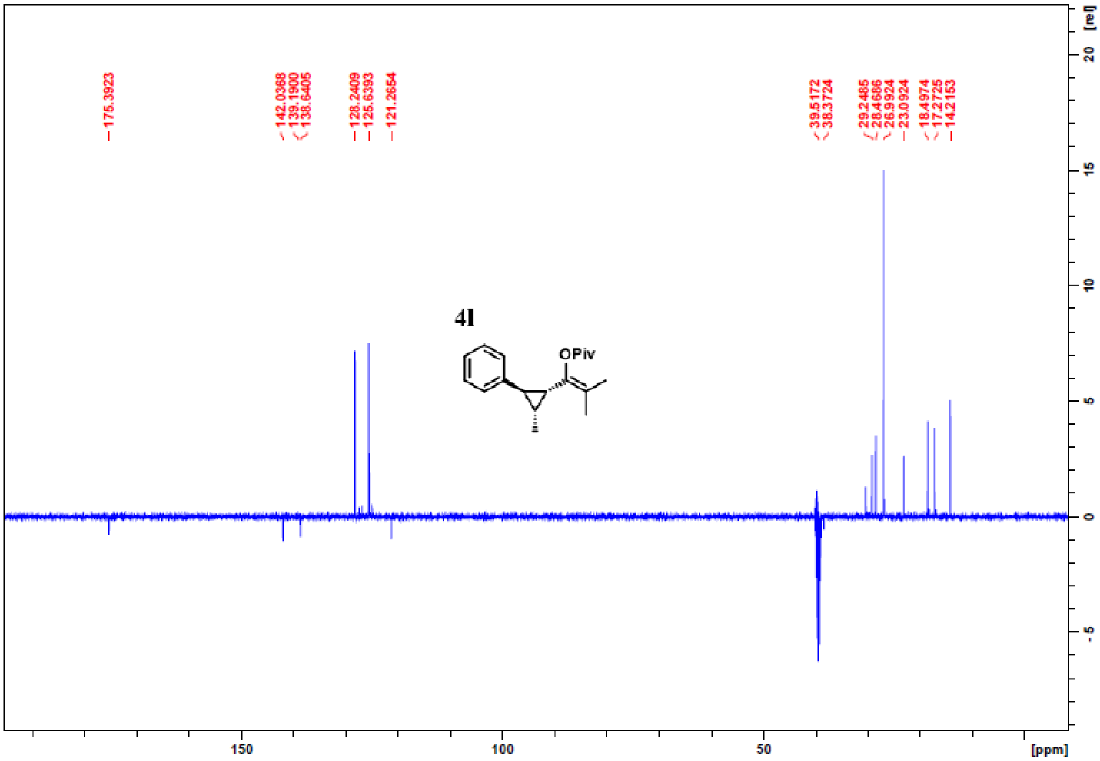

Figure S158. ^{13}C{1H} NMR (125 MHz) spectrum of **4l** in DMSO-d_6

***1*-[cis-(*trans*-*2*,*3*-diphenylcyclopropyl)]-*2*-methyl-prop-*1*-enyl-*2*,*2*-dimethylpropanoate (3m)**

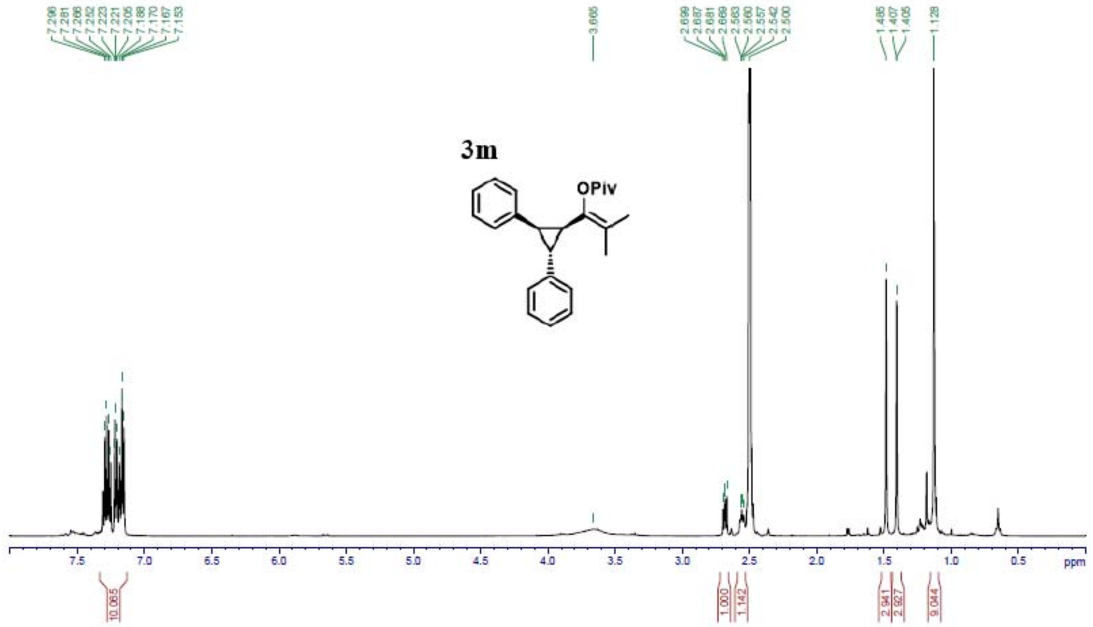

Figure S159. ^{1}H NMR (500 MHz) spectrum of **3m** in DMSO-d_6

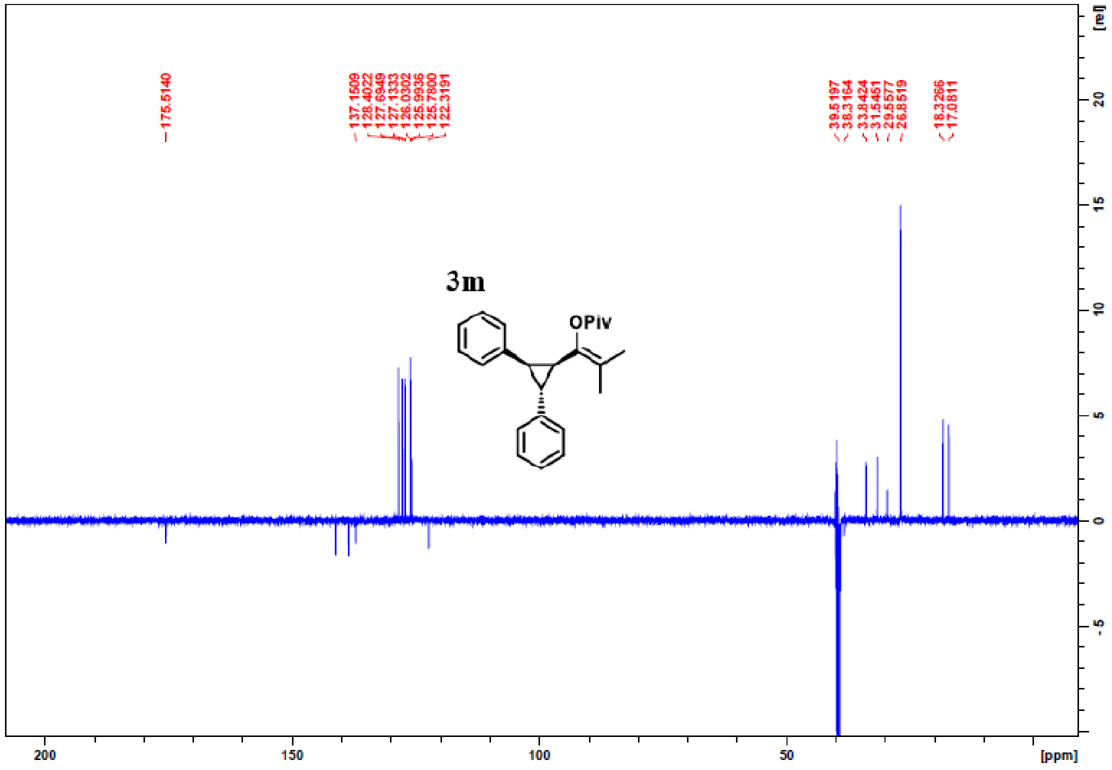

Figure S160. ^{13}C{1H} NMR (125 MHz) spectrum of **3m** in DMSO-d_6

[(*Z*)-*1*-[cyclohexylidene-[*2*-(*2*-methoxy-*2*-oxo-ethyl)-*3*-phenyl-cyclopropyl]methyl]-*2,2*-dimethylpropanoate (3n)

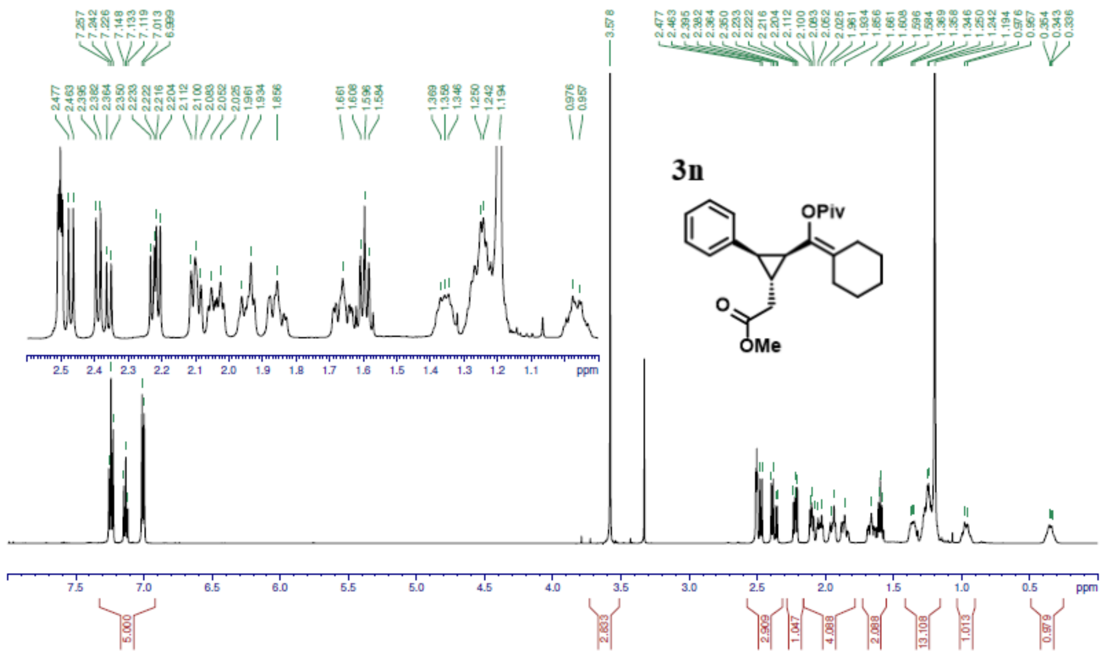

Figure S161. ^{1}H NMR (500 MHz) spectrum of **3n** in DMSO-d_6

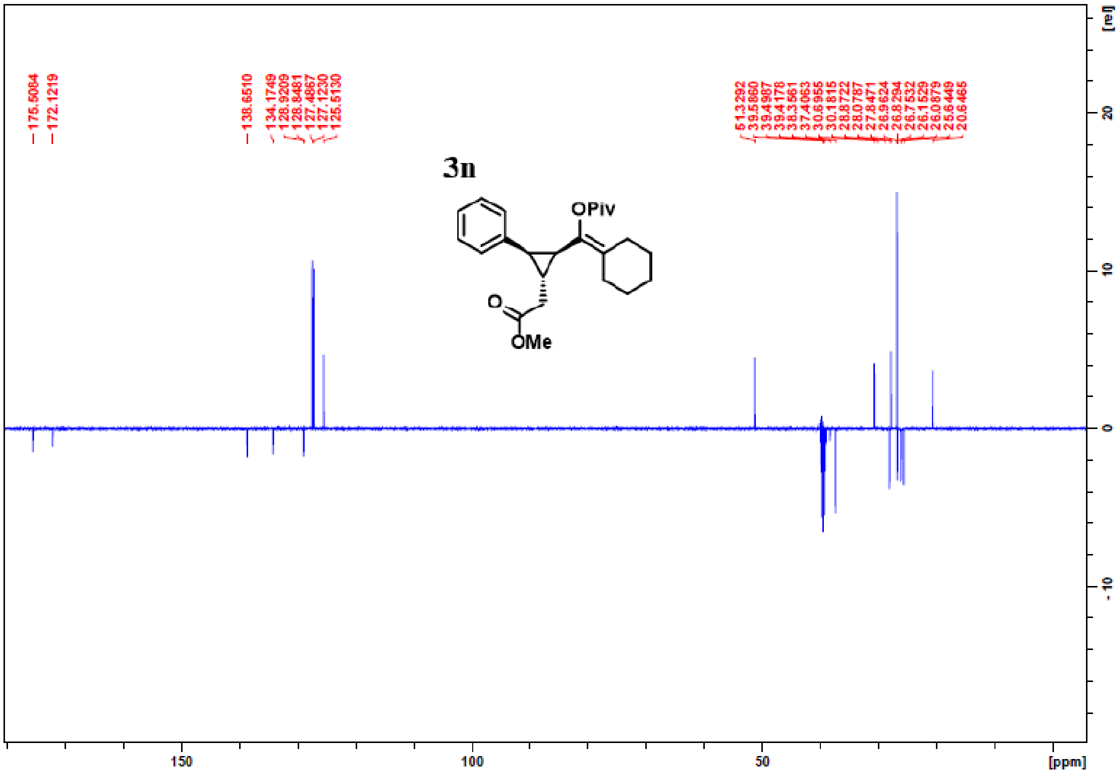

Figure S162. $^{13}C\{^{1}H\}$ NMR (125 MHz) spectrum of **3n** in DMSO-d_6

***1*-[*cis*-*2*-acetoxy-*2*-phenyl-cyclopropyl]-*2*-methyl-prop-*1*-enyl *2,2*-dimethylpropanoate (3o)**

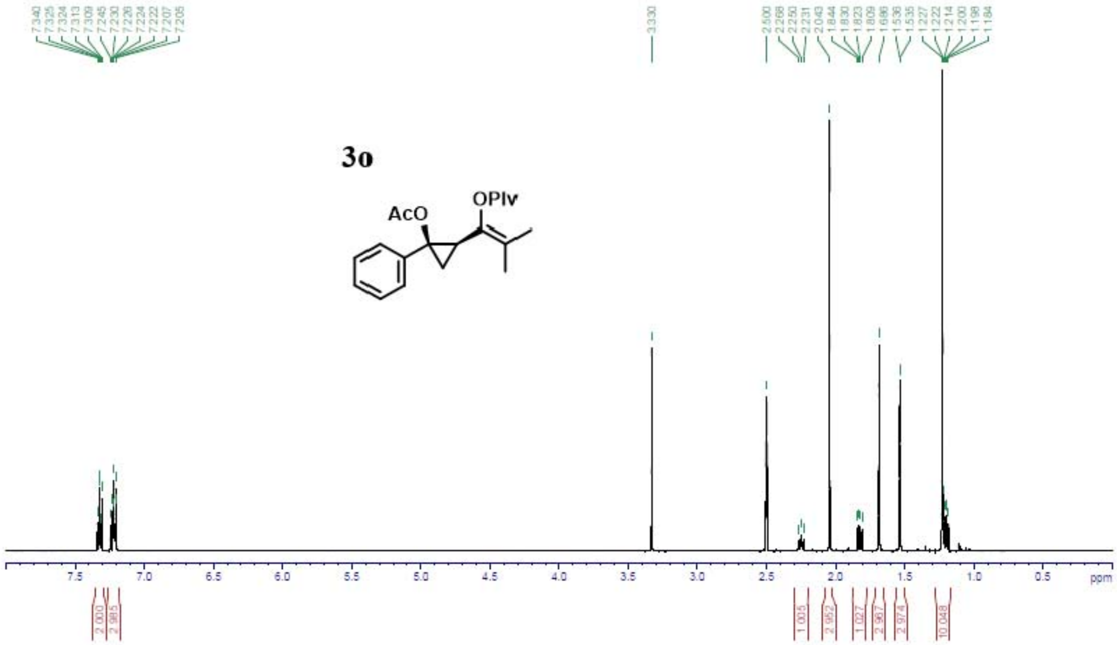

Figure S163. ^{1}H NMR (500 MHz) spectrum of **3o** in DMSO-d_6

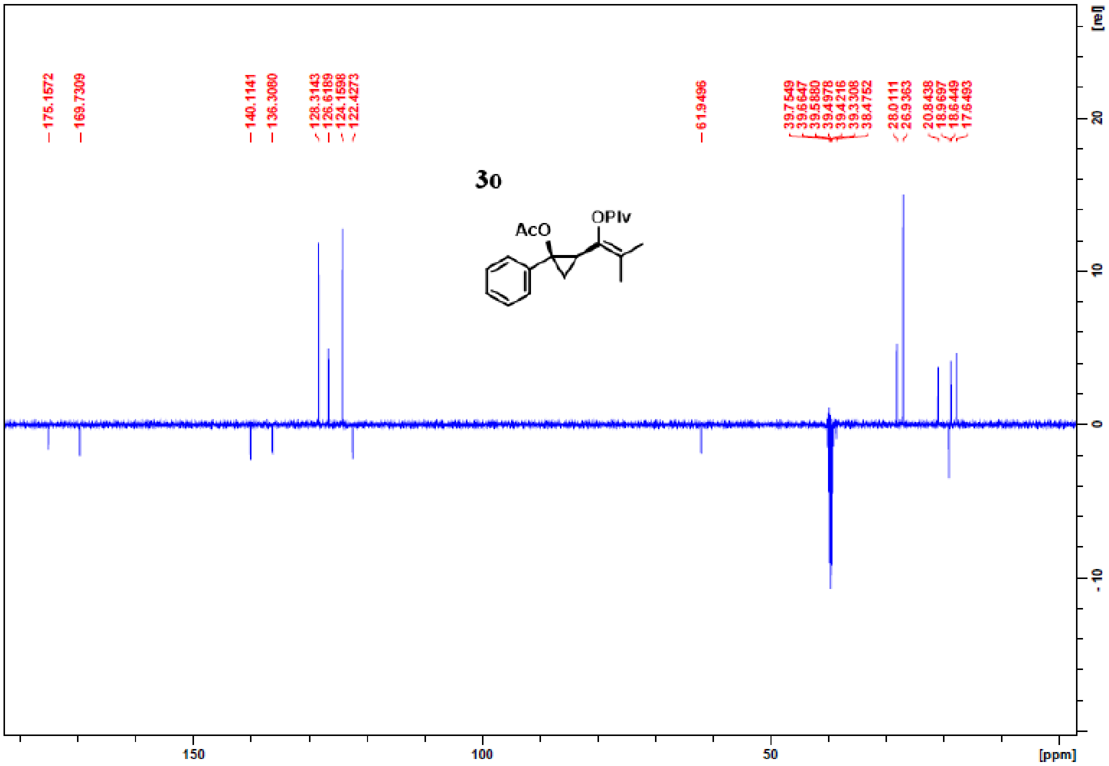

Figure S164. ^{13}C{1H} NMR (125 MHz) spectrum of **3o** in DMSO-d_6

***1*-[*trans*-*2*-acetoxy-*2*-phenyl-cyclopropyl]-*2*-methyl-prop-*1*-enyl *2,2*-dimethylpropanoate (4o)**

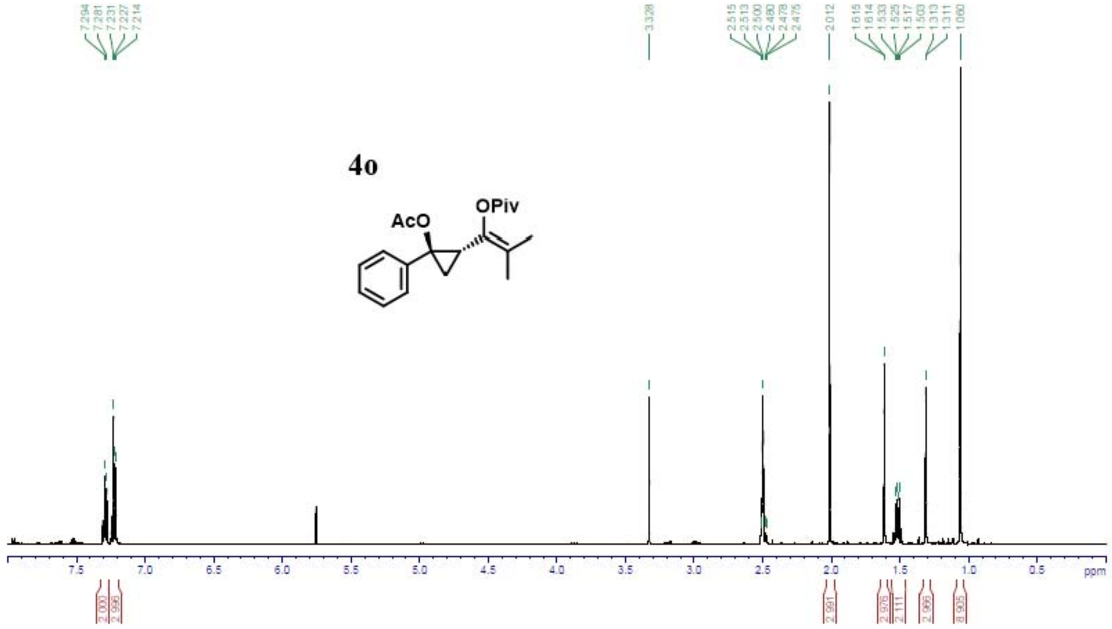

Figure S165. ^{1}H NMR (500 MHz) spectrum of **4o** in DMSO-d_6

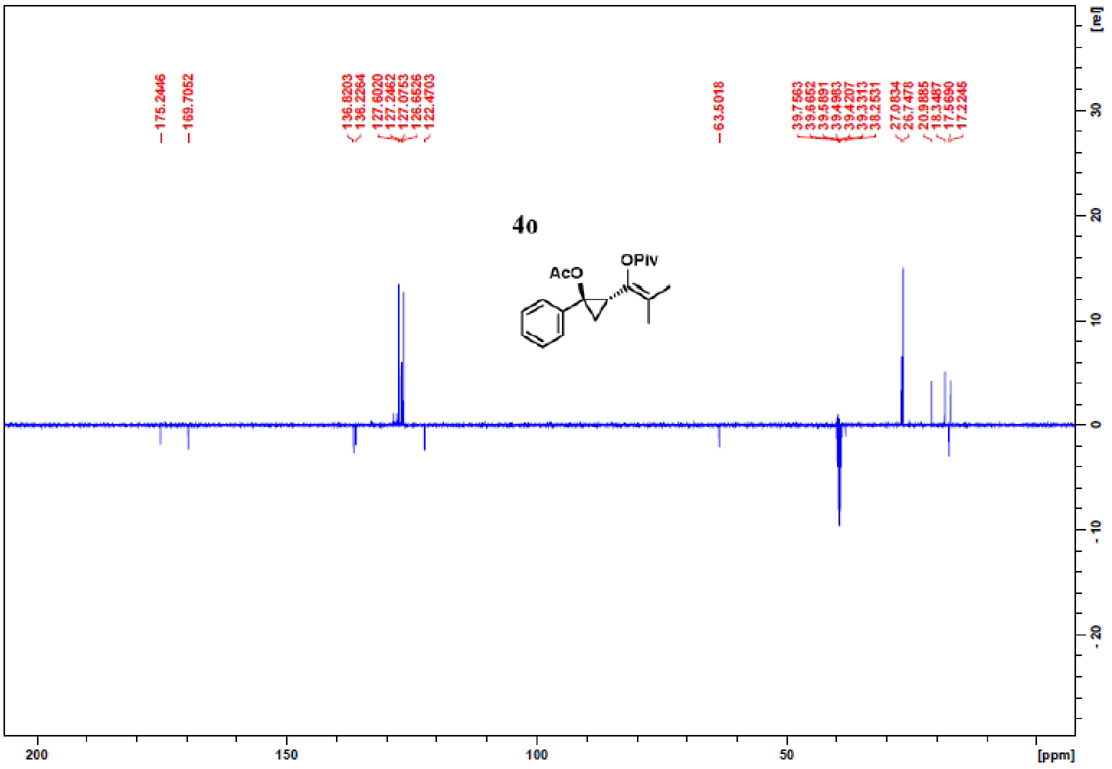

Figure S166. ^{13}C{1H} NMR (125 MHz) spectrum of **4o** in DMSO-d_6

[(*Z*)-*2*-methyl-*1*-[norcaran-*7*-yl]prop-*1*-enyl]-*2*,*2*-dimethylpropanoate (3p)

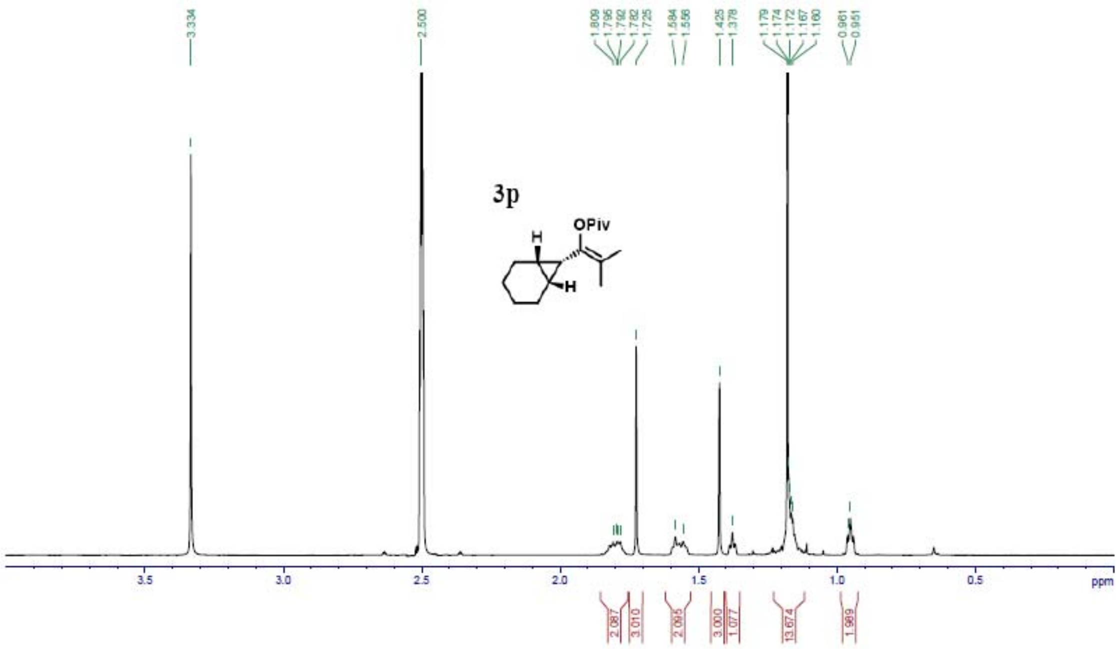

Figure S167. ^{1}H NMR (500 MHz) spectrum of **3p** in DMSO-d_6

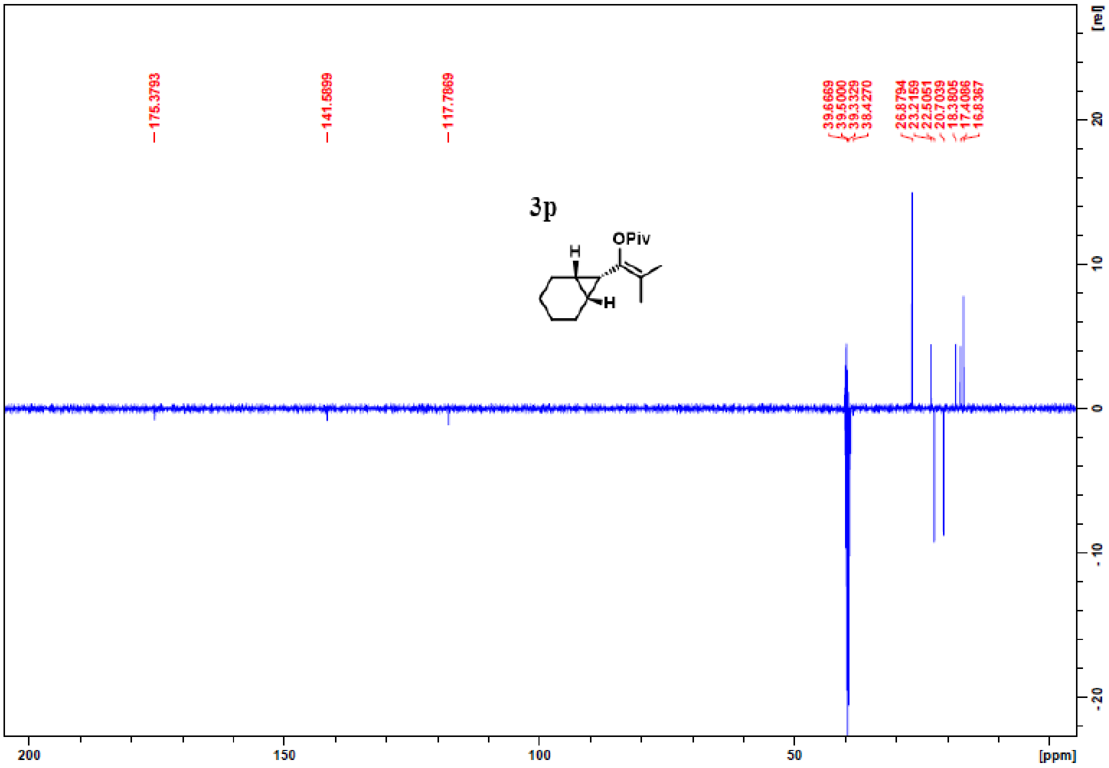

Figure S168. $^{13}C\{1H\}$ NMR (125 MHz) spectrum of **3p** in DMSO-d_6

***1*-[*cis*-*2*-(*2*-methoxy-*2*-oxo-ethyl)-*3*-phenyl-cyclopropyl]-cyclohexylidene-*2*,*2*-dimethylpropanoate (3q) and *1*-[*trans*-*2*-(*2*-methoxy-*2*-oxo-ethyl)-*3*-phenyl-cyclopropyl]-cyclohexylidene-*2*,*2*-dimethylpropanoate (4q)**

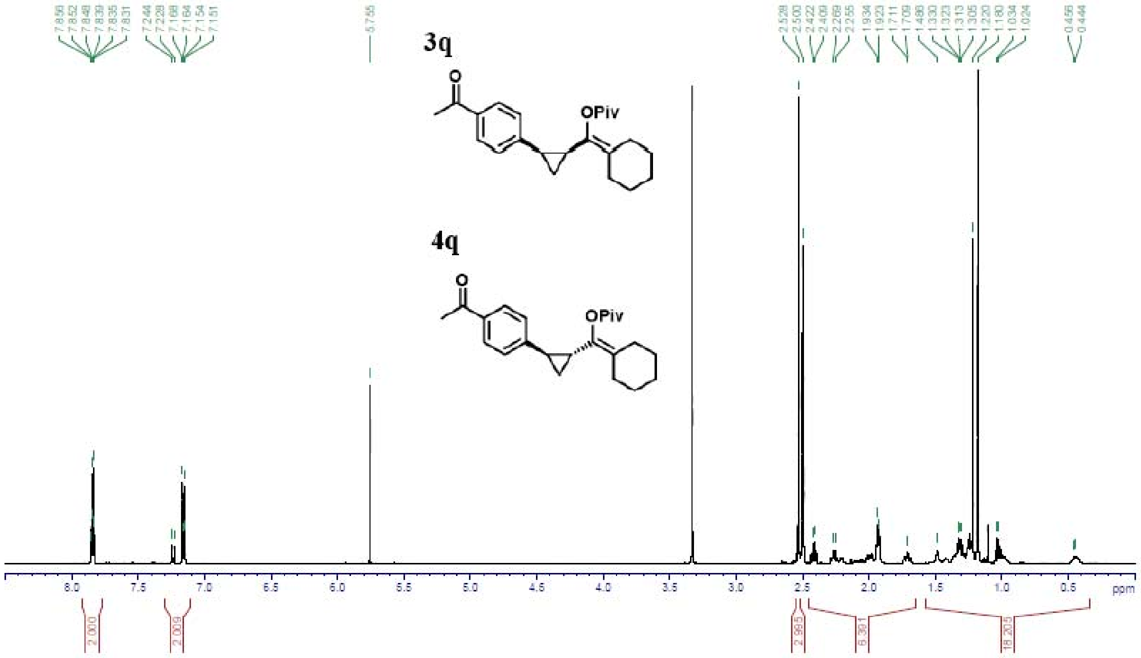

Figure S169. ^{1}H NMR (500 MHz) spectrum of **3q-4q** in DMSO-d_6

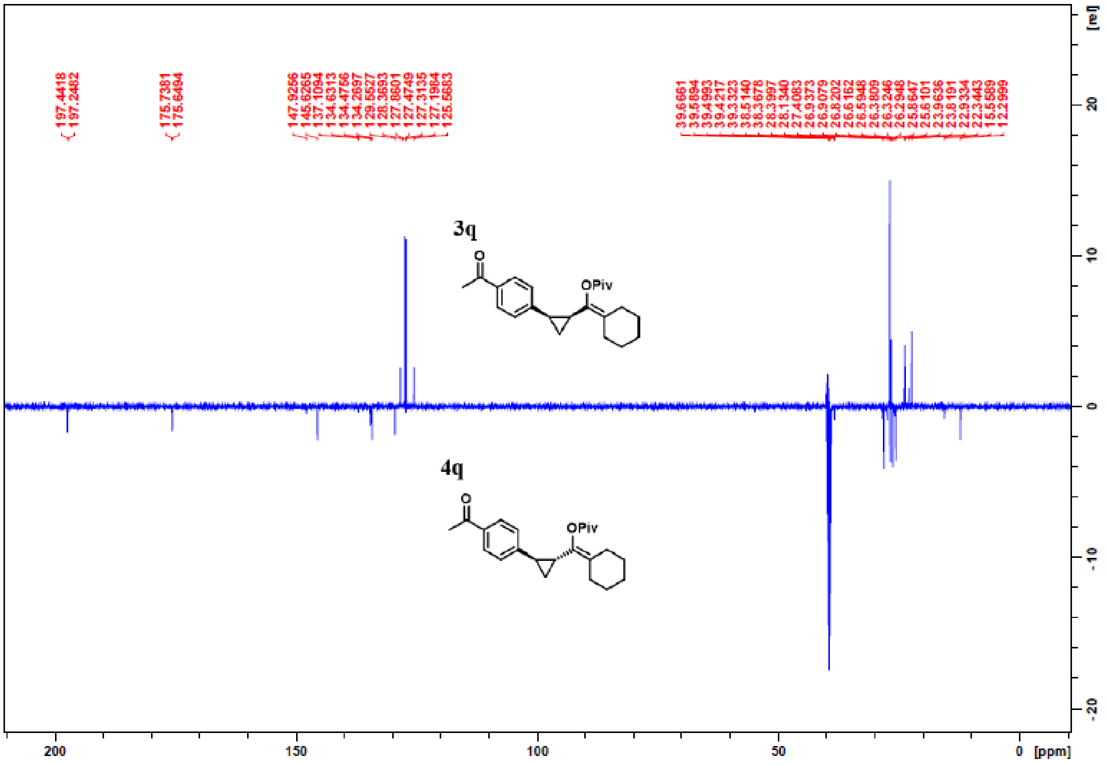

Figure S170. ^{13}C{1H} NMR (125 MHz) spectrum of **3q-4q** in DMSO-d_6

Cis-[(E)-1-[2-cyclopentylcyclopropyl]-2-phenyl-vinyl]-2,2-dimethylpropanoate (3r)

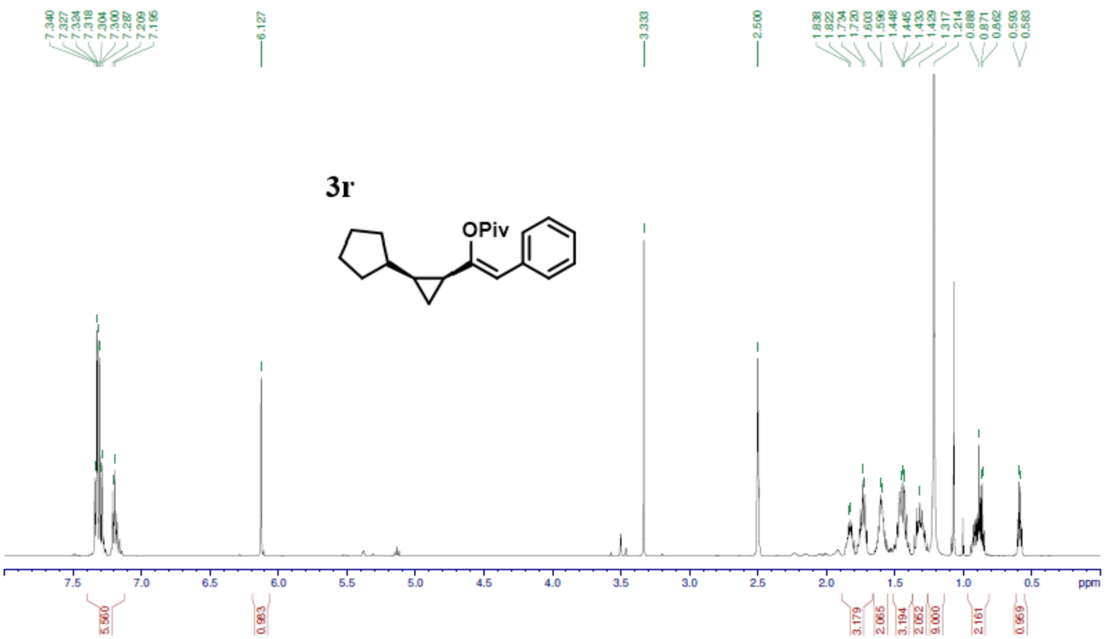

Figure S171. ^{1}H NMR (500 MHz) spectrum of **3r** in DMSO-d_6

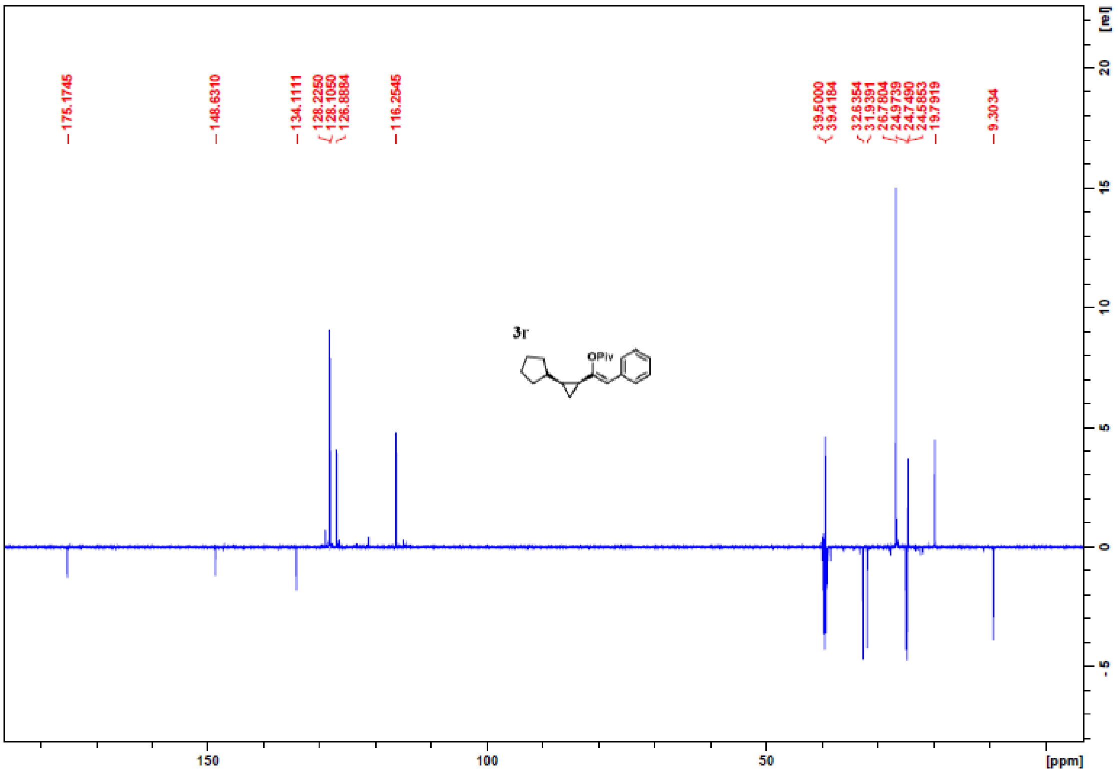

Figure S172. $^{13}C\{1H\}$ NMR (125 MHz) spectrum of **3r** in DMSO-d_6

***Trans*-[(*E*)-*1*-[*2*-cyclopentylcyclopropyl]-*2*-phenyl-vinyl]-*2,2*-dimethylpropanoate (4r)**

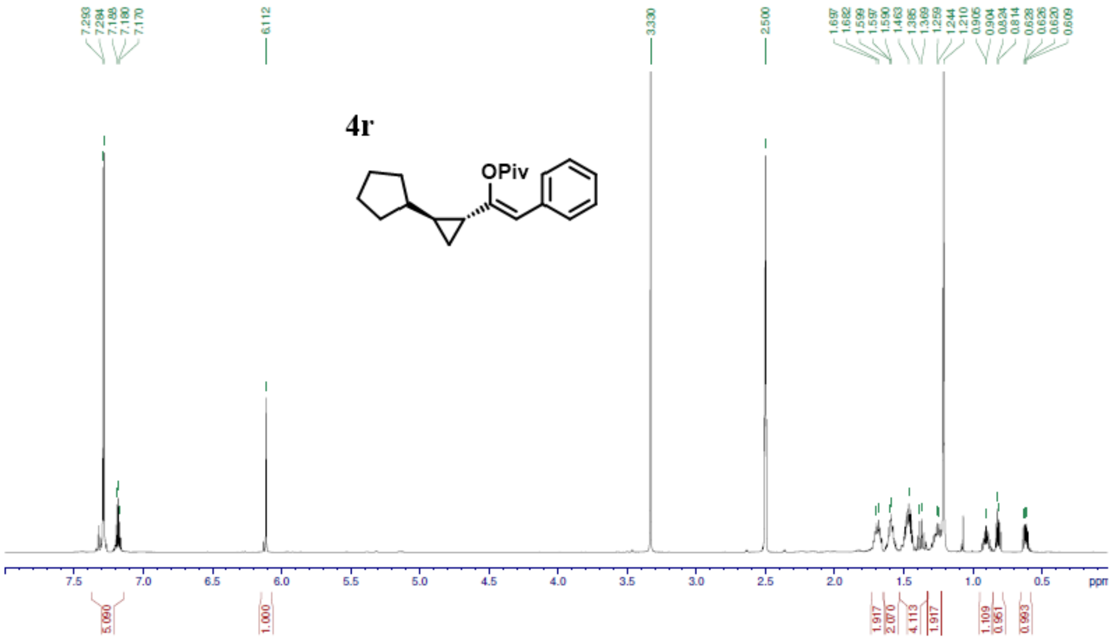

Figure S173. ^{1}H NMR (500 MHz) spectrum of **4r** in DMSO-d_6

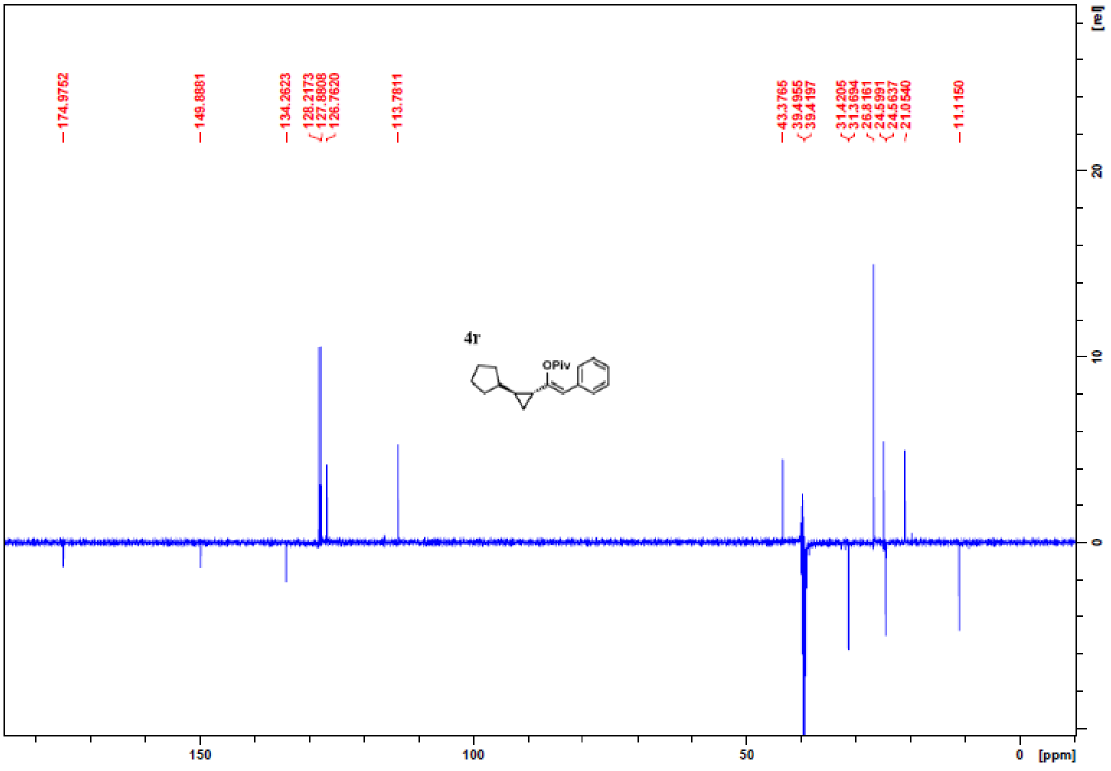

Figure S174. ^{13}C{1H} NMR (125 MHz) spectrum of **4r** in DMSO-d_6

***Cis*-tert-Butyl-2-[(Z)-2-acetoxy-2-[2-(3-bromophenyl)cyclopropyl]vinyl]benzoate (3s) and *Trans*-tert-Butyl-*3*-[(*Z*)-*2*-acetoxy-*2*-[*2*-(*3*-bromophenyl)cyclopropyl]vinyl]benzoate (4s)**

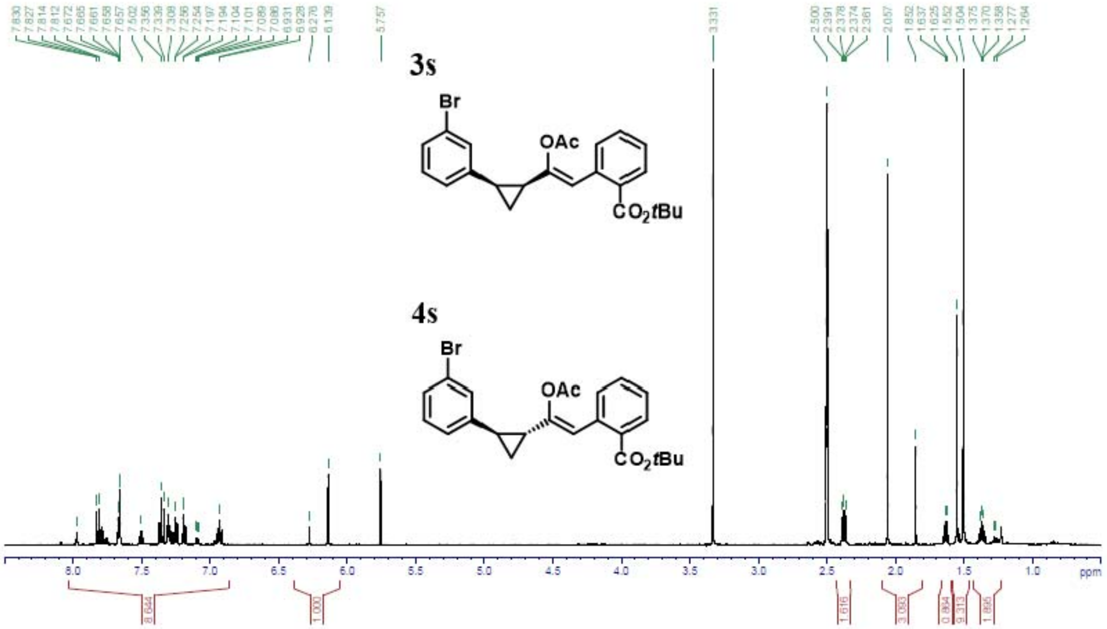

Figure S175. ^{1}H NMR (500 MHz) spectrum of **3s-4s** in DMSO-d_6

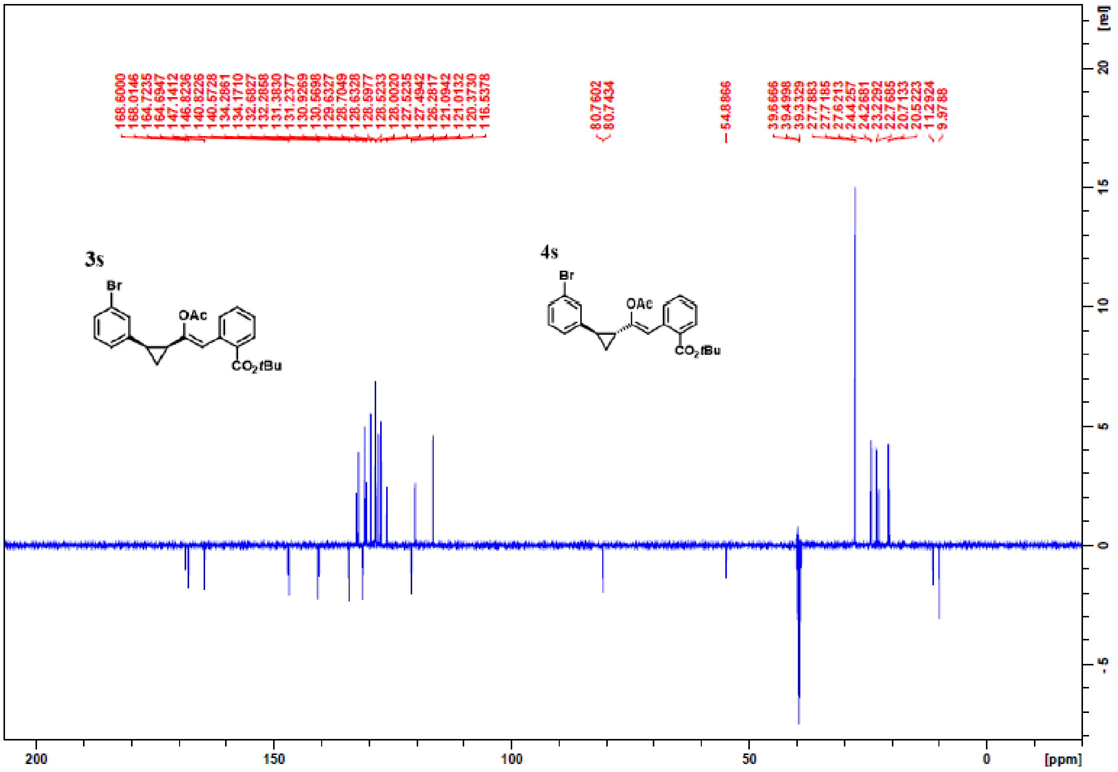

Figure S176. ^{13}C{1H} NMR (125 MHz) spectrum of **3s-4s** in DMSO-d_6

***Cis*-{tert-Butyl-*3*-[(*Z*)-*2*-acetoxy-*2*-[*2*-(*2*-iodophenyl)cyclopropyl]vinyl]benzoate} (3t) and *Trans*-{tert-Butyl-*3*-[(*Z*)-*2*-acetoxy-*2*-[*2*-(*2*-iodophenyl)cyclopropyl]vinyl]benzoate} (4t)**

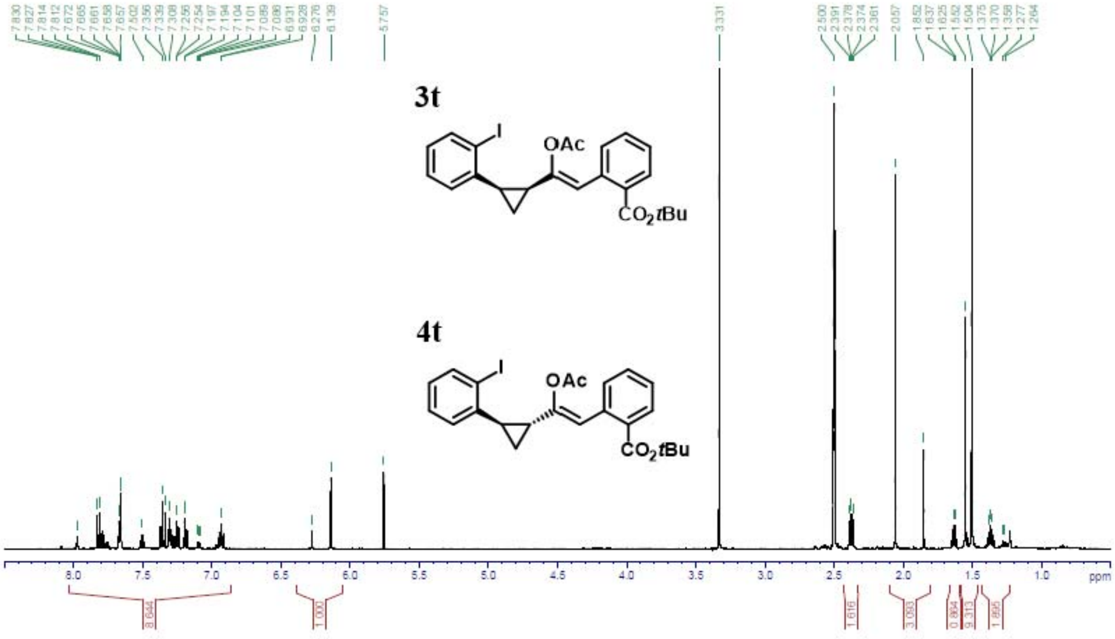

Figure S177. ^{1}H NMR (500 MHz) spectrum of **3t-4t** in DMSO-d_6

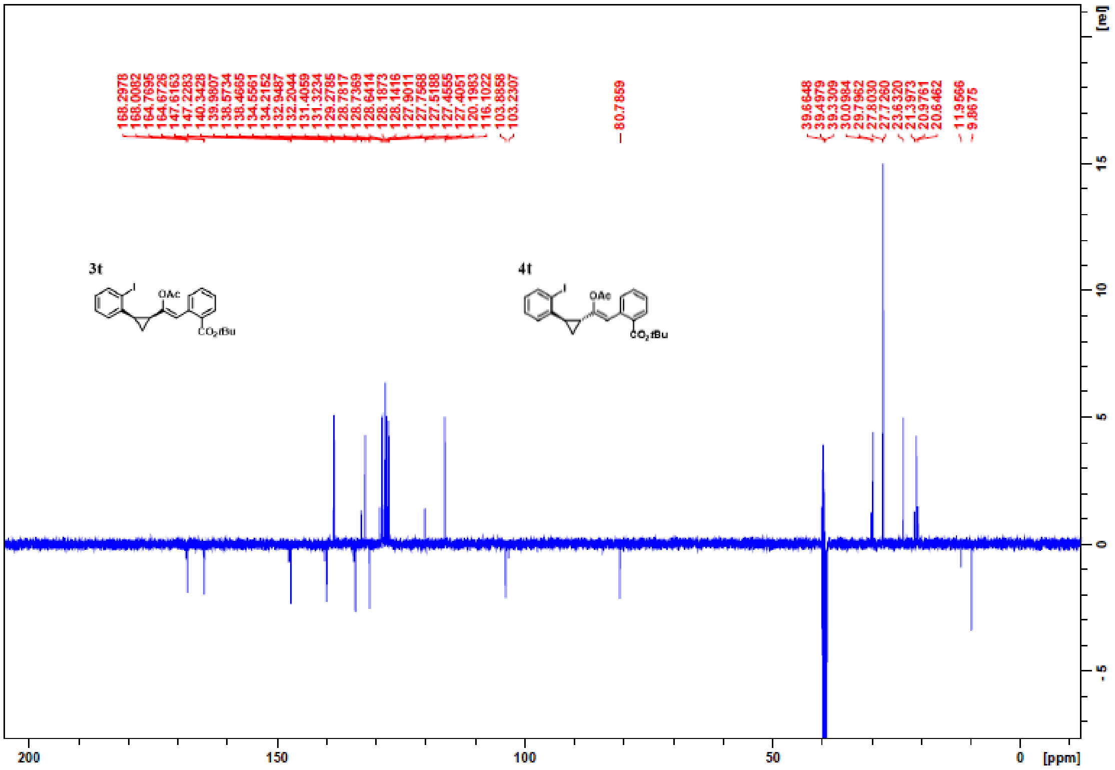

Figure S178. ^{13}C{1H} NMR (125 MHz) spectrum of **3t-4t** in DMSO-d_6
